# Additional data for evaluation of the excited state dipole moments of anisole

**DOI:** 10.1016/j.dib.2018.09.110

**Published:** 2018-10-03

**Authors:** Mirko Matthias Lindic, Matthias Zajonz, Marie-Luise Hebestreit, Michael Schneider, W. Leo Meerts, Michael Schmitt

**Affiliations:** a Heinrich-Heine-Universität, Institut für Physikalische Chemie I, D-40225 Düsseldorf, Germany; b Radboud University, Institute for Molecules and Materials, Felix Laboratory, Toernooiveld 7c, 6525 ED Nijmegen, the Netherlands

## Abstract

We present the temperature dependent density, fluorescence emission and absorption spectroscopic data, that are needed for an evaluation of the excited state dipole moment of anisole in ethyl acetate via the methods of thermochromic shifts. Furthermore, the rotationally resolved electronic Stark spectrum of anisole in the molecular beam is presented. Finally, the Cartesian coordinates of the CC2/cc-pVTZ optimized structures of anisole are given in bohr units. For details about the evaluation of the dipole moments from the given data, see the connected research article: Lindic et al. (2018) [1].

**Specifications table**

**Table:** 

Subject area	*Physics, Chemistry*
More specific subject area	*Molecular Spectroscopy, Physical Chemistry*
Type of data	*Table, text file, graph, figure*
How data was acquired	*High resolution electronic Stark spectroscopy. (Home-built apparatus)*
	*Determination of the cavity size using a density meter (Anton Paar DMA4500M)*
	*Thermochromic shifts of emission (Varian Cary Eclipse) and absorption spectra (Varian Cary 50 Scan UV spectrometer).*
Data format	*Raw and analyzed*
Experimental factors	*The chemicals used were of spectroscopic grade.*
Experimental features	*Gas phase excited state dipole moments of anisole are obtained from CW rotationally resolved electronic Stark spectra in a molecular beam. Solution values are obtained from thermochromic shifts of the fluorescence emission and absorption spectra of anisole in ethyl acetate.*
Data source location	*Data might be obtained in electronic form from the authors*
Data accessibility	*Data are contained in this article*

**Value of the data**

• Temperature dependent absorption and emission data can be used for alternative evaluation schemes for dipole moments etc.
• The temperature dependent density data can be used for own determinations of cavity sizes.
• The *ab initio* structures give the optimized structures at CC2/cc-pVTZ level of theory and can be used as good starting geometries for other levels of theory.

## 1 Data

• Fig. S1: Plot of the inverse density of the solution of anisole in ethyl acetate versus the weight fraction of anisole at 293 K along with the linear fit of the data.
• Fig. S2: Rotationally resolved electronic Stark spectrum of the electronic origin of d3-anisole at 36,387.31 cm^-1^. The field free spectrum had been obtained before by Pasquini et al. [2].
• Table S1: Measured density of the solution of anisole in ethyl acetate versus the weight fraction between 0 and 0.013 of the solute between 258 K and 348 K. The densities are given as kg/m^3^.
• Table S2: Absorption spectra (in wavenumber) of anisole in ethyl acetate between 258 K and 348 K in steps of 5 K. These data are available as Table S2.doc and Table S2.csv. The raw data (in nm) are available as Table 2.dat
• Table S3: Emission spectra (in wavenumber) of anisole in ethyl acetate between 258 K and 348 K in steps of 5 K. These data are available as Table S3.doc and Table S3.csv. The raw data (in nm) are available as Table 3.dat
• Table S4: Linearized laser induced fluorescence Stark spectrum of anisole at an electric field strength of 819.09 V/cm. The first column gives the relative frequency in 1 MHz increment, the second column the relative intensities.
• Table S5: Cartesian coordinates of anisole *S*_0_ in Bohr units from the CC2/cc-pVTZ calculations using the Turbomole program package [3].
• Table S6: Cartesian coordinates of anisole *S*_1_ in Bohr units from the CC2/cc-pVTZ calculations using the Turbomole program package [3].

## 2 Experimental design, materials and methods

The density ρ of the solution of anisole in ethyl acetate was measured at different mass fractions *w* in a temperature range of 263 K and 343 K with an increment of 2 K using a density meter (Anton Paar DMA4500M). The cavity volume is determined from the slope of the graph of ρ^-1^ vs. the mole fraction *w* at each temperature [1]. A plot of the inverse density of the solution of anisole in ethyl acetate versus the weight fraction of anisole along with the linear fit of the data is shown in Fig. S1.

Absorption spectra of anisole, dissolved in ethyl acetate have been recorded in a temperature range of − 10 °C and + 70 °C with 5 °C increment using a Varian Cary 50 Scan UV spectrometer [1]. A custom built coolable and heatable cell holder has been constructed using a pair of Peltier elements from Uwe Electronics GmbH. The hot side was cooled using a Julabo Corio 600F cooler with Thermal G, as cooling liquid. The cell holder is mounted in a vacuum chamber to avoid condensation of water on the windows at low temperatures. Fluorescence emission spectra were recorded in a Varian Cary Eclipse spectrometer using the same cell holder as for the absorption measurements.
