# Supplementary material for: Additional data for evaluation of the excited state dipole moments of anisole
Source: Data Brief. 2018 Oct 3;21:313–5. doi: 10.1016/j.dib.2018.09.110 (PMC6197573; doi:10.1016/j.dib.2018.09.110)
Supplement: Supplementary file 2 — Supplementary material [file mmc2.docx]

*Figure S1: Plot of the inverse density of the solution of anisole in ethyl acetate versus the weight fraction of anisole at 293 K along with the linear fit of the data.*

*
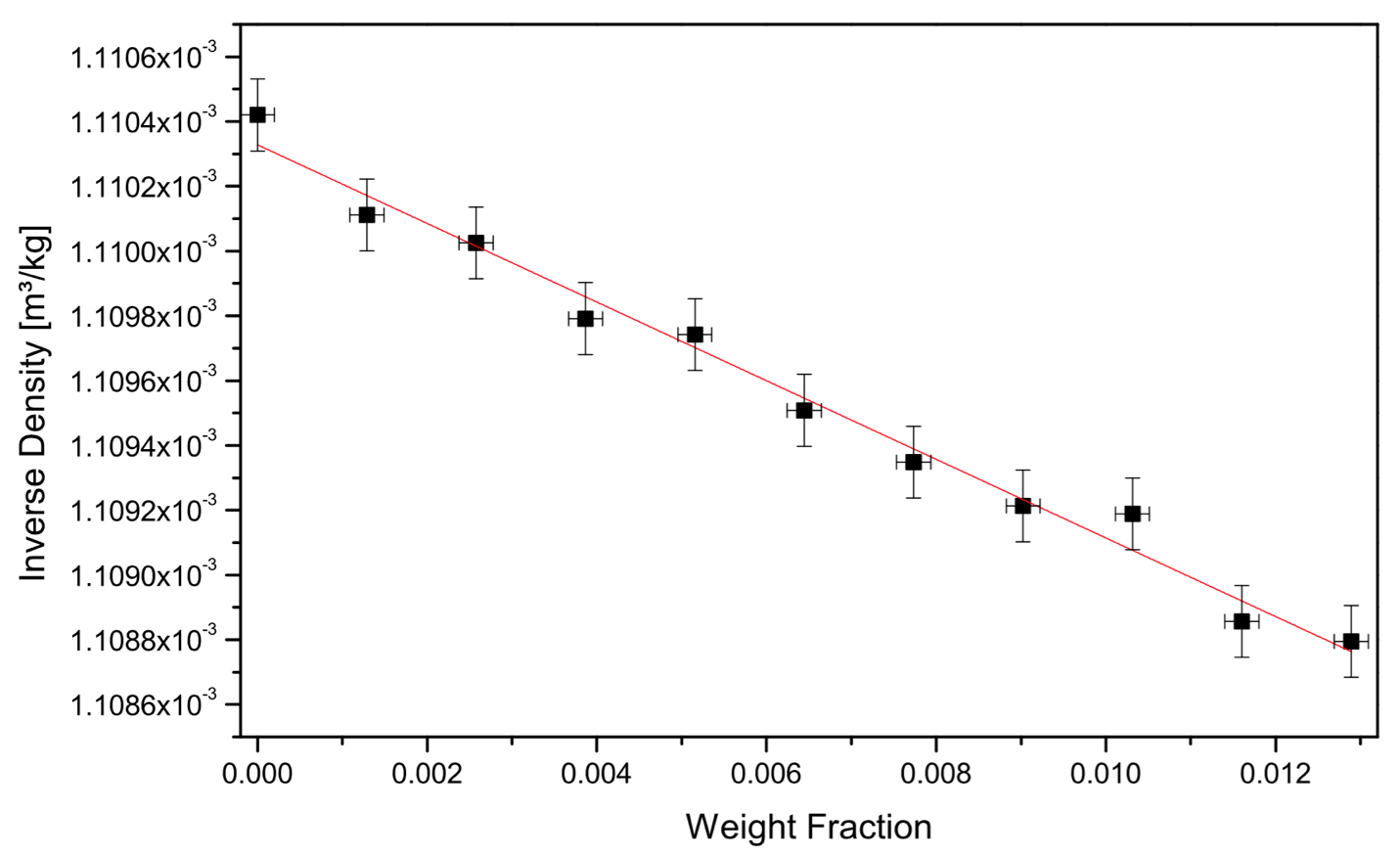
*
