# Supplementary material for: Additional data for evaluation of the excited state dipole moments of anisole
Source: Data Brief. 2018 Oct 3;21:313–5. doi: 10.1016/j.dib.2018.09.110 (PMC6197573; doi:10.1016/j.dib.2018.09.110)
Supplement: Supplementary file 3 — Supplementary material [file mmc3.docx]

*
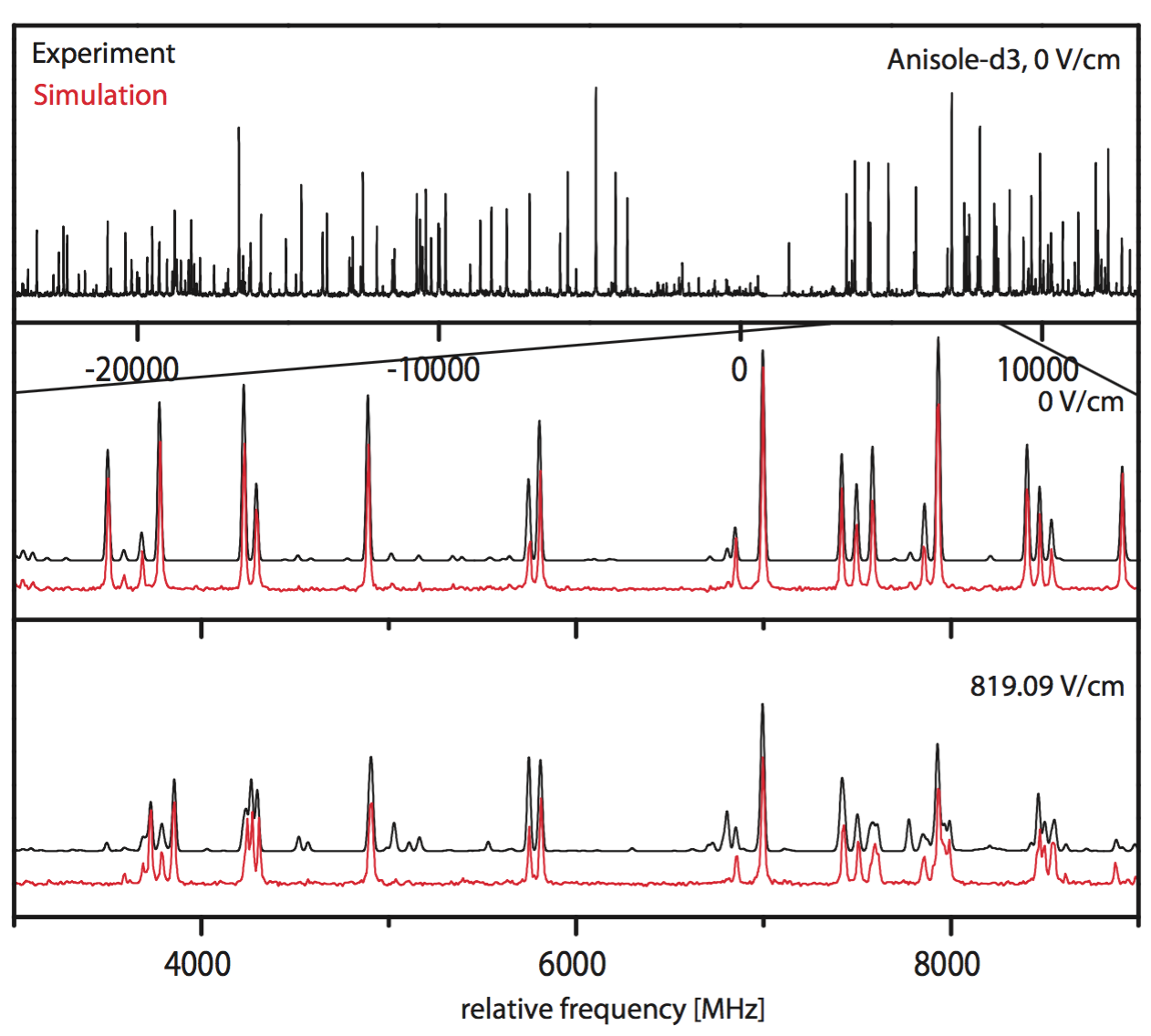
Figure S2: Rotationally resolved electronic Stark spectrum of the electronic origin of d3-anisole at 36387.31 cm^−1^.* The field free spectrum had been obtained before by Pasquini et al. [1] *.*
