# Supplementary material for: Additional data for evaluation of the excited state dipole moments of anisole
Source: Data Brief. 2018 Oct 3;21:313–5. doi: 10.1016/j.dib.2018.09.110 (PMC6197573; doi:10.1016/j.dib.2018.09.110)
Supplement: Supplementary file 4 — Supplementary material [file mmc4.docx]

*Table S1: Measured density of the solution of anisole in ethyl acetate versus the weight fraction w between 0 and 0,0129 of the solute between 258 K and 348 K. The densities are given as kg/m^3^.*

| w | 0,0000 | 0,0013 | 0,0026 | 0,0039 | 0,0052 | 0,0064 | 0,0077 | 0,0090 | 0,0103 | 0,0116 | 0,0129 |
| --- | --- | --- | --- | --- | --- | --- | --- | --- | --- | --- | --- |
| 263,15 K | 936,61 | 936,73 | 936,79 | 936,85 | 937,00 | 936,75 | 937,31 | 937,40 | 937,43 | 937,65 | 937,72 |
| 265,15 K | 934,29 | 934,38 | 934,44 | 934,64 | 934,65 | 934,85 | 934,97 | 935,07 | 935,06 | 935,34 | 935,36 |
| 267,15 K | 931,91 | 932,04 | 932,09 | 932,27 | 932,30 | 932,48 | 932,60 | 932,70 | 932,71 | 932,97 | 933,01 |
| 269,15 K | 929,54 | 929,65 | 929,72 | 929,89 | 929,92 | 930,10 | 930,22 | 930,33 | 930,34 | 930,60 | 930,63 |
| 271,15 K | 927,16 | 927,28 | 927,34 | 927,51 | 927,54 | 927,72 | 927,84 | 927,96 | 927,98 | 928,22 | 928,27 |
| 273,15 K | 924,77 | 924,89 | 924,96 | 925,13 | 925,17 | 925,34 | 925,46 | 925,58 | 925,60 | 925,86 | 925,89 |
| 275,15 K | 922,38 | 922,51 | 922,57 | 922,75 | 922,78 | 922,96 | 923,09 | 923,20 | 923,21 | 923,47 | 923,51 |
| 277,15 K | 919,99 | 920,12 | 920,17 | 920,36 | 920,39 | 920,57 | 920,70 | 920,81 | 920,83 | 921,09 | 921,13 |
| 279,15 K | 917,59 | 917,72 | 917,78 | 917,96 | 918,00 | 918,18 | 918,31 | 918,42 | 918,44 | 918,70 | 918,74 |
| 281,15 K | 915,19 | 915,32 | 915,38 | 915,57 | 915,60 | 915,79 | 915,91 | 916,02 | 916,04 | 916,30 | 916,34 |
| 283,15 K | 912,79 | 912,92 | 912,97 | 913,16 | 913,20 | 913,38 | 913,51 | 913,62 | 913,64 | 913,91 | 913,94 |
| 285,15 K | 910,38 | 910,51 | 910,57 | 910,75 | 910,79 | 910,98 | 911,11 | 911,22 | 911,24 | 911,50 | 911,54 |
| 287,15 K | 907,96 | 908,09 | 908,15 | 908,34 | 908,38 | 908,57 | 908,69 | 908,80 | 908,83 | 909,09 | 909,13 |
| 289,15 K | 905,54 | 905,67 | 905,74 | 905,92 | 905,96 | 906,15 | 906,28 | 906,39 | 906,41 | 906,68 | 906,72 |
| 291,15 K | 903,12 | 903,24 | 903,31 | 903,50 | 903,54 | 903,73 | 903,86 | 903,97 | 903,99 | 904,26 | 904,30 |
| 293,15 K | 900,68 | 900,81 | 900,88 | 901,07 | 901,11 | 901,30 | 901,43 | 901,54 | 901,56 | 901,83 | 901,88 |
| 295,15 K | 898,25 | 898,37 | 898,44 | 898,63 | 898,67 | 898,86 | 899,00 | 899,11 | 899,13 | 899,40 | 899,45 |
| 297,15 K | 895,80 | 895,93 | 896,00 | 896,19 | 896,23 | 896,42 | 896,56 | 896,67 | 896,69 | 896,97 | 897,01 |
| 299,15 K | 893,35 | 893,48 | 893,55 | 893,74 | 893,79 | 893,98 | 894,11 | 894,22 | 894,25 | 894,52 | 894,57 |
| 301,15 K | 890,89 | 891,02 | 891,10 | 891,29 | 891,33 | 891,53 | 891,66 | 891,77 | 891,80 | 892,07 | 892,12 |
| 303,15 K | 888,43 | 888,56 | 888,63 | 888,83 | 888,87 | 889,07 | 889,20 | 889,32 | 889,34 | 889,62 | 889,67 |
| 305,15 K | 885,96 | 886,09 | 886,16 | 886,36 | 886,40 | 886,60 | 886,73 | 886,85 | 886,88 | 887,16 | 887,21 |
| 307,15 K | 883,48 | 883,61 | 883,69 | 883,88 | 883,93 | 884,13 | 884,26 | 884,38 | 884,41 | 884,69 | 884,74 |
| 309,15 K | 880,99 | 881,13 | 881,21 | 881,40 | 881,45 | 881,65 | 881,78 | 881,90 | 881,93 | 882,21 | 882,26 |
| 311,15 K | 878,50 | 878,64 | 878,71 | 878,92 | 878,96 | 879,16 | 879,30 | 879,42 | 879,44 | 879,73 | 879,78 |
| 313,15 K | 876,00 | 876,14 | 876,22 | 876,42 | 876,47 | 876,67 | 876,81 | 876,93 | 876,95 | 877,24 | 877,29 |
| 315,15 K | 873,50 | 873,64 | 873,72 | 873,92 | 873,96 | 874,17 | 874,31 | 874,43 | 874,46 | 874,75 | 874,80 |
| 317,15 K | 870,98 | 871,13 | 871,20 | 871,41 | 871,45 | 871,66 | 871,80 | 871,93 | 871,95 | 872,24 | 872,30 |
| 319,15 K | 868,46 | 868,61 | 868,69 | 868,90 | 868,94 | 869,15 | 869,29 | 869,41 | 869,44 | 869,73 | 869,79 |
| 321,15 K | 865,93 | 866,08 | 866,16 | 866,37 | 866,41 | 866,62 | 866,76 | 866,89 | 866,92 | 867,21 | 867,26 |
| 323,15 K | 863,39 | 863,54 | 863,63 | 863,83 | 863,88 | 864,09 | 864,23 | 864,35 | 864,39 | 864,67 | 864,73 |
| 3251,5 K | 860,84 | 861,00 | 861,08 | 861,29 | 861,34 | 861,54 | 861,69 | 861,81 | 861,85 | 862,13 | 862,19 |
| 327,15 K | 858,29 | 858,44 | 858,53 | 858,73 | 858,78 | 858,99 | 859,13 | 859,26 | 859,30 | 859,58 | 859,65 |
| 329,15 K | 855,73 | 855,88 | 855,96 | 856,16 | 856,22 | 856,43 | 856,57 | 856,70 | 856,74 | 857,02 | 857,09 |
| 331,15 K | 853,15 | 853,30 | 853,39 | 853,59 | 853,65 | 853,85 | 854,00 | 854,13 | 854,17 | 854,45 | 854,52 |
| 333,15 K | 850,57 | 850,71 | 850,80 | 851,00 | 851,07 | 851,27 | 851,42 | 851,54 | 851,59 | 851,87 | 851,94 |
| 335,15 K | 847,97 | 848,12 | 848,21 | 848,41 | 848,47 | 848,68 | 848,83 | 848,96 | 849,01 | 849,29 | 849,36 |
| 337,15 K | 845,36 | 845,51 | 845,61 | 845,80 | 845,87 | 846,08 | 846,23 | 846,36 | 846,41 | 846,69 | 846,76 |
| 339,15 K | 842,75 | 842,90 | 842,99 | 843,19 | 843,25 | 843,46 | 843,61 | 843,75 | 843,80 | 844,08 | 844,15 |
| 341,15 K | 840,12 | 840,27 | 840,37 | 840,56 | 840,63 | 840,84 | 840,99 | 841,12 | 841,19 | 841,46 | 841,54 |
| 343,15 K | 837,51 | 837,66 | 837,76 | 837,96 | 838,03 | 838,24 | 838,39 | 838,52 | 838,58 | 838,86 | 838,93 |
