# Supplementary material for: Additional data for evaluation of the excited state dipole moments of anisole
Source: Data Brief. 2018 Oct 3;21:313–5. doi: 10.1016/j.dib.2018.09.110 (PMC6197573; doi:10.1016/j.dib.2018.09.110)
Supplement: Supplementary file 5 — Supplementary material [file mmc5.docx]

*Table S2: Absorption spectra of anisole in ethyl acetate between 258 K and 348 K in steps of 5 K.*

|  | *258 K* | *263 K* | *268 K* | *273 K* | *278 K* | *283 K* | *288 K* | *293 K* | *298 K* | *303 K* | *308 K* | *313 K* | *318 K* | *323 K* | *228 K* | *233 K* | *238 K* | *243 K* | *348 K* |
| --- | --- | --- | --- | --- | --- | --- | --- | --- | --- | --- | --- | --- | --- | --- | --- | --- | --- | --- | --- |
| *38000.12003* | *0.62933* | *0.63127* | *0.6333* | *0.63495* | *0.6377* | *0.64187* | *0.64562* | *0.6522* | *0.65725* | *0.66354* | *0.67269* | *0.68255* | *0.69147* | *0.69912* | *0.70638* | *0.71773* | *0.72355* | *0.73337* | *0.74212* |
| *37995.04758* | *0.6318* | *0.6337* | *0.63568* | *0.63729* | *0.64002* | *0.64413* | *0.64783* | *0.65441* | *0.6594* | *0.66562* | *0.67466* | *0.68442* | *0.69322* | *0.7008* | *0.7081* | *0.71939* | *0.72517* | *0.73492* | *0.74356* |
| *37989.97649* | *0.63422* | *0.6361* | *0.63802* | *0.6396* | *0.64232* | *0.64637* | *0.65002* | *0.6566* | *0.66154* | *0.66769* | *0.67661* | *0.68626* | *0.69494* | *0.70247* | *0.7098* | *0.72104* | *0.72679* | *0.73648* | *0.745* |
| *37984.90675* | *0.63661* | *0.63846* | *0.64033* | *0.64189* | *0.6446* | *0.64859* | *0.65219* | *0.65877* | *0.66366* | *0.66974* | *0.67855* | *0.6881* | *0.69666* | *0.70412* | *0.71148* | *0.72268* | *0.72839* | *0.73803* | *0.74644* |
| *37979.83837* | *0.63896* | *0.64079* | *0.64262* | *0.64414* | *0.64685* | *0.65078* | *0.65434* | *0.66093* | *0.66576* | *0.67178* | *0.68046* | *0.68991* | *0.69835* | *0.70576* | *0.71316* | *0.7243* | *0.72999* | *0.73958* | *0.74787* |
| *37974.77133* | *0.64127* | *0.64308* | *0.64486* | *0.64637* | *0.64907* | *0.65295* | *0.65646* | *0.66306* | *0.66785* | *0.67379* | *0.68236* | *0.6917* | *0.70003* | *0.70738* | *0.71481* | *0.72592* | *0.73157* | *0.74113* | *0.74929* |
| *37969.70565* | *0.64354* | *0.64533* | *0.64708* | *0.64856* | *0.65126* | *0.65509* | *0.65857* | *0.66518* | *0.66991* | *0.67578* | *0.68424* | *0.69348* | *0.70169* | *0.70898* | *0.71645* | *0.72752* | *0.73314* | *0.74268* | *0.75071* |
| *37964.64132* | *0.64577* | *0.64755* | *0.64926* | *0.65073* | *0.65342* | *0.65721* | *0.66065* | *0.66728* | *0.67196* | *0.67775* | *0.68609* | *0.69523* | *0.70333* | *0.71056* | *0.71807* | *0.7291* | *0.73471* | *0.74421* | *0.75212* |
| *37959.57834* | *0.64795* | *0.64973* | *0.6514* | *0.65286* | *0.65556* | *0.6593* | *0.66271* | *0.66935* | *0.67398* | *0.6797* | *0.68793* | *0.69697* | *0.70494* | *0.71213* | *0.71968* | *0.73066* | *0.73625* | *0.74574* | *0.75352* |
| *37954.51672* | *0.6501* | *0.65186* | *0.65351* | *0.65496* | *0.65766* | *0.66137* | *0.66474* | *0.6714* | *0.67598* | *0.68163* | *0.68974* | *0.69868* | *0.70654* | *0.71367* | *0.72126* | *0.73221* | *0.73778* | *0.74727* | *0.7549* |
| *37949.45644* | *0.6522* | *0.65396* | *0.65558* | *0.65702* | *0.65974* | *0.6634* | *0.66674* | *0.67343* | *0.67795* | *0.68353* | *0.69152* | *0.70036* | *0.70812* | *0.7152* | *0.72283* | *0.73374* | *0.7393* | *0.74878* | *0.75628* |
| *37944.39751* | *0.65426* | *0.65602* | *0.65762* | *0.65905* | *0.66178* | *0.66541* | *0.66872* | *0.67543* | *0.6799* | *0.68541* | *0.69328* | *0.70203* | *0.70968* | *0.7167* | *0.72437* | *0.73526* | *0.7408* | *0.75028* | *0.75764* |
| *37939.33993* | *0.65628* | *0.65804* | *0.65962* | *0.66105* | *0.66379* | *0.66738* | *0.67067* | *0.6774* | *0.68182* | *0.68726* | *0.69502* | *0.70367* | *0.71121* | *0.71819* | *0.72589* | *0.73675* | *0.74228* | *0.75177* | *0.75898* |
| *37934.28369* | *0.65825* | *0.66001* | *0.66157* | *0.66301* | *0.66577* | *0.66933* | *0.67259* | *0.67935* | *0.68372* | *0.68909* | *0.69673* | *0.70529* | *0.71272* | *0.71965* | *0.72739* | *0.73822* | *0.74374* | *0.75324* | *0.76031* |
| *37929.22881* | *0.66018* | *0.66194* | *0.66349* | *0.66493* | *0.66771* | *0.67124* | *0.67448* | *0.68127* | *0.68559* | *0.69089* | *0.69841* | *0.70688* | *0.7142* | *0.72108* | *0.72886* | *0.73967* | *0.74518* | *0.7547* | *0.76163* |
| *37924.17527* | *0.66207* | *0.66383* | *0.66537* | *0.66682* | *0.66961* | *0.67312* | *0.67634* | *0.68316* | *0.68743* | *0.69266* | *0.70007* | *0.70844* | *0.71566* | *0.7225* | *0.73031* | *0.74109* | *0.7466* | *0.75614* | *0.76292* |
| *37919.12307* | *0.6639* | *0.66568* | *0.66721* | *0.66867* | *0.67148* | *0.67497* | *0.67817* | *0.68502* | *0.68924* | *0.6944* | *0.7017* | *0.70998* | *0.7171* | *0.72389* | *0.73174* | *0.74249* | *0.748* | *0.75757* | *0.7642* |
| *37914.07223* | *0.6657* | *0.66748* | *0.66901* | *0.67048* | *0.67332* | *0.67678* | *0.67997* | *0.68685* | *0.69102* | *0.69611* | *0.70329* | *0.71149* | *0.71851* | *0.72525* | *0.73314* | *0.74387* | *0.74937* | *0.75897* | *0.76546* |
| *37909.02273* | *0.66744* | *0.66924* | *0.67077* | *0.67226* | *0.67512* | *0.67856* | *0.68174* | *0.68865* | *0.69277* | *0.69779* | *0.70486* | *0.71297* | *0.71989* | *0.72659* | *0.73451* | *0.74522* | *0.75072* | *0.76036* | *0.7667* |
| *37903.97457* | *0.66914* | *0.67095* | *0.67248* | *0.67399* | *0.67688* | *0.6803* | *0.68347* | *0.69041* | *0.69449* | *0.69943* | *0.7064* | *0.71442* | *0.72125* | *0.72791* | *0.73585* | *0.74655* | *0.75205* | *0.76173* | *0.76791* |
| *37898.92776* | *0.6708* | *0.67262* | *0.67416* | *0.67568* | *0.6786* | *0.68201* | *0.68517* | *0.69215* | *0.69617* | *0.70105* | *0.70791* | *0.71585* | *0.72258* | *0.72919* | *0.73717* | *0.74784* | *0.75335* | *0.76307* | *0.7691* |
| *37893.88229* | *0.6724* | *0.67424* | *0.67579* | *0.67734* | *0.68028* | *0.68368* | *0.68683* | *0.69384* | *0.69782* | *0.70263* | *0.70938* | *0.71724* | *0.72388* | *0.73046* | *0.73846* | *0.74912* | *0.75462* | *0.76438* | *0.77027* |
| *37888.83817* | *0.67396* | *0.67582* | *0.67738* | *0.67895* | *0.68193* | *0.68531* | *0.68846* | *0.6955* | *0.69944* | *0.70418* | *0.71083* | *0.7186* | *0.72516* | *0.73169* | *0.73972* | *0.75036* | *0.75586* | *0.76568* | *0.77142* |
| *37883.79538* | *0.67547* | *0.67735* | *0.67892* | *0.68053* | *0.68353* | *0.6869* | *0.69005* | *0.69713* | *0.70102* | *0.70569* | *0.71224* | *0.71993* | *0.7264* | *0.7329* | *0.74094* | *0.75157* | *0.75708* | *0.76695* | *0.77253* |
| *37878.75394* | *0.67694* | *0.67884* | *0.68043* | *0.68206* | *0.6851* | *0.68846* | *0.69161* | *0.69872* | *0.70256* | *0.70717* | *0.71361* | *0.72123* | *0.72762* | *0.73407* | *0.74214* | *0.75275* | *0.75827* | *0.76818* | *0.77363* |
| *37873.71384* | *0.67835* | *0.68028* | *0.68189* | *0.68355* | *0.68662* | *0.68998* | *0.69313* | *0.70027* | *0.70407* | *0.70861* | *0.71496* | *0.7225* | *0.7288* | *0.73522* | *0.74331* | *0.7539* | *0.75942* | *0.7694* | *0.77469* |
| *37868.67509* | *0.67972* | *0.68167* | *0.6833* | *0.685* | *0.6881* | *0.69146* | *0.69461* | *0.70178* | *0.70554* | *0.71002* | *0.71627* | *0.72374* | *0.72996* | *0.73634* | *0.74445* | *0.75503* | *0.76055* | *0.77058* | *0.77573* |
| *37863.63767* | *0.68104* | *0.68302* | *0.68467* | *0.68641* | *0.68955* | *0.69289* | *0.69606* | *0.70325* | *0.70698* | *0.71139* | *0.71754* | *0.72494* | *0.73109* | *0.73743* | *0.74555* | *0.75611* | *0.76165* | *0.77173* | *0.77674* |
| *37858.60159* | *0.68232* | *0.68432* | *0.686* | *0.68777* | *0.69095* | *0.69429* | *0.69746* | *0.70469* | *0.70837* | *0.71272* | *0.71878* | *0.72611* | *0.73218* | *0.73849* | *0.74662* | *0.75717* | *0.76271* | *0.77285* | *0.77771* |
| *37853.56686* | *0.68355* | *0.68558* | *0.68729* | *0.6891* | *0.69231* | *0.69565* | *0.69883* | *0.70608* | *0.70973* | *0.71402* | *0.71999* | *0.72725* | *0.73325* | *0.73952* | *0.74766* | *0.7582* | *0.76374* | *0.77394* | *0.77866* |
| *37848.53346* | *0.68472* | *0.68679* | *0.68853* | *0.69038* | *0.69362* | *0.69697* | *0.70016* | *0.70743* | *0.71104* | *0.71528* | *0.72115* | *0.72835* | *0.73428* | *0.74052* | *0.74866* | *0.75919* | *0.76473* | *0.775* | *0.77958* |
| *37843.5014* | *0.68586* | *0.68795* | *0.68972* | *0.69161* | *0.6949* | *0.69825* | *0.70145* | *0.70874* | *0.71232* | *0.7165* | *0.72229* | *0.72942* | *0.73528* | *0.74149* | *0.74963* | *0.76014* | *0.7657* | *0.77602* | *0.78046* |
| *37838.47067* | *0.68694* | *0.68907* | *0.69088* | *0.69281* | *0.69613* | *0.69948* | *0.7027* | *0.71001* | *0.71355* | *0.71767* | *0.72338* | *0.73045* | *0.73625* | *0.74243* | *0.75057* | *0.76107* | *0.76662* | *0.77701* | *0.78132* |
| *37833.44129* | *0.68798* | *0.69014* | *0.69198* | *0.69396* | *0.69732* | *0.70068* | *0.7039* | *0.71124* | *0.71475* | *0.71881* | *0.72444* | *0.73145* | *0.73719* | *0.74334* | *0.75147* | *0.76195* | *0.76752* | *0.77796* | *0.78214* |
| *37828.41324* | *0.68897* | *0.69116* | *0.69305* | *0.69507* | *0.69846* | *0.70183* | *0.70507* | *0.71242* | *0.7159* | *0.71991* | *0.72547* | *0.73242* | *0.7381* | *0.74422* | *0.75234* | *0.76281* | *0.76838* | *0.77888* | *0.78292* |
| *37823.38653* | *0.68991* | *0.69214* | *0.69407* | *0.69614* | *0.69957* | *0.70294* | *0.7062* | *0.71356* | *0.71701* | *0.72097* | *0.72645* | *0.73335* | *0.73898* | *0.74506* | *0.75317* | *0.76363* | *0.7692* | *0.77976* | *0.78368* |
| *37818.36115* | *0.69081* | *0.69308* | *0.69504* | *0.69716* | *0.70063* | *0.70401* | *0.70729* | *0.71466* | *0.71808* | *0.722* | *0.7274* | *0.73424* | *0.73982* | *0.74588* | *0.75397* | *0.76441* | *0.76998* | *0.7806* | *0.7844* |
| *37813.33711* | *0.69166* | *0.69396* | *0.69598* | *0.69814* | *0.70164* | *0.70504* | *0.70833* | *0.71572* | *0.71911* | *0.72298* | *0.72831* | *0.7351* | *0.74063* | *0.74666* | *0.75474* | *0.76516* | *0.77073* | *0.78141* | *0.78508* |
| *37808.31441* | *0.69247* | *0.69481* | *0.69687* | *0.69908* | *0.70262* | *0.70602* | *0.70934* | *0.71673* | *0.72009* | *0.72392* | *0.72919* | *0.73593* | *0.74141* | *0.74741* | *0.75547* | *0.76587* | *0.77145* | *0.78218* | *0.78573* |
| *37803.29303* | *0.69323* | *0.69561* | *0.69771* | *0.69998* | *0.70355* | *0.70696* | *0.7103* | *0.7177* | *0.72104* | *0.72481* | *0.73002* | *0.73672* | *0.74216* | *0.74813* | *0.75616* | *0.76655* | *0.77213* | *0.78291* | *0.78634* |
| *37798.273* | *0.69395* | *0.69636* | *0.69852* | *0.70084* | *0.70444* | *0.70786* | *0.71122* | *0.71862* | *0.72194* | *0.72567* | *0.73082* | *0.73747* | *0.74287* | *0.74882* | *0.75682* | *0.76719* | *0.77277* | *0.7836* | *0.78692* |
| *37793.25429* | *0.69462* | *0.69707* | *0.69928* | *0.70165* | *0.70528* | *0.70872* | *0.7121* | *0.7195* | *0.7228* | *0.72649* | *0.73159* | *0.73819* | *0.74355* | *0.74947* | *0.75744* | *0.76779* | *0.77337* | *0.78425* | *0.78747* |
| *37788.23692* | *0.69525* | *0.69774* | *0.7* | *0.70242* | *0.70609* | *0.70954* | *0.71294* | *0.72034* | *0.72361* | *0.72727* | *0.73231* | *0.73888* | *0.7442* | *0.7501* | *0.75803* | *0.76836* | *0.77393* | *0.78486* | *0.78797* |
| *37783.22088* | *0.69583* | *0.69837* | *0.70068* | *0.70315* | *0.70685* | *0.71032* | *0.71374* | *0.72113* | *0.72439* | *0.72801* | *0.733* | *0.73953* | *0.74482* | *0.75069* | *0.75858* | *0.76889* | *0.77446* | *0.78543* | *0.78845* |
| *37778.20617* | *0.69637* | *0.69895* | *0.70132* | *0.70384* | *0.70757* | *0.71105* | *0.7145* | *0.72188* | *0.72512* | *0.72871* | *0.73365* | *0.74015* | *0.74541* | *0.75125* | *0.7591* | *0.76939* | *0.77496* | *0.78597* | *0.78888* |
| *37773.19279* | *0.69687* | *0.69949* | *0.70191* | *0.70449* | *0.70825* | *0.71175* | *0.71521* | *0.72258* | *0.72581* | *0.72936* | *0.73427* | *0.74073* | *0.74597* | *0.75178* | *0.75959* | *0.76985* | *0.77541* | *0.78646* | *0.78928* |
| *37768.18074* | *0.69733* | *0.69999* | *0.70247* | *0.7051* | *0.70889* | *0.7124* | *0.71589* | *0.72324* | *0.72646* | *0.72998* | *0.73485* | *0.74128* | *0.74649* | *0.75229* | *0.76004* | *0.77028* | *0.77583* | *0.78691* | *0.78965* |
| *37763.17002* | *0.69775* | *0.70045* | *0.70299* | *0.70567* | *0.70949* | *0.71301* | *0.71653* | *0.72386* | *0.72707* | *0.73056* | *0.73539* | *0.74179* | *0.74699* | *0.75276* | *0.76046* | *0.77067* | *0.77621* | *0.78733* | *0.78998* |
| *37758.16063* | *0.69813* | *0.70087* | *0.70346* | *0.7062* | *0.71004* | *0.71358* | *0.71713* | *0.72444* | *0.72763* | *0.7311* | *0.7359* | *0.74227* | *0.74745* | *0.7532* | *0.76084* | *0.77103* | *0.77656* | *0.7877* | *0.79027* |
| *37753.15257* | *0.69847* | *0.70126* | *0.7039* | *0.7067* | *0.71056* | *0.71412* | *0.71768* | *0.72497* | *0.72816* | *0.7316* | *0.73638* | *0.74272* | *0.74789* | *0.75361* | *0.76119* | *0.77135* | *0.77687* | *0.78803* | *0.79053* |
| *37748.14584* | *0.69877* | *0.7016* | *0.7043* | *0.70715* | *0.71104* | *0.71461* | *0.7182* | *0.72546* | *0.72864* | *0.73207* | *0.73682* | *0.74314* | *0.74829* | *0.75399* | *0.76151* | *0.77164* | *0.77715* | *0.78833* | *0.79075* |
| *37743.14044* | *0.69903* | *0.70191* | *0.70467* | *0.70757* | *0.71148* | *0.71507* | *0.71868* | *0.72592* | *0.72909* | *0.73249* | *0.73722* | *0.74352* | *0.74867* | *0.75434* | *0.76179* | *0.77189* | *0.77739* | *0.78859* | *0.79094* |
| *37738.13636* | *0.69926* | *0.70218* | *0.705* | *0.70795* | *0.71188* | *0.71548* | *0.71912* | *0.72633* | *0.7295* | *0.73288* | *0.73759* | *0.74387* | *0.74901* | *0.75467* | *0.76205* | *0.77212* | *0.77759* | *0.78881* | *0.79109* |
| *37733.13361* | *0.69945* | *0.70241* | *0.70529* | *0.7083* | *0.71225* | *0.71587* | *0.71953* | *0.7267* | *0.72986* | *0.73324* | *0.73793* | *0.7442* | *0.74933* | *0.75496* | *0.76227* | *0.77231* | *0.77776* | *0.78899* | *0.79121* |
| *37728.13219* | *0.69961* | *0.70261* | *0.70555* | *0.70861* | *0.71258* | *0.71621* | *0.7199* | *0.72703* | *0.7302* | *0.73355* | *0.73824* | *0.74449* | *0.74962* | *0.75523* | *0.76246* | *0.77247* | *0.7779* | *0.78913* | *0.7913* |
| *37723.13209* | *0.69973* | *0.70277* | *0.70578* | *0.70888* | *0.71287* | *0.71652* | *0.72023* | *0.72732* | *0.73049* | *0.73383* | *0.73852* | *0.74475* | *0.74989* | *0.75548* | *0.76263* | *0.77259* | *0.77801* | *0.78924* | *0.79135* |
| *37718.13332* | *0.69982* | *0.70291* | *0.70597* | *0.70913* | *0.71313* | *0.71679* | *0.72053* | *0.72757* | *0.73075* | *0.73408* | *0.73876* | *0.74498* | *0.75012* | *0.75569* | *0.76276* | *0.77269* | *0.77808* | *0.78931* | *0.79137* |
| *37713.13587* | *0.69988* | *0.70301* | *0.70613* | *0.70934* | *0.71336* | *0.71703* | *0.72079* | *0.72779* | *0.73097* | *0.7343* | *0.73898* | *0.74519* | *0.75034* | *0.75588* | *0.76287* | *0.77276* | *0.77812* | *0.78934* | *0.79137* |
| *37708.13975* | *0.69991* | *0.70308* | *0.70626* | *0.70952* | *0.71355* | *0.71724* | *0.72102* | *0.72797* | *0.73115* | *0.73448* | *0.73916* | *0.74537* | *0.75052* | *0.75605* | *0.76295* | *0.7728* | *0.77813* | *0.78934* | *0.79132* |
| *37703.14495* | *0.69991* | *0.70312* | *0.70636* | *0.70966* | *0.71371* | *0.71741* | *0.72121* | *0.72812* | *0.73131* | *0.73463* | *0.73932* | *0.74552* | *0.75069* | *0.75619* | *0.763* | *0.77281* | *0.77812* | *0.78931* | *0.79125* |
| *37698.15147* | *0.69988* | *0.70313* | *0.70643* | *0.70978* | *0.71384* | *0.71755* | *0.72138* | *0.72824* | *0.73143* | *0.73475* | *0.73945* | *0.74565* | *0.75083* | *0.75631* | *0.76303* | *0.77279* | *0.77807* | *0.78924* | *0.79116* |
| *37693.15931* | *0.69982* | *0.70312* | *0.70647* | *0.70987* | *0.71394* | *0.71767* | *0.72151* | *0.72832* | *0.73152* | *0.73484* | *0.73955* | *0.74575* | *0.75094* | *0.75641* | *0.76303* | *0.77275* | *0.778* | *0.78914* | *0.79103* |
| *37688.16848* | *0.69974* | *0.70308* | *0.70649* | *0.70994* | *0.71401* | *0.71775* | *0.72161* | *0.72836* | *0.73158* | *0.7349* | *0.73963* | *0.74583* | *0.75104* | *0.75649* | *0.76301* | *0.77268* | *0.77789* | *0.78902* | *0.79087* |
| *37683.17897* | *0.69963* | *0.70301* | *0.70648* | *0.70997* | *0.71406* | *0.7178* | *0.72169* | *0.72838* | *0.7316* | *0.73494* | *0.73968* | *0.74588* | *0.75112* | *0.75654* | *0.76297* | *0.77259* | *0.77777* | *0.78886* | *0.79069* |
| *37678.19078* | *0.69951* | *0.70292* | *0.70645* | *0.70998* | *0.71407* | *0.71783* | *0.72173* | *0.72837* | *0.73161* | *0.73494* | *0.73971* | *0.74592* | *0.75117* | *0.75658* | *0.76291* | *0.77247* | *0.77762* | *0.78867* | *0.79049* |
| *37673.20391* | *0.69935* | *0.70281* | *0.7064* | *0.70997* | *0.71406* | *0.71783* | *0.72175* | *0.72833* | *0.73158* | *0.73493* | *0.73972* | *0.74593* | *0.75121* | *0.75659* | *0.76282* | *0.77234* | *0.77744* | *0.78846* | *0.79026* |
| *37668.21836* | *0.69918* | *0.70268* | *0.70632* | *0.70994* | *0.71403* | *0.71781* | *0.72174* | *0.72827* | *0.73153* | *0.73489* | *0.73971* | *0.74592* | *0.75123* | *0.75659* | *0.76272* | *0.77218* | *0.77725* | *0.78822* | *0.79* |
| *37663.23413* | *0.69899* | *0.70253* | *0.70622* | *0.70988* | *0.71398* | *0.71776* | *0.72171* | *0.72818* | *0.73145* | *0.73482* | *0.73968* | *0.7459* | *0.75123* | *0.75657* | *0.7626* | *0.772* | *0.77703* | *0.78795* | *0.78973* |
| *37658.25122* | *0.69879* | *0.70236* | *0.70611* | *0.7098* | *0.7139* | *0.71769* | *0.72166* | *0.72806* | *0.73135* | *0.73474* | *0.73963* | *0.74586* | *0.75122* | *0.75654* | *0.76246* | *0.77181* | *0.77679* | *0.78766* | *0.78943* |
| *37653.26962* | *0.69856* | *0.70217* | *0.70597* | *0.70971* | *0.71381* | *0.7176* | *0.72158* | *0.72793* | *0.73123* | *0.73464* | *0.73956* | *0.7458* | *0.75119* | *0.75649* | *0.7623* | *0.7716* | *0.77654* | *0.78735* | *0.78912* |
| *37648.28935* | *0.69833* | *0.70197* | *0.70582* | *0.70959* | *0.71369* | *0.7175* | *0.72149* | *0.72777* | *0.73109* | *0.73452* | *0.73948* | *0.74573* | *0.75115* | *0.75643* | *0.76214* | *0.77137* | *0.77626* | *0.78702* | *0.78878* |
| *37643.31039* | *0.69808* | *0.70176* | *0.70566* | *0.70946* | *0.71356* | *0.71737* | *0.72137* | *0.72759* | *0.73094* | *0.73438* | *0.73938* | *0.74564* | *0.7511* | *0.75636* | *0.76195* | *0.77113* | *0.77598* | *0.78667* | *0.78843* |
| *37638.33275* | *0.69782* | *0.70153* | *0.70548* | *0.70932* | *0.71341* | *0.71723* | *0.72124* | *0.7274* | *0.73076* | *0.73422* | *0.73927* | *0.74554* | *0.75103* | *0.75628* | *0.76176* | *0.77087* | *0.77567* | *0.78631* | *0.78807* |
| *37633.35642* | *0.69755* | *0.7013* | *0.70529* | *0.70916* | *0.71325* | *0.71707* | *0.72109* | *0.72719* | *0.73057* | *0.73405* | *0.73914* | *0.74544* | *0.75096* | *0.75618* | *0.76156* | *0.77061* | *0.77536* | *0.78593* | *0.78769* |
| *37628.38141* | *0.69727* | *0.70105* | *0.70509* | *0.70899* | *0.71308* | *0.7169* | *0.72093* | *0.72697* | *0.73037* | *0.73387* | *0.73901* | *0.74532* | *0.75088* | *0.75608* | *0.76135* | *0.77033* | *0.77503* | *0.78553* | *0.7873* |
| *37623.40772* | *0.69698* | *0.7008* | *0.70488* | *0.70881* | *0.71289* | *0.71672* | *0.72076* | *0.72674* | *0.73015* | *0.73368* | *0.73887* | *0.74519* | *0.75079* | *0.75597* | *0.76113* | *0.77005* | *0.7747* | *0.78512* | *0.7869* |
| *37618.43534* | *0.6967* | *0.70054* | *0.70467* | *0.70862* | *0.7127* | *0.71652* | *0.72057* | *0.72649* | *0.72993* | *0.73349* | *0.73872* | *0.74506* | *0.7507* | *0.75585* | *0.76091* | *0.76976* | *0.77436* | *0.7847* | *0.78649* |
| *37613.46427* | *0.6964* | *0.70028* | *0.70445* | *0.70843* | *0.7125* | *0.71632* | *0.72038* | *0.72624* | *0.72969* | *0.73328* | *0.73856* | *0.74493* | *0.7506* | *0.75573* | *0.76068* | *0.76947* | *0.77401* | *0.78428* | *0.78607* |
| *37608.49452* | *0.69611* | *0.70002* | *0.70422* | *0.70823* | *0.71229* | *0.71611* | *0.72017* | *0.72598* | *0.72945* | *0.73307* | *0.7384* | *0.74479* | *0.7505* | *0.75561* | *0.76046* | *0.76917* | *0.77366* | *0.78384* | *0.78565* |
| *37603.52608* | *0.69582* | *0.69975* | *0.704* | *0.70802* | *0.71208* | *0.7159* | *0.71996* | *0.72571* | *0.72921* | *0.73285* | *0.73824* | *0.74465* | *0.7504* | *0.75549* | *0.76023* | *0.76887* | *0.7733* | *0.78341* | *0.78522* |
| *37598.55895* | *0.69553* | *0.69949* | *0.70377* | *0.70782* | *0.71186* | *0.71568* | *0.71975* | *0.72544* | *0.72896* | *0.73264* | *0.73808* | *0.74451* | *0.75029* | *0.75536* | *0.76* | *0.76857* | *0.77295* | *0.78297* | *0.7848* |
| *37593.59314* | *0.69524* | *0.69923* | *0.70355* | *0.70761* | *0.71165* | *0.71547* | *0.71953* | *0.72518* | *0.72871* | *0.73242* | *0.73792* | *0.74437* | *0.75019* | *0.75524* | *0.75977* | *0.76827* | *0.77259* | *0.78252* | *0.78437* |
| *37588.62864* | *0.69496* | *0.69898* | *0.70332* | *0.7074* | *0.71143* | *0.71525* | *0.71932* | *0.72491* | *0.72846* | *0.7322* | *0.73776* | *0.74423* | *0.7501* | *0.75512* | *0.75955* | *0.76797* | *0.77224* | *0.78208* | *0.78394* |
| *37583.66544* | *0.69469* | *0.69873* | *0.70311* | *0.7072* | *0.71122* | *0.71503* | *0.7191* | *0.72464* | *0.72822* | *0.73199* | *0.73761* | *0.7441* | *0.75* | *0.755* | *0.75933* | *0.76768* | *0.77189* | *0.78164* | *0.78351* |
| *37578.70356* | *0.69443* | *0.69849* | *0.7029* | *0.707* | *0.71101* | *0.71482* | *0.71889* | *0.72438* | *0.72798* | *0.73178* | *0.73746* | *0.74397* | *0.74992* | *0.75489* | *0.75912* | *0.7674* | *0.77155* | *0.78121* | *0.78309* |
| *37573.74299* | *0.69418* | *0.69827* | *0.70269* | *0.70681* | *0.71081* | *0.71462* | *0.71868* | *0.72413* | *0.72775* | *0.73159* | *0.73732* | *0.74385* | *0.74984* | *0.75479* | *0.75892* | *0.76712* | *0.77121* | *0.78079* | *0.78268* |
| *37568.78373* | *0.69395* | *0.69805* | *0.7025* | *0.70663* | *0.71062* | *0.71442* | *0.71848* | *0.72389* | *0.72752* | *0.7314* | *0.73719* | *0.74375* | *0.74977* | *0.75469* | *0.75873* | *0.76686* | *0.77089* | *0.78037* | *0.78227* |
| *37563.82578* | *0.69373* | *0.69785* | *0.70232* | *0.70646* | *0.71044* | *0.71423* | *0.71828* | *0.72366* | *0.72731* | *0.73122* | *0.73707* | *0.74365* | *0.74971* | *0.75461* | *0.75856* | *0.7666* | *0.77058* | *0.77996* | *0.78188* |
| *37558.86913* | *0.69352* | *0.69766* | *0.70216* | *0.7063* | *0.71027* | *0.71405* | *0.7181* | *0.72344* | *0.72711* | *0.73105* | *0.73696* | *0.74356* | *0.74966* | *0.75453* | *0.7584* | *0.76636* | *0.77028* | *0.77957* | *0.78149* |
| *37553.9138* | *0.69334* | *0.6975* | *0.70201* | *0.70615* | *0.71012* | *0.71389* | *0.71793* | *0.72324* | *0.72692* | *0.7309* | *0.73687* | *0.74349* | *0.74963* | *0.75447* | *0.75825* | *0.76614* | *0.76999* | *0.77919* | *0.78112* |
| *37548.95977* | *0.69318* | *0.69735* | *0.70187* | *0.70602* | *0.70998* | *0.71374* | *0.71778* | *0.72306* | *0.72676* | *0.73077* | *0.7368* | *0.74344* | *0.74961* | *0.75443* | *0.75812* | *0.76593* | *0.76973* | *0.77883* | *0.78077* |
| *37544.00704* | *0.69304* | *0.69723* | *0.70176* | *0.70591* | *0.70986* | *0.71361* | *0.71764* | *0.72289* | *0.72661* | *0.73066* | *0.73674* | *0.74341* | *0.74961* | *0.7544* | *0.75801* | *0.76575* | *0.76948* | *0.77849* | *0.78044* |
| *37539.05563* | *0.69293* | *0.69713* | *0.70167* | *0.70582* | *0.70976* | *0.7135* | *0.71752* | *0.72275* | *0.72648* | *0.73056* | *0.73671* | *0.74339* | *0.74963* | *0.75439* | *0.75793* | *0.76558* | *0.76926* | *0.77817* | *0.78012* |
| *37534.10552* | *0.69284* | *0.69705* | *0.7016* | *0.70575* | *0.70968* | *0.71341* | *0.71742* | *0.72263* | *0.72637* | *0.73049* | *0.73669* | *0.7434* | *0.74967* | *0.7544* | *0.75786* | *0.76544* | *0.76905* | *0.77787* | *0.77983* |
| *37529.15671* | *0.69278* | *0.697* | *0.70156* | *0.7057* | *0.70962* | *0.71335* | *0.71734* | *0.72254* | *0.72629* | *0.73045* | *0.7367* | *0.74343* | *0.74973* | *0.75444* | *0.75782* | *0.76532* | *0.76888* | *0.7776* | *0.77956* |
| *37524.20921* | *0.69276* | *0.69698* | *0.70154* | *0.70568* | *0.70959* | *0.71331* | *0.71729* | *0.72247* | *0.72624* | *0.73043* | *0.73674* | *0.74348* | *0.74981* | *0.75449* | *0.75781* | *0.76523* | *0.76872* | *0.77736* | *0.77932* |
| *37519.26302* | *0.69276* | *0.697* | *0.70156* | *0.70568* | *0.70959* | *0.71329* | *0.71726* | *0.72244* | *0.72622* | *0.73044* | *0.7368* | *0.74357* | *0.74992* | *0.75457* | *0.75783* | *0.76516* | *0.7686* | *0.77714* | *0.7791* |
| *37514.31813* | *0.69281* | *0.69704* | *0.7016* | *0.70571* | *0.70962* | *0.71331* | *0.71727* | *0.72244* | *0.72622* | *0.73048* | *0.73689* | *0.74368* | *0.75006* | *0.75468* | *0.75787* | *0.76513* | *0.76851* | *0.77696* | *0.77891* |
| *37509.37454* | *0.69288* | *0.69712* | *0.70168* | *0.70578* | *0.70968* | *0.71336* | *0.7173* | *0.72247* | *0.72626* | *0.73056* | *0.73702* | *0.74382* | *0.75023* | *0.75481* | *0.75795* | *0.76512* | *0.76845* | *0.77681* | *0.77876* |
| *37504.43225* | *0.693* | *0.69724* | *0.70179* | *0.70587* | *0.70977* | *0.71344* | *0.71736* | *0.72254* | *0.72634* | *0.73067* | *0.73718* | *0.74399* | *0.75042* | *0.75497* | *0.75806* | *0.76516* | *0.76842* | *0.77669* | *0.77863* |
| *37499.49127* | *0.69316* | *0.6974* | *0.70193* | *0.706* | *0.7099* | *0.71355* | *0.71746* | *0.72265* | *0.72645* | *0.73081* | *0.73737* | *0.7442* | *0.75065* | *0.75517* | *0.75821* | *0.76522* | *0.76843* | *0.77662* | *0.77855* |
| *37494.55158* | *0.69336* | *0.6976* | *0.70212* | *0.70617* | *0.71007* | *0.7137* | *0.7176* | *0.7228* | *0.72661* | *0.731* | *0.7376* | *0.74444* | *0.75091* | *0.7554* | *0.75839* | *0.76532* | *0.76848* | *0.77658* | *0.7785* |
| *37489.6132* | *0.6936* | *0.69784* | *0.70234* | *0.70638* | *0.71027* | *0.71389* | *0.71778* | *0.72299* | *0.7268* | *0.73122* | *0.73786* | *0.74472* | *0.75121* | *0.75566* | *0.75861* | *0.76547* | *0.76857* | *0.77658* | *0.77848* |
| *37484.67612* | *0.69389* | *0.69812* | *0.70261* | *0.70663* | *0.71052* | *0.71413* | *0.71799* | *0.72322* | *0.72704* | *0.73149* | *0.73817* | *0.74503* | *0.75154* | *0.75595* | *0.75887* | *0.76565* | *0.7687* | *0.77663* | *0.77851* |
| *37479.74034* | *0.69423* | *0.69845* | *0.70292* | *0.70691* | *0.71081* | *0.7144* | *0.71825* | *0.7235* | *0.72732* | *0.7318* | *0.73851* | *0.74539* | *0.75191* | *0.75629* | *0.75917* | *0.76587* | *0.76887* | *0.77672* | *0.77858* |
| *37474.80586* | *0.69462* | *0.69883* | *0.70328* | *0.70725* | *0.71115* | *0.71472* | *0.71855* | *0.72383* | *0.72764* | *0.73215* | *0.7389* | *0.74579* | *0.75232* | *0.75666* | *0.75952* | *0.76614* | *0.76908* | *0.77685* | *0.77869* |
| *37469.87268* | *0.69506* | *0.69926* | *0.70368* | *0.70762* | *0.71153* | *0.71509* | *0.7189* | *0.72421* | *0.72802* | *0.73255* | *0.73934* | *0.74623* | *0.75277* | *0.75707* | *0.7599* | *0.76645* | *0.76934* | *0.77704* | *0.77885* |
| *37464.9408* | *0.69555* | *0.69974* | *0.70413* | *0.70805* | *0.71196* | *0.7155* | *0.7193* | *0.72464* | *0.72845* | *0.733* | *0.73982* | *0.74671* | *0.75326* | *0.75753* | *0.76034* | *0.76681* | *0.76965* | *0.77727* | *0.77905* |
| *37460.01022* | *0.6961* | *0.70028* | *0.70464* | *0.70852* | *0.71244* | *0.71597* | *0.71974* | *0.72512* | *0.72892* | *0.7335* | *0.74035* | *0.74725* | *0.7538* | *0.75803* | *0.76082* | *0.76721* | *0.77001* | *0.77755* | *0.77931* |
| *37455.08093* | *0.6967* | *0.70086* | *0.70519* | *0.70905* | *0.71297* | *0.71648* | *0.72024* | *0.72566* | *0.72946* | *0.73406* | *0.74093* | *0.74783* | *0.75438* | *0.75857* | *0.76135* | *0.76767* | *0.77041* | *0.77789* | *0.77961* |
| *37450.15294* | *0.69736* | *0.70151* | *0.7058* | *0.70962* | *0.71355* | *0.71705* | *0.72079* | *0.72626* | *0.73004* | *0.73467* | *0.74156* | *0.74845* | *0.755* | *0.75916* | *0.76193* | *0.76817* | *0.77087* | *0.77828* | *0.77996* |
| *37445.22625* | *0.69808* | *0.70221* | *0.70647* | *0.71026* | *0.7142* | *0.71768* | *0.72139* | *0.72691* | *0.73069* | *0.73533* | *0.74224* | *0.74913* | *0.75568* | *0.75979* | *0.76256* | *0.76873* | *0.77138* | *0.77873* | *0.78037* |
| *37440.30085* | *0.69887* | *0.70297* | *0.70719* | *0.71094* | *0.71489* | *0.71836* | *0.72206* | *0.72762* | *0.73139* | *0.73605* | *0.74297* | *0.74986* | *0.7564* | *0.76047* | *0.76325* | *0.76934* | *0.77195* | *0.77923* | *0.78083* |
| *37435.37675* | *0.69971* | *0.70379* | *0.70797* | *0.71169* | *0.71565* | *0.7191* | *0.72277* | *0.7284* | *0.73215* | *0.73682* | *0.74376* | *0.75065* | *0.75718* | *0.76121* | *0.76399* | *0.77001* | *0.77257* | *0.77979* | *0.78135* |
| *37430.45394* | *0.70062* | *0.70468* | *0.70881* | *0.71249* | *0.71646* | *0.7199* | *0.72355* | *0.72924* | *0.73297* | *0.73766* | *0.74461* | *0.75149* | *0.758* | *0.76199* | *0.76478* | *0.77073* | *0.77325* | *0.78041* | *0.78192* |
| *37425.53243* | *0.7016* | *0.70562* | *0.70971* | *0.71335* | *0.71734* | *0.72076* | *0.72439* | *0.73014* | *0.73386* | *0.73856* | *0.74551* | *0.75238* | *0.75888* | *0.76283* | *0.76563* | *0.77151* | *0.77398* | *0.78109* | *0.78255* |
| *37420.61221* | *0.70264* | *0.70664* | *0.71067* | *0.71428* | *0.71828* | *0.72168* | *0.7253* | *0.73111* | *0.7348* | *0.73952* | *0.74648* | *0.75333* | *0.75981* | *0.76371* | *0.76653* | *0.77235* | *0.77478* | *0.78183* | *0.78324* |
| *37415.69329* | *0.70375* | *0.70772* | *0.7117* | *0.71526* | *0.71928* | *0.72267* | *0.72626* | *0.73214* | *0.73582* | *0.74054* | *0.7475* | *0.75434* | *0.7608* | *0.76466* | *0.7675* | *0.77324* | *0.77563* | *0.78263* | *0.78399* |
| *37410.77566* | *0.70493* | *0.70886* | *0.71279* | *0.71631* | *0.72035* | *0.72372* | *0.7273* | *0.73324* | *0.7369* | *0.74163* | *0.74858* | *0.7554* | *0.76184* | *0.76565* | *0.76852* | *0.7742* | *0.77655* | *0.7835* | *0.7848* |
| *37405.85932* | *0.70618* | *0.71008* | *0.71395* | *0.71743* | *0.72149* | *0.72484* | *0.72839* | *0.73441* | *0.73804* | *0.74278* | *0.74972* | *0.75653* | *0.76294* | *0.76671* | *0.7696* | *0.77521* | *0.77753* | *0.78443* | *0.78568* |
| *37400.94427* | *0.7075* | *0.71136* | *0.71518* | *0.71861* | *0.72269* | *0.72602* | *0.72956* | *0.73565* | *0.73926* | *0.744* | *0.75093* | *0.75771* | *0.7641* | *0.76782* | *0.77074* | *0.77629* | *0.77857* | *0.78543* | *0.78661* |
| *37396.03052* | *0.70889* | *0.71272* | *0.71648* | *0.71986* | *0.72396* | *0.72728* | *0.7308* | *0.73696* | *0.74054* | *0.74528* | *0.7522* | *0.75896* | *0.76532* | *0.76898* | *0.77195* | *0.77743* | *0.77967* | *0.78649* | *0.78761* |
| *37391.11805* | *0.71036* | *0.71415* | *0.71784* | *0.72118* | *0.7253* | *0.7286* | *0.7321* | *0.73834* | *0.7419* | *0.74663* | *0.75353* | *0.76027* | *0.76659* | *0.77021* | *0.77321* | *0.77863* | *0.78084* | *0.78762* | *0.78867* |
| *37386.20688* | *0.7119* | *0.71565* | *0.71928* | *0.72257* | *0.72671* | *0.73* | *0.73348* | *0.7398* | *0.74332* | *0.74806* | *0.75493* | *0.76164* | *0.76793* | *0.77149* | *0.77454* | *0.7799* | *0.78207* | *0.78881* | *0.7898* |
| *37381.29699* | *0.71352* | *0.71722* | *0.72079* | *0.72403* | *0.72819* | *0.73146* | *0.73492* | *0.74133* | *0.74482* | *0.74955* | *0.7564* | *0.76307* | *0.76932* | *0.77284* | *0.77593* | *0.78123* | *0.78336* | *0.79007* | *0.791* |
| *37376.3884* | *0.71521* | *0.71887* | *0.72237* | *0.72556* | *0.72975* | *0.733* | *0.73644* | *0.74293* | *0.74638* | *0.75111* | *0.75793* | *0.76457* | *0.77078* | *0.77424* | *0.77738* | *0.78262* | *0.78473* | *0.7914* | *0.79226* |
| *37371.48109* | *0.71698* | *0.72059* | *0.72402* | *0.72717* | *0.73138* | *0.73461* | *0.73804* | *0.7446* | *0.74802* | *0.75274* | *0.75953* | *0.76613* | *0.77229* | *0.77571* | *0.7789* | *0.78408* | *0.78615* | *0.79279* | *0.79359* |
| *37366.57508* | *0.71883* | *0.72239* | *0.72575* | *0.72885* | *0.73308* | *0.7363* | *0.7397* | *0.74635* | *0.74974* | *0.75444* | *0.76119* | *0.76776* | *0.77387* | *0.77724* | *0.78048* | *0.7856* | *0.78765* | *0.79426* | *0.79498* |
| *37361.67035* | *0.72075* | *0.72426* | *0.72756* | *0.7306* | *0.73486* | *0.73806* | *0.74144* | *0.74818* | *0.75152* | *0.75621* | *0.76293* | *0.76945* | *0.77552* | *0.77883* | *0.78213* | *0.78719* | *0.78921* | *0.79579* | *0.79644* |
| *37356.7669* | *0.72275* | *0.72621* | *0.72943* | *0.73242* | *0.73671* | *0.73989* | *0.74326* | *0.75007* | *0.75338* | *0.75806* | *0.76473* | *0.77121* | *0.77722* | *0.78048* | *0.78384* | *0.78885* | *0.79083* | *0.79739* | *0.79797* |
| *37351.86475* | *0.72484* | *0.72824* | *0.73139* | *0.73432* | *0.73863* | *0.7418* | *0.74515* | *0.75205* | *0.75532* | *0.75997* | *0.76659* | *0.77303* | *0.77899* | *0.78219* | *0.78561* | *0.79057* | *0.79253* | *0.79905* | *0.79956* |
| *37346.96388* | *0.727* | *0.73034* | *0.73341* | *0.7363* | *0.74064* | *0.74379* | *0.74712* | *0.7541* | *0.75732* | *0.76196* | *0.76853* | *0.77492* | *0.78082* | *0.78397* | *0.78745* | *0.79235* | *0.79428* | *0.80079* | *0.80122* |
| *37342.0643* | *0.72923* | *0.73252* | *0.73552* | *0.73835* | *0.74271* | *0.74585* | *0.74916* | *0.75622* | *0.7594* | *0.76402* | *0.77053* | *0.77687* | *0.78271* | *0.7858* | *0.78935* | *0.7942* | *0.79611* | *0.80259* | *0.80295* |
| *37337.166* | *0.73155* | *0.73478* | *0.7377* | *0.74047* | *0.74487* | *0.74798* | *0.75128* | *0.75842* | *0.76156* | *0.76615* | *0.7726* | *0.77889* | *0.78467* | *0.7877* | *0.79132* | *0.79612* | *0.798* | *0.80445* | *0.80475* |
| *37332.26899* | *0.73395* | *0.73711* | *0.73996* | *0.74268* | *0.74709* | *0.75019* | *0.75347* | *0.7607* | *0.76379* | *0.76835* | *0.77474* | *0.78097* | *0.78669* | *0.78967* | *0.79335* | *0.7981* | *0.79995* | *0.80639* | *0.80661* |
| *37327.37326* | *0.73642* | *0.73952* | *0.74229* | *0.74495* | *0.7494* | *0.75248* | *0.75574* | *0.76304* | *0.76609* | *0.77062* | *0.77695* | *0.78312* | *0.78877* | *0.79169* | *0.79544* | *0.80014* | *0.80197* | *0.80839* | *0.80853* |
| *37322.47882* | *0.73897* | *0.74201* | *0.7447* | *0.74731* | *0.75178* | *0.75484* | *0.75809* | *0.76547* | *0.76846* | *0.77296* | *0.77922* | *0.78534* | *0.79091* | *0.79378* | *0.7976* | *0.80225* | *0.80406* | *0.81045* | *0.81053* |
| *37317.58566* | *0.7416* | *0.74458* | *0.74718* | *0.74974* | *0.75423* | *0.75728* | *0.76051* | *0.76796* | *0.7709* | *0.77537* | *0.78157* | *0.78761* | *0.79312* | *0.79593* | *0.79982* | *0.80442* | *0.8062* | *0.81258* | *0.81258* |
| *37312.69378* | *0.74431* | *0.74722* | *0.74974* | *0.75224* | *0.75676* | *0.75979* | *0.763* | *0.77053* | *0.77342* | *0.77785* | *0.78397* | *0.78996* | *0.79539* | *0.79814* | *0.8021* | *0.80665* | *0.80841* | *0.81477* | *0.8147* |
| *37307.80318* | *0.7471* | *0.74994* | *0.75238* | *0.75482* | *0.75937* | *0.76238* | *0.76557* | *0.77317* | *0.77601* | *0.7804* | *0.78644* | *0.79236* | *0.79772* | *0.80041* | *0.80444* | *0.80895* | *0.81069* | *0.81702* | *0.81689* |
| *37302.91387* | *0.74996* | *0.75273* | *0.75509* | *0.75747* | *0.76205* | *0.76504* | *0.76822* | *0.77588* | *0.77866* | *0.78302* | *0.78898* | *0.79483* | *0.80011* | *0.80274* | *0.80684* | *0.8113* | *0.81302* | *0.81934* | *0.81913* |
| *37298.02584* | *0.75289* | *0.75559* | *0.75787* | *0.7602* | *0.7648* | *0.76777* | *0.77093* | *0.77866* | *0.78139* | *0.7857* | *0.79158* | *0.79736* | *0.80256* | *0.80513* | *0.8093* | *0.81372* | *0.81542* | *0.82171* | *0.82144* |
| *37293.13909* | *0.75591* | *0.75853* | *0.76073* | *0.763* | *0.76762* | *0.77058* | *0.77372* | *0.78151* | *0.78418* | *0.78845* | *0.79424* | *0.79995* | *0.80506* | *0.80757* | *0.81182* | *0.81619* | *0.81787* | *0.82414* | *0.82381* |
| *37288.25362* | *0.75899* | *0.76155* | *0.76366* | *0.76587* | *0.77052* | *0.77345* | *0.77658* | *0.78443* | *0.78704* | *0.79127* | *0.79697* | *0.8026* | *0.80763* | *0.81008* | *0.81439* | *0.81873* | *0.82038* | *0.82663* | *0.82624* |
| *37283.36943* | *0.76215* | *0.76463* | *0.76666* | *0.76882* | *0.77348* | *0.7764* | *0.77951* | *0.78741* | *0.78997* | *0.79415* | *0.79976* | *0.80531* | *0.81026* | *0.81264* | *0.81703* | *0.82132* | *0.82295* | *0.82918* | *0.82872* |
| *37278.48651* | *0.76538* | *0.76778* | *0.76973* | *0.77183* | *0.77652* | *0.77941* | *0.78251* | *0.79046* | *0.79296* | *0.79709* | *0.8026* | *0.80807* | *0.81293* | *0.81526* | *0.81971* | *0.82396* | *0.82557* | *0.83178* | *0.83126* |
| *37273.60488* | *0.76868* | *0.77101* | *0.77287* | *0.77492* | *0.77962* | *0.78249* | *0.78558* | *0.79357* | *0.79601* | *0.80009* | *0.80551* | *0.81089* | *0.81567* | *0.81793* | *0.82245* | *0.82666* | *0.82825* | *0.83443* | *0.83386* |
| *37268.72453* | *0.77204* | *0.7743* | *0.77608* | *0.77807* | *0.78279* | *0.78564* | *0.78871* | *0.79674* | *0.79913* | *0.80315* | *0.80847* | *0.81377* | *0.81846* | *0.82066* | *0.82524* | *0.82941* | *0.83098* | *0.83714* | *0.83651* |
| *37263.84545* | *0.77548* | *0.77765* | *0.77935* | *0.78129* | *0.78602* | *0.78886* | *0.7919* | *0.79998* | *0.8023* | *0.80627* | *0.81148* | *0.8167* | *0.8213* | *0.82344* | *0.82808* | *0.83221* | *0.83376* | *0.83989* | *0.83921* |
| *37258.96765* | *0.77898* | *0.78108* | *0.7827* | *0.78457* | *0.78932* | *0.79213* | *0.79516* | *0.80327* | *0.80553* | *0.80944* | *0.81455* | *0.81968* | *0.82419* | *0.82627* | *0.83097* | *0.83506* | *0.83659* | *0.84269* | *0.84196* |
| *37254.09113* | *0.78254* | *0.78456* | *0.7861* | *0.78792* | *0.79269* | *0.79547* | *0.79849* | *0.80662* | *0.80882* | *0.81267* | *0.81768* | *0.82272* | *0.82713* | *0.82915* | *0.83391* | *0.83795* | *0.83946* | *0.84554* | *0.84476* |
| *37249.21588* | *0.78617* | *0.78811* | *0.78956* | *0.79133* | *0.79611* | *0.79887* | *0.80187* | *0.81002* | *0.81216* | *0.81595* | *0.82085* | *0.8258* | *0.83012* | *0.83207* | *0.83689* | *0.8409* | *0.84239* | *0.84843* | *0.84761* |
| *37244.34191* | *0.78986* | *0.79171* | *0.79309* | *0.7948* | *0.79959* | *0.80233* | *0.80531* | *0.81348* | *0.81555* | *0.81928* | *0.82407* | *0.82893* | *0.83315* | *0.83505* | *0.83992* | *0.84388* | *0.84535* | *0.85136* | *0.85049* |
| *37239.46922* | *0.7936* | *0.79538* | *0.79667* | *0.79833* | *0.80312* | *0.80584* | *0.8088* | *0.81698* | *0.81899* | *0.82266* | *0.82734* | *0.8321* | *0.83623* | *0.83806* | *0.84298* | *0.84691* | *0.84836* | *0.85434* | *0.85343* |
| *37234.59779* | *0.7974* | *0.79909* | *0.80031* | *0.80192* | *0.80672* | *0.8094* | *0.81235* | *0.82053* | *0.82248* | *0.82609* | *0.83065* | *0.83532* | *0.83935* | *0.84112* | *0.84609* | *0.84998* | *0.8514* | *0.85734* | *0.8564* |
| *37229.72765* | *0.80125* | *0.80287* | *0.80401* | *0.80556* | *0.81036* | *0.81302* | *0.81595* | *0.82413* | *0.82602* | *0.82956* | *0.83401* | *0.83858* | *0.84251* | *0.84422* | *0.84924* | *0.85308* | *0.85448* | *0.86039* | *0.85941* |
| *37224.85878* | *0.80516* | *0.80669* | *0.80775* | *0.80925* | *0.81405* | *0.81669* | *0.8196* | *0.82777* | *0.8296* | *0.83307* | *0.8374* | *0.84188* | *0.84571* | *0.84736* | *0.85241* | *0.85622* | *0.8576* | *0.86346* | *0.86245* |
| *37219.99118* | *0.80911* | *0.81056* | *0.81155* | *0.813* | *0.81779* | *0.8204* | *0.82329* | *0.83146* | *0.83321* | *0.83661* | *0.84083* | *0.84521* | *0.84895* | *0.85053* | *0.85563* | *0.85939* | *0.86075* | *0.86657* | *0.86553* |
| *37215.12485* | *0.81311* | *0.81448* | *0.81539* | *0.81679* | *0.82158* | *0.82416* | *0.82703* | *0.83518* | *0.83687* | *0.8402* | *0.8443* | *0.84858* | *0.85222* | *0.85374* | *0.85887* | *0.8626* | *0.86393* | *0.8697* | *0.86864* |
| *37210.2598* | *0.81715* | *0.81844* | *0.81928* | *0.82063* | *0.82541* | *0.82796* | *0.83081* | *0.83893* | *0.84056* | *0.84382* | *0.8478* | *0.85198* | *0.85552* | *0.85698* | *0.86214* | *0.86583* | *0.86714* | *0.87286* | *0.87178* |
| *37205.39602* | *0.82123* | *0.82244* | *0.82321* | *0.8245* | *0.82928* | *0.8318* | *0.83463* | *0.84272* | *0.84428* | *0.84747* | *0.85134* | *0.85541* | *0.85885* | *0.86026* | *0.86544* | *0.86908* | *0.87037* | *0.87604* | *0.87494* |
| *37200.53351* | *0.82535* | *0.82648* | *0.82718* | *0.82842* | *0.83319* | *0.83568* | *0.83848* | *0.84654* | *0.84803* | *0.85115* | *0.8549* | *0.85886* | *0.86221* | *0.86355* | *0.86876* | *0.87236* | *0.87362* | *0.87924* | *0.87813* |
| *37195.67227* | *0.8295* | *0.83055* | *0.83118* | *0.83238* | *0.83713* | *0.83958* | *0.84237* | *0.85038* | *0.85181* | *0.85485* | *0.85848* | *0.86234* | *0.8656* | *0.86688* | *0.8721* | *0.87566* | *0.8769* | *0.88245* | *0.88133* |
| *37190.8123* | *0.83369* | *0.83466* | *0.83522* | *0.83637* | *0.8411* | *0.84352* | *0.84629* | *0.85424* | *0.85561* | *0.85858* | *0.86209* | *0.86585* | *0.869* | *0.87022* | *0.87546* | *0.87898* | *0.88019* | *0.88569* | *0.88456* |
| *37185.9536* | *0.8379* | *0.83879* | *0.83929* | *0.84039* | *0.8451* | *0.84749* | *0.85023* | *0.85813* | *0.85944* | *0.86233* | *0.86572* | *0.86937* | *0.87243* | *0.87359* | *0.87883* | *0.88231* | *0.88349* | *0.88893* | *0.88779* |
| *37181.09617* | *0.84214* | *0.84295* | *0.84338* | *0.84444* | *0.84912* | *0.85148* | *0.8542* | *0.86203* | *0.86328* | *0.86609* | *0.86936* | *0.87291* | *0.87587* | *0.87697* | *0.88222* | *0.88565* | *0.88681* | *0.89218* | *0.89104* |
| *37176.24001* | *0.8464* | *0.84713* | *0.8475* | *0.84852* | *0.85317* | *0.85549* | *0.85818* | *0.86595* | *0.86713* | *0.86987* | *0.87302* | *0.87646* | *0.87932* | *0.88037* | *0.88561* | *0.889* | *0.89013* | *0.89543* | *0.8943* |
| *37171.38511* | *0.85068* | *0.85133* | *0.85165* | *0.85261* | *0.85724* | *0.85952* | *0.86219* | *0.86987* | *0.87099* | *0.87366* | *0.87669* | *0.88002* | *0.88279* | *0.88378* | *0.88901* | *0.89236* | *0.89346* | *0.89869* | *0.89756* |
| *37166.53149* | *0.85498* | *0.85555* | *0.8558* | *0.85673* | *0.86132* | *0.86357* | *0.86621* | *0.87381* | *0.87487* | *0.87745* | *0.88036* | *0.88359* | *0.88626* | *0.8872* | *0.89242* | *0.89572* | *0.8968* | *0.90195* | *0.90083* |
| *37161.67913* | *0.85928* | *0.85978* | *0.85998* | *0.86086* | *0.86541* | *0.86762* | *0.87023* | *0.87774* | *0.87874* | *0.88125* | *0.88404* | *0.88716* | *0.88974* | *0.89062* | *0.89583* | *0.89908* | *0.90013* | *0.9052* | *0.90409* |
| *37156.82804* | *0.86359* | *0.86402* | *0.86416* | *0.865* | *0.86951* | *0.87169* | *0.87427* | *0.88168* | *0.88262* | *0.88505* | *0.88772* | *0.89074* | *0.89322* | *0.89405* | *0.89923* | *0.90244* | *0.90346* | *0.90845* | *0.90736* |
| *37151.97821* | *0.86791* | *0.86826* | *0.86835* | *0.86915* | *0.87362* | *0.87575* | *0.8783* | *0.88561* | *0.88649* | *0.88884* | *0.8914* | *0.89431* | *0.8967* | *0.89747* | *0.90263* | *0.90579* | *0.90678* | *0.91169* | *0.91061* |
| *37147.12966* | *0.87222* | *0.8725* | *0.87254* | *0.8733* | *0.87772* | *0.87982* | *0.88234* | *0.88954* | *0.89036* | *0.89263* | *0.89507* | *0.89788* | *0.90018* | *0.90089* | *0.90602* | *0.90913* | *0.91009* | *0.91491* | *0.91385* |
| *37142.28236* | *0.87654* | *0.87675* | *0.87673* | *0.87745* | *0.88183* | *0.88388* | *0.88637* | *0.89345* | *0.89422* | *0.89641* | *0.89874* | *0.90144* | *0.90365* | *0.90431* | *0.9094* | *0.91246* | *0.91339* | *0.91812* | *0.91709* |
| *37137.43633* | *0.88084* | *0.88098* | *0.88092* | *0.8816* | *0.88593* | *0.88793* | *0.89039* | *0.89736* | *0.89806* | *0.90017* | *0.90239* | *0.90499* | *0.90711* | *0.90772* | *0.91277* | *0.91578* | *0.91668* | *0.92131* | *0.9203* |
| *37132.59157* | *0.88513* | *0.88521* | *0.8851* | *0.88575* | *0.89001* | *0.89198* | *0.8944* | *0.90124* | *0.90189* | *0.90392* | *0.90603* | *0.90852* | *0.91055* | *0.91111* | *0.91612* | *0.91907* | *0.91994* | *0.92448* | *0.9235* |
| *37127.74807* | *0.88941* | *0.88942* | *0.88927* | *0.88988* | *0.89408* | *0.89601* | *0.89839* | *0.9051* | *0.90569* | *0.90765* | *0.90965* | *0.91203* | *0.91398* | *0.91449* | *0.91944* | *0.92234* | *0.92318* | *0.92762* | *0.92667* |
| *37122.90583* | *0.89366* | *0.89361* | *0.89342* | *0.89399* | *0.89814* | *0.90002* | *0.90236* | *0.90893* | *0.90947* | *0.91135* | *0.91324* | *0.91553* | *0.91739* | *0.91785* | *0.92275* | *0.92559* | *0.9264* | *0.93073* | *0.92981* |
| *37118.06486* | *0.89789* | *0.89778* | *0.89756* | *0.89809* | *0.90217* | *0.904* | *0.90631* | *0.91274* | *0.91323* | *0.91503* | *0.91681* | *0.91899* | *0.92077* | *0.92119* | *0.92602* | *0.92881* | *0.92958* | *0.93382* | *0.93293* |
| *37113.22515* | *0.9021* | *0.90192* | *0.90166* | *0.90217* | *0.90618* | *0.90796* | *0.91023* | *0.91651* | *0.91695* | *0.91867* | *0.92035* | *0.92243* | *0.92413* | *0.92451* | *0.92927* | *0.932* | *0.93274* | *0.93686* | *0.93601* |
| *37108.3867* | *0.90627* | *0.90604* | *0.90574* | *0.90622* | *0.91015* | *0.91189* | *0.91412* | *0.92024* | *0.92063* | *0.92228* | *0.92386* | *0.92584* | *0.92746* | *0.92779* | *0.93248* | *0.93515* | *0.93586* | *0.93987* | *0.93906* |
| *37103.54951* | *0.9104* | *0.91012* | *0.90979* | *0.91023* | *0.91409* | *0.91578* | *0.91797* | *0.92394* | *0.92428* | *0.92585* | *0.92733* | *0.92922* | *0.93075* | *0.93104* | *0.93565* | *0.93827* | *0.93894* | *0.94284* | *0.94207* |
| *37098.71358* | *0.91449* | *0.91416* | *0.9138* | *0.91421* | *0.918* | *0.91964* | *0.92178* | *0.92758* | *0.92788* | *0.92938* | *0.93076* | *0.93255* | *0.93401* | *0.93426* | *0.93878* | *0.94134* | *0.94198* | *0.94577* | *0.94503* |
| *37093.87892* | *0.91854* | *0.91816* | *0.91777* | *0.91815* | *0.92186* | *0.92345* | *0.92554* | *0.93118* | *0.93144* | *0.93286* | *0.93415* | *0.93584* | *0.93722* | *0.93743* | *0.94187* | *0.94437* | *0.94498* | *0.94865* | *0.94795* |
| *37089.04551* | *0.92254* | *0.92211* | *0.9217* | *0.92205* | *0.92567* | *0.92721* | *0.92926* | *0.93473* | *0.93494* | *0.9363* | *0.93749* | *0.93909* | *0.9404* | *0.94057* | *0.94492* | *0.94735* | *0.94792* | *0.95148* | *0.95082* |
| *37084.21336* | *0.92648* | *0.92601* | *0.92558* | *0.9259* | *0.92943* | *0.93093* | *0.93293* | *0.93822* | *0.93839* | *0.93967* | *0.94078* | *0.94229* | *0.94352* | *0.94366* | *0.94791* | *0.95027* | *0.95082* | *0.95425* | *0.95364* |
| *37079.38247* | *0.93037* | *0.92985* | *0.9294* | *0.9297* | *0.93314* | *0.93458* | *0.93654* | *0.94166* | *0.94178* | *0.943* | *0.94402* | *0.94543* | *0.9466* | *0.9467* | *0.95085* | *0.95315* | *0.95366* | *0.95697* | *0.9564* |
| *37074.55284* | *0.93419* | *0.93363* | *0.93317* | *0.93344* | *0.93679* | *0.93818* | *0.94008* | *0.94502* | *0.94511* | *0.94626* | *0.94719* | *0.94852* | *0.94962* | *0.94968* | *0.95373* | *0.95596* | *0.95644* | *0.95964* | *0.95911* |
| *37069.72447* | *0.93794* | *0.93735* | *0.93687* | *0.93712* | *0.94038* | *0.94172* | *0.94357* | *0.94832* | *0.94838* | *0.94946* | *0.95031* | *0.95155* | *0.95258* | *0.95261* | *0.95655* | *0.95872* | *0.95916* | *0.96223* | *0.96175* |
| *37064.89736* | *0.94163* | *0.94101* | *0.94051* | *0.94074* | *0.9439* | *0.94518* | *0.94698* | *0.95155* | *0.95157* | *0.95259* | *0.95336* | *0.95452* | *0.95548* | *0.95549* | *0.95931* | *0.96141* | *0.96182* | *0.96477* | *0.96432* |
| *37060.0715* | *0.94524* | *0.94459* | *0.94408* | *0.94428* | *0.94734* | *0.94858* | *0.95032* | *0.95471* | *0.95469* | *0.95564* | *0.95635* | *0.95742* | *0.95832* | *0.9583* | *0.962* | *0.96403* | *0.96442* | *0.96724* | *0.96683* |
| *37055.2469* | *0.94877* | *0.94809* | *0.94757* | *0.94776* | *0.95072* | *0.9519* | *0.95359* | *0.95778* | *0.95774* | *0.95863* | *0.95926* | *0.96025* | *0.96109* | *0.96104* | *0.96463* | *0.96659* | *0.96694* | *0.96963* | *0.96927* |
| *37050.42355* | *0.95221* | *0.95151* | *0.95099* | *0.95115* | *0.95401* | *0.95514* | *0.95677* | *0.96078* | *0.96071* | *0.96153* | *0.96209* | *0.96301* | *0.96379* | *0.96372* | *0.96718* | *0.96907* | *0.96939* | *0.97196* | *0.97164* |
| *37045.60146* | *0.95557* | *0.95485* | *0.95432* | *0.95447* | *0.95722* | *0.9583* | *0.95987* | *0.96369* | *0.96359* | *0.96436* | *0.96485* | *0.96569* | *0.96642* | *0.96632* | *0.96966* | *0.97147* | *0.97176* | *0.9742* | *0.97392* |
| *37040.78063* | *0.95884* | *0.9581* | *0.95757* | *0.9577* | *0.96034* | *0.96137* | *0.96288* | *0.96651* | *0.96639* | *0.96709* | *0.96753* | *0.9683* | *0.96897* | *0.96885* | *0.97206* | *0.97379* | *0.97406* | *0.97637* | *0.97613* |
| *37035.96105* | *0.96201* | *0.96125* | *0.96072* | *0.96084* | *0.96337* | *0.96435* | *0.9658* | *0.96923* | *0.96909* | *0.96974* | *0.97012* | *0.97082* | *0.97144* | *0.97131* | *0.97438* | *0.97604* | *0.97628* | *0.97846* | *0.97826* |
| *37031.14272* | *0.96507* | *0.96431* | *0.96379* | *0.96388* | *0.96631* | *0.96723* | *0.96862* | *0.97187* | *0.97171* | *0.9723* | *0.97262* | *0.97326* | *0.97383* | *0.97368* | *0.97661* | *0.9782* | *0.97841* | *0.98047* | *0.9803* |
| *37026.32565* | *0.96804* | *0.96727* | *0.96675* | *0.96683* | *0.96914* | *0.97001* | *0.97135* | *0.9744* | *0.97422* | *0.97477* | *0.97504* | *0.97561* | *0.97613* | *0.97596* | *0.97876* | *0.98027* | *0.98045* | *0.98239* | *0.98225* |
| *37021.50983* | *0.97089* | *0.97013* | *0.96961* | *0.96968* | *0.97188* | *0.9727* | *0.97397* | *0.97683* | *0.97664* | *0.97713* | *0.97735* | *0.97786* | *0.97834* | *0.97816* | *0.98082* | *0.98225* | *0.98241* | *0.98422* | *0.98412* |
| *37016.69526* | *0.97364* | *0.97287* | *0.97236* | *0.97242* | *0.9745* | *0.97527* | *0.97648* | *0.97916* | *0.97895* | *0.9794* | *0.97958* | *0.98003* | *0.98046* | *0.98027* | *0.98279* | *0.98414* | *0.98427* | *0.98596* | *0.98589* |
| *37011.88195* | *0.97627* | *0.97551* | *0.97501* | *0.97505* | *0.97702* | *0.97774* | *0.97888* | *0.98137* | *0.98116* | *0.98156* | *0.9817* | *0.98209* | *0.98248* | *0.98229* | *0.98466* | *0.98593* | *0.98605* | *0.98761* | *0.98756* |
| *37007.06988* | *0.97877* | *0.97802* | *0.97754* | *0.97757* | *0.97943* | *0.98009* | *0.98117* | *0.98348* | *0.98326* | *0.98362* | *0.98371* | *0.98406* | *0.98441* | *0.98421* | *0.98643* | *0.98763* | *0.98772* | *0.98916* | *0.98914* |
| *37002.25907* | *0.98116* | *0.98042* | *0.97995* | *0.97997* | *0.98171* | *0.98233* | *0.98334* | *0.98547* | *0.98524* | *0.98556* | *0.98563* | *0.98592* | *0.98624* | *0.98603* | *0.98811* | *0.98922* | *0.9893* | *0.99062* | *0.99062* |
| *36997.44951* | *0.98342* | *0.9827* | *0.98224* | *0.98225* | *0.98388* | *0.98445* | *0.9854* | *0.98734* | *0.98711* | *0.98739* | *0.98743* | *0.98768* | *0.98796* | *0.98775* | *0.98969* | *0.99072* | *0.99077* | *0.99198* | *0.992* |
| *36992.6412* | *0.98555* | *0.98484* | *0.98441* | *0.98441* | *0.98592* | *0.98645* | *0.98733* | *0.9891* | *0.98887* | *0.98911* | *0.98912* | *0.98933* | *0.98958* | *0.98937* | *0.99116* | *0.99211* | *0.99215* | *0.99324* | *0.99328* |
| *36987.83414* | *0.98754* | *0.98686* | *0.98645* | *0.98644* | *0.98784* | *0.98832* | *0.98913* | *0.99073* | *0.9905* | *0.99071* | *0.9907* | *0.99087* | *0.99108* | *0.99088* | *0.99252* | *0.99339* | *0.99342* | *0.99439* | *0.99445* |
| *36983.02833* | *0.9894* | *0.98875* | *0.98835* | *0.98834* | *0.98963* | *0.99006* | *0.9908* | *0.99224* | *0.99202* | *0.99219* | *0.99216* | *0.9923* | *0.99248* | *0.99228* | *0.99378* | *0.99457* | *0.99458* | *0.99544* | *0.99551* |
| *36978.22376* | *0.99112* | *0.9905* | *0.99013* | *0.99011* | *0.99128* | *0.99167* | *0.99235* | *0.99362* | *0.99341* | *0.99355* | *0.99351* | *0.99361* | *0.99377* | *0.99357* | *0.99492* | *0.99563* | *0.99563* | *0.99639* | *0.99646* |
| *36973.42045* | *0.99269* | *0.99211* | *0.99176* | *0.99175* | *0.9928* | *0.99314* | *0.99375* | *0.99487* | *0.99467* | *0.99479* | *0.99473* | *0.99481* | *0.99494* | *0.99475* | *0.99596* | *0.99659* | *0.99658* | *0.99723* | *0.9973* |
| *36968.61838* | *0.99412* | *0.99358* | *0.99326* | *0.99324* | *0.99418* | *0.99448* | *0.99502* | *0.99599* | *0.9958* | *0.9959* | *0.99583* | *0.99588* | *0.99599* | *0.99582* | *0.99688* | *0.99742* | *0.99741* | *0.99796* | *0.99804* |
| *36963.81756* | *0.9954* | *0.9949* | *0.99461* | *0.99459* | *0.99542* | *0.99568* | *0.99616* | *0.99698* | *0.9968* | *0.99688* | *0.99681* | *0.99684* | *0.99693* | *0.99677* | *0.99768* | *0.99815* | *0.99813* | *0.99858* | *0.99865* |
| *36959.01798* | *0.99653* | *0.99608* | *0.99582* | *0.9958* | *0.99652* | *0.99674* | *0.99715* | *0.99783* | *0.99767* | *0.99773* | *0.99765* | *0.99767* | *0.99774* | *0.9976* | *0.99837* | *0.99876* | *0.99873* | *0.99909* | *0.99916* |
| *36954.21966* | *0.9975* | *0.99711* | *0.99688* | *0.99686* | *0.99747* | *0.99765* | *0.99799* | *0.99854* | *0.9984* | *0.99844* | *0.99838* | *0.99838* | *0.99843* | *0.99831* | *0.99894* | *0.99925* | *0.99922* | *0.99949* | *0.99954* |
| *36949.42257* | *0.99832* | *0.99798* | *0.99779* | *0.99777* | *0.99827* | *0.99842* | *0.99869* | *0.99912* | *0.999* | *0.99903* | *0.99897* | *0.99896* | *0.999* | *0.9989* | *0.99939* | *0.99962* | *0.9996* | *0.99977* | *0.99981* |
| *36944.62674* | *0.99898* | *0.9987* | *0.99855* | *0.99853* | *0.99892* | *0.99904* | *0.99924* | *0.99955* | *0.99946* | *0.99948* | *0.99943* | *0.99942* | *0.99944* | *0.99936* | *0.99971* | *0.99987* | *0.99985* | *0.99994* | *0.99997* |
| *36939.83214* | *0.99948* | *0.99927* | *0.99915* | *0.99913* | *0.99943* | *0.99951* | *0.99965* | *0.99984* | *0.99978* | *0.99979* | *0.99975* | *0.99974* | *0.99976* | *0.9997* | *0.99992* | *0.99999* | *0.99998* | *1* | *1* |
| *36935.0388* | *0.99982* | *0.99967* | *0.99959* | *0.99958* | *0.99977* | *0.99983* | *0.9999* | *0.99999* | *0.99996* | *0.99996* | *0.99994* | *0.99994* | *0.99994* | *0.99991* | *1* | *1* | *1* | *0.99994* | *0.99991* |
| *36930.24669* | *0.99999* | *0.99992* | *0.99988* | *0.99987* | *0.99996* | *0.99999* | *1* | *1* | *1* | *1* | *1* | *1* | *1* | *1* | *0.99996* | *0.99988* | *0.99989* | *0.99977* | *0.99971* |
| *36925.45583* | *1* | *1* | *1* | *1* | *1* | *1* | *0.99995* | *0.99986* | *0.9999* | *0.9999* | *0.99992* | *0.99993* | *0.99993* | *0.99996* | *0.99979* | *0.99964* | *0.99967* | *0.99948* | *0.99938* |
| *36920.66621* | *0.99984* | *0.99992* | *0.99996* | *0.99997* | *0.99988* | *0.99985* | *0.99974* | *0.99958* | *0.99965* | *0.99965* | *0.9997* | *0.99973* | *0.99972* | *0.99979* | *0.9995* | *0.99928* | *0.99932* | *0.99907* | *0.99894* |
| *36915.87784* | *0.99951* | *0.99967* | *0.99976* | *0.99978* | *0.99959* | *0.99955* | *0.99937* | *0.99914* | *0.99926* | *0.99926* | *0.99935* | *0.99939* | *0.99938* | *0.99948* | *0.99908* | *0.99879* | *0.99885* | *0.99854* | *0.99837* |
| *36911.09071* | *0.99901* | *0.99926* | *0.9994* | *0.99942* | *0.99915* | *0.99909* | *0.99885* | *0.99857* | *0.99872* | *0.99874* | *0.99885* | *0.99892* | *0.99891* | *0.99905* | *0.99853* | *0.99817* | *0.99826* | *0.9979* | *0.99769* |
| *36906.30481* | *0.99834* | *0.99868* | *0.99886* | *0.9989* | *0.99855* | *0.99847* | *0.99817* | *0.99784* | *0.99804* | *0.99806* | *0.99822* | *0.99831* | *0.99831* | *0.99849* | *0.99786* | *0.99743* | *0.99754* | *0.99714* | *0.99688* |
| *36901.52016* | *0.9975* | *0.99794* | *0.99817* | *0.99822* | *0.99778* | *0.99769* | *0.99734* | *0.99697* | *0.99721* | *0.99725* | *0.99744* | *0.99757* | *0.99757* | *0.99779* | *0.99706* | *0.99657* | *0.99671* | *0.99627* | *0.99595* |
| *36896.73675* | *0.99649* | *0.99702* | *0.9973* | *0.99736* | *0.99686* | *0.99675* | *0.99635* | *0.99595* | *0.99624* | *0.99629* | *0.99653* | *0.99669* | *0.99669* | *0.99697* | *0.99613* | *0.99558* | *0.99575* | *0.99528* | *0.9949* |
| *36891.95458* | *0.99531* | *0.99594* | *0.99627* | *0.99635* | *0.99577* | *0.99566* | *0.99519* | *0.99478* | *0.99512* | *0.99519* | *0.99548* | *0.99568* | *0.99569* | *0.99601* | *0.99507* | *0.99446* | *0.99467* | *0.99417* | *0.99373* |
| *36887.17365* | *0.99395* | *0.99469* | *0.99507* | *0.99516* | *0.99451* | *0.9944* | *0.99388* | *0.99346* | *0.99386* | *0.99395* | *0.99428* | *0.99453* | *0.99455* | *0.99492* | *0.99389* | *0.99322* | *0.99347* | *0.99295* | *0.99245* |
| *36882.39396* | *0.99242* | *0.99326* | *0.9937* | *0.99381* | *0.9931* | *0.99298* | *0.99242* | *0.992* | *0.99245* | *0.99256* | *0.99295* | *0.99324* | *0.99327* | *0.99369* | *0.99259* | *0.99186* | *0.99214* | *0.99161* | *0.99104* |
| *36877.6155* | *0.99071* | *0.99167* | *0.99216* | *0.99229* | *0.99152* | *0.9914* | *0.99079* | *0.99039* | *0.99089* | *0.99104* | *0.99148* | *0.99182* | *0.99186* | *0.99234* | *0.99115* | *0.99038* | *0.9907* | *0.99016* | *0.98952* |
| *36872.83829* | *0.98884* | *0.98991* | *0.99045* | *0.9906* | *0.98978* | *0.98966* | *0.98901* | *0.98863* | *0.9892* | *0.98937* | *0.98986* | *0.99027* | *0.99032* | *0.99086* | *0.98959* | *0.98877* | *0.98914* | *0.98859* | *0.98788* |
| *36868.06231* | *0.98679* | *0.98798* | *0.98858* | *0.98875* | *0.98787* | *0.98777* | *0.98707* | *0.98673* | *0.98736* | *0.98756* | *0.98811* | *0.98858* | *0.98865* | *0.98924* | *0.98791* | *0.98704* | *0.98746* | *0.98691* | *0.98613* |
| *36863.28757* | *0.98457* | *0.98588* | *0.98654* | *0.98673* | *0.98581* | *0.98572* | *0.98498* | *0.98468* | *0.98537* | *0.98561* | *0.98623* | *0.98675* | *0.98684* | *0.98749* | *0.9861* | *0.98519* | *0.98566* | *0.98512* | *0.98426* |
| *36858.51406* | *0.98218* | *0.98361* | *0.98433* | *0.98455* | *0.98359* | *0.98351* | *0.98273* | *0.98249* | *0.98325* | *0.98352* | *0.9842* | *0.9848* | *0.98491* | *0.98562* | *0.98417* | *0.98322* | *0.98374* | *0.98322* | *0.98228* |
| *36853.7418* | *0.97962* | *0.98118* | *0.98195* | *0.9822* | *0.9812* | *0.98114* | *0.98033* | *0.98016* | *0.98098* | *0.98129* | *0.98204* | *0.98271* | *0.98284* | *0.98362* | *0.98212* | *0.98113* | *0.98171* | *0.98121* | *0.98019* |
| *36848.97076* | *0.97689* | *0.97858* | *0.97942* | *0.97969* | *0.97866* | *0.97862* | *0.97778* | *0.97769* | *0.97858* | *0.97893* | *0.97975* | *0.98049* | *0.98064* | *0.98149* | *0.97995* | *0.97893* | *0.97957* | *0.9791* | *0.97799* |
| *36844.20097* | *0.974* | *0.97582* | *0.97671* | *0.97702* | *0.97597* | *0.97595* | *0.97508* | *0.97508* | *0.97603* | *0.97643* | *0.97732* | *0.97814* | *0.97832* | *0.97923* | *0.97766* | *0.97661* | *0.97731* | *0.97687* | *0.97568* |
| *36839.4324* | *0.97094* | *0.97289* | *0.97385* | *0.97419* | *0.97311* | *0.97312* | *0.97223* | *0.97233* | *0.97336* | *0.97379* | *0.97476* | *0.97567* | *0.97587* | *0.97686* | *0.97525* | *0.97417* | *0.97494* | *0.97455* | *0.97326* |
| *36834.66507* | *0.96771* | *0.9698* | *0.97083* | *0.9712* | *0.97011* | *0.97015* | *0.96923* | *0.96945* | *0.97054* | *0.97103* | *0.97208* | *0.97307* | *0.9733* | *0.97435* | *0.97272* | *0.97163* | *0.97247* | *0.97212* | *0.97074* |
| *36829.89898* | *0.96433* | *0.96655* | *0.96765* | *0.96805* | *0.96695* | *0.96702* | *0.96609* | *0.96643* | *0.9676* | *0.96813* | *0.96926* | *0.97034* | *0.9706* | *0.97173* | *0.97009* | *0.96897* | *0.96988* | *0.96958* | *0.96812* |
| *36825.13412* | *0.96078* | *0.96315* | *0.96431* | *0.96475* | *0.96365* | *0.96376* | *0.96281* | *0.96328* | *0.96453* | *0.96511* | *0.96632* | *0.96749* | *0.96779* | *0.96899* | *0.96734* | *0.96621* | *0.96719* | *0.96695* | *0.9654* |
| *36820.37049* | *0.95708* | *0.95959* | *0.96082* | *0.96129* | *0.9602* | *0.96035* | *0.95939* | *0.96* | *0.96132* | *0.96196* | *0.96325* | *0.96453* | *0.96485* | *0.96613* | *0.96448* | *0.96334* | *0.9644* | *0.96423* | *0.96258* |
| *36815.60809* | *0.95322* | *0.95588* | *0.95718* | *0.95769* | *0.9566* | *0.95679* | *0.95583* | *0.9566* | *0.958* | *0.95869* | *0.96006* | *0.96144* | *0.9618* | *0.96316* | *0.96151* | *0.96037* | *0.96151* | *0.9614* | *0.95966* |
| *36810.84693* | *0.94922* | *0.95201* | *0.95338* | *0.95394* | *0.95286* | *0.9531* | *0.95213* | *0.95307* | *0.95454* | *0.95529* | *0.95675* | *0.95824* | *0.95864* | *0.96008* | *0.95843* | *0.9573* | *0.95852* | *0.95849* | *0.95665* |
| *36806.08699* | *0.94506* | *0.948* | *0.94945* | *0.95004* | *0.94899* | *0.94928* | *0.94831* | *0.94942* | *0.95097* | *0.95178* | *0.95333* | *0.95492* | *0.95536* | *0.95688* | *0.95526* | *0.95413* | *0.95543* | *0.95548* | *0.95356* |
| *36801.32829* | *0.94075* | *0.94385* | *0.94536* | *0.946* | *0.94498* | *0.94532* | *0.94436* | *0.94565* | *0.94728* | *0.94815* | *0.94979* | *0.9515* | *0.95197* | *0.95358* | *0.95198* | *0.95086* | *0.95225* | *0.95239* | *0.95037* |
| *36796.57082* | *0.93631* | *0.93955* | *0.94114* | *0.94183* | *0.94083* | *0.94124* | *0.94028* | *0.94176* | *0.94348* | *0.94441* | *0.94614* | *0.94796* | *0.94848* | *0.95017* | *0.9486* | *0.9475* | *0.94897* | *0.94921* | *0.9471* |
| *36791.81457* | *0.93172* | *0.93512* | *0.93678* | *0.93751* | *0.93656* | *0.93702* | *0.93608* | *0.93776* | *0.93956* | *0.94055* | *0.94237* | *0.94432* | *0.94488* | *0.94666* | *0.94513* | *0.94404* | *0.94561* | *0.94594* | *0.94375* |
| *36787.05956* | *0.927* | *0.93055* | *0.93229* | *0.93307* | *0.93216* | *0.93269* | *0.93176* | *0.93365* | *0.93553* | *0.93659* | *0.93851* | *0.94058* | *0.94118* | *0.94304* | *0.94157* | *0.94051* | *0.94217* | *0.9426* | *0.94032* |
| *36782.30578* | *0.92214* | *0.92585* | *0.92767* | *0.9285* | *0.92763* | *0.92824* | *0.92733* | *0.92944* | *0.9314* | *0.93253* | *0.93454* | *0.93673* | *0.93739* | *0.93934* | *0.93791* | *0.93688* | *0.93864* | *0.93918* | *0.93681* |
| *36777.55322* | *0.91716* | *0.92102* | *0.92292* | *0.9238* | *0.92299* | *0.92367* | *0.92278* | *0.92512* | *0.92716* | *0.92836* | *0.93047* | *0.93279* | *0.93349* | *0.93553* | *0.93417* | *0.93318* | *0.93503* | *0.93568* | *0.93323* |
| *36772.80189* | *0.91205* | *0.91606* | *0.91805* | *0.91899* | *0.91824* | *0.91899* | *0.91813* | *0.9207* | *0.92283* | *0.9241* | *0.9263* | *0.92876* | *0.92951* | *0.93164* | *0.93035* | *0.92939* | *0.93134* | *0.93211* | *0.92958* |
| *36768.05179* | *0.90683* | *0.91099* | *0.91306* | *0.91406* | *0.91337* | *0.9142* | *0.91338* | *0.91618* | *0.91839* | *0.91974* | *0.92204* | *0.92463* | *0.92544* | *0.92766* | *0.92644* | *0.92553* | *0.92758* | *0.92848* | *0.92587* |
| *36763.30292* | *0.90148* | *0.9058* | *0.90795* | *0.90901* | *0.9084* | *0.90931* | *0.90853* | *0.91158* | *0.91387* | *0.91529* | *0.91769* | *0.92042* | *0.92128* | *0.9236* | *0.92246* | *0.9216* | *0.92375* | *0.92478* | *0.92209* |
| *36758.55527* | *0.89603* | *0.9005* | *0.90274* | *0.90386* | *0.90332* | *0.90432* | *0.90358* | *0.90688* | *0.90926* | *0.91075* | *0.91325* | *0.91613* | *0.91704* | *0.91946* | *0.9184* | *0.9176* | *0.91986* | *0.92101* | *0.91825* |
| *36753.80885* | *0.89047* | *0.8951* | *0.89742* | *0.8986* | *0.89815* | *0.89924* | *0.89855* | *0.9021* | *0.90456* | *0.90613* | *0.90873* | *0.91176* | *0.91273* | *0.91523* | *0.91427* | *0.91353* | *0.9159* | *0.91718* | *0.91436* |
| *36749.06366* | *0.88481* | *0.88959* | *0.892* | *0.89325* | *0.89289* | *0.89406* | *0.89343* | *0.89724* | *0.89979* | *0.90143* | *0.90414* | *0.90731* | *0.90834* | *0.91094* | *0.91008* | *0.90941* | *0.91188* | *0.9133* | *0.91041* |
| *36744.31969* | *0.87905* | *0.88398* | *0.88648* | *0.8878* | *0.88753* | *0.8888* | *0.88823* | *0.8923* | *0.89493* | *0.89666* | *0.89946* | *0.90279* | *0.90388* | *0.90658* | *0.90582* | *0.90522* | *0.9078* | *0.90937* | *0.90642* |
| *36739.57694* | *0.87319* | *0.87829* | *0.88087* | *0.88226* | *0.88209* | *0.88346* | *0.88295* | *0.88729* | *0.89001* | *0.89181* | *0.89472* | *0.8982* | *0.89935* | *0.90215* | *0.9015* | *0.90099* | *0.90367* | *0.90539* | *0.90238* |
| *36734.83542* | *0.86725* | *0.8725* | *0.87518* | *0.87664* | *0.87658* | *0.87805* | *0.8776* | *0.88221* | *0.88501* | *0.8869* | *0.88991* | *0.89354* | *0.89477* | *0.89766* | *0.89713* | *0.8967* | *0.89949* | *0.90136* | *0.8983* |
| *36730.09512* | *0.86123* | *0.86663* | *0.86941* | *0.87094* | *0.87099* | *0.87256* | *0.87218* | *0.87707* | *0.87995* | *0.88192* | *0.88504* | *0.88883* | *0.89012* | *0.89311* | *0.89271* | *0.89236* | *0.89526* | *0.89728* | *0.89418* |
| *36725.35605* | *0.85514* | *0.86069* | *0.86356* | *0.86516* | *0.86533* | *0.86701* | *0.86671* | *0.87187* | *0.87483* | *0.87688* | *0.88011* | *0.88406* | *0.88542* | *0.88851* | *0.88823* | *0.88798* | *0.891* | *0.89317* | *0.89002* |
| *36720.6182* | *0.84897* | *0.85467* | *0.85764* | *0.85932* | *0.8596* | *0.8614* | *0.86117* | *0.86661* | *0.86966* | *0.87179* | *0.87513* | *0.87924* | *0.88067* | *0.88386* | *0.88372* | *0.88357* | *0.88669* | *0.88902* | *0.88584* |
| *36715.88157* | *0.84274* | *0.84858* | *0.85165* | *0.85342* | *0.85382* | *0.85573* | *0.85559* | *0.8613* | *0.86443* | *0.86665* | *0.87009* | *0.87437* | *0.87587* | *0.87916* | *0.87916* | *0.87912* | *0.88235* | *0.88484* | *0.88163* |
| *36711.14616* | *0.83644* | *0.84244* | *0.84561* | *0.84745* | *0.84799* | *0.85001* | *0.84996* | *0.85594* | *0.85916* | *0.86147* | *0.86501* | *0.86946* | *0.87104* | *0.87443* | *0.87457* | *0.87464* | *0.87798* | *0.88063* | *0.8774* |
| *36706.41197* | *0.8301* | *0.83624* | *0.83951* | *0.84144* | *0.84211* | *0.84424* | *0.84429* | *0.85055* | *0.85385* | *0.85624* | *0.85989* | *0.86451* | *0.86616* | *0.86965* | *0.86994* | *0.87013* | *0.87358* | *0.8764* | *0.87315* |
| *36701.67901* | *0.82371* | *0.82999* | *0.83336* | *0.83538* | *0.83618* | *0.83844* | *0.83858* | *0.84512* | *0.8485* | *0.85098* | *0.85474* | *0.85952* | *0.86126* | *0.86485* | *0.86529* | *0.8656* | *0.86916* | *0.87215* | *0.86888* |
| *36696.94726* | *0.81727* | *0.8237* | *0.82717* | *0.82928* | *0.83023* | *0.8326* | *0.83285* | *0.83965* | *0.84312* | *0.84568* | *0.84955* | *0.85451* | *0.85632* | *0.86002* | *0.86061* | *0.86105* | *0.86472* | *0.86787* | *0.8646* |
| *36692.21674* | *0.8108* | *0.81737* | *0.82095* | *0.82314* | *0.82424* | *0.82673* | *0.82709* | *0.83416* | *0.83771* | *0.84036* | *0.84434* | *0.84947* | *0.85137* | *0.85516* | *0.85591* | *0.85648* | *0.86027* | *0.86359* | *0.86032* |
| *36687.48743* | *0.8043* | *0.811* | *0.81469* | *0.81698* | *0.81822* | *0.82084* | *0.82131* | *0.82865* | *0.83227* | *0.83502* | *0.8391* | *0.84441* | *0.84639* | *0.85028* | *0.8512* | *0.85191* | *0.8558* | *0.85929* | *0.85603* |
| *36682.75935* | *0.79777* | *0.80461* | *0.80841* | *0.81079* | *0.81219* | *0.81494* | *0.81552* | *0.82312* | *0.82682* | *0.82965* | *0.83385* | *0.83933* | *0.8414* | *0.84539* | *0.84648* | *0.84733* | *0.85133* | *0.85499* | *0.85174* |
| *36678.03248* | *0.79123* | *0.7982* | *0.80211* | *0.80459* | *0.80614* | *0.80902* | *0.80972* | *0.81757* | *0.82136* | *0.82428* | *0.82858* | *0.83424* | *0.8364* | *0.8405* | *0.84175* | *0.84275* | *0.84686* | *0.85069* | *0.84746* |
| *36673.30683* | *0.78468* | *0.79178* | *0.7958* | *0.79837* | *0.80008* | *0.80309* | *0.80392* | *0.81202* | *0.81588* | *0.8189* | *0.82331* | *0.82915* | *0.8314* | *0.83559* | *0.83702* | *0.83817* | *0.84239* | *0.84639* | *0.84319* |
| *36668.58239* | *0.77812* | *0.78535* | *0.78948* | *0.79215* | *0.79402* | *0.79717* | *0.79812* | *0.80647* | *0.81041* | *0.81351* | *0.81803* | *0.82405* | *0.8264* | *0.83069* | *0.83229* | *0.83359* | *0.83792* | *0.84209* | *0.83893* |
| *36663.85918* | *0.77157* | *0.77892* | *0.78316* | *0.78593* | *0.78797* | *0.79125* | *0.79233* | *0.80091* | *0.80493* | *0.80813* | *0.81276* | *0.81896* | *0.8214* | *0.82579* | *0.82757* | *0.82903* | *0.83346* | *0.8378* | *0.83468* |
| *36659.13718* | *0.76502* | *0.77249* | *0.77685* | *0.77972* | *0.78193* | *0.78534* | *0.78655* | *0.79537* | *0.79947* | *0.80275* | *0.80749* | *0.81387* | *0.81641* | *0.82089* | *0.82285* | *0.82448* | *0.82902* | *0.83352* | *0.83046* |
| *36654.4164* | *0.75848* | *0.76607* | *0.77055* | *0.77353* | *0.7759* | *0.77944* | *0.7808* | *0.78984* | *0.79401* | *0.79738* | *0.80223* | *0.8088* | *0.81143* | *0.81601* | *0.81816* | *0.81996* | *0.82459* | *0.82926* | *0.82626* |
| *36649.69683* | *0.75197* | *0.75967* | *0.76427* | *0.76735* | *0.76989* | *0.77357* | *0.77507* | *0.78432* | *0.78857* | *0.79203* | *0.79699* | *0.80374* | *0.80647* | *0.81115* | *0.81348* | *0.81545* | *0.82018* | *0.82503* | *0.82209* |
| *36644.97848* | *0.74548* | *0.7533* | *0.75801* | *0.7612* | *0.76392* | *0.76773* | *0.76938* | *0.77883* | *0.78315* | *0.78671* | *0.79177* | *0.7987* | *0.80154* | *0.80631* | *0.80882* | *0.81097* | *0.8158* | *0.82081* | *0.81794* |
| *36640.26134* | *0.73903* | *0.74695* | *0.75178* | *0.75508* | *0.75797* | *0.76192* | *0.76371* | *0.77336* | *0.77775* | *0.7814* | *0.78658* | *0.79369* | *0.79663* | *0.8015* | *0.80419* | *0.80652* | *0.81145* | *0.81662* | *0.81384* |
| *36635.54542* | *0.73262* | *0.74064* | *0.74559* | *0.74899* | *0.75206* | *0.75616* | *0.7581* | *0.76793* | *0.77239* | *0.77613* | *0.78141* | *0.78871* | *0.79175* | *0.79672* | *0.7996* | *0.80211* | *0.80713* | *0.81246* | *0.80977* |
| *36630.83071* | *0.72625* | *0.73437* | *0.73944* | *0.74296* | *0.7462* | *0.75043* | *0.75253* | *0.76253* | *0.76707* | *0.7709* | *0.77629* | *0.78376* | *0.78692* | *0.79198* | *0.79504* | *0.79774* | *0.80285* | *0.80834* | *0.80574* |
| *36626.11722* | *0.71994* | *0.72815* | *0.73335* | *0.73697* | *0.74039* | *0.74476* | *0.74701* | *0.75717* | *0.76178* | *0.7657* | *0.7712* | *0.77886* | *0.78212* | *0.78727* | *0.79052* | *0.79341* | *0.79861* | *0.80426* | *0.80176* |
| *36621.40493* | *0.71368* | *0.72198* | *0.7273* | *0.73104* | *0.73464* | *0.73914* | *0.74155* | *0.75186* | *0.75654* | *0.76055* | *0.76616* | *0.774* | *0.77737* | *0.78261* | *0.78604* | *0.78913* | *0.79442* | *0.80022* | *0.79783* |
| *36616.69386* | *0.70749* | *0.71587* | *0.72132* | *0.72517* | *0.72895* | *0.73359* | *0.73616* | *0.74661* | *0.75135* | *0.75545* | *0.76116* | *0.76919* | *0.77267* | *0.778* | *0.78162* | *0.7849* | *0.79027* | *0.79622* | *0.79396* |
| *36611.984* | *0.70137* | *0.70983* | *0.71541* | *0.71936* | *0.72332* | *0.7281* | *0.73083* | *0.74141* | *0.74622* | *0.75041* | *0.75623* | *0.76443* | *0.76803* | *0.77345* | *0.77724* | *0.78072* | *0.78617* | *0.79227* | *0.79013* |
| *36607.27536* | *0.69532* | *0.70387* | *0.70956* | *0.71363* | *0.71777* | *0.72269* | *0.72558* | *0.73626* | *0.74114* | *0.74542* | *0.75135* | *0.75973* | *0.76344* | *0.76895* | *0.77293* | *0.7766* | *0.78213* | *0.78838* | *0.78637* |
| *36602.56792* | *0.68936* | *0.69798* | *0.7038* | *0.70799* | *0.7123* | *0.71735* | *0.72041* | *0.73119* | *0.73613* | *0.7405* | *0.74653* | *0.75509* | *0.75892* | *0.76451* | *0.76867* | *0.77255* | *0.77815* | *0.78454* | *0.78267* |
| *36597.8617* | *0.68349* | *0.69218* | *0.69812* | *0.70242* | *0.70691* | *0.7121* | *0.71533* | *0.72619* | *0.73119* | *0.73565* | *0.74178* | *0.75052* | *0.75447* | *0.76014* | *0.76448* | *0.76856* | *0.77423* | *0.78076* | *0.77903* |
| *36593.15668* | *0.67772* | *0.68646* | *0.69253* | *0.69695* | *0.70162* | *0.70693* | *0.71033* | *0.72126* | *0.72632* | *0.73087* | *0.73711* | *0.74602* | *0.75009* | *0.75584* | *0.76035* | *0.76464* | *0.77038* | *0.77704* | *0.77547* |
| *36588.45287* | *0.67205* | *0.68085* | *0.68704* | *0.69157* | *0.69641* | *0.70186* | *0.70543* | *0.71641* | *0.72154* | *0.72617* | *0.73251* | *0.7416* | *0.74578* | *0.75162* | *0.7563* | *0.76079* | *0.7666* | *0.77339* | *0.77197* |
| *36583.75028* | *0.66648* | *0.67533* | *0.68165* | *0.68629* | *0.69131* | *0.69689* | *0.70063* | *0.71164* | *0.71683* | *0.72156* | *0.728* | *0.73725* | *0.74156* | *0.74747* | *0.75233* | *0.75702* | *0.76289* | *0.76981* | *0.76855* |
| *36579.04889* | *0.66103* | *0.66992* | *0.67637* | *0.68112* | *0.68631* | *0.69202* | *0.69593* | *0.70697* | *0.71221* | *0.71702* | *0.72357* | *0.73299* | *0.73742* | *0.7434* | *0.74843* | *0.75332* | *0.75925* | *0.76629* | *0.76521* |
| *36574.34871* | *0.65569* | *0.66463* | *0.6712* | *0.67606* | *0.68142* | *0.68726* | *0.69134* | *0.70238* | *0.70769* | *0.71258* | *0.71923* | *0.72882* | *0.73337* | *0.73943* | *0.74461* | *0.74972* | *0.7557* | *0.76286* | *0.76194* |
| *36569.64974* | *0.65048* | *0.65945* | *0.66615* | *0.67112* | *0.67665* | *0.68261* | *0.68686* | *0.6979* | *0.70326* | *0.70824* | *0.71498* | *0.72474* | *0.72941* | *0.73554* | *0.74089* | *0.74619* | *0.75222* | *0.7595* | *0.75876* |
| *36564.95197* | *0.6454* | *0.6544* | *0.66122* | *0.66631* | *0.672* | *0.67808* | *0.6825* | *0.69351* | *0.69893* | *0.70399* | *0.71083* | *0.72075* | *0.72554* | *0.73174* | *0.73725* | *0.74276* | *0.74883* | *0.75622* | *0.75567* |
| *36560.25542* | *0.64045* | *0.64948* | *0.65642* | *0.66161* | *0.66747* | *0.67367* | *0.67825* | *0.68923* | *0.6947* | *0.69984* | *0.70678* | *0.71686* | *0.72178* | *0.72804* | *0.7337* | *0.73942* | *0.74553* | *0.75302* | *0.75266* |
| *36555.56006* | *0.63564* | *0.64469* | *0.65175* | *0.65705* | *0.66307* | *0.66938* | *0.67414* | *0.68506* | *0.69058* | *0.69581* | *0.70284* | *0.71308* | *0.71812* | *0.72445* | *0.73025* | *0.73617* | *0.74232* | *0.74991* | *0.74974* |
| *36550.86592* | *0.63098* | *0.64004* | *0.64722* | *0.65263* | *0.6588* | *0.66523* | *0.67015* | *0.681* | *0.68658* | *0.69188* | *0.69901* | *0.7094* | *0.71456* | *0.72095* | *0.7269* | *0.73302* | *0.73921* | *0.74688* | *0.74691* |
| *36546.17298* | *0.62647* | *0.63553* | *0.64283* | *0.64835* | *0.65467* | *0.66121* | *0.6663* | *0.67706* | *0.68269* | *0.68807* | *0.69529* | *0.70583* | *0.71111* | *0.71756* | *0.72365* | *0.72997* | *0.73619* | *0.74395* | *0.74418* |
| *36541.48124* | *0.62211* | *0.63118* | *0.63859* | *0.64421* | *0.65068* | *0.65733* | *0.66258* | *0.67324* | *0.67892* | *0.68437* | *0.69169* | *0.70238* | *0.70778* | *0.71429* | *0.72051* | *0.72702* | *0.73326* | *0.74111* | *0.74154* |
| *36536.79071* | *0.61791* | *0.62697* | *0.6345* | *0.64022* | *0.64683* | *0.65359* | *0.65901* | *0.66955* | *0.67527* | *0.6808* | *0.68821* | *0.69904* | *0.70456* | *0.71112* | *0.71747* | *0.72418* | *0.73044* | *0.73837* | *0.739* |
| *36532.10139* | *0.61387* | *0.62292* | *0.63056* | *0.63638* | *0.64313* | *0.64999* | *0.65557* | *0.66598* | *0.67175* | *0.67735* | *0.68485* | *0.69581* | *0.70146* | *0.70807* | *0.71455* | *0.72145* | *0.72772* | *0.73572* | *0.73655* |
| *36527.41326* | *0.61* | *0.61904* | *0.62678* | *0.6327* | *0.63959* | *0.64655* | *0.65228* | *0.66255* | *0.66836* | *0.67403* | *0.68161* | *0.69272* | *0.69849* | *0.70514* | *0.71173* | *0.71882* | *0.72511* | *0.73317* | *0.73421* |
| *36522.72634* | *0.6063* | *0.61531* | *0.62317* | *0.62918* | *0.6362* | *0.64325* | *0.64915* | *0.65925* | *0.6651* | *0.67084* | *0.67851* | *0.68974* | *0.69563* | *0.70233* | *0.70903* | *0.71631* | *0.7226* | *0.73073* | *0.73198* |
| *36518.04062* | *0.60278* | *0.61176* | *0.61972* | *0.62583* | *0.63297* | *0.64012* | *0.64616* | *0.65608* | *0.66197* | *0.66779* | *0.67554* | *0.6869* | *0.6929* | *0.69965* | *0.70645* | *0.71391* | *0.72021* | *0.72839* | *0.72984* |
| *36513.35611* | *0.59943* | *0.60838* | *0.61644* | *0.62264* | *0.6299* | *0.63713* | *0.64333* | *0.65306* | *0.65899* | *0.66487* | *0.6727* | *0.68418* | *0.69031* | *0.69709* | *0.70399* | *0.71162* | *0.71792* | *0.72615* | *0.72781* |
| *36508.6728* | *0.59627* | *0.60518* | *0.61334* | *0.61962* | *0.627* | *0.63432* | *0.64066* | *0.65018* | *0.65615* | *0.66209* | *0.67* | *0.6816* | *0.68784* | *0.69465* | *0.70165* | *0.70946* | *0.71574* | *0.72402* | *0.72589* |
| *36503.99068* | *0.59329* | *0.60216* | *0.61041* | *0.61678* | *0.62427* | *0.63166* | *0.63814* | *0.64745* | *0.65345* | *0.65945* | *0.66744* | *0.67915* | *0.6855* | *0.69235* | *0.69943* | *0.70741* | *0.71368* | *0.72199* | *0.72408* |
| *36499.30977* | *0.5905* | *0.59932* | *0.60766* | *0.61411* | *0.6217* | *0.62917* | *0.63579* | *0.64487* | *0.6509* | *0.65696* | *0.66502* | *0.67684* | *0.6833* | *0.69018* | *0.69734* | *0.70548* | *0.71174* | *0.72008* | *0.72237* |
| *36494.63006* | *0.58791* | *0.59666* | *0.6051* | *0.61162* | *0.61931* | *0.62685* | *0.6336* | *0.64244* | *0.6485* | *0.65462* | *0.66275* | *0.67467* | *0.68124* | *0.68814* | *0.69538* | *0.70367* | *0.70991* | *0.71828* | *0.72077* |
| *36489.95155* | *0.58551* | *0.59419* | *0.60271* | *0.60931* | *0.61709* | *0.62469* | *0.63158* | *0.64016* | *0.64625* | *0.65242* | *0.66062* | *0.67264* | *0.67932* | *0.68624* | *0.69354* | *0.70198* | *0.7082* | *0.71658* | *0.71928* |
| *36485.27424* | *0.5833* | *0.59192* | *0.60052* | *0.60718* | *0.61505* | *0.62271* | *0.62972* | *0.63804* | *0.64416* | *0.65038* | *0.65864* | *0.67075* | *0.67754* | *0.68447* | *0.69183* | *0.70042* | *0.7066* | *0.71501* | *0.7179* |
| *36480.59812* | *0.5813* | *0.58984* | *0.59851* | *0.60523* | *0.61319* | *0.6209* | *0.62803* | *0.63608* | *0.64222* | *0.64849* | *0.65681* | *0.669* | *0.67589* | *0.68284* | *0.69025* | *0.69898* | *0.70513* | *0.71354* | *0.71663* |
| *36475.92321* | *0.57949* | *0.58795* | *0.5967* | *0.60348* | *0.6115* | *0.61927* | *0.62651* | *0.63427* | *0.64044* | *0.64675* | *0.65514* | *0.6674* | *0.67439* | *0.68135* | *0.68881* | *0.69767* | *0.70378* | *0.71219* | *0.71548* |
| *36471.24949* | *0.57789* | *0.58626* | *0.59507* | *0.60191* | *0.61* | *0.61781* | *0.62516* | *0.63263* | *0.63881* | *0.64517* | *0.65361* | *0.66595* | *0.67304* | *0.68* | *0.6875* | *0.69648* | *0.70255* | *0.71096* | *0.71443* |
| *36466.57697* | *0.57649* | *0.58477* | *0.59364* | *0.60053* | *0.60868* | *0.61653* | *0.62398* | *0.63115* | *0.63735* | *0.64374* | *0.65224* | *0.66465* | *0.67183* | *0.6788* | *0.68632* | *0.69542* | *0.70143* | *0.70984* | *0.7135* |
| *36461.90565* | *0.5753* | *0.58347* | *0.59241* | *0.59934* | *0.60754* | *0.61543* | *0.62298* | *0.62984* | *0.63605* | *0.64248* | *0.65103* | *0.66349* | *0.67076* | *0.67773* | *0.68527* | *0.69448* | *0.70045* | *0.70883* | *0.71267* |
| *36457.23552* | *0.57432* | *0.58238* | *0.59137* | *0.59834* | *0.60659* | *0.61451* | *0.62214* | *0.62869* | *0.63491* | *0.64137* | *0.64997* | *0.66248* | *0.66984* | *0.6768* | *0.68436* | *0.69367* | *0.69958* | *0.70795* | *0.71196* |
| *36452.56659* | *0.57354* | *0.5815* | *0.59053* | *0.59753* | *0.60582* | *0.61376* | *0.62148* | *0.62771* | *0.63394* | *0.64043* | *0.64907* | *0.66162* | *0.66907* | *0.67602* | *0.68358* | *0.69299* | *0.69883* | *0.70718* | *0.71135* |
| *36447.89886* | *0.57298* | *0.58081* | *0.58988* | *0.59691* | *0.60523* | *0.6132* | *0.621* | *0.6269* | *0.63313* | *0.63964* | *0.64832* | *0.66092* | *0.66844* | *0.67538* | *0.68293* | *0.69243* | *0.69821* | *0.70652* | *0.71086* |
| *36443.23232* | *0.57262* | *0.58033* | *0.58944* | *0.59649* | *0.60483* | *0.61281* | *0.62068* | *0.62625* | *0.63249* | *0.63902* | *0.64773* | *0.66036* | *0.66795* | *0.67488* | *0.68243* | *0.692* | *0.69771* | *0.70598* | *0.71048* |
| *36438.56698* | *0.57247* | *0.58005* | *0.58919* | *0.59625* | *0.60462* | *0.6126* | *0.62053* | *0.62577* | *0.63201* | *0.63856* | *0.6473* | *0.65995* | *0.66762* | *0.67452* | *0.68205* | *0.6917* | *0.69733* | *0.70556* | *0.7102* |
| *36433.90283* | *0.57253* | *0.57998* | *0.58914* | *0.59621* | *0.60459* | *0.61257* | *0.62056* | *0.62547* | *0.6317* | *0.63826* | *0.64703* | *0.6597* | *0.66743* | *0.6743* | *0.68181* | *0.69152* | *0.69707* | *0.70525* | *0.71003* |
| *36429.23987* | *0.5728* | *0.58011* | *0.58928* | *0.59636* | *0.60474* | *0.61272* | *0.62076* | *0.62533* | *0.63155* | *0.63812* | *0.64692* | *0.65959* | *0.66738* | *0.67423* | *0.6817* | *0.69146* | *0.69693* | *0.70506* | *0.70997* |
| *36424.57811* | *0.57328* | *0.58044* | *0.58962* | *0.5967* | *0.60507* | *0.61304* | *0.62112* | *0.62536* | *0.63157* | *0.63815* | *0.64696* | *0.65964* | *0.66748* | *0.67429* | *0.68173* | *0.69153* | *0.69691* | *0.70499* | *0.71001* |
| *36419.91754* | *0.57396* | *0.58098* | *0.59016* | *0.59723* | *0.60559* | *0.61354* | *0.62166* | *0.62556* | *0.63176* | *0.63833* | *0.64716* | *0.65983* | *0.66773* | *0.6745* | *0.68189* | *0.69172* | *0.69701* | *0.70502* | *0.71016* |
| *36415.25817* | *0.57486* | *0.58171* | *0.59089* | *0.59795* | *0.60628* | *0.61422* | *0.62236* | *0.62592* | *0.63211* | *0.63868* | *0.64751* | *0.66017* | *0.66811* | *0.67484* | *0.68217* | *0.69203* | *0.69722* | *0.70517* | *0.71042* |
| *36410.59998* | *0.57595* | *0.58265* | *0.59182* | *0.59885* | *0.60716* | *0.61507* | *0.62323* | *0.62646* | *0.63263* | *0.63918* | *0.64802* | *0.66065* | *0.66864* | *0.67532* | *0.68259* | *0.69246* | *0.69756* | *0.70544* | *0.71077* |
| *36405.94299* | *0.57725* | *0.58378* | *0.59294* | *0.59994* | *0.60821* | *0.61609* | *0.62426* | *0.62716* | *0.6333* | *0.63984* | *0.64868* | *0.66129* | *0.66931* | *0.67594* | *0.68314* | *0.69301* | *0.698* | *0.70581* | *0.71122* |
| *36401.28719* | *0.57876* | *0.58512* | *0.59424* | *0.60122* | *0.60944* | *0.61728* | *0.62545* | *0.62803* | *0.63415* | *0.64067* | *0.6495* | *0.66206* | *0.67011* | *0.67669* | *0.68381* | *0.69368* | *0.69857* | *0.70629* | *0.71177* |
| *36396.63258* | *0.58046* | *0.58665* | *0.59574* | *0.60267* | *0.61085* | *0.61863* | *0.6268* | *0.62906* | *0.63515* | *0.64164* | *0.65046* | *0.66298* | *0.67106* | *0.67757* | *0.68461* | *0.69446* | *0.69924* | *0.70688* | *0.71242* |
| *36391.97916* | *0.58236* | *0.58837* | *0.59742* | *0.6043* | *0.61242* | *0.62016* | *0.62831* | *0.63026* | *0.63631* | *0.64277* | *0.65158* | *0.66405* | *0.67213* | *0.67859* | *0.68554* | *0.69536* | *0.70002* | *0.70757* | *0.71317* |
| *36387.32693* | *0.58445* | *0.59028* | *0.59928* | *0.60611* | *0.61416* | *0.62184* | *0.62997* | *0.63162* | *0.63763* | *0.64406* | *0.65284* | *0.66525* | *0.67335* | *0.67973* | *0.68658* | *0.69637* | *0.70092* | *0.70837* | *0.714* |
| *36382.67588* | *0.58674* | *0.59238* | *0.60133* | *0.60809* | *0.61607* | *0.62369* | *0.63178* | *0.63314* | *0.6391* | *0.64549* | *0.65425* | *0.66658* | *0.67469* | *0.681* | *0.68775* | *0.69748* | *0.70191* | *0.70928* | *0.71493* |
| *36378.02603* | *0.58921* | *0.59466* | *0.60355* | *0.61024* | *0.61814* | *0.62569* | *0.63375* | *0.63482* | *0.64073* | *0.64708* | *0.6558* | *0.66806* | *0.67616* | *0.6824* | *0.68903* | *0.69871* | *0.70302* | *0.71028* | *0.71595* |
| *36373.37737* | *0.59187* | *0.59713* | *0.60594* | *0.61256* | *0.62037* | *0.62785* | *0.63585* | *0.63665* | *0.64251* | *0.64881* | *0.65749* | *0.66966* | *0.67776* | *0.68391* | *0.69043* | *0.70004* | *0.70422* | *0.71138* | *0.71705* |
| *36368.72989* | *0.59472* | *0.59977* | *0.60851* | *0.61504* | *0.62276* | *0.63016* | *0.6381* | *0.63864* | *0.64444* | *0.65068* | *0.65932* | *0.6714* | *0.67948* | *0.68555* | *0.69194* | *0.70147* | *0.70553* | *0.71258* | *0.71823* |
| *36364.0836* | *0.59774* | *0.6026* | *0.61124* | *0.61769* | *0.6253* | *0.63261* | *0.64049* | *0.64077* | *0.64652* | *0.6527* | *0.66129* | *0.67326* | *0.68132* | *0.6873* | *0.69357* | *0.703* | *0.70693* | *0.71388* | *0.7195* |
| *36359.4385* | *0.60094* | *0.60559* | *0.61414* | *0.62049* | *0.62799* | *0.63521* | *0.64302* | *0.64306* | *0.64874* | *0.65485* | *0.66338* | *0.67524* | *0.68328* | *0.68917* | *0.6953* | *0.70463* | *0.70843* | *0.71526* | *0.72085* |
| *36354.79458* | *0.6043* | *0.60875* | *0.6172* | *0.62344* | *0.63083* | *0.63795* | *0.64568* | *0.64549* | *0.6511* | *0.65714* | *0.66561* | *0.67735* | *0.68536* | *0.69115* | *0.69713* | *0.70636* | *0.71002* | *0.71674* | *0.72227* |
| *36350.15185* | *0.60783* | *0.61207* | *0.62041* | *0.62654* | *0.63381* | *0.64083* | *0.64846* | *0.64806* | *0.6536* | *0.65956* | *0.66796* | *0.67958* | *0.68754* | *0.69324* | *0.69907* | *0.70818* | *0.7117* | *0.7183* | *0.72377* |
| *36345.51031* | *0.61153* | *0.61555* | *0.62378* | *0.62979* | *0.63693* | *0.64384* | *0.65137* | *0.65078* | *0.65623* | *0.66211* | *0.67044* | *0.68192* | *0.68984* | *0.69543* | *0.70111* | *0.71008* | *0.71347* | *0.71995* | *0.72534* |
| *36340.86995* | *0.61538* | *0.61919* | *0.62729* | *0.63317* | *0.64017* | *0.64698* | *0.6544* | *0.65362* | *0.65899* | *0.66478* | *0.67303* | *0.68437* | *0.69223* | *0.69772* | *0.70324* | *0.71207* | *0.71532* | *0.72167* | *0.72697* |
| *36336.23078* | *0.61938* | *0.62297* | *0.63094* | *0.63669* | *0.64355* | *0.65024* | *0.65754* | *0.6566* | *0.66188* | *0.66758* | *0.67574* | *0.68693* | *0.69473* | *0.70011* | *0.70546* | *0.71414* | *0.71725* | *0.72348* | *0.72868* |
| *36331.59279* | *0.62353* | *0.6269* | *0.63473* | *0.64034* | *0.64705* | *0.65362* | *0.66079* | *0.65971* | *0.6649* | *0.67049* | *0.67856* | *0.68959* | *0.69733* | *0.70259* | *0.70778* | *0.7163* | *0.71926* | *0.72536* | *0.73044* |
| *36326.95598* | *0.62781* | *0.63097* | *0.63865* | *0.64411* | *0.65067* | *0.65711* | *0.66415* | *0.66294* | *0.66803* | *0.67351* | *0.68149* | *0.69236* | *0.70002* | *0.70516* | *0.71017* | *0.71852* | *0.72135* | *0.72732* | *0.73226* |
| *36322.32036* | *0.63224* | *0.63517* | *0.6427* | *0.64801* | *0.65441* | *0.66071* | *0.66761* | *0.66628* | *0.67128* | *0.67665* | *0.68452* | *0.69522* | *0.7028* | *0.70782* | *0.71265* | *0.72082* | *0.7235* | *0.72934* | *0.73414* |
| *36317.68592* | *0.63679* | *0.6395* | *0.64687* | *0.65201* | *0.65825* | *0.66442* | *0.67116* | *0.66975* | *0.67463* | *0.67989* | *0.68766* | *0.69817* | *0.70566* | *0.71056* | *0.71521* | *0.72319* | *0.72573* | *0.73143* | *0.73607* |
| *36313.05266* | *0.64146* | *0.64395* | *0.65115* | *0.65613* | *0.6622* | *0.66822* | *0.67481* | *0.67332* | *0.6781* | *0.68323* | *0.69088* | *0.70121* | *0.70861* | *0.71338* | *0.71784* | *0.72562* | *0.72801* | *0.73358* | *0.73805* |
| *36308.42059* | *0.64625* | *0.64852* | *0.65554* | *0.66035* | *0.66624* | *0.67212* | *0.67854* | *0.677* | *0.68166* | *0.68666* | *0.69419* | *0.70433* | *0.71163* | *0.71627* | *0.72054* | *0.72812* | *0.73036* | *0.73579* | *0.74008* |
| *36303.78969* | *0.65115* | *0.65319* | *0.66004* | *0.66467* | *0.67038* | *0.67611* | *0.68235* | *0.68078* | *0.68532* | *0.69019* | *0.69759* | *0.70753* | *0.71472* | *0.71923* | *0.72331* | *0.73067* | *0.73276* | *0.73806* | *0.74215* |
| *36299.15998* | *0.65615* | *0.65797* | *0.66463* | *0.66907* | *0.6746* | *0.68018* | *0.68624* | *0.68465* | *0.68907* | *0.6938* | *0.70107* | *0.7108* | *0.71788* | *0.72226* | *0.72613* | *0.73327* | *0.73522* | *0.74038* | *0.74426* |
| *36294.53145* | *0.66126* | *0.66285* | *0.66931* | *0.67357* | *0.67891* | *0.68433* | *0.6902* | *0.68862* | *0.69291* | *0.69749* | *0.70463* | *0.71414* | *0.72111* | *0.72534* | *0.72902* | *0.73592* | *0.73772* | *0.74275* | *0.7464* |
| *36289.9041* | *0.66645* | *0.66782* | *0.67408* | *0.67814* | *0.68329* | *0.68854* | *0.69422* | *0.69267* | *0.69682* | *0.70126* | *0.70825* | *0.71754* | *0.72439* | *0.72848* | *0.73195* | *0.73862* | *0.74028* | *0.74516* | *0.74858* |
| *36285.27793* | *0.67172* | *0.67287* | *0.67892* | *0.68278* | *0.68774* | *0.69283* | *0.6983* | *0.6968* | *0.70081* | *0.7051* | *0.71194* | *0.72101* | *0.72772* | *0.73167* | *0.73494* | *0.74136* | *0.74287* | *0.74761* | *0.75079* |
| *36280.65294* | *0.67707* | *0.678* | *0.68384* | *0.68749* | *0.69225* | *0.69717* | *0.70244* | *0.701* | *0.70488* | *0.709* | *0.71569* | *0.72453* | *0.7311* | *0.73491* | *0.73797* | *0.74413* | *0.7455* | *0.7501* | *0.75302* |
| *36276.02912* | *0.68249* | *0.6832* | *0.68882* | *0.69226* | *0.69681* | *0.70157* | *0.70662* | *0.70527* | *0.709* | *0.71296* | *0.71949* | *0.72809* | *0.73453* | *0.73819* | *0.74104* | *0.74694* | *0.74816* | *0.75262* | *0.75527* |
| *36271.40649* | *0.68797* | *0.68846* | *0.69386* | *0.69709* | *0.70143* | *0.70601* | *0.71084* | *0.7096* | *0.71318* | *0.71697* | *0.72334* | *0.7317* | *0.73799* | *0.7415* | *0.74414* | *0.74977* | *0.75085* | *0.75517* | *0.75754* |
| *36266.78503* | *0.6935* | *0.69378* | *0.69895* | *0.70196* | *0.70609* | *0.7105* | *0.7151* | *0.71399* | *0.71742* | *0.72104* | *0.72723* | *0.73535* | *0.74148* | *0.74485* | *0.74727* | *0.75263* | *0.75357* | *0.75775* | *0.75983* |
| *36262.16475* | *0.69908* | *0.69915* | *0.70408* | *0.70687* | *0.71079* | *0.71502* | *0.71939* | *0.71843* | *0.7217* | *0.72514* | *0.73116* | *0.73903* | *0.74501* | *0.74822* | *0.75043* | *0.75551* | *0.75631* | *0.76035* | *0.76213* |
| *36257.54565* | *0.7047* | *0.70455* | *0.70925* | *0.71181* | *0.71552* | *0.71957* | *0.7237* | *0.72291* | *0.72602* | *0.72928* | *0.73513* | *0.74274* | *0.74855* | *0.75161* | *0.75361* | *0.75841* | *0.75906* | *0.76296* | *0.76443* |
| *36252.92772* | *0.71035* | *0.70999* | *0.71445* | *0.71678* | *0.72028* | *0.72414* | *0.72802* | *0.72742* | *0.73037* | *0.73345* | *0.73911* | *0.74647* | *0.75212* | *0.75502* | *0.7568* | *0.76131* | *0.76183* | *0.76559* | *0.76674* |
| *36248.31097* | *0.71602* | *0.71546* | *0.71967* | *0.72176* | *0.72505* | *0.72872* | *0.73236* | *0.73197* | *0.73476* | *0.73764* | *0.74312* | *0.75021* | *0.75569* | *0.75844* | *0.76* | *0.76422* | *0.7646* | *0.76823* | *0.76905* |
| *36243.6954* | *0.72171* | *0.72095* | *0.72491* | *0.72676* | *0.72983* | *0.73332* | *0.73671* | *0.73654* | *0.73916* | *0.74185* | *0.74714* | *0.75397* | *0.75927* | *0.76187* | *0.76321* | *0.76714* | *0.76738* | *0.77087* | *0.77135* |
| *36239.081* | *0.7274* | *0.72644* | *0.73015* | *0.73177* | *0.73462* | *0.73792* | *0.74105* | *0.74113* | *0.74357* | *0.74607* | *0.75116* | *0.75773* | *0.76285* | *0.76529* | *0.76642* | *0.77005* | *0.77016* | *0.77351* | *0.77365* |
| *36234.46778* | *0.7331* | *0.73194* | *0.7354* | *0.73677* | *0.7394* | *0.74252* | *0.74538* | *0.74573* | *0.748* | *0.7503* | *0.75519* | *0.76149* | *0.76643* | *0.76871* | *0.76963* | *0.77295* | *0.77293* | *0.77615* | *0.77593* |
| *36229.85573* | *0.73878* | *0.73743* | *0.74063* | *0.74177* | *0.74418* | *0.7471* | *0.74971* | *0.75033* | *0.75243* | *0.75452* | *0.75922* | *0.76525* | *0.77* | *0.77212* | *0.77283* | *0.77584* | *0.7757* | *0.77878* | *0.77821* |
| *36225.24485* | *0.74445* | *0.74292* | *0.74586* | *0.74675* | *0.74894* | *0.75167* | *0.75401* | *0.75493* | *0.75685* | *0.75874* | *0.76323* | *0.76899* | *0.77355* | *0.77552* | *0.77601* | *0.77872* | *0.77845* | *0.78139* | *0.78046* |
| *36220.63515* | *0.75009* | *0.74838* | *0.75106* | *0.7517* | *0.75368* | *0.75622* | *0.75829* | *0.75952* | *0.76126* | *0.76295* | *0.76723* | *0.77271* | *0.77708* | *0.7789* | *0.77917* | *0.78158* | *0.78118* | *0.784* | *0.78269* |
| *36216.02662* | *0.7557* | *0.75381* | *0.75623* | *0.75663* | *0.75838* | *0.76074* | *0.76253* | *0.76409* | *0.76565* | *0.76713* | *0.7712* | *0.77641* | *0.78059* | *0.78225* | *0.78231* | *0.78441* | *0.78389* | *0.78658* | *0.78489* |
| *36211.41927* | *0.76127* | *0.75921* | *0.76137* | *0.76152* | *0.76306* | *0.76522* | *0.76674* | *0.76864* | *0.77001* | *0.77129* | *0.77515* | *0.78008* | *0.78406* | *0.78557* | *0.78542* | *0.78721* | *0.78658* | *0.78913* | *0.78707* |
| *36206.81308* | *0.76679* | *0.76456* | *0.76646* | *0.76636* | *0.76769* | *0.76966* | *0.77091* | *0.77316* | *0.77435* | *0.77541* | *0.77906* | *0.78372* | *0.7875* | *0.78885* | *0.7885* | *0.78998* | *0.78923* | *0.79166* | *0.78921* |
| *36202.20807* | *0.77226* | *0.76987* | *0.7715* | *0.77116* | *0.77227* | *0.77405* | *0.77502* | *0.77764* | *0.77864* | *0.77949* | *0.78293* | *0.78731* | *0.79089* | *0.79209* | *0.79154* | *0.79271* | *0.79185* | *0.79415* | *0.79132* |
| *36197.60423* | *0.77766* | *0.77511* | *0.77649* | *0.77589* | *0.7768* | *0.77839* | *0.77908* | *0.78208* | *0.78289* | *0.78353* | *0.78675* | *0.79086* | *0.79424* | *0.79529* | *0.79453* | *0.79539* | *0.79443* | *0.79661* | *0.79339* |
| *36193.00156* | *0.78299* | *0.78029* | *0.7814* | *0.78057* | *0.78126* | *0.78266* | *0.78308* | *0.78646* | *0.78709* | *0.78752* | *0.79052* | *0.79436* | *0.79754* | *0.79843* | *0.79748* | *0.79803* | *0.79697* | *0.79902* | *0.79541* |
| *36188.40006* | *0.78823* | *0.7854* | *0.78625* | *0.78517* | *0.78566* | *0.78687* | *0.78701* | *0.79079* | *0.79123* | *0.79144* | *0.79423* | *0.7978* | *0.80077* | *0.80152* | *0.80037* | *0.80062* | *0.79946* | *0.80139* | *0.79739* |
| *36183.79973* | *0.79339* | *0.79042* | *0.79102* | *0.78969* | *0.78998* | *0.79101* | *0.79086* | *0.79505* | *0.79531* | *0.79531* | *0.79787* | *0.80117* | *0.80395* | *0.80455* | *0.80321* | *0.80316* | *0.80189* | *0.80371* | *0.79932* |
| *36179.20057* | *0.79846* | *0.79535* | *0.7957* | *0.79413* | *0.79421* | *0.79506* | *0.79464* | *0.79924* | *0.79931* | *0.7991* | *0.80145* | *0.80448* | *0.80705* | *0.80751* | *0.80598* | *0.80563* | *0.80428* | *0.80597* | *0.80119* |
| *36174.60258* | *0.80342* | *0.80019* | *0.80028* | *0.79848* | *0.79837* | *0.79903* | *0.79834* | *0.80335* | *0.80324* | *0.80281* | *0.80494* | *0.80771* | *0.81009* | *0.8104* | *0.80869* | *0.80804* | *0.8066* | *0.80818* | *0.80301* |
| *36170.00575* | *0.80827* | *0.80493* | *0.80477* | *0.80273* | *0.80242* | *0.80291* | *0.80194* | *0.80738* | *0.80708* | *0.80644* | *0.80836* | *0.81087* | *0.81304* | *0.81322* | *0.81132* | *0.81039* | *0.80886* | *0.81033* | *0.80477* |
| *36165.4101* | *0.813* | *0.80955* | *0.80915* | *0.80687* | *0.80638* | *0.80669* | *0.80545* | *0.81131* | *0.81083* | *0.80999* | *0.81168* | *0.81394* | *0.81591* | *0.81595* | *0.81388* | *0.81266* | *0.81105* | *0.81241* | *0.80646* |
| *36160.81561* | *0.81761* | *0.81406* | *0.81341* | *0.81091* | *0.81024* | *0.81037* | *0.80886* | *0.81515* | *0.81449* | *0.81343* | *0.81492* | *0.81692* | *0.81869* | *0.8186* | *0.81636* | *0.81485* | *0.81318* | *0.81442* | *0.80809* |
| *36156.22229* | *0.82209* | *0.81844* | *0.81756* | *0.81483* | *0.81398* | *0.81394* | *0.81216* | *0.81888* | *0.81804* | *0.81678* | *0.81805* | *0.8198* | *0.82138* | *0.82116* | *0.81875* | *0.81697* | *0.81522* | *0.81636* | *0.80965* |
| *36151.63014* | *0.82643* | *0.8227* | *0.82158* | *0.81863* | *0.8176* | *0.8174* | *0.81535* | *0.82249* | *0.82148* | *0.82002* | *0.82108* | *0.82259* | *0.82398* | *0.82362* | *0.82105* | *0.81901* | *0.81719* | *0.81823* | *0.81114* |
| *36147.03915* | *0.83062* | *0.82681* | *0.82546* | *0.8223* | *0.8211* | *0.82074* | *0.81842* | *0.826* | *0.82481* | *0.82315* | *0.824* | *0.82527* | *0.82647* | *0.82599* | *0.82326* | *0.82095* | *0.81908* | *0.82001* | *0.81255* |
| *36142.44933* | *0.83466* | *0.83078* | *0.82921* | *0.82583* | *0.82448* | *0.82395* | *0.82138* | *0.82937* | *0.82802* | *0.82616* | *0.8268* | *0.82784* | *0.82885* | *0.82825* | *0.82538* | *0.82281* | *0.82088* | *0.82171* | *0.81389* |
| *36137.86067* | *0.83854* | *0.8346* | *0.83281* | *0.82923* | *0.82772* | *0.82703* | *0.8242* | *0.83262* | *0.8311* | *0.82904* | *0.82948* | *0.8303* | *0.83113* | *0.83041* | *0.82739* | *0.82457* | *0.82259* | *0.82333* | *0.81515* |
| *36133.27318* | *0.84226* | *0.83827* | *0.83627* | *0.83248* | *0.83082* | *0.82998* | *0.8269* | *0.83573* | *0.83405* | *0.8318* | *0.83204* | *0.83264* | *0.83329* | *0.83246* | *0.82929* | *0.82624* | *0.8242* | *0.82485* | *0.81632* |
| *36128.68686* | *0.84581* | *0.84177* | *0.83956* | *0.83559* | *0.83377* | *0.83279* | *0.82946* | *0.8387* | *0.83686* | *0.83443* | *0.83447* | *0.83486* | *0.83533* | *0.83439* | *0.83109* | *0.8278* | *0.82573* | *0.82628* | *0.8174* |
| *36124.10169* | *0.84918* | *0.8451* | *0.8427* | *0.83853* | *0.83658* | *0.83545* | *0.83189* | *0.84153* | *0.83953* | *0.83691* | *0.83677* | *0.83694* | *0.83724* | *0.8362* | *0.83277* | *0.82926* | *0.82715* | *0.82761* | *0.8184* |
| *36119.51769* | *0.85237* | *0.84826* | *0.84567* | *0.84132* | *0.83924* | *0.83797* | *0.83417* | *0.8442* | *0.84205* | *0.83926* | *0.83892* | *0.8389* | *0.83903* | *0.83789* | *0.83434* | *0.83062* | *0.82847* | *0.82885* | *0.81931* |
| *36114.93486* | *0.85537* | *0.85125* | *0.84847* | *0.84395* | *0.84174* | *0.84033* | *0.8363* | *0.84671* | *0.84442* | *0.84145* | *0.84093* | *0.84072* | *0.84069* | *0.83945* | *0.83579* | *0.83186* | *0.82969* | *0.82998* | *0.82013* |
| *36110.35319* | *0.85818* | *0.85404* | *0.85109* | *0.8464* | *0.84407* | *0.84253* | *0.83828* | *0.84906* | *0.84663* | *0.8435* | *0.8428* | *0.84241* | *0.84222* | *0.84089* | *0.83711* | *0.83299* | *0.8308* | *0.831* | *0.82085* |
| *36105.77268* | *0.86079* | *0.85665* | *0.85353* | *0.84868* | *0.84624* | *0.84458* | *0.84011* | *0.85124* | *0.84868* | *0.84538* | *0.84451* | *0.84395* | *0.84361* | *0.84219* | *0.83831* | *0.834* | *0.83179* | *0.83192* | *0.82147* |
| *36101.19333* | *0.8632* | *0.85907* | *0.85579* | *0.85078* | *0.84824* | *0.84646* | *0.84178* | *0.85325* | *0.85056* | *0.84711* | *0.84607* | *0.84534* | *0.84486* | *0.84336* | *0.83938* | *0.8349* | *0.83268* | *0.83273* | *0.82199* |
| *36096.61514* | *0.8654* | *0.86128* | *0.85786* | *0.8527* | *0.85007* | *0.84817* | *0.84328* | *0.85509* | *0.85227* | *0.84867* | *0.84747* | *0.84658* | *0.84596* | *0.84439* | *0.84032* | *0.83568* | *0.83345* | *0.83342* | *0.82241* |
| *36092.03812* | *0.86739* | *0.8633* | *0.85973* | *0.85444* | *0.85172* | *0.8497* | *0.84463* | *0.85674* | *0.8538* | *0.85006* | *0.8487* | *0.84767* | *0.84692* | *0.84527* | *0.84112* | *0.83633* | *0.8341* | *0.834* | *0.82273* |
| *36087.46225* | *0.86916* | *0.8651* | *0.8614* | *0.85599* | *0.85318* | *0.85107* | *0.8458* | *0.85821* | *0.85516* | *0.85128* | *0.84978* | *0.84861* | *0.84773* | *0.84602* | *0.84179* | *0.83685* | *0.83463* | *0.83446* | *0.82294* |
| *36082.88755* | *0.87072* | *0.8667* | *0.86288* | *0.85734* | *0.85447* | *0.85225* | *0.8468* | *0.85949* | *0.85633* | *0.85233* | *0.85068* | *0.84938* | *0.84838* | *0.84661* | *0.84231* | *0.83725* | *0.83503* | *0.83479* | *0.82305* |
| *36078.314* | *0.87205* | *0.86808* | *0.86415* | *0.8585* | *0.85556* | *0.85325* | *0.84763* | *0.86057* | *0.85732* | *0.85319* | *0.85141* | *0.84999* | *0.84888* | *0.84706* | *0.8427* | *0.83752* | *0.83531* | *0.83501* | *0.82304* |
| *36073.74162* | *0.87316* | *0.86925* | *0.86521* | *0.85946* | *0.85646* | *0.85407* | *0.84828* | *0.86146* | *0.85812* | *0.85388* | *0.85196* | *0.85044* | *0.84923* | *0.84736* | *0.84294* | *0.83766* | *0.83547* | *0.8351* | *0.82293* |
| *36069.17039* | *0.87404* | *0.87019* | *0.86607* | *0.86022* | *0.85718* | *0.8547* | *0.84876* | *0.86216* | *0.85873* | *0.85438* | *0.85234* | *0.85073* | *0.84941* | *0.8475* | *0.84303* | *0.83766* | *0.83549* | *0.83507* | *0.82271* |
| *36064.60032* | *0.87469* | *0.87092* | *0.86671* | *0.86078* | *0.85769* | *0.85514* | *0.84905* | *0.86265* | *0.85914* | *0.85469* | *0.85254* | *0.85084* | *0.84944* | *0.84749* | *0.84297* | *0.83753* | *0.83539* | *0.83491* | *0.82237* |
| *36060.03141* | *0.8751* | *0.87141* | *0.86713* | *0.86113* | *0.85801* | *0.8554* | *0.84916* | *0.86293* | *0.85936* | *0.85482* | *0.85256* | *0.85078* | *0.8493* | *0.84732* | *0.84277* | *0.83726* | *0.83515* | *0.83461* | *0.82192* |
| *36055.46366* | *0.87528* | *0.87168* | *0.86734* | *0.86128* | *0.85813* | *0.85545* | *0.84909* | *0.86301* | *0.85938* | *0.85475* | *0.85239* | *0.85055* | *0.849* | *0.84699* | *0.84241* | *0.83685* | *0.83478* | *0.83419* | *0.82135* |
| *36050.89706* | *0.87522* | *0.87172* | *0.86733* | *0.86121* | *0.85805* | *0.85532* | *0.84883* | *0.86288* | *0.85919* | *0.85449* | *0.85204* | *0.85015* | *0.84853* | *0.8465* | *0.8419* | *0.8363* | *0.83428* | *0.83364* | *0.82067* |
| *36046.33162* | *0.87491* | *0.87153* | *0.8671* | *0.86093* | *0.85777* | *0.85498* | *0.84839* | *0.86253* | *0.8588* | *0.85403* | *0.85151* | *0.84957* | *0.8479* | *0.84585* | *0.84124* | *0.83561* | *0.83364* | *0.83295* | *0.81987* |
| *36041.76734* | *0.87437* | *0.8711* | *0.86665* | *0.86044* | *0.85728* | *0.85445* | *0.84775* | *0.86198* | *0.85821* | *0.85338* | *0.85078* | *0.84882* | *0.84709* | *0.84504* | *0.84042* | *0.83478* | *0.83286* | *0.83213* | *0.81896* |
| *36037.20421* | *0.87359* | *0.87044* | *0.86597* | *0.85974* | *0.85659* | *0.85372* | *0.84693* | *0.86121* | *0.85741* | *0.85253* | *0.84987* | *0.84788* | *0.84612* | *0.84407* | *0.83945* | *0.83381* | *0.83195* | *0.83117* | *0.81792* |
| *36032.64224* | *0.87256* | *0.86955* | *0.86507* | *0.85882* | *0.85569* | *0.85279* | *0.84592* | *0.86022* | *0.85641* | *0.85149* | *0.84876* | *0.84677* | *0.84498* | *0.84293* | *0.83832* | *0.8327* | *0.83089* | *0.83007* | *0.81677* |
| *36028.08142* | *0.87129* | *0.86842* | *0.86394* | *0.85769* | *0.85459* | *0.85166* | *0.84472* | *0.85902* | *0.85519* | *0.85024* | *0.84747* | *0.84548* | *0.84367* | *0.84163* | *0.83703* | *0.83144* | *0.8297* | *0.82884* | *0.81549* |
| *36023.52176* | *0.86978* | *0.86705* | *0.86259* | *0.85635* | *0.85327* | *0.85033* | *0.84332* | *0.8576* | *0.85377* | *0.84879* | *0.84598* | *0.84401* | *0.84219* | *0.84016* | *0.83559* | *0.83003* | *0.82837* | *0.82747* | *0.8141* |
| *36018.96325* | *0.86802* | *0.86545* | *0.86102* | *0.85478* | *0.85175* | *0.8488* | *0.84174* | *0.85597* | *0.85214* | *0.84715* | *0.8443* | *0.84236* | *0.84054* | *0.83853* | *0.83398* | *0.82849* | *0.8269* | *0.82597* | *0.81259* |
| *36014.40589* | *0.86602* | *0.86361* | *0.85922* | *0.85301* | *0.85003* | *0.84707* | *0.83996* | *0.85412* | *0.8503* | *0.8453* | *0.84244* | *0.84054* | *0.83871* | *0.83674* | *0.83222* | *0.82679* | *0.82528* | *0.82432* | *0.81095* |
| *36009.84969* | *0.86378* | *0.86153* | *0.85719* | *0.85101* | *0.84809* | *0.84514* | *0.83799* | *0.85205* | *0.84825* | *0.84325* | *0.84038* | *0.83853* | *0.83672* | *0.83478* | *0.8303* | *0.82496* | *0.82353* | *0.82254* | *0.8092* |
| *36005.29464* | *0.8613* | *0.85922* | *0.85494* | *0.84881* | *0.84595* | *0.84301* | *0.83584* | *0.84976* | *0.84599* | *0.84101* | *0.83813* | *0.83634* | *0.83456* | *0.83265* | *0.82822* | *0.82297* | *0.82163* | *0.82062* | *0.80732* |
| *36000.74074* | *0.85857* | *0.85667* | *0.85246* | *0.84638* | *0.84361* | *0.84068* | *0.83349* | *0.84726* | *0.84353* | *0.83856* | *0.83569* | *0.83398* | *0.83222* | *0.83036* | *0.82599* | *0.82085* | *0.8196* | *0.81856* | *0.80533* |
| *35996.188* | *0.85561* | *0.85389* | *0.84977* | *0.84375* | *0.84106* | *0.83815* | *0.83095* | *0.84454* | *0.84086* | *0.83592* | *0.83306* | *0.83143* | *0.82972* | *0.82791* | *0.8236* | *0.81858* | *0.81743* | *0.81636* | *0.80321* |
| *35991.6364* | *0.85241* | *0.85088* | *0.84685* | *0.84091* | *0.8383* | *0.83542* | *0.82823* | *0.84161* | *0.83798* | *0.83308* | *0.83024* | *0.82871* | *0.82705* | *0.8253* | *0.82105* | *0.81616* | *0.81511* | *0.81403* | *0.80098* |
| *35987.08596* | *0.84897* | *0.84764* | *0.84371* | *0.83785* | *0.83534* | *0.83249* | *0.82532* | *0.83847* | *0.8349* | *0.83005* | *0.82724* | *0.82582* | *0.82422* | *0.82252* | *0.81834* | *0.81361* | *0.81266* | *0.81156* | *0.79863* |
| *35982.53666* | *0.8453* | *0.84417* | *0.84035* | *0.83459* | *0.83218* | *0.82937* | *0.82222* | *0.83511* | *0.83161* | *0.82682* | *0.82405* | *0.82275* | *0.82121* | *0.81959* | *0.81549* | *0.81091* | *0.81007* | *0.80895* | *0.79615* |
| *35977.98852* | *0.8414* | *0.84048* | *0.83678* | *0.83111* | *0.82882* | *0.82605* | *0.81894* | *0.83155* | *0.82813* | *0.82339* | *0.82068* | *0.8195* | *0.81805* | *0.81649* | *0.81248* | *0.80807* | *0.80734* | *0.80621* | *0.79356* |
| *35973.44153* | *0.83727* | *0.83656* | *0.833* | *0.82744* | *0.82526* | *0.82255* | *0.81548* | *0.82778* | *0.82444* | *0.81978* | *0.81713* | *0.81609* | *0.81472* | *0.81324* | *0.80931* | *0.80509* | *0.80447* | *0.80333* | *0.79085* |
| *35968.89568* | *0.83292* | *0.83242* | *0.829* | *0.82356* | *0.82151* | *0.81885* | *0.81183* | *0.82381* | *0.82056* | *0.81598* | *0.81339* | *0.81251* | *0.81123* | *0.80983* | *0.806* | *0.80197* | *0.80147* | *0.80032* | *0.78803* |
| *35964.35099* | *0.82834* | *0.82806* | *0.82479* | *0.81948* | *0.81756* | *0.81496* | *0.80801* | *0.81963* | *0.81649* | *0.81199* | *0.80948* | *0.80876* | *0.80758* | *0.80627* | *0.80254* | *0.79872* | *0.79833* | *0.79717* | *0.78509* |
| *35959.80744* | *0.82354* | *0.82349* | *0.82038* | *0.81521* | *0.81342* | *0.81089* | *0.80401* | *0.81526* | *0.81222* | *0.80782* | *0.8054* | *0.80484* | *0.80377* | *0.80255* | *0.79893* | *0.79533* | *0.79506* | *0.7939* | *0.78203* |
| *35955.26504* | *0.81853* | *0.8187* | *0.81577* | *0.81073* | *0.8091* | *0.80664* | *0.79983* | *0.81069* | *0.80777* | *0.80347* | *0.80114* | *0.80076* | *0.79981* | *0.79868* | *0.79517* | *0.7918* | *0.79166* | *0.79049* | *0.77886* |
| *35950.72379* | *0.81331* | *0.81371* | *0.81095* | *0.80607* | *0.80458* | *0.8022* | *0.79549* | *0.80593* | *0.80313* | *0.79894* | *0.79671* | *0.79652* | *0.79569* | *0.79467* | *0.79128* | *0.78814* | *0.78813* | *0.78696* | *0.77558* |
| *35946.18369* | *0.80788* | *0.80851* | *0.80594* | *0.80122* | *0.79989* | *0.79759* | *0.79097* | *0.80098* | *0.79831* | *0.79424* | *0.79211* | *0.79213* | *0.79143* | *0.79051* | *0.78724* | *0.78435* | *0.78447* | *0.7833* | *0.77218* |
| *35941.64473* | *0.80225* | *0.80311* | *0.80074* | *0.79619* | *0.79501* | *0.7928* | *0.78629* | *0.79584* | *0.79331* | *0.78937* | *0.78735* | *0.78758* | *0.78701* | *0.78621* | *0.78306* | *0.78043* | *0.78068* | *0.77952* | *0.76868* |
| *35937.10692* | *0.79641* | *0.79752* | *0.79534* | *0.79097* | *0.78996* | *0.78784* | *0.78144* | *0.79053* | *0.78813* | *0.78432* | *0.78243* | *0.78287* | *0.78245* | *0.78176* | *0.77874* | *0.77638* | *0.77677* | *0.77561* | *0.76507* |
| *35932.57025* | *0.79039* | *0.79173* | *0.78977* | *0.78557* | *0.78474* | *0.78271* | *0.77644* | *0.78504* | *0.78278* | *0.77912* | *0.77735* | *0.77802* | *0.77775* | *0.77718* | *0.77429* | *0.77221* | *0.77273* | *0.77158* | *0.76135* |
| *35928.03473* | *0.78417* | *0.78575* | *0.78401* | *0.78* | *0.77934* | *0.77742* | *0.77127* | *0.77937* | *0.77727* | *0.77375* | *0.77212* | *0.77302* | *0.77291* | *0.77246* | *0.76971* | *0.76791* | *0.76857* | *0.76743* | *0.75752* |
| *35923.50035* | *0.77777* | *0.77959* | *0.77807* | *0.77426* | *0.77378* | *0.77196* | *0.76596* | *0.77354* | *0.77159* | *0.76822* | *0.76674* | *0.76788* | *0.76794* | *0.7676* | *0.765* | *0.7635* | *0.7643* | *0.76317* | *0.7536* |
| *35918.96712* | *0.77119* | *0.77326* | *0.77197* | *0.76836* | *0.76806* | *0.76635* | *0.76049* | *0.76754* | *0.76576* | *0.76254* | *0.76121* | *0.7626* | *0.76283* | *0.76262* | *0.76017* | *0.75896* | *0.7599* | *0.75879* | *0.74956* |
| *35914.43503* | *0.76444* | *0.76675* | *0.76569* | *0.76229* | *0.76217* | *0.76058* | *0.75487* | *0.76138* | *0.75977* | *0.75672* | *0.75554* | *0.75719* | *0.75759* | *0.75751* | *0.75521* | *0.75431* | *0.75539* | *0.7543* | *0.74543* |
| *35909.90409* | *0.75751* | *0.76007* | *0.75926* | *0.75606* | *0.75614* | *0.75466* | *0.74911* | *0.75507* | *0.75363* | *0.75075* | *0.74973* | *0.75164* | *0.75223* | *0.75228* | *0.75013* | *0.74955* | *0.75077* | *0.7497* | *0.7412* |
| *35905.37429* | *0.75043* | *0.75322* | *0.75266* | *0.74969* | *0.74995* | *0.7486* | *0.74322* | *0.74861* | *0.74735* | *0.74464* | *0.74379* | *0.74597* | *0.74674* | *0.74693* | *0.74494* | *0.74467* | *0.74604* | *0.74498* | *0.73688* |
| *35900.84563* | *0.74318* | *0.74622* | *0.74591* | *0.74316* | *0.74362* | *0.74239* | *0.73718* | *0.74201* | *0.74092* | *0.73839* | *0.73771* | *0.74017* | *0.74113* | *0.74146* | *0.73963* | *0.73969* | *0.7412* | *0.74017* | *0.73245* |
| *35896.31811* | *0.73579* | *0.73907* | *0.73901* | *0.73649* | *0.73715* | *0.73604* | *0.73101* | *0.73527* | *0.73437* | *0.73201* | *0.73152* | *0.73425* | *0.73541* | *0.73588* | *0.73421* | *0.7346* | *0.73626* | *0.73525* | *0.72794* |
| *35891.79174* | *0.72824* | *0.73177* | *0.73197* | *0.72968* | *0.73054* | *0.72956* | *0.72472* | *0.72839* | *0.72768* | *0.72551* | *0.7252* | *0.72822* | *0.72957* | *0.73019* | *0.72868* | *0.72941* | *0.73121* | *0.73023* | *0.72333* |
| *35887.2665* | *0.72056* | *0.72432* | *0.7248* | *0.72274* | *0.7238* | *0.72296* | *0.7183* | *0.72139* | *0.72086* | *0.71889* | *0.71876* | *0.72207* | *0.72363* | *0.72439* | *0.72305* | *0.72412* | *0.72607* | *0.72512* | *0.71864* |
| *35882.74241* | *0.71274* | *0.71675* | *0.71749* | *0.71567* | *0.71694* | *0.71622* | *0.71176* | *0.71426* | *0.71393* | *0.71215* | *0.71221* | *0.71582* | *0.71758* | *0.71849* | *0.71732* | *0.71873* | *0.72083* | *0.71991* | *0.71386* |
| *35878.21946* | *0.7048* | *0.70904* | *0.71005* | *0.70847* | *0.70995* | *0.70937* | *0.70511* | *0.70702* | *0.70688* | *0.70529* | *0.70555* | *0.70946* | *0.71144* | *0.71249* | *0.71149* | *0.71325* | *0.7155* | *0.71461* | *0.709* |
| *35873.69765* | *0.69673* | *0.70121* | *0.70249* | *0.70116* | *0.70284* | *0.7024* | *0.69835* | *0.69967* | *0.69973* | *0.69834* | *0.6988* | *0.703* | *0.70519* | *0.70639* | *0.70557* | *0.70767* | *0.71007* | *0.70922* | *0.70405* |
| *35869.17697* | *0.68855* | *0.69326* | *0.69482* | *0.69373* | *0.69562* | *0.69532* | *0.69148* | *0.69221* | *0.69247* | *0.69128* | *0.69194* | *0.69645* | *0.69886* | *0.70021* | *0.69955* | *0.70202* | *0.70456* | *0.70374* | *0.69903* |
| *35864.65744* | *0.68026* | *0.6852* | *0.68703* | *0.6862* | *0.68829* | *0.68814* | *0.68451* | *0.68465* | *0.68511* | *0.68412* | *0.68499* | *0.68981* | *0.69244* | *0.69394* | *0.69346* | *0.69627* | *0.69896* | *0.69819* | *0.69392* |
| *35860.13905* | *0.67186* | *0.67703* | *0.67914* | *0.67856* | *0.68086* | *0.68085* | *0.67744* | *0.677* | *0.67766* | *0.67688* | *0.67795* | *0.68309* | *0.68593* | *0.68758* | *0.68728* | *0.69045* | *0.69329* | *0.69255* | *0.68875* |
| *35855.62179* | *0.66337* | *0.66876* | *0.67116* | *0.67082* | *0.67333* | *0.67347* | *0.67027* | *0.66927* | *0.67013* | *0.66955* | *0.67083* | *0.67628* | *0.67934* | *0.68114* | *0.68102* | *0.68455* | *0.68753* | *0.68684* | *0.6835* |
| *35851.10567* | *0.65479* | *0.6604* | *0.66307* | *0.66299* | *0.66571* | *0.66599* | *0.66302* | *0.66145* | *0.66251* | *0.66214* | *0.66363* | *0.6694* | *0.67268* | *0.67463* | *0.67469* | *0.67857* | *0.6817* | *0.68105* | *0.67818* |
| *35846.59069* | *0.64612* | *0.65195* | *0.65491* | *0.65508* | *0.65801* | *0.65843* | *0.65569* | *0.65355* | *0.65481* | *0.65465* | *0.65636* | *0.66244* | *0.66595* | *0.66805* | *0.66829* | *0.67252* | *0.6758* | *0.67519* | *0.6728* |
| *35842.07685* | *0.63738* | *0.64342* | *0.64666* | *0.64708* | *0.65022* | *0.65079* | *0.64827* | *0.64559* | *0.64705* | *0.6471* | *0.64902* | *0.65542* | *0.65915* | *0.6614* | *0.66182* | *0.66641* | *0.66983* | *0.66927* | *0.66735* |
| *35837.56414* | *0.62857* | *0.63482* | *0.63833* | *0.63901* | *0.64235* | *0.64307* | *0.64078* | *0.63756* | *0.63922* | *0.63948* | *0.64161* | *0.64833* | *0.65228* | *0.65469* | *0.65529* | *0.66023* | *0.66379* | *0.66329* | *0.66184* |
| *35833.05257* | *0.61969* | *0.62615* | *0.62993* | *0.63087* | *0.63441* | *0.63528* | *0.63322* | *0.62948* | *0.63134* | *0.6318* | *0.63415* | *0.64119* | *0.64536* | *0.64792* | *0.6487* | *0.65399* | *0.65769* | *0.65724* | *0.65626* |
| *35828.54214* | *0.61075* | *0.61741* | *0.62147* | *0.62266* | *0.62641* | *0.62743* | *0.6256* | *0.62134* | *0.6234* | *0.62407* | *0.62664* | *0.63399* | *0.63839* | *0.6411* | *0.64205* | *0.64769* | *0.65154* | *0.65114* | *0.65064* |
| *35824.03284* | *0.60176* | *0.60862* | *0.61296* | *0.6144* | *0.61834* | *0.61951* | *0.61791* | *0.61316* | *0.61541* | *0.61629* | *0.61907* | *0.62675* | *0.63136* | *0.63422* | *0.63535* | *0.64134* | *0.64532* | *0.64498* | *0.64495* |
| *35819.52468* | *0.59273* | *0.59977* | *0.60439* | *0.60608* | *0.61022* | *0.61153* | *0.61017* | *0.60493* | *0.60739* | *0.60847* | *0.61147* | *0.61946* | *0.62429* | *0.6273* | *0.62861* | *0.63494* | *0.63906* | *0.63878* | *0.63922* |
| *35815.01765* | *0.58365* | *0.59089* | *0.59577* | *0.59771* | *0.60205* | *0.60351* | *0.60238* | *0.59668* | *0.59932* | *0.60061* | *0.60382* | *0.61213* | *0.61718* | *0.62034* | *0.62182* | *0.62849* | *0.63275* | *0.63253* | *0.63344* |
| *35810.51175* | *0.57455* | *0.58196* | *0.58711* | *0.58929* | *0.59383* | *0.59543* | *0.59455* | *0.58839* | *0.59123* | *0.59271* | *0.59614* | *0.60476* | *0.61003* | *0.61333* | *0.61499* | *0.622* | *0.62639* | *0.62623* | *0.62761* |
| *35806.00699* | *0.56541* | *0.573* | *0.57841* | *0.58085* | *0.58557* | *0.58732* | *0.58667* | *0.58008* | *0.58311* | *0.58479* | *0.58844* | *0.59737* | *0.60285* | *0.6063* | *0.60813* | *0.61547* | *0.61999* | *0.6199* | *0.62174* |
| *35801.50336* | *0.55626* | *0.56402* | *0.56969* | *0.57236* | *0.57727* | *0.57917* | *0.57875* | *0.57176* | *0.57497* | *0.57685* | *0.58071* | *0.58995* | *0.59564* | *0.59923* | *0.60124* | *0.6089* | *0.61355* | *0.61352* | *0.61583* |
| *35797.00086* | *0.54709* | *0.55501* | *0.56094* | *0.56385* | *0.56895* | *0.57098* | *0.5708* | *0.56342* | *0.56681* | *0.56889* | *0.57296* | *0.5825* | *0.58841* | *0.59213* | *0.59432* | *0.6023* | *0.60708* | *0.60712* | *0.60988* |
| *35792.4995* | *0.53791* | *0.54599* | *0.55217* | *0.55532* | *0.5606* | *0.56277* | *0.56282* | *0.55508* | *0.55865* | *0.56092* | *0.56519* | *0.57504* | *0.58115* | *0.58502* | *0.58737* | *0.59567* | *0.60057* | *0.60068* | *0.60389* |
| *35787.99927* | *0.52873* | *0.53697* | *0.54339* | *0.54678* | *0.55222* | *0.55454* | *0.55482* | *0.54674* | *0.55048* | *0.55294* | *0.55742* | *0.56757* | *0.57388* | *0.57788* | *0.58041* | *0.58901* | *0.59404* | *0.59422* | *0.59788* |
| *35783.50017* | *0.51955* | *0.52793* | *0.5346* | *0.53822* | *0.54384* | *0.54629* | *0.5468* | *0.5384* | *0.54231* | *0.54496* | *0.54964* | *0.56009* | *0.5666* | *0.57073* | *0.57342* | *0.58233* | *0.58748* | *0.58773* | *0.59183* |
| *35779.0022* | *0.51038* | *0.51891* | *0.52581* | *0.52965* | *0.53544* | *0.53803* | *0.53877* | *0.53007* | *0.53415* | *0.53698* | *0.54186* | *0.5526* | *0.55931* | *0.56357* | *0.56643* | *0.57563* | *0.58089* | *0.58123* | *0.58575* |
| *35774.50536* | *0.50122* | *0.50988* | *0.51702* | *0.52108* | *0.52704* | *0.52976* | *0.53073* | *0.52176* | *0.526* | *0.529* | *0.53409* | *0.54512* | *0.55202* | *0.55641* | *0.55943* | *0.56892* | *0.57429* | *0.5747* | *0.57965* |
| *35770.00965* | *0.49209* | *0.50088* | *0.50824* | *0.51252* | *0.51863* | *0.52149* | *0.52268* | *0.51346* | *0.51786* | *0.52104* | *0.52632* | *0.53763* | *0.54472* | *0.54924* | *0.55242* | *0.56219* | *0.56768* | *0.56817* | *0.57353* |
| *35765.51507* | *0.48298* | *0.49189* | *0.49947* | *0.50396* | *0.51023* | *0.51322* | *0.51463* | *0.5052* | *0.50975* | *0.5131* | *0.51857* | *0.53016* | *0.53743* | *0.54207* | *0.54541* | *0.55545* | *0.56105* | *0.56162* | *0.56739* |
| *35761.02162* | *0.47389* | *0.48292* | *0.49072* | *0.49542* | *0.50184* | *0.50495* | *0.50658* | *0.49696* | *0.50166* | *0.50517* | *0.51083* | *0.5227* | *0.53015* | *0.5349* | *0.5384* | *0.54871* | *0.55441* | *0.55506* | *0.56123* |
| *35756.5293* | *0.46485* | *0.47398* | *0.48199* | *0.48689* | *0.49346* | *0.49669* | *0.49854* | *0.48875* | *0.49359* | *0.49727* | *0.50311* | *0.51525* | *0.52288* | *0.52775* | *0.5314* | *0.54196* | *0.54777* | *0.5485* | *0.55506* |
| *35752.03811* | *0.45584* | *0.46508* | *0.47329* | *0.47839* | *0.48509* | *0.48845* | *0.49051* | *0.48059* | *0.48557* | *0.4894* | *0.49542* | *0.50783* | *0.51562* | *0.5206* | *0.5244* | *0.53521* | *0.54112* | *0.54194* | *0.54888* |
| *35747.54804* | *0.44688* | *0.45621* | *0.46462* | *0.46991* | *0.47675* | *0.48023* | *0.48249* | *0.47246* | *0.47758* | *0.48157* | *0.48776* | *0.50042* | *0.50838* | *0.51347* | *0.51742* | *0.52847* | *0.53448* | *0.53538* | *0.54269* |
| *35743.05911* | *0.43796* | *0.44739* | *0.45598* | *0.46146* | *0.46844* | *0.47203* | *0.4745* | *0.46439* | *0.46963* | *0.47377* | *0.48013* | *0.49305* | *0.50116* | *0.50636* | *0.51045* | *0.52173* | *0.52783* | *0.52883* | *0.53649* |
| *35738.5713* | *0.4291* | *0.43861* | *0.44739* | *0.45305* | *0.46015* | *0.46385* | *0.46652* | *0.45637* | *0.46173* | *0.46601* | *0.47254* | *0.4857* | *0.49397* | *0.49927* | *0.50351* | *0.515* | *0.5212* | *0.52228* | *0.53029* |
| *35734.08462* | *0.4203* | *0.42989* | *0.43884* | *0.44468* | *0.4519* | *0.45571* | *0.45858* | *0.4484* | *0.45388* | *0.45829* | *0.46499* | *0.47839* | *0.4868* | *0.4922* | *0.49658* | *0.50828* | *0.51457* | *0.51575* | *0.52408* |
| *35729.59906* | *0.41157* | *0.42122* | *0.43034* | *0.43635* | *0.44368* | *0.4476* | *0.45066* | *0.44049* | *0.44608* | *0.45063* | *0.45748* | *0.47111* | *0.47967* | *0.48517* | *0.48968* | *0.50158* | *0.50795* | *0.50922* | *0.51788* |
| *35725.11463* | *0.4029* | *0.41262* | *0.4219* | *0.42807* | *0.43551* | *0.43953* | *0.44278* | *0.43264* | *0.43834* | *0.44301* | *0.45002* | *0.46388* | *0.47257* | *0.47816* | *0.48281* | *0.49489* | *0.50135* | *0.50272* | *0.51168* |
| *35720.63133* | *0.3943* | *0.40407* | *0.41351* | *0.41984* | *0.42738* | *0.4315* | *0.43493* | *0.42486* | *0.43066* | *0.43545* | *0.44261* | *0.45669* | *0.46552* | *0.47119* | *0.47596* | *0.48823* | *0.49476* | *0.49623* | *0.50549* |
| *35716.14915* | *0.38577* | *0.3956* | *0.40518* | *0.41166* | *0.4193* | *0.42352* | *0.42713* | *0.41715* | *0.42304* | *0.42795* | *0.43525* | *0.44955* | *0.4585* | *0.46425* | *0.46915* | *0.48159* | *0.4882* | *0.48976* | *0.4993* |
| *35711.66809* | *0.37732* | *0.3872* | *0.39692* | *0.40355* | *0.41127* | *0.41558* | *0.41937* | *0.40951* | *0.41549* | *0.42051* | *0.42795* | *0.44245* | *0.45152* | *0.45735* | *0.46238* | *0.47497* | *0.48166* | *0.48332* | *0.49313* |
| *35707.18816* | *0.36896* | *0.37887* | *0.38872* | *0.39549* | *0.4033* | *0.4077* | *0.41165* | *0.40195* | *0.40801* | *0.41314* | *0.4207* | *0.43541* | *0.44459* | *0.4505* | *0.45565* | *0.46839* | *0.47514* | *0.4769* | *0.48697* |
| *35702.70936* | *0.36068* | *0.37063* | *0.3806* | *0.3875* | *0.39539* | *0.39987* | *0.40399* | *0.39447* | *0.40061* | *0.40583* | *0.41353* | *0.42843* | *0.43771* | *0.44369* | *0.44895* | *0.46183* | *0.46865* | *0.47051* | *0.48082* |
| *35698.23167* | *0.35249* | *0.36246* | *0.37255* | *0.37958* | *0.38754* | *0.39211* | *0.39638* | *0.38707* | *0.39328* | *0.3986* | *0.40641* | *0.4215* | *0.43088* | *0.43693* | *0.44231* | *0.45531* | *0.4622* | *0.46416* | *0.47469* |
| *35693.75511* | *0.34438* | *0.35438* | *0.36458* | *0.37172* | *0.37976* | *0.3844* | *0.38883* | *0.37975* | *0.38603* | *0.39143* | *0.39936* | *0.41463* | *0.42411* | *0.43022* | *0.4357* | *0.44883* | *0.45577* | *0.45784* | *0.46858* |
| *35689.27968* | *0.33638* | *0.34639* | *0.35668* | *0.36395* | *0.37205* | *0.37676* | *0.38134* | *0.37252* | *0.37886* | *0.38435* | *0.39239* | *0.40783* | *0.41739* | *0.42356* | *0.42915* | *0.44238* | *0.44938* | *0.45155* | *0.46249* |
| *35684.80536* | *0.32847* | *0.33849* | *0.34888* | *0.35625* | *0.3644* | *0.36919* | *0.37391* | *0.36538* | *0.37177* | *0.37733* | *0.38548* | *0.40109* | *0.41074* | *0.41696* | *0.42265* | *0.43597* | *0.44303* | *0.4453* | *0.45643* |
| *35680.33217* | *0.32066* | *0.33068* | *0.34116* | *0.34862* | *0.35683* | *0.36168* | *0.36654* | *0.35833* | *0.36477* | *0.3704* | *0.37865* | *0.39442* | *0.40414* | *0.41041* | *0.4162* | *0.42961* | *0.43671* | *0.43909* | *0.45039* |
| *35675.8601* | *0.31295* | *0.32297* | *0.33352* | *0.34108* | *0.34934* | *0.35425* | *0.35924* | *0.35137* | *0.35785* | *0.36355* | *0.3719* | *0.38782* | *0.3976* | *0.40393* | *0.40981* | *0.42329* | *0.43044* | *0.43293* | *0.44437* |
| *35671.38915* | *0.30535* | *0.31536* | *0.32598* | *0.33363* | *0.34193* | *0.3469* | *0.35201* | *0.34451* | *0.35103* | *0.35679* | *0.36522* | *0.38128* | *0.39113* | *0.3975* | *0.40348* | *0.41702* | *0.42421* | *0.42681* | *0.43839* |
| *35666.91932* | *0.29785* | *0.30785* | *0.31853* | *0.32626* | *0.33459* | *0.33962* | *0.34486* | *0.33774* | *0.34429* | *0.35011* | *0.35863* | *0.37483* | *0.38473* | *0.39114* | *0.3972* | *0.41079* | *0.41803* | *0.42073* | *0.43244* |
| *35662.4506* | *0.29046* | *0.30044* | *0.31118* | *0.31899* | *0.32734* | *0.33242* | *0.33777* | *0.33107* | *0.33765* | *0.34352* | *0.35211* | *0.36844* | *0.3784* | *0.38484* | *0.39099* | *0.40462* | *0.41189* | *0.41471* | *0.42652* |
| *35657.98301* | *0.28318* | *0.29314* | *0.30393* | *0.3118* | *0.32018* | *0.3253* | *0.33076* | *0.3245* | *0.33111* | *0.33702* | *0.34568* | *0.36214* | *0.37214* | *0.37861* | *0.38483* | *0.3985* | *0.40581* | *0.40873* | *0.42063* |
| *35653.51654* | *0.27601* | *0.28594* | *0.29677* | *0.3047* | *0.3131* | *0.31827* | *0.32384* | *0.31804* | *0.32465* | *0.3306* | *0.33934* | *0.35591* | *0.36594* | *0.37245* | *0.37875* | *0.39243* | *0.39977* | *0.4028* | *0.41478* |
| *35649.05119* | *0.26896* | *0.27886* | *0.28972* | *0.2977* | *0.30612* | *0.31133* | *0.31699* | *0.31167* | *0.3183* | *0.32428* | *0.33308* | *0.34976* | *0.35983* | *0.36635* | *0.37273* | *0.38642* | *0.39379* | *0.39693* | *0.40897* |
| *35644.58695* | *0.26202* | *0.27188* | *0.28277* | *0.2908* | *0.29922* | *0.30447* | *0.31022* | *0.30541* | *0.31204* | *0.31805* | *0.32691* | *0.34369* | *0.35378* | *0.36033* | *0.36677* | *0.38047* | *0.38786* | *0.39111* | *0.4032* |
| *35640.12384* | *0.25519* | *0.26501* | *0.27592* | *0.284* | *0.29242* | *0.2977* | *0.30354* | *0.29925* | *0.30588* | *0.31192* | *0.32083* | *0.3377* | *0.34782* | *0.35438* | *0.36089* | *0.37457* | *0.38199* | *0.38535* | *0.39747* |
| *35635.66184* | *0.24849* | *0.25826* | *0.26918* | *0.27729* | *0.28571* | *0.29102* | *0.29694* | *0.29319* | *0.29983* | *0.30588* | *0.31484* | *0.3318* | *0.34193* | *0.3485* | *0.35507* | *0.36874* | *0.37617* | *0.37965* | *0.39179* |
| *35631.20096* | *0.2419* | *0.25161* | *0.26254* | *0.27069* | *0.2791* | *0.28443* | *0.29042* | *0.28724* | *0.29387* | *0.29993* | *0.30893* | *0.32598* | *0.33612* | *0.3427* | *0.34933* | *0.36297* | *0.37042* | *0.37401* | *0.38614* |
| *35626.74119* | *0.23542* | *0.24509* | *0.25602* | *0.26419* | *0.27258* | *0.27794* | *0.284* | *0.28139* | *0.28801* | *0.29408* | *0.30313* | *0.32024* | *0.33038* | *0.33698* | *0.34366* | *0.35726* | *0.36472* | *0.36842* | *0.38055* |
| *35622.28254* | *0.22907* | *0.23868* | *0.2496* | *0.25779* | *0.26617* | *0.27154* | *0.27766* | *0.27565* | *0.28225* | *0.28833* | *0.29741* | *0.3146* | *0.32473* | *0.33133* | *0.33806* | *0.35161* | *0.35908* | *0.3629* | *0.375* |
| *35617.82501* | *0.22284* | *0.23238* | *0.24329* | *0.25149* | *0.25985* | *0.26523* | *0.27142* | *0.27002* | *0.2766* | *0.28268* | *0.29178* | *0.30903* | *0.31916* | *0.32576* | *0.33254* | *0.34603* | *0.35351* | *0.35744* | *0.3695* |
| *35613.36859* | *0.21673* | *0.2262* | *0.23709* | *0.2453* | *0.25363* | *0.25903* | *0.26527* | *0.26449* | *0.27105* | *0.27712* | *0.28625* | *0.30356* | *0.31367* | *0.32027* | *0.32709* | *0.34052* | *0.348* | *0.35204* | *0.36404* |
| *35608.91329* | *0.21073* | *0.22014* | *0.231* | *0.23921* | *0.24752* | *0.25292* | *0.2592* | *0.25906* | *0.2656* | *0.27167* | *0.28081* | *0.29817* | *0.30827* | *0.31485* | *0.32172* | *0.33507* | *0.34256* | *0.34671* | *0.35864* |
| *35604.4591* | *0.20486* | *0.21419* | *0.22503* | *0.23323* | *0.2415* | *0.24691* | *0.25324* | *0.25375* | *0.26025* | *0.2663* | *0.27547* | *0.29287* | *0.30294* | *0.30952* | *0.31642* | *0.32969* | *0.33718* | *0.34144* | *0.35329* |
| *35600.00603* | *0.19911* | *0.20837* | *0.21916* | *0.22736* | *0.23559* | *0.241* | *0.24736* | *0.24854* | *0.255* | *0.26104* | *0.27022* | *0.28766* | *0.2977* | *0.30427* | *0.31121* | *0.32438* | *0.33186* | *0.33624* | *0.34799* |
| *35595.55407* | *0.19348* | *0.20266* | *0.21341* | *0.2216* | *0.22978* | *0.23519* | *0.24158* | *0.24343* | *0.24986* | *0.25588* | *0.26506* | *0.28253* | *0.29255* | *0.2991* | *0.30607* | *0.31914* | *0.32662* | *0.33111* | *0.34275* |
| *35591.10322* | *0.18797* | *0.19706* | *0.20777* | *0.21594* | *0.22407* | *0.22948* | *0.2359* | *0.23843* | *0.24482* | *0.25081* | *0.26* | *0.2775* | *0.28748* | *0.29401* | *0.301* | *0.31397* | *0.32144* | *0.32604* | *0.33756* |
| *35586.65348* | *0.18258* | *0.19159* | *0.20224* | *0.21039* | *0.21847* | *0.22387* | *0.23031* | *0.23353* | *0.23987* | *0.24585* | *0.25503* | *0.27255* | *0.28249* | *0.289* | *0.29602* | *0.30887* | *0.31633* | *0.32104* | *0.33242* |
| *35582.20486* | *0.1773* | *0.18623* | *0.19683* | *0.20494* | *0.21297* | *0.21836* | *0.22481* | *0.22873* | *0.23503* | *0.24097* | *0.25016* | *0.26769* | *0.27759* | *0.28407* | *0.29112* | *0.30384* | *0.31129* | *0.31611* | *0.32734* |
| *35577.75735* | *0.17215* | *0.18099* | *0.19152* | *0.19961* | *0.20758* | *0.21295* | *0.21942* | *0.22404* | *0.23029* | *0.2362* | *0.24538* | *0.26292* | *0.27277* | *0.27922* | *0.28629* | *0.29889* | *0.30632* | *0.31125* | *0.32232* |
| *35573.31096* | *0.16712* | *0.17586* | *0.18633* | *0.19438* | *0.20229* | *0.20764* | *0.21412* | *0.21945* | *0.22565* | *0.23152* | *0.24069* | *0.25824* | *0.26804* | *0.27446* | *0.28154* | *0.29401* | *0.30142* | *0.30646* | *0.31736* |
| *35568.86567* | *0.1622* | *0.17085* | *0.18125* | *0.18926* | *0.1971* | *0.20244* | *0.20892* | *0.21496* | *0.22111* | *0.22694* | *0.2361* | *0.25365* | *0.26339* | *0.26978* | *0.27688* | *0.2892* | *0.29659* | *0.30174* | *0.31245* |
| *35564.42149* | *0.1574* | *0.16595* | *0.17628* | *0.18424* | *0.19202* | *0.19733* | *0.20381* | *0.21057* | *0.21666* | *0.22246* | *0.2316* | *0.24915* | *0.25882* | *0.26518* | *0.27229* | *0.28446* | *0.29183* | *0.29709* | *0.30761* |
| *35559.97843* | *0.15272* | *0.16117* | *0.17142* | *0.17933* | *0.18704* | *0.19233* | *0.1988* | *0.20629* | *0.21232* | *0.21807* | *0.22719* | *0.24473* | *0.25434* | *0.26066* | *0.26778* | *0.2798* | *0.28715* | *0.29251* | *0.30282* |
| *35555.53647* | *0.14815* | *0.15651* | *0.16667* | *0.17453* | *0.18216* | *0.18743* | *0.19389* | *0.20209* | *0.20807* | *0.21377* | *0.22287* | *0.2404* | *0.24994* | *0.25623* | *0.26335* | *0.27521* | *0.28253* | *0.288* | *0.2981* |
| *35551.09563* | *0.14369* | *0.15195* | *0.16203* | *0.16984* | *0.17739* | *0.18263* | *0.18907* | *0.198* | *0.20391* | *0.20957* | *0.21864* | *0.23615* | *0.24563* | *0.25188* | *0.259* | *0.27069* | *0.27799* | *0.28356* | *0.29343* |
| *35546.65589* | *0.13935* | *0.14751* | *0.1575* | *0.16524* | *0.17272* | *0.17793* | *0.18435* | *0.194* | *0.19985* | *0.20546* | *0.21451* | *0.23199* | *0.2414* | *0.2476* | *0.25473* | *0.26625* | *0.27351* | *0.27919* | *0.28883* |
| *35542.21726* | *0.13512* | *0.14318* | *0.15308* | *0.16076* | *0.16815* | *0.17333* | *0.17973* | *0.1901* | *0.19588* | *0.20144* | *0.21046* | *0.22792* | *0.23725* | *0.24341* | *0.25053* | *0.26188* | *0.26911* | *0.27489* | *0.28429* |
| *35537.77974* | *0.131* | *0.13895* | *0.14876* | *0.15638* | *0.16368* | *0.16882* | *0.1752* | *0.18629* | *0.192* | *0.19751* | *0.2065* | *0.22393* | *0.23318* | *0.2393* | *0.24642* | *0.25758* | *0.26478* | *0.27067* | *0.27981* |
| *35533.34333* | *0.12699* | *0.13484* | *0.14455* | *0.15209* | *0.15932* | *0.16442* | *0.17077* | *0.18257* | *0.18822* | *0.19367* | *0.20262* | *0.22003* | *0.2292* | *0.23527* | *0.24238* | *0.25336* | *0.26053* | *0.26651* | *0.27539* |
| *35528.90803* | *0.12309* | *0.13083* | *0.14044* | *0.14792* | *0.15505* | *0.16012* | *0.16643* | *0.17894* | *0.18452* | *0.18991* | *0.19883* | *0.2162* | *0.22529* | *0.23132* | *0.23842* | *0.24921* | *0.25634* | *0.26243* | *0.27103* |
| *35524.47383* | *0.1193* | *0.12693* | *0.13644* | *0.14384* | *0.15089* | *0.15591* | *0.16218* | *0.1754* | *0.18092* | *0.18625* | *0.19513* | *0.21246* | *0.22147* | *0.22744* | *0.23453* | *0.24513* | *0.25223* | *0.25841* | *0.26674* |
| *35520.04074* | *0.11561* | *0.12314* | *0.13254* | *0.13986* | *0.14682* | *0.1518* | *0.15803* | *0.17195* | *0.1774* | *0.18267* | *0.19151* | *0.2088* | *0.21773* | *0.22365* | *0.23072* | *0.24113* | *0.24819* | *0.25447* | *0.26251* |
| *35515.60876* | *0.11202* | *0.11944* | *0.12874* | *0.13598* | *0.14285* | *0.14779* | *0.15398* | *0.16858* | *0.17396* | *0.17918* | *0.18798* | *0.20522* | *0.21406* | *0.21993* | *0.22699* | *0.2372* | *0.24422* | *0.25059* | *0.25834* |
| *35511.17788* | *0.10854* | *0.11585* | *0.12504* | *0.1322* | *0.13897* | *0.14387* | *0.15001* | *0.1653* | *0.17061* | *0.17576* | *0.18453* | *0.20172* | *0.21047* | *0.21629* | *0.22333* | *0.23334* | *0.24032* | *0.24678* | *0.25424* |
| *35506.74811* | *0.10515* | *0.11236* | *0.12144* | *0.12852* | *0.13519* | *0.14004* | *0.14614* | *0.16211* | *0.16734* | *0.17244* | *0.18115* | *0.1983* | *0.20696* | *0.21272* | *0.21975* | *0.22955* | *0.23649* | *0.24305* | *0.2502* |
| *35502.31944* | *0.10187* | *0.10897* | *0.11793* | *0.12493* | *0.13151* | *0.13631* | *0.14235* | *0.15899* | *0.16416* | *0.16919* | *0.17786* | *0.19496* | *0.20352* | *0.20923* | *0.21624* | *0.22584* | *0.23273* | *0.23938* | *0.24622* |
| *35497.89188* | *0.09868* | *0.10568* | *0.11453* | *0.12144* | *0.12792* | *0.13267* | *0.13866* | *0.15595* | *0.16105* | *0.16602* | *0.17465* | *0.19169* | *0.20016* | *0.20582* | *0.2128* | *0.22219* | *0.22904* | *0.23578* | *0.2423* |
| *35493.46542* | *0.09559* | *0.10248* | *0.11121* | *0.11804* | *0.12442* | *0.12912* | *0.13505* | *0.153* | *0.15802* | *0.16293* | *0.17151* | *0.1885* | *0.19688* | *0.20248* | *0.20944* | *0.21862* | *0.22542* | *0.23225* | *0.23845* |
| *35489.04006* | *0.09259* | *0.09938* | *0.10799* | *0.11473* | *0.12101* | *0.12566* | *0.13154* | *0.15012* | *0.15507* | *0.15991* | *0.16845* | *0.18538* | *0.19367* | *0.19921* | *0.20614* | *0.21511* | *0.22187* | *0.22878* | *0.23466* |
| *35484.61581* | *0.08968* | *0.09637* | *0.10487* | *0.11152* | *0.11769* | *0.12229* | *0.12811* | *0.14731* | *0.15219* | *0.15697* | *0.16546* | *0.18233* | *0.19052* | *0.19601* | *0.20292* | *0.21168* | *0.21838* | *0.22538* | *0.23093* |
| *35480.19266* | *0.08687* | *0.09344* | *0.10183* | *0.10839* | *0.11447* | *0.11901* | *0.12476* | *0.14458* | *0.14939* | *0.15411* | *0.16255* | *0.17936* | *0.18745* | *0.19288* | *0.19976* | *0.20831* | *0.21497* | *0.22205* | *0.22727* |
| *35475.77062* | *0.08414* | *0.09061* | *0.09888* | *0.10535* | *0.11132* | *0.11582* | *0.1215* | *0.14192* | *0.14666* | *0.15132* | *0.15971* | *0.17645* | *0.18446* | *0.18983* | *0.19668* | *0.20501* | *0.21162* | *0.21878* | *0.22367* |
| *35471.34967* | *0.0815* | *0.08787* | *0.09602* | *0.10239* | *0.10827* | *0.11271* | *0.11833* | *0.13933* | *0.144* | *0.14859* | *0.15694* | *0.17362* | *0.18153* | *0.18684* | *0.19366* | *0.20178* | *0.20834* | *0.21558* | *0.22012* |
| *35466.92983* | *0.07894* | *0.08521* | *0.09324* | *0.09953* | *0.1053* | *0.10968* | *0.11523* | *0.1368* | *0.14141* | *0.14594* | *0.15423* | *0.17085* | *0.17866* | *0.18392* | *0.1907* | *0.19861* | *0.20512* | *0.21244* | *0.21665* |
| *35462.51109* | *0.07647* | *0.08264* | *0.09055* | *0.09674* | *0.10241* | *0.10674* | *0.11222* | *0.13435* | *0.13889* | *0.14336* | *0.1516* | *0.16815* | *0.17587* | *0.18106* | *0.18782* | *0.19551* | *0.20197* | *0.20936* | *0.21323* |
| *35458.09345* | *0.07408* | *0.08015* | *0.08794* | *0.09404* | *0.09961* | *0.10388* | *0.10929* | *0.13196* | *0.13643* | *0.14084* | *0.14903* | *0.16551* | *0.17314* | *0.17828* | *0.18499* | *0.19247* | *0.19888* | *0.20635* | *0.20987* |
| *35453.67691* | *0.07177* | *0.07774* | *0.08542* | *0.09141* | *0.09688* | *0.1011* | *0.10644* | *0.12963* | *0.13404* | *0.13838* | *0.14653* | *0.16294* | *0.17047* | *0.17555* | *0.18224* | *0.1895* | *0.19586* | *0.2034* | *0.20657* |
| *35449.26147* | *0.06953* | *0.07541* | *0.08297* | *0.08887* | *0.09424* | *0.09839* | *0.10366* | *0.12737* | *0.13171* | *0.13599* | *0.14409* | *0.16043* | *0.16787* | *0.17289* | *0.17954* | *0.18659* | *0.1929* | *0.20051* | *0.20334* |
| *35444.84713* | *0.06738* | *0.07315* | *0.0806* | *0.0864* | *0.09167* | *0.09577* | *0.10096* | *0.12517* | *0.12945* | *0.13367* | *0.14171* | *0.15799* | *0.16533* | *0.17029* | *0.1769* | *0.18375* | *0.19* | *0.19768* | *0.20016* |
| *35440.43389* | *0.06529* | *0.07097* | *0.0783* | *0.08401* | *0.08918* | *0.09322* | *0.09834* | *0.12302* | *0.12724* | *0.1314* | *0.1394* | *0.1556* | *0.16285* | *0.16776* | *0.17433* | *0.18096* | *0.18717* | *0.1949* | *0.19704* |
| *35436.02175* | *0.06328* | *0.06887* | *0.07608* | *0.0817* | *0.08676* | *0.09075* | *0.09579* | *0.12093* | *0.12509* | *0.12919* | *0.13714* | *0.15327* | *0.16043* | *0.16528* | *0.17181* | *0.17824* | *0.18439* | *0.19219* | *0.19398* |
| *35431.61071* | *0.06134* | *0.06684* | *0.07393* | *0.07946* | *0.08442* | *0.08835* | *0.09331* | *0.1189* | *0.123* | *0.12704* | *0.13494* | *0.151* | *0.15807* | *0.16286* | *0.16936* | *0.17558* | *0.18167* | *0.18954* | *0.19098* |
| *35427.20076* | *0.05947* | *0.06487* | *0.07185* | *0.07728* | *0.08215* | *0.08602* | *0.09091* | *0.11692* | *0.12096* | *0.12495* | *0.1328* | *0.14879* | *0.15576* | *0.1605* | *0.16696* | *0.17297* | *0.17901* | *0.18694* | *0.18804* |
| *35422.79191* | *0.05767* | *0.06298* | *0.06985* | *0.07518* | *0.07995* | *0.08376* | *0.08857* | *0.115* | *0.11898* | *0.12291* | *0.13072* | *0.14663* | *0.15352* | *0.1582* | *0.16461* | *0.17042* | *0.17641* | *0.18439* | *0.18515* |
| *35418.38416* | *0.05593* | *0.06115* | *0.06791* | *0.07315* | *0.07782* | *0.08158* | *0.08631* | *0.11312* | *0.11705* | *0.12092* | *0.12868* | *0.14452* | *0.15132* | *0.15595* | *0.16232* | *0.16793* | *0.17387* | *0.1819* | *0.18232* |
| *35413.97751* | *0.05426* | *0.05939* | *0.06603* | *0.07118* | *0.07576* | *0.07946* | *0.08411* | *0.1113* | *0.11517* | *0.11899* | *0.1267* | *0.14247* | *0.14919* | *0.15376* | *0.16009* | *0.1655* | *0.17138* | *0.17947* | *0.17955* |
| *35409.57195* | *0.05264* | *0.05769* | *0.06422* | *0.06928* | *0.07376* | *0.0774* | *0.08197* | *0.10952* | *0.11334* | *0.11711* | *0.12478* | *0.14047* | *0.1471* | *0.15162* | *0.15791* | *0.16312* | *0.16895* | *0.17709* | *0.17683* |
| *35405.16749* | *0.05109* | *0.05605* | *0.06248* | *0.06744* | *0.07183* | *0.07541* | *0.07991* | *0.1078* | *0.11156* | *0.11527* | *0.1229* | *0.13852* | *0.14506* | *0.14953* | *0.15578* | *0.1608* | *0.16658* | *0.17476* | *0.17416* |
| *35400.76413* | *0.0496* | *0.05447* | *0.06079* | *0.06567* | *0.06996* | *0.07349* | *0.0779* | *0.10611* | *0.10982* | *0.11349* | *0.12107* | *0.13662* | *0.14308* | *0.1475* | *0.15369* | *0.15853* | *0.16425* | *0.17248* | *0.17155* |
