# Supplementary material for: Additional data for evaluation of the excited state dipole moments of anisole
Source: Data Brief. 2018 Oct 3;21:313–5. doi: 10.1016/j.dib.2018.09.110 (PMC6197573; doi:10.1016/j.dib.2018.09.110)
Supplement: Supplementary file 6 — Supplementary material [file mmc6.docx]

*Table S3: Emission spectra of anisole in ethyl acetate between 258 K and 348 K in steps of 5 K.*

|  | *258 K* | *263 K* | *268 K* | *273 K* | *278 K* | *283 K* | *288 K* | *293 K* | *298 K* | *303 K* | *308 K* | *313 K* | *318 K* | *323 K* | *228 K* | *233 K* | *238 K* | *243 K* | *348 K* |
| --- | --- | --- | --- | --- | --- | --- | --- | --- | --- | --- | --- | --- | --- | --- | --- | --- | --- | --- | --- |
| *35714.28571* | *0.44095* | *0.44363* | *0.4405* | *0.43031* | *0.43219* | *0.43464* | *0.43114* | *0.42997* | *0.43219* | *0.42741* | *0.41376* | *0.41337* | *0.41291* | *0.41347* | *0.41498* | *0.40329* | *0.39714* | *0.39381* | *0.38373* |
| *35704.07434* | *0.44473* | *0.44769* | *0.44434* | *0.43425* | *0.43597* | *0.43838* | *0.4349* | *0.43374* | *0.4359* | *0.43115* | *0.41784* | *0.41711* | *0.41652* | *0.41708* | *0.41852* | *0.40693* | *0.40105* | *0.39737* | *0.3876* |
| *35693.8688* | *0.44853* | *0.45177* | *0.44819* | *0.43821* | *0.43978* | *0.44214* | *0.43868* | *0.43754* | *0.43964* | *0.43492* | *0.42195* | *0.42088* | *0.42017* | *0.42071* | *0.42209* | *0.4106* | *0.40499* | *0.40096* | *0.39151* |
| *35683.6691* | *0.45236* | *0.45587* | *0.45207* | *0.4422* | *0.44362* | *0.44591* | *0.44247* | *0.44135* | *0.44339* | *0.43872* | *0.42609* | *0.42467* | *0.42383* | *0.42438* | *0.42568* | *0.4143* | *0.40895* | *0.40458* | *0.39545* |
| *35673.47522* | *0.4562* | *0.45998* | *0.45596* | *0.44621* | *0.44747* | *0.44971* | *0.44629* | *0.44518* | *0.44717* | *0.44254* | *0.43025* | *0.42849* | *0.42753* | *0.42807* | *0.4293* | *0.41803* | *0.41294* | *0.40823* | *0.39941* |
| *35663.28716* | *0.46007* | *0.46411* | *0.45988* | *0.45026* | *0.45136* | *0.45354* | *0.45012* | *0.44903* | *0.45096* | *0.44639* | *0.43444* | *0.43234* | *0.43126* | *0.43179* | *0.43295* | *0.42179* | *0.41695* | *0.41192* | *0.4034* |
| *35653.10493* | *0.46396* | *0.46826* | *0.46383* | *0.45434* | *0.45526* | *0.45738* | *0.45397* | *0.4529* | *0.45477* | *0.45027* | *0.43866* | *0.43622* | *0.43501* | *0.43554* | *0.43662* | *0.42557* | *0.42099* | *0.41563* | *0.40743* |
| *35642.9285* | *0.46787* | *0.47242* | *0.46779* | *0.45844* | *0.45919* | *0.46125* | *0.45784* | *0.45678* | *0.4586* | *0.45417* | *0.44289* | *0.44013* | *0.4388* | *0.43931* | *0.44032* | *0.42939* | *0.42506* | *0.41938* | *0.41148* |
| *35632.75788* | *0.47181* | *0.47659* | *0.47178* | *0.46257* | *0.46315* | *0.46513* | *0.46172* | *0.46069* | *0.46245* | *0.45809* | *0.44716* | *0.44406* | *0.44261* | *0.44312* | *0.44404* | *0.43324* | *0.42914* | *0.42315* | *0.41555* |
| *35622.59307* | *0.47577* | *0.48078* | *0.47579* | *0.46673* | *0.46712* | *0.46905* | *0.46563* | *0.46461* | *0.46631* | *0.46205* | *0.45144* | *0.44802* | *0.44645* | *0.44695* | *0.44779* | *0.43712* | *0.43326* | *0.42696* | *0.41966* |
| *35612.43405* | *0.47975* | *0.48499* | *0.47983* | *0.47091* | *0.47113* | *0.47298* | *0.46955* | *0.46855* | *0.4702* | *0.46603* | *0.45576* | *0.45201* | *0.45032* | *0.45081* | *0.45157* | *0.44102* | *0.43739* | *0.4308* | *0.42379* |
| *35602.28083* | *0.48375* | *0.48921* | *0.48388* | *0.47513* | *0.47515* | *0.47693* | *0.47349* | *0.47251* | *0.4741* | *0.47003* | *0.46009* | *0.45602* | *0.45422* | *0.4547* | *0.45538* | *0.44496* | *0.44155* | *0.43467* | *0.42794* |
| *35592.13339* | *0.48778* | *0.49344* | *0.48796* | *0.47937* | *0.47921* | *0.48091* | *0.47745* | *0.47649* | *0.47802* | *0.47407* | *0.46445* | *0.46007* | *0.45814* | *0.45861* | *0.45921* | *0.44892* | *0.44573* | *0.43857* | *0.43213* |
| *35581.99174* | *0.49183* | *0.49768* | *0.49206* | *0.48363* | *0.48328* | *0.48491* | *0.48143* | *0.48049* | *0.48196* | *0.47813* | *0.46882* | *0.46413* | *0.4621* | *0.46256* | *0.46307* | *0.45291* | *0.44994* | *0.4425* | *0.43634* |
| *35571.85586* | *0.49591* | *0.50194* | *0.49619* | *0.48793* | *0.48738* | *0.48893* | *0.48542* | *0.48451* | *0.48592* | *0.48221* | *0.47323* | *0.46823* | *0.46608* | *0.46653* | *0.46696* | *0.45694* | *0.45416* | *0.44646* | *0.44057* |
| *35561.72576* | *0.50001* | *0.50621* | *0.50034* | *0.49225* | *0.49151* | *0.49298* | *0.48944* | *0.48854* | *0.4899* | *0.48632* | *0.47765* | *0.47235* | *0.47009* | *0.47053* | *0.47088* | *0.46099* | *0.45841* | *0.45046* | *0.44483* |
| *35551.60142* | *0.50413* | *0.5105* | *0.50451* | *0.49659* | *0.49566* | *0.49704* | *0.49347* | *0.49259* | *0.4939* | *0.49046* | *0.48209* | *0.4765* | *0.47413* | *0.47456* | *0.47482* | *0.46506* | *0.46268* | *0.45448* | *0.44912* |
| *35541.48285* | *0.50827* | *0.51479* | *0.5087* | *0.50097* | *0.49984* | *0.50113* | *0.49752* | *0.49666* | *0.49791* | *0.49463* | *0.48655* | *0.48067* | *0.4782* | *0.47862* | *0.4788* | *0.46917* | *0.46697* | *0.45853* | *0.45343* |
| *35531.37004* | *0.51244* | *0.5191* | *0.51291* | *0.50536* | *0.50404* | *0.50525* | *0.50159* | *0.50075* | *0.50195* | *0.49882* | *0.49104* | *0.48487* | *0.48229* | *0.4827* | *0.4828* | *0.47331* | *0.47129* | *0.46261* | *0.45776* |
| *35521.26298* | *0.51663* | *0.52341* | *0.51715* | *0.50979* | *0.50826* | *0.50938* | *0.50567* | *0.50486* | *0.506* | *0.50304* | *0.49554* | *0.48909* | *0.48641* | *0.48681* | *0.48682* | *0.47747* | *0.47562* | *0.46673* | *0.46212* |
| *35511.16167* | *0.52085* | *0.52774* | *0.52141* | *0.51423* | *0.51251* | *0.51354* | *0.50977* | *0.50899* | *0.51007* | *0.50728* | *0.50006* | *0.49334* | *0.49056* | *0.49095* | *0.49088* | *0.48166* | *0.47997* | *0.47087* | *0.4665* |
| *35501.0661* | *0.52509* | *0.53207* | *0.5257* | *0.5187* | *0.51679* | *0.51772* | *0.5139* | *0.51313* | *0.51416* | *0.51156* | *0.50461* | *0.49761* | *0.49474* | *0.49512* | *0.49496* | *0.48587* | *0.48434* | *0.47504* | *0.47091* |
| *35490.97627* | *0.52935* | *0.53642* | *0.53* | *0.5232* | *0.52109* | *0.52193* | *0.51804* | *0.5173* | *0.51827* | *0.51585* | *0.50916* | *0.50191* | *0.49894* | *0.49931* | *0.49907* | *0.49012* | *0.48873* | *0.47924* | *0.47533* |
| *35480.89217* | *0.53364* | *0.54077* | *0.53433* | *0.52772* | *0.52542* | *0.52616* | *0.52219* | *0.52148* | *0.5224* | *0.52018* | *0.51374* | *0.50623* | *0.50317* | *0.50353* | *0.50321* | *0.49439* | *0.49314* | *0.48347* | *0.47978* |
| *35470.8138* | *0.53795* | *0.54514* | *0.53869* | *0.53226* | *0.52977* | *0.53041* | *0.52637* | *0.52569* | *0.52655* | *0.52453* | *0.51833* | *0.51057* | *0.50743* | *0.50777* | *0.50738* | *0.49869* | *0.49757* | *0.48773* | *0.48425* |
| *35460.74116* | *0.54229* | *0.54951* | *0.54306* | *0.53682* | *0.53414* | *0.53468* | *0.53056* | *0.52991* | *0.53072* | *0.5289* | *0.52294* | *0.51494* | *0.51171* | *0.51205* | *0.51158* | *0.50301* | *0.50201* | *0.49202* | *0.48874* |
| *35450.67424* | *0.54665* | *0.55389* | *0.54746* | *0.54141* | *0.53854* | *0.53898* | *0.53477* | *0.53415* | *0.5349* | *0.5333* | *0.52757* | *0.51932* | *0.51602* | *0.51635* | *0.5158* | *0.50736* | *0.50647* | *0.49633* | *0.49325* |
| *35440.61303* | *0.55103* | *0.55827* | *0.55188* | *0.54602* | *0.54297* | *0.5433* | *0.539* | *0.53841* | *0.53911* | *0.53773* | *0.53221* | *0.52374* | *0.52036* | *0.52067* | *0.52005* | *0.51173* | *0.51095* | *0.50068* | *0.49778* |
| *35430.55753* | *0.55544* | *0.56266* | *0.55632* | *0.55065* | *0.54742* | *0.54765* | *0.54325* | *0.54269* | *0.54334* | *0.54218* | *0.53686* | *0.52817* | *0.52472* | *0.52502* | *0.52433* | *0.51613* | *0.51544* | *0.50505* | *0.50232* |
| *35420.50773* | *0.55987* | *0.56706* | *0.56078* | *0.5553* | *0.55189* | *0.55202* | *0.54752* | *0.54699* | *0.54758* | *0.54666* | *0.54153* | *0.53262* | *0.5291* | *0.5294* | *0.52864* | *0.52055* | *0.51995* | *0.50945* | *0.50689* |
| *35410.46363* | *0.56433* | *0.57146* | *0.56527* | *0.55997* | *0.55639* | *0.55641* | *0.5518* | *0.55131* | *0.55185* | *0.55116* | *0.54621* | *0.5371* | *0.53351* | *0.5338* | *0.53297* | *0.525* | *0.52448* | *0.51387* | *0.51148* |
| *35400.42523* | *0.56881* | *0.57587* | *0.56977* | *0.56465* | *0.56091* | *0.56082* | *0.5561* | *0.55565* | *0.55613* | *0.55569* | *0.55091* | *0.54159* | *0.53795* | *0.53822* | *0.53733* | *0.52947* | *0.52902* | *0.51832* | *0.51608* |
| *35390.39252* | *0.57331* | *0.58029* | *0.5743* | *0.56936* | *0.56546* | *0.56526* | *0.56043* | *0.56001* | *0.56044* | *0.56024* | *0.55562* | *0.54611* | *0.54241* | *0.54267* | *0.54172* | *0.53396* | *0.53357* | *0.5228* | *0.5207* |
| *35380.36549* | *0.57783* | *0.58471* | *0.57885* | *0.57409* | *0.57003* | *0.56972* | *0.56476* | *0.56439* | *0.56477* | *0.56481* | *0.56033* | *0.55064* | *0.54689* | *0.54715* | *0.54614* | *0.53848* | *0.53813* | *0.5273* | *0.52534* |
| *35370.34414* | *0.58238* | *0.58913* | *0.58343* | *0.57883* | *0.57463* | *0.5742* | *0.56912* | *0.5688* | *0.56911* | *0.56941* | *0.56506* | *0.55519* | *0.5514* | *0.55164* | *0.55058* | *0.54302* | *0.54271* | *0.53182* | *0.52999* |
| *35360.32847* | *0.58695* | *0.59356* | *0.58802* | *0.58359* | *0.57924* | *0.57871* | *0.5735* | *0.57322* | *0.57348* | *0.57403* | *0.5698* | *0.55976* | *0.55592* | *0.55616* | *0.55505* | *0.54758* | *0.5473* | *0.53637* | *0.53466* |
| *35350.31847* | *0.59155* | *0.59799* | *0.59263* | *0.58837* | *0.58389* | *0.58324* | *0.57789* | *0.57766* | *0.57787* | *0.57868* | *0.57455* | *0.56435* | *0.56047* | *0.56071* | *0.55955* | *0.55216* | *0.5519* | *0.54095* | *0.53934* |
| *35340.31414* | *0.59617* | *0.60242* | *0.59726* | *0.59316* | *0.58855* | *0.58779* | *0.5823* | *0.58213* | *0.58227* | *0.58335* | *0.57931* | *0.56896* | *0.56504* | *0.56527* | *0.56407* | *0.55676* | *0.55651* | *0.54555* | *0.54403* |
| *35330.31546* | *0.60081* | *0.60685* | *0.60192* | *0.59797* | *0.59324* | *0.59236* | *0.58673* | *0.58661* | *0.5867* | *0.58804* | *0.58407* | *0.57358* | *0.56964* | *0.56986* | *0.56862* | *0.56138* | *0.56114* | *0.55017* | *0.54874* |
| *35320.32244* | *0.60547* | *0.61129* | *0.60659* | *0.60279* | *0.59795* | *0.59696* | *0.59118* | *0.59112* | *0.59115* | *0.59275* | *0.58885* | *0.57822* | *0.57425* | *0.57447* | *0.57319* | *0.56603* | *0.56577* | *0.55481* | *0.55346* |
| *35310.33508* | *0.61015* | *0.61573* | *0.61128* | *0.60763* | *0.60268* | *0.60157* | *0.59564* | *0.59565* | *0.59562* | *0.59748* | *0.59362* | *0.58287* | *0.57888* | *0.57909* | *0.57779* | *0.57068* | *0.57041* | *0.55947* | *0.5582* |
| *35300.35336* | *0.61486* | *0.62017* | *0.616* | *0.61247* | *0.60743* | *0.60621* | *0.60013* | *0.6002* | *0.60011* | *0.60223* | *0.59841* | *0.58754* | *0.58354* | *0.58374* | *0.58241* | *0.57536* | *0.57506* | *0.56416* | *0.56294* |
| *35290.37728* | *0.61958* | *0.62462* | *0.62073* | *0.61733* | *0.61221* | *0.61087* | *0.60463* | *0.60478* | *0.60462* | *0.607* | *0.6032* | *0.59222* | *0.58821* | *0.58841* | *0.58706* | *0.58006* | *0.57972* | *0.56886* | *0.5677* |
| *35280.40684* | *0.62433* | *0.62906* | *0.62547* | *0.6222* | *0.617* | *0.61555* | *0.60915* | *0.60937* | *0.60916* | *0.61179* | *0.60799* | *0.59691* | *0.5929* | *0.5931* | *0.59173* | *0.58477* | *0.58438* | *0.57359* | *0.57246* |
| *35270.44203* | *0.6291* | *0.63351* | *0.63024* | *0.62708* | *0.62181* | *0.62025* | *0.61369* | *0.61399* | *0.61371* | *0.6166* | *0.61279* | *0.60162* | *0.5976* | *0.5978* | *0.59642* | *0.5895* | *0.58905* | *0.57833* | *0.57724* |
| *35260.48285* | *0.63388* | *0.63795* | *0.63502* | *0.63197* | *0.62665* | *0.62497* | *0.61824* | *0.61863* | *0.61828* | *0.62143* | *0.61759* | *0.60633* | *0.60232* | *0.60252* | *0.60114* | *0.59424* | *0.59373* | *0.58309* | *0.58202* |
| *35250.52929* | *0.63869* | *0.6424* | *0.63982* | *0.63687* | *0.6315* | *0.62972* | *0.62281* | *0.62329* | *0.62288* | *0.62628* | *0.6224* | *0.61106* | *0.60706* | *0.60726* | *0.60588* | *0.599* | *0.59841* | *0.58787* | *0.58681* |
| *35240.58135* | *0.64351* | *0.64685* | *0.64463* | *0.64178* | *0.63637* | *0.63448* | *0.6274* | *0.62797* | *0.62749* | *0.63114* | *0.6272* | *0.6158* | *0.61182* | *0.61201* | *0.61063* | *0.60377* | *0.60309* | *0.59266* | *0.59161* |
| *35230.63902* | *0.64836* | *0.6513* | *0.64946* | *0.64669* | *0.64126* | *0.63925* | *0.63201* | *0.63268* | *0.63213* | *0.63601* | *0.63201* | *0.62055* | *0.61659* | *0.61678* | *0.61542* | *0.60855* | *0.60778* | *0.59748* | *0.59641* |
| *35220.7023* | *0.65322* | *0.65575* | *0.65431* | *0.65161* | *0.64617* | *0.64405* | *0.63663* | *0.63741* | *0.63678* | *0.64091* | *0.63681* | *0.62531* | *0.62137* | *0.62157* | *0.62022* | *0.61335* | *0.61248* | *0.6023* | *0.60122* |
| *35210.77118* | *0.65809* | *0.66021* | *0.65917* | *0.65653* | *0.65109* | *0.64886* | *0.64127* | *0.64216* | *0.64146* | *0.64581* | *0.64162* | *0.63007* | *0.62617* | *0.62636* | *0.62504* | *0.61816* | *0.61717* | *0.60714* | *0.60603* |
| *35200.84567* | *0.66299* | *0.66466* | *0.66404* | *0.66146* | *0.65603* | *0.6537* | *0.64593* | *0.64693* | *0.64615* | *0.65073* | *0.64642* | *0.63484* | *0.63097* | *0.63117* | *0.62988* | *0.62298* | *0.62187* | *0.61199* | *0.61085* |
| *35190.92574* | *0.66789* | *0.66912* | *0.66892* | *0.66639* | *0.66098* | *0.65854* | *0.6506* | *0.65173* | *0.65087* | *0.65566* | *0.65122* | *0.63962* | *0.63579* | *0.63599* | *0.63473* | *0.62781* | *0.62657* | *0.61686* | *0.61567* |
| *35181.01141* | *0.67282* | *0.67358* | *0.67381* | *0.67132* | *0.66595* | *0.66341* | *0.65529* | *0.65654* | *0.6556* | *0.6606* | *0.65602* | *0.64441* | *0.64062* | *0.64083* | *0.63961* | *0.63264* | *0.63126* | *0.62174* | *0.62049* |
| *35171.10266* | *0.67775* | *0.67804* | *0.67872* | *0.67626* | *0.67093* | *0.66828* | *0.65999* | *0.66138* | *0.66036* | *0.66556* | *0.66081* | *0.6492* | *0.64546* | *0.64567* | *0.6445* | *0.63749* | *0.63596* | *0.62662* | *0.62532* |
| *35161.19949* | *0.6827* | *0.68251* | *0.68363* | *0.68119* | *0.67592* | *0.67317* | *0.66471* | *0.66624* | *0.66513* | *0.67052* | *0.6656* | *0.65399* | *0.65031* | *0.65052* | *0.6494* | *0.64234* | *0.64066* | *0.63152* | *0.63014* |
| *35151.3019* | *0.68767* | *0.68698* | *0.68856* | *0.68612* | *0.68093* | *0.67808* | *0.66944* | *0.67112* | *0.66992* | *0.67549* | *0.67038* | *0.65878* | *0.65516* | *0.65538* | *0.65432* | *0.6472* | *0.64535* | *0.63643* | *0.63497* |
| *35141.40988* | *0.69264* | *0.69145* | *0.69349* | *0.69105* | *0.68594* | *0.683* | *0.67419* | *0.67602* | *0.67472* | *0.68047* | *0.67515* | *0.66358* | *0.66002* | *0.66025* | *0.65926* | *0.65206* | *0.65004* | *0.64134* | *0.63979* |
| *35131.52342* | *0.69762* | *0.69593* | *0.69843* | *0.69598* | *0.69096* | *0.68792* | *0.67895* | *0.68094* | *0.67955* | *0.68545* | *0.67992* | *0.66838* | *0.66489* | *0.66512* | *0.6642* | *0.65693* | *0.65473* | *0.64626* | *0.64462* |
| *35121.64253* | *0.70261* | *0.70042* | *0.70337* | *0.7009* | *0.69599* | *0.69286* | *0.68372* | *0.68588* | *0.68439* | *0.69043* | *0.68468* | *0.67317* | *0.66976* | *0.67* | *0.66916* | *0.6618* | *0.65941* | *0.65119* | *0.64944* |
| *35111.76719* | *0.70761* | *0.70491* | *0.70832* | *0.70581* | *0.70103* | *0.69781* | *0.68851* | *0.69083* | *0.68924* | *0.69543* | *0.68943* | *0.67797* | *0.67464* | *0.67489* | *0.67413* | *0.66667* | *0.66409* | *0.65612* | *0.65425* |
| *35101.8974* | *0.71262* | *0.70941* | *0.71327* | *0.71072* | *0.70607* | *0.70277* | *0.6933* | *0.69581* | *0.69411* | *0.70042* | *0.69418* | *0.68277* | *0.67952* | *0.67977* | *0.67911* | *0.67155* | *0.66876* | *0.66105* | *0.65907* |
| *35092.03316* | *0.71763* | *0.71392* | *0.71823* | *0.71563* | *0.71112* | *0.70773* | *0.69811* | *0.7008* | *0.699* | *0.70541* | *0.69891* | *0.68756* | *0.6844* | *0.68466* | *0.68409* | *0.67642* | *0.67343* | *0.66599* | *0.66387* |
| *35082.17446* | *0.72265* | *0.71844* | *0.72318* | *0.72052* | *0.71617* | *0.7127* | *0.70293* | *0.70581* | *0.70389* | *0.71041* | *0.70363* | *0.69235* | *0.68928* | *0.68955* | *0.68909* | *0.6813* | *0.67809* | *0.67092* | *0.66868* |
| *35072.3213* | *0.72766* | *0.72297* | *0.72814* | *0.7254* | *0.72122* | *0.71768* | *0.70775* | *0.71083* | *0.70881* | *0.7154* | *0.70833* | *0.69713* | *0.69416* | *0.69444* | *0.69408* | *0.68617* | *0.68274* | *0.67586* | *0.67347* |
| *35062.47368* | *0.73268* | *0.72752* | *0.73309* | *0.73028* | *0.72628* | *0.72265* | *0.71259* | *0.71587* | *0.71373* | *0.72039* | *0.71303* | *0.70191* | *0.69904* | *0.69933* | *0.69909* | *0.69104* | *0.68738* | *0.6808* | *0.67826* |
| *35052.63158* | *0.73771* | *0.73208* | *0.73804* | *0.73514* | *0.73133* | *0.72763* | *0.71743* | *0.72093* | *0.71866* | *0.72537* | *0.71771* | *0.70668* | *0.70392* | *0.70422* | *0.7041* | *0.6959* | *0.69202* | *0.68573* | *0.68304* |
| *35042.795* | *0.74273* | *0.73665* | *0.74299* | *0.73998* | *0.73638* | *0.73262* | *0.72228* | *0.72599* | *0.7236* | *0.73035* | *0.72237* | *0.71145* | *0.70879* | *0.7091* | *0.70911* | *0.70076* | *0.69664* | *0.69067* | *0.68781* |
| *35032.96395* | *0.74774* | *0.74124* | *0.74793* | *0.74482* | *0.74143* | *0.7376* | *0.72713* | *0.73107* | *0.72855* | *0.73532* | *0.72702* | *0.7162* | *0.71366* | *0.71398* | *0.71412* | *0.70562* | *0.70125* | *0.69559* | *0.69257* |
| *35023.13841* | *0.75276* | *0.74585* | *0.75287* | *0.74963* | *0.74647* | *0.74258* | *0.73199* | *0.73615* | *0.73351* | *0.74028* | *0.73165* | *0.72095* | *0.71852* | *0.71885* | *0.71912* | *0.71046* | *0.70585* | *0.70052* | *0.69732* |
| *35013.31838* | *0.75776* | *0.75048* | *0.7578* | *0.75443* | *0.7515* | *0.74755* | *0.73686* | *0.74125* | *0.73847* | *0.74524* | *0.73627* | *0.72569* | *0.72337* | *0.72371* | *0.72413* | *0.7153* | *0.71044* | *0.70543* | *0.70206* |
| *35003.50385* | *0.76277* | *0.75513* | *0.76271* | *0.75922* | *0.75653* | *0.75253* | *0.74172* | *0.74635* | *0.74344* | *0.75018* | *0.74087* | *0.73041* | *0.72822* | *0.72857* | *0.72914* | *0.72013* | *0.71502* | *0.71034* | *0.70678* |
| *34993.69483* | *0.76776* | *0.75981* | *0.76762* | *0.76398* | *0.76154* | *0.75749* | *0.74659* | *0.75146* | *0.74841* | *0.75511* | *0.74545* | *0.73512* | *0.73305* | *0.73342* | *0.73414* | *0.72495* | *0.71958* | *0.71524* | *0.71149* |
| *34983.8913* | *0.77274* | *0.7645* | *0.77251* | *0.76872* | *0.76655* | *0.76245* | *0.75145* | *0.75657* | *0.75338* | *0.76002* | *0.75* | *0.73982* | *0.73788* | *0.73825* | *0.73913* | *0.72976* | *0.72412* | *0.72013* | *0.71619* |
| *34974.09326* | *0.77771* | *0.76922* | *0.77739* | *0.77344* | *0.77154* | *0.7674* | *0.75631* | *0.76168* | *0.75835* | *0.76492* | *0.75454* | *0.7445* | *0.74269* | *0.74308* | *0.74411* | *0.73455* | *0.72865* | *0.72501* | *0.72088* |
| *34964.30071* | *0.78267* | *0.77397* | *0.78226* | *0.77814* | *0.77652* | *0.77234* | *0.76117* | *0.76679* | *0.76332* | *0.7698* | *0.75906* | *0.74917* | *0.74749* | *0.74789* | *0.74909* | *0.73934* | *0.73316* | *0.72987* | *0.72554* |
| *34954.51365* | *0.78761* | *0.77874* | *0.7871* | *0.78282* | *0.78148* | *0.77726* | *0.76603* | *0.7719* | *0.76829* | *0.77465* | *0.76355* | *0.75382* | *0.75228* | *0.75268* | *0.75406* | *0.7441* | *0.73766* | *0.73472* | *0.73019* |
| *34944.73206* | *0.79253* | *0.78354* | *0.79193* | *0.78747* | *0.78642* | *0.78217* | *0.77088* | *0.77701* | *0.77325* | *0.77949* | *0.76803* | *0.75846* | *0.75705* | *0.75746* | *0.75901* | *0.74885* | *0.74214* | *0.73956* | *0.73482* |
| *34934.95594* | *0.79744* | *0.78837* | *0.79674* | *0.79209* | *0.79135* | *0.78707* | *0.77572* | *0.78211* | *0.77821* | *0.78431* | *0.77247* | *0.76307* | *0.7618* | *0.76222* | *0.76395* | *0.75358* | *0.7466* | *0.74438* | *0.73944* |
| *34925.18529* | *0.80233* | *0.79322* | *0.80152* | *0.79669* | *0.79625* | *0.79194* | *0.78056* | *0.7872* | *0.78315* | *0.7891* | *0.7769* | *0.76767* | *0.76653* | *0.76696* | *0.76887* | *0.7583* | *0.75104* | *0.74918* | *0.74403* |
| *34915.4201* | *0.80719* | *0.79811* | *0.80628* | *0.80125* | *0.80113* | *0.7968* | *0.78538* | *0.79228* | *0.78809* | *0.79387* | *0.78129* | *0.77224* | *0.77125* | *0.77169* | *0.77378* | *0.76299* | *0.75546* | *0.75396* | *0.7486* |
| *34905.66038* | *0.81203* | *0.80302* | *0.81102* | *0.80579* | *0.80598* | *0.80164* | *0.79019* | *0.79735* | *0.79301* | *0.79861* | *0.78567* | *0.77679* | *0.77594* | *0.77639* | *0.77867* | *0.76767* | *0.75986* | *0.75873* | *0.75316* |
| *34895.90611* | *0.81684* | *0.80796* | *0.81572* | *0.8103* | *0.81081* | *0.80645* | *0.79499* | *0.80241* | *0.79792* | *0.80332* | *0.79001* | *0.78132* | *0.78061* | *0.78107* | *0.78353* | *0.77232* | *0.76424* | *0.76347* | *0.75769* |
| *34886.15728* | *0.82162* | *0.81292* | *0.8204* | *0.81477* | *0.81561* | *0.81124* | *0.79977* | *0.80744* | *0.80281* | *0.80799* | *0.79433* | *0.78583* | *0.78526* | *0.78572* | *0.78838* | *0.77695* | *0.76859* | *0.76819* | *0.7622* |
| *34876.41391* | *0.82638* | *0.81792* | *0.82505* | *0.81921* | *0.82037* | *0.816* | *0.80453* | *0.81246* | *0.80769* | *0.81264* | *0.79861* | *0.79031* | *0.78988* | *0.79036* | *0.7932* | *0.78155* | *0.77292* | *0.77289* | *0.76668* |
| *34866.67597* | *0.8311* | *0.82294* | *0.82966* | *0.82362* | *0.82511* | *0.82073* | *0.80928* | *0.81746* | *0.81254* | *0.81725* | *0.80287* | *0.79476* | *0.79448* | *0.79496* | *0.798* | *0.78613* | *0.77723* | *0.77756* | *0.77114* |
| *34856.94348* | *0.83579* | *0.82798* | *0.83424* | *0.82799* | *0.82981* | *0.82543* | *0.814* | *0.82243* | *0.81737* | *0.82183* | *0.8071* | *0.79919* | *0.79905* | *0.79953* | *0.80277* | *0.79068* | *0.78151* | *0.78221* | *0.77557* |
| *34847.21641* | *0.84044* | *0.83304* | *0.83878* | *0.83232* | *0.83447* | *0.8301* | *0.8187* | *0.82737* | *0.82217* | *0.82637* | *0.8113* | *0.80358* | *0.8036* | *0.80408* | *0.80751* | *0.79521* | *0.78577* | *0.78682* | *0.77998* |
| *34837.49477* | *0.84506* | *0.83812* | *0.84329* | *0.83662* | *0.8391* | *0.83474* | *0.82337* | *0.83229* | *0.82695* | *0.83088* | *0.81546* | *0.80795* | *0.80811* | *0.8086* | *0.81222* | *0.7997* | *0.79* | *0.79141* | *0.78436* |
| *34827.77855* | *0.84964* | *0.84321* | *0.84775* | *0.84088* | *0.84369* | *0.83934* | *0.82802* | *0.83717* | *0.8317* | *0.83534* | *0.81959* | *0.81229* | *0.81259* | *0.81308* | *0.8169* | *0.80417* | *0.79421* | *0.79597* | *0.78872* |
| *34818.06775* | *0.85417* | *0.84832* | *0.85218* | *0.8451* | *0.84823* | *0.8439* | *0.83264* | *0.84201* | *0.83642* | *0.83976* | *0.82369* | *0.8166* | *0.81704* | *0.81753* | *0.82154* | *0.80861* | *0.79838* | *0.8005* | *0.79304* |
| *34808.36237* | *0.85866* | *0.85344* | *0.85656* | *0.84927* | *0.85273* | *0.84842* | *0.83723* | *0.84682* | *0.8411* | *0.84414* | *0.82776* | *0.82088* | *0.82146* | *0.82195* | *0.82615* | *0.81301* | *0.80253* | *0.805* | *0.79734* |
| *34798.66239* | *0.86311* | *0.85856* | *0.8609* | *0.85341* | *0.85719* | *0.8529* | *0.84179* | *0.85158* | *0.84574* | *0.84847* | *0.83179* | *0.82512* | *0.82584* | *0.82633* | *0.83073* | *0.81738* | *0.80665* | *0.80947* | *0.80161* |
| *34788.96782* | *0.86751* | *0.86368* | *0.86519* | *0.8575* | *0.8616* | *0.85733* | *0.84631* | *0.85631* | *0.85035* | *0.85275* | *0.83578* | *0.82933* | *0.83019* | *0.83068* | *0.83526* | *0.82172* | *0.81074* | *0.8139* | *0.80584* |
| *34779.27865* | *0.87186* | *0.8688* | *0.86943* | *0.86155* | *0.86596* | *0.86172* | *0.85079* | *0.86098* | *0.85492* | *0.85699* | *0.83974* | *0.8335* | *0.8345* | *0.83498* | *0.83976* | *0.82602* | *0.8148* | *0.81829* | *0.81004* |
| *34769.59488* | *0.87616* | *0.8739* | *0.87362* | *0.86555* | *0.87027* | *0.86606* | *0.85523* | *0.86561* | *0.85944* | *0.86119* | *0.84366* | *0.83764* | *0.83877* | *0.83925* | *0.84421* | *0.83028* | *0.81883* | *0.82265* | *0.81422* |
| *34759.91649* | *0.88041* | *0.87899* | *0.87777* | *0.8695* | *0.87453* | *0.87036* | *0.85964* | *0.87018* | *0.86391* | *0.86533* | *0.84755* | *0.84174* | *0.84301* | *0.84348* | *0.84862* | *0.83451* | *0.82283* | *0.82697* | *0.81835* |
| *34750.2435* | *0.8846* | *0.88406* | *0.88185* | *0.87341* | *0.87873* | *0.8746* | *0.864* | *0.8747* | *0.86834* | *0.86941* | *0.8514* | *0.8458* | *0.8472* | *0.84767* | *0.85298* | *0.8387* | *0.82679* | *0.83125* | *0.82246* |
| *34740.57588* | *0.88873* | *0.8891* | *0.88589* | *0.87727* | *0.88288* | *0.87879* | *0.86831* | *0.87917* | *0.87272* | *0.87345* | *0.8552* | *0.84982* | *0.85135* | *0.85181* | *0.8573* | *0.84285* | *0.83072* | *0.83549* | *0.82653* |
| *34730.91364* | *0.89281* | *0.8941* | *0.88987* | *0.88108* | *0.88698* | *0.88292* | *0.87258* | *0.88357* | *0.87704* | *0.87743* | *0.85897* | *0.85381* | *0.85546* | *0.85591* | *0.86157* | *0.84696* | *0.83462* | *0.8397* | *0.83056* |
| *34721.25678* | *0.89683* | *0.89906* | *0.89379* | *0.88484* | *0.89101* | *0.887* | *0.8768* | *0.88791* | *0.88132* | *0.88136* | *0.8627* | *0.85775* | *0.85952* | *0.85996* | *0.8658* | *0.85102* | *0.83848* | *0.84385* | *0.83456* |
| *34711.60528* | *0.90079* | *0.90396* | *0.89765* | *0.88855* | *0.89498* | *0.89102* | *0.88097* | *0.89219* | *0.88553* | *0.88523* | *0.86639* | *0.86165* | *0.86354* | *0.86397* | *0.86997* | *0.85505* | *0.84231* | *0.84797* | *0.83852* |
| *34701.95915* | *0.90468* | *0.90881* | *0.90145* | *0.89221* | *0.8989* | *0.89499* | *0.88509* | *0.89639* | *0.88968* | *0.88904* | *0.87003* | *0.86551* | *0.86752* | *0.86793* | *0.87408* | *0.85903* | *0.8461* | *0.85204* | *0.84245* |
| *34692.31838* | *0.90851* | *0.9136* | *0.9052* | *0.89582* | *0.90275* | *0.89889* | *0.88915* | *0.90053* | *0.89378* | *0.8928* | *0.87363* | *0.86933* | *0.87145* | *0.87184* | *0.87815* | *0.86297* | *0.84986* | *0.85607* | *0.84633* |
| *34682.68296* | *0.91228* | *0.91831* | *0.90887* | *0.89937* | *0.90653* | *0.90273* | *0.89315* | *0.9046* | *0.89781* | *0.89649* | *0.87719* | *0.8731* | *0.87532* | *0.87571* | *0.88216* | *0.86686* | *0.85358* | *0.86005* | *0.85018* |
| *34673.05289* | *0.91597* | *0.92294* | *0.91249* | *0.90287* | *0.91025* | *0.9065* | *0.8971* | *0.90859* | *0.90177* | *0.90012* | *0.88071* | *0.87682* | *0.87916* | *0.87952* | *0.88611* | *0.87071* | *0.85726* | *0.86399* | *0.85399* |
| *34663.42817* | *0.9196* | *0.92749* | *0.91604* | *0.90631* | *0.9139* | *0.91021* | *0.90099* | *0.91251* | *0.90567* | *0.9037* | *0.88419* | *0.8805* | *0.88294* | *0.88328* | *0.89001* | *0.8745* | *0.8609* | *0.86787* | *0.85776* |
| *34653.8088* | *0.92315* | *0.93194* | *0.91952* | *0.90969* | *0.91748* | *0.91385* | *0.90481* | *0.91634* | *0.9095* | *0.90721* | *0.88761* | *0.88414* | *0.88667* | *0.88699* | *0.89385* | *0.87826* | *0.86451* | *0.87171* | *0.86148* |
| *34644.19476* | *0.92664* | *0.93628* | *0.92293* | *0.91302* | *0.921* | *0.91743* | *0.90857* | *0.9201* | *0.91326* | *0.91065* | *0.891* | *0.88772* | *0.89034* | *0.89065* | *0.89763* | *0.88196* | *0.86807* | *0.8755* | *0.86517* |
| *34634.58605* | *0.93005* | *0.94051* | *0.92628* | *0.9163* | *0.92444* | *0.92093* | *0.91227* | *0.92377* | *0.91695* | *0.91403* | *0.89434* | *0.89126* | *0.89397* | *0.89425* | *0.90134* | *0.88561* | *0.8716* | *0.87924* | *0.86881* |
| *34624.98267* | *0.93339* | *0.94463* | *0.92955* | *0.91951* | *0.92781* | *0.92436* | *0.91589* | *0.92737* | *0.92056* | *0.91735* | *0.89763* | *0.89475* | *0.89754* | *0.8978* | *0.905* | *0.88921* | *0.87508* | *0.88293* | *0.87241* |
| *34615.38462* | *0.93665* | *0.94862* | *0.93276* | *0.92267* | *0.93111* | *0.92773* | *0.91945* | *0.93087* | *0.9241* | *0.9206* | *0.90088* | *0.89819* | *0.90106* | *0.90129* | *0.90859* | *0.89276* | *0.87853* | *0.88657* | *0.87597* |
| *34605.79188* | *0.93983* | *0.95248* | *0.93589* | *0.92576* | *0.93433* | *0.93102* | *0.92294* | *0.93429* | *0.92756* | *0.92379* | *0.90408* | *0.90158* | *0.90452* | *0.90473* | *0.91212* | *0.89626* | *0.88193* | *0.89015* | *0.87949* |
| *34596.20446* | *0.94293* | *0.9562* | *0.93895* | *0.9288* | *0.93748* | *0.93423* | *0.92636* | *0.93762* | *0.93094* | *0.9269* | *0.90723* | *0.90492* | *0.90793* | *0.90811* | *0.91558* | *0.89971* | *0.88529* | *0.89368* | *0.88296* |
| *34586.62235* | *0.94596* | *0.95978* | *0.94194* | *0.93178* | *0.94055* | *0.93737* | *0.92971* | *0.94086* | *0.93424* | *0.92995* | *0.91033* | *0.9082* | *0.91128* | *0.91143* | *0.91897* | *0.9031* | *0.88861* | *0.89716* | *0.88638* |
| *34577.04555* | *0.94891* | *0.96322* | *0.94485* | *0.93469* | *0.94355* | *0.94044* | *0.93298* | *0.94401* | *0.93746* | *0.93294* | *0.91338* | *0.91144* | *0.91457* | *0.9147* | *0.9223* | *0.90644* | *0.89189* | *0.90058* | *0.88976* |
| *34567.47405* | *0.95178* | *0.9665* | *0.94769* | *0.93755* | *0.94647* | *0.94343* | *0.93618* | *0.94707* | *0.9406* | *0.93585* | *0.91639* | *0.91462* | *0.9178* | *0.9179* | *0.92556* | *0.90973* | *0.89512* | *0.90395* | *0.8931* |
| *34557.90785* | *0.95456* | *0.96963* | *0.95045* | *0.94034* | *0.94931* | *0.94634* | *0.9393* | *0.95004* | *0.94366* | *0.9387* | *0.91935* | *0.91775* | *0.92098* | *0.92104* | *0.92875* | *0.91296* | *0.89831* | *0.90726* | *0.89638* |
| *34548.34694* | *0.95726* | *0.9726* | *0.95313* | *0.94307* | *0.95207* | *0.94917* | *0.94234* | *0.95291* | *0.94663* | *0.94147* | *0.92225* | *0.92082* | *0.92409* | *0.92413* | *0.93187* | *0.91613* | *0.90145* | *0.91051* | *0.89963* |
| *34538.79132* | *0.95988* | *0.97541* | *0.95574* | *0.94573* | *0.95476* | *0.95193* | *0.94531* | *0.95569* | *0.94952* | *0.94418* | *0.92511* | *0.92384* | *0.92715* | *0.92715* | *0.93493* | *0.91925* | *0.90455* | *0.91371* | *0.90282* |
| *34529.24098* | *0.96242* | *0.97806* | *0.95828* | *0.94834* | *0.95736* | *0.95461* | *0.9482* | *0.95838* | *0.95233* | *0.94682* | *0.92792* | *0.9268* | *0.93014* | *0.93011* | *0.93791* | *0.92231* | *0.9076* | *0.91685* | *0.90597* |
| *34519.69592* | *0.96487* | *0.98054* | *0.96073* | *0.95088* | *0.95989* | *0.9572* | *0.95101* | *0.96098* | *0.95504* | *0.94939* | *0.93067* | *0.92971* | *0.93308* | *0.93301* | *0.94082* | *0.92532* | *0.91061* | *0.91994* | *0.90906* |
| *34510.15614* | *0.96724* | *0.98286* | *0.96311* | *0.95336* | *0.96233* | *0.95972* | *0.95374* | *0.96348* | *0.95768* | *0.95189* | *0.93338* | *0.93256* | *0.93595* | *0.93585* | *0.94366* | *0.92827* | *0.91357* | *0.92296* | *0.91211* |
| *34500.62163* | *0.96952* | *0.98502* | *0.96541* | *0.95577* | *0.9647* | *0.96216* | *0.95638* | *0.96588* | *0.96023* | *0.95432* | *0.93603* | *0.93536* | *0.93876* | *0.93863* | *0.94642* | *0.93115* | *0.91649* | *0.92593* | *0.91511* |
| *34491.09239* | *0.97172* | *0.98702* | *0.96764* | *0.95812* | *0.96698* | *0.96452* | *0.95895* | *0.9682* | *0.96269* | *0.95668* | *0.93863* | *0.93809* | *0.94151* | *0.94134* | *0.94912* | *0.93398* | *0.91936* | *0.92884* | *0.91806* |
| *34481.56841* | *0.97384* | *0.98885* | *0.96978* | *0.9604* | *0.96919* | *0.9668* | *0.96143* | *0.97042* | *0.96506* | *0.95897* | *0.94118* | *0.94077* | *0.94419* | *0.94399* | *0.95174* | *0.93675* | *0.92218* | *0.93168* | *0.92097* |
| *34472.04969* | *0.97587* | *0.99053* | *0.97185* | *0.96262* | *0.97131* | *0.96899* | *0.96383* | *0.97255* | *0.96735* | *0.9612* | *0.94367* | *0.9434* | *0.94681* | *0.94658* | *0.95429* | *0.93947* | *0.92496* | *0.93447* | *0.92382* |
| *34462.53622* | *0.97781* | *0.99205* | *0.97384* | *0.96477* | *0.97336* | *0.97111* | *0.96615* | *0.97458* | *0.96955* | *0.96335* | *0.94612* | *0.94596* | *0.94937* | *0.9491* | *0.95676* | *0.94212* | *0.92768* | *0.9372* | *0.92662* |
| *34453.028* | *0.97967* | *0.99342* | *0.97576* | *0.96686* | *0.97532* | *0.97315* | *0.96839* | *0.97653* | *0.97167* | *0.96543* | *0.94851* | *0.94847* | *0.95186* | *0.95156* | *0.95917* | *0.94471* | *0.93036* | *0.93987* | *0.92937* |
| *34443.52503* | *0.98144* | *0.99464* | *0.97759* | *0.96888* | *0.9772* | *0.9751* | *0.97055* | *0.97838* | *0.9737* | *0.96745* | *0.95084* | *0.95091* | *0.95429* | *0.95396* | *0.9615* | *0.94724* | *0.93299* | *0.94248* | *0.93207* |
| *34434.0273* | *0.98313* | *0.99572* | *0.97935* | *0.97084* | *0.97901* | *0.97698* | *0.97262* | *0.98014* | *0.97564* | *0.9694* | *0.95312* | *0.9533* | *0.95666* | *0.95629* | *0.96375* | *0.94971* | *0.93557* | *0.94503* | *0.93471* |
| *34424.5348* | *0.98473* | *0.99666* | *0.98103* | *0.97273* | *0.98073* | *0.97878* | *0.97461* | *0.98182* | *0.97751* | *0.97128* | *0.95535* | *0.95563* | *0.95896* | *0.95856* | *0.96593* | *0.95212* | *0.9381* | *0.94752* | *0.93731* |
| *34415.04754* | *0.98625* | *0.99747* | *0.98264* | *0.97456* | *0.98237* | *0.9805* | *0.97652* | *0.98341* | *0.97928* | *0.97309* | *0.95753* | *0.9579* | *0.9612* | *0.96076* | *0.96804* | *0.95447* | *0.94058* | *0.94995* | *0.93985* |
| *34405.5655* | *0.98769* | *0.99816* | *0.98416* | *0.97632* | *0.98394* | *0.98213* | *0.97835* | *0.98492* | *0.98098* | *0.97484* | *0.95965* | *0.96011* | *0.96337* | *0.9629* | *0.97008* | *0.95676* | *0.94302* | *0.95231* | *0.94235* |
| *34396.08869* | *0.98904* | *0.99872* | *0.98562* | *0.97802* | *0.98543* | *0.9837* | *0.9801* | *0.98634* | *0.98259* | *0.97652* | *0.96172* | *0.96226* | *0.96547* | *0.96497* | *0.97204* | *0.95899* | *0.9454* | *0.95462* | *0.94479* |
| *34386.6171* | *0.99031* | *0.99918* | *0.98699* | *0.97965* | *0.98684* | *0.98518* | *0.98176* | *0.98767* | *0.98412* | *0.97813* | *0.96373* | *0.96434* | *0.96751* | *0.96699* | *0.97393* | *0.96116* | *0.94773* | *0.95686* | *0.94717* |
| *34377.15072* | *0.9915* | *0.99952* | *0.9883* | *0.98121* | *0.98817* | *0.98659* | *0.98335* | *0.98893* | *0.98557* | *0.97968* | *0.96569* | *0.96637* | *0.96949* | *0.96893* | *0.97575* | *0.96326* | *0.95002* | *0.95905* | *0.94951* |
| *34367.68956* | *0.9926* | *0.99977* | *0.98953* | *0.98271* | *0.98942* | *0.98792* | *0.98485* | *0.99011* | *0.98694* | *0.98117* | *0.96759* | *0.96834* | *0.9714* | *0.97082* | *0.9775* | *0.96531* | *0.95225* | *0.96117* | *0.95179* |
| *34358.23359* | *0.99363* | *0.99993* | *0.99068* | *0.98415* | *0.9906* | *0.98917* | *0.98628* | *0.99121* | *0.98824* | *0.98259* | *0.96944* | *0.97025* | *0.97325* | *0.97264* | *0.97917* | *0.96729* | *0.95443* | *0.96324* | *0.95402* |
| *34348.78284* | *0.99457* | *1* | *0.99177* | *0.98552* | *0.99171* | *0.99035* | *0.98763* | *0.99223* | *0.98945* | *0.98395* | *0.97123* | *0.97209* | *0.97503* | *0.9744* | *0.98077* | *0.96921* | *0.95656* | *0.96524* | *0.95619* |
| *34339.33727* | *0.99543* | *0.99999* | *0.99278* | *0.98683* | *0.99274* | *0.99146* | *0.98891* | *0.99318* | *0.99059* | *0.98525* | *0.97297* | *0.97388* | *0.97675* | *0.97609* | *0.98231* | *0.97108* | *0.95864* | *0.96718* | *0.95832* |
| *34329.89691* | *0.99622* | *0.99992* | *0.99372* | *0.98807* | *0.9937* | *0.99249* | *0.9901* | *0.99406* | *0.99166* | *0.98648* | *0.97466* | *0.9756* | *0.9784* | *0.97772* | *0.98377* | *0.97288* | *0.96067* | *0.96906* | *0.96038* |
| *34320.46173* | *0.99693* | *0.99978* | *0.99459* | *0.98925* | *0.99458* | *0.99345* | *0.99122* | *0.99487* | *0.99265* | *0.98766* | *0.97628* | *0.97727* | *0.97999* | *0.97929* | *0.98516* | *0.97461* | *0.96265* | *0.97089* | *0.9624* |
| *34311.03174* | *0.99756* | *0.99958* | *0.99539* | *0.99036* | *0.9954* | *0.99434* | *0.99227* | *0.99561* | *0.99358* | *0.98877* | *0.97786* | *0.97887* | *0.98151* | *0.9808* | *0.98649* | *0.97629* | *0.96457* | *0.97265* | *0.96436* |
| *34301.60692* | *0.99812* | *0.99933* | *0.99613* | *0.99142* | *0.99614* | *0.99516* | *0.99325* | *0.99629* | *0.99443* | *0.98983* | *0.97938* | *0.98041* | *0.98297* | *0.98224* | *0.98775* | *0.97791* | *0.96645* | *0.97435* | *0.96627* |
| *34292.18729* | *0.9986* | *0.99903* | *0.99679* | *0.9924* | *0.99682* | *0.99591* | *0.99415* | *0.9969* | *0.99522* | *0.99082* | *0.98084* | *0.9819* | *0.98437* | *0.98363* | *0.98894* | *0.97947* | *0.96827* | *0.976* | *0.96812* |
| *34282.77282* | *0.99901* | *0.9987* | *0.9974* | *0.99333* | *0.99742* | *0.9966* | *0.99498* | *0.99745* | *0.99594* | *0.99176* | *0.98225* | *0.98332* | *0.9857* | *0.98495* | *0.99006* | *0.98096* | *0.97004* | *0.97758* | *0.96992* |
| *34273.36352* | *0.99935* | *0.99834* | *0.99793* | *0.99419* | *0.99796* | *0.99721* | *0.99575* | *0.99794* | *0.99659* | *0.99265* | *0.9836* | *0.98468* | *0.98697* | *0.98622* | *0.99112* | *0.9824* | *0.97176* | *0.97911* | *0.97167* |
| *34263.95939* | *0.99961* | *0.99794* | *0.9984* | *0.99499* | *0.99844* | *0.99777* | *0.99644* | *0.99837* | *0.99718* | *0.99348* | *0.9849* | *0.98598* | *0.98818* | *0.98742* | *0.99211* | *0.98378* | *0.97343* | *0.98057* | *0.97336* |
| *34254.56042* | *0.99981* | *0.99752* | *0.99881* | *0.99573* | *0.99884* | *0.99825* | *0.99707* | *0.99875* | *0.99771* | *0.99425* | *0.98615* | *0.98722* | *0.98933* | *0.98856* | *0.99304* | *0.9851* | *0.97505* | *0.98199* | *0.975* |
| *34245.1666* | *0.99994* | *0.99709* | *0.99916* | *0.99641* | *0.99919* | *0.99868* | *0.99763* | *0.99907* | *0.99818* | *0.99497* | *0.98734* | *0.98841* | *0.99041* | *0.98965* | *0.9939* | *0.98635* | *0.97661* | *0.98334* | *0.97659* |
| *34235.77793* | *1* | *0.99664* | *0.99944* | *0.99703* | *0.99947* | *0.99904* | *0.99813* | *0.99934* | *0.99859* | *0.99564* | *0.98847* | *0.98953* | *0.99143* | *0.99068* | *0.99471* | *0.98755* | *0.97812* | *0.98463* | *0.97812* |
| *34226.39441* | *1* | *0.99618* | *0.99967* | *0.99759* | *0.99969* | *0.99934* | *0.99857* | *0.99956* | *0.99895* | *0.99625* | *0.98955* | *0.99059* | *0.9924* | *0.99165* | *0.99545* | *0.9887* | *0.97958* | *0.98587* | *0.9796* |
| *34217.01603* | *0.99993* | *0.99571* | *0.99984* | *0.99809* | *0.99985* | *0.99959* | *0.99895* | *0.99974* | *0.99925* | *0.99682* | *0.99058* | *0.9916* | *0.9933* | *0.99256* | *0.99613* | *0.98978* | *0.98099* | *0.98706* | *0.98102* |
| *34207.64279* | *0.99979* | *0.99523* | *0.99995* | *0.99853* | *0.99996* | *0.99977* | *0.99926* | *0.99987* | *0.9995* | *0.99734* | *0.99155* | *0.99254* | *0.99414* | *0.99341* | *0.99675* | *0.9908* | *0.98235* | *0.98818* | *0.98239* |
| *34198.27468* | *0.9996* | *0.99476* | *1* | *0.99891* | *1* | *0.9999* | *0.99952* | *0.99995* | *0.99969* | *0.9978* | *0.99246* | *0.99343* | *0.99492* | *0.99421* | *0.99732* | *0.99177* | *0.98366* | *0.98925* | *0.98371* |
| *34188.9117* | *0.99935* | *0.99428* | *1* | *0.99923* | *0.99999* | *0.99998* | *0.99972* | *1* | *0.99984* | *0.99822* | *0.99333* | *0.99426* | *0.99565* | *0.99496* | *0.99783* | *0.99268* | *0.98491* | *0.99027* | *0.98498* |
| *34179.55385* | *0.99904* | *0.9938* | *0.99994* | *0.9995* | *0.99992* | *1* | *0.99987* | *1* | *0.99994* | *0.99859* | *0.99414* | *0.99503* | *0.99631* | *0.99565* | *0.99828* | *0.99354* | *0.98612* | *0.99123* | *0.98619* |
| *34170.20112* | *0.99867* | *0.99333* | *0.99984* | *0.99971* | *0.9998* | *0.99997* | *0.99996* | *0.99997* | *0.99999* | *0.99892* | *0.99489* | *0.99574* | *0.99692* | *0.99629* | *0.99867* | *0.99434* | *0.98727* | *0.99214* | *0.98735* |
| *34160.85351* | *0.99824* | *0.99286* | *0.99968* | *0.99986* | *0.99963* | *0.99989* | *1* | *0.99989* | *1* | *0.9992* | *0.9956* | *0.9964* | *0.99747* | *0.99687* | *0.99902* | *0.99508* | *0.98837* | *0.993* | *0.98846* |
| *34151.51101* | *0.99776* | *0.99239* | *0.99947* | *0.99996* | *0.9994* | *0.99975* | *0.99999* | *0.99979* | *0.99997* | *0.99944* | *0.99625* | *0.997* | *0.99797* | *0.9974* | *0.9993* | *0.99577* | *0.98942* | *0.9938* | *0.98951* |
| *34142.17362* | *0.99723* | *0.99192* | *0.99921* | *1* | *0.99913* | *0.99957* | *0.99993* | *0.99965* | *0.99989* | *0.99963* | *0.99684* | *0.99755* | *0.99841* | *0.99788* | *0.99954* | *0.99641* | *0.99042* | *0.99455* | *0.99051* |
| *34132.84133* | *0.99664* | *0.99146* | *0.99891* | *0.99999* | *0.99881* | *0.99935* | *0.99982* | *0.99947* | *0.99977* | *0.99979* | *0.99739* | *0.99803* | *0.99879* | *0.99831* | *0.99973* | *0.99699* | *0.99137* | *0.99525* | *0.99146* |
| *34123.51414* | *0.99601* | *0.991* | *0.99855* | *0.99992* | *0.99844* | *0.99907* | *0.99966* | *0.99927* | *0.99962* | *0.9999* | *0.99788* | *0.99847* | *0.99912* | *0.99869* | *0.99987* | *0.99752* | *0.99226* | *0.9959* | *0.99236* |
| *34114.19205* | *0.99532* | *0.99054* | *0.99815* | *0.9998* | *0.99802* | *0.99875* | *0.99946* | *0.99904* | *0.99943* | *0.99997* | *0.99832* | *0.99885* | *0.9994* | *0.99902* | *0.99996* | *0.99799* | *0.99311* | *0.9965* | *0.99321* |
| *34104.87505* | *0.99459* | *0.99009* | *0.99771* | *0.99963* | *0.99756* | *0.99839* | *0.99922* | *0.99878* | *0.9992* | *1* | *0.99871* | *0.99917* | *0.99962* | *0.9993* | *1* | *0.99841* | *0.99391* | *0.99705* | *0.994* |
| *34095.56314* | *0.99382* | *0.98963* | *0.99723* | *0.99941* | *0.99706* | *0.99799* | *0.99893* | *0.99849* | *0.99894* | *0.99999* | *0.99904* | *0.99944* | *0.99979* | *0.99953* | *1* | *0.99879* | *0.99465* | *0.99755* | *0.99475* |
| *34086.25631* | *0.993* | *0.98918* | *0.9967* | *0.99914* | *0.99651* | *0.99755* | *0.9986* | *0.99817* | *0.99864* | *0.99995* | *0.99933* | *0.99966* | *0.99991* | *0.99972* | *0.99995* | *0.99911* | *0.99535* | *0.998* | *0.99544* |
| *34076.95456* | *0.99213* | *0.98872* | *0.99613* | *0.99881* | *0.99592* | *0.99706* | *0.99824* | *0.99784* | *0.99831* | *0.99987* | *0.99956* | *0.99982* | *0.99998* | *0.99985* | *0.99986* | *0.99938* | *0.996* | *0.99841* | *0.99608* |
| *34067.65789* | *0.99122* | *0.98826* | *0.99552* | *0.99844* | *0.9953* | *0.99654* | *0.99783* | *0.99747* | *0.99796* | *0.99975* | *0.99975* | *0.99993* | *1* | *0.99995* | *0.99972* | *0.9996* | *0.99659* | *0.99876* | *0.99668* |
| *34058.36629* | *0.99028* | *0.98779* | *0.99487* | *0.99802* | *0.99463* | *0.99598* | *0.99738* | *0.99709* | *0.99757* | *0.99959* | *0.99988* | *0.99999* | *0.99997* | *1* | *0.99954* | *0.99977* | *0.99714* | *0.99907* | *0.99722* |
| *34049.07975* | *0.98929* | *0.98732* | *0.99419* | *0.99755* | *0.99392* | *0.99539* | *0.9969* | *0.99668* | *0.99715* | *0.99941* | *0.99997* | *1* | *0.99989* | *1* | *0.99933* | *0.99989* | *0.99764* | *0.99934* | *0.99771* |
| *34039.79828* | *0.98827* | *0.98683* | *0.99346* | *0.99703* | *0.99318* | *0.99476* | *0.99639* | *0.99625* | *0.99671* | *0.99918* | *1* | *0.99996* | *0.99977* | *0.99996* | *0.99907* | *0.99997* | *0.99809* | *0.99956* | *0.99816* |
| *34030.52187* | *0.98721* | *0.98634* | *0.99271* | *0.99646* | *0.99241* | *0.9941* | *0.99584* | *0.9958* | *0.99624* | *0.99893* | *0.99999* | *0.99987* | *0.99959* | *0.99988* | *0.99877* | *1* | *0.99849* | *0.99973* | *0.99855* |
| *34021.25051* | *0.98611* | *0.98583* | *0.99191* | *0.99585* | *0.9916* | *0.9934* | *0.99525* | *0.99532* | *0.99574* | *0.99864* | *0.99992* | *0.99972* | *0.99938* | *0.99975* | *0.99844* | *0.99998* | *0.99885* | *0.99986* | *0.9989* |
| *34011.9842* | *0.98498* | *0.98531* | *0.99108* | *0.9952* | *0.99075* | *0.99267* | *0.99464* | *0.99483* | *0.99522* | *0.99832* | *0.99981* | *0.99953* | *0.99911* | *0.99959* | *0.99807* | *0.99992* | *0.99915* | *0.99995* | *0.9992* |
| *34002.72294* | *0.98382* | *0.98478* | *0.99022* | *0.9945* | *0.98987* | *0.99191* | *0.99399* | *0.99432* | *0.99468* | *0.99797* | *0.99965* | *0.99929* | *0.9988* | *0.99938* | *0.99767* | *0.99981* | *0.99941* | *1* | *0.99945* |
| *33993.46672* | *0.98262* | *0.98423* | *0.98933* | *0.99375* | *0.98896* | *0.99112* | *0.99332* | *0.99379* | *0.99411* | *0.99759* | *0.99944* | *0.99901* | *0.99845* | *0.99914* | *0.99723* | *0.99966* | *0.99962* | *1* | *0.99965* |
| *33984.21554* | *0.9814* | *0.98366* | *0.98841* | *0.99297* | *0.98802* | *0.99031* | *0.99261* | *0.99324* | *0.99352* | *0.99718* | *0.99919* | *0.99867* | *0.99806* | *0.99886* | *0.99676* | *0.99946* | *0.99979* | *0.99996* | *0.99981* |
| *33974.96939* | *0.98014* | *0.98306* | *0.98745* | *0.99214* | *0.98705* | *0.98946* | *0.99188* | *0.99267* | *0.99291* | *0.99674* | *0.99889* | *0.99829* | *0.99762* | *0.99853* | *0.99626* | *0.99922* | *0.9999* | *0.99988* | *0.99992* |
| *33965.72827* | *0.97886* | *0.98245* | *0.98647* | *0.99127* | *0.98606* | *0.98859* | *0.99112* | *0.99208* | *0.99227* | *0.99627* | *0.99854* | *0.99787* | *0.99714* | *0.99817* | *0.99572* | *0.99894* | *0.99997* | *0.99976* | *0.99998* |
| *33956.49218* | *0.97755* | *0.98181* | *0.98545* | *0.99035* | *0.98503* | *0.98769* | *0.99033* | *0.99148* | *0.99162* | *0.99577* | *0.99815* | *0.9974* | *0.99662* | *0.99778* | *0.99516* | *0.99862* | *1* | *0.99961* | *1* |
| *33947.26111* | *0.97621* | *0.98114* | *0.98441* | *0.9894* | *0.98398* | *0.98676* | *0.98951* | *0.99085* | *0.99094* | *0.99524* | *0.99771* | *0.99688* | *0.99606* | *0.99735* | *0.99456* | *0.99826* | *0.99998* | *0.99941* | *0.99997* |
| *33938.03506* | *0.97484* | *0.98045* | *0.98334* | *0.98841* | *0.98289* | *0.98581* | *0.98867* | *0.99021* | *0.99025* | *0.99469* | *0.99723* | *0.99632* | *0.99546* | *0.99688* | *0.99394* | *0.99785* | *0.99991* | *0.99917* | *0.9999* |
| *33928.81402* | *0.97346* | *0.97973* | *0.98225* | *0.98738* | *0.98179* | *0.98484* | *0.98781* | *0.98955* | *0.98953* | *0.99411* | *0.9967* | *0.99572* | *0.99483* | *0.99638* | *0.99329* | *0.99741* | *0.9998* | *0.9989* | *0.99978* |
| *33919.59799* | *0.97204* | *0.97899* | *0.98113* | *0.98631* | *0.98066* | *0.98384* | *0.98692* | *0.98887* | *0.9888* | *0.99351* | *0.99613* | *0.99508* | *0.99415* | *0.99584* | *0.99261* | *0.99693* | *0.99965* | *0.99859* | *0.99962* |
| *33910.38697* | *0.97061* | *0.97821* | *0.97998* | *0.98521* | *0.9795* | *0.98282* | *0.986* | *0.98817* | *0.98804* | *0.99287* | *0.99551* | *0.99439* | *0.99344* | *0.99527* | *0.99191* | *0.99641* | *0.99945* | *0.99825* | *0.99942* |
| *33901.18094* | *0.96915* | *0.9774* | *0.97881* | *0.98406* | *0.97832* | *0.98178* | *0.98507* | *0.98745* | *0.98727* | *0.99221* | *0.99486* | *0.99366* | *0.99269* | *0.99467* | *0.99118* | *0.99585* | *0.99921* | *0.99786* | *0.99917* |
| *33891.97992* | *0.96767* | *0.97656* | *0.97761* | *0.98288* | *0.97712* | *0.98071* | *0.98411* | *0.98671* | *0.98648* | *0.99153* | *0.99415* | *0.99289* | *0.99191* | *0.99404* | *0.99042* | *0.99526* | *0.99892* | *0.99745* | *0.99888* |
| *33882.78388* | *0.96617* | *0.97568* | *0.97639* | *0.98167* | *0.9759* | *0.97963* | *0.98312* | *0.98596* | *0.98567* | *0.99082* | *0.99341* | *0.99208* | *0.9911* | *0.99337* | *0.98964* | *0.99462* | *0.99859* | *0.997* | *0.99854* |
| *33873.59284* | *0.96465* | *0.97478* | *0.97514* | *0.98042* | *0.97465* | *0.97852* | *0.98212* | *0.98518* | *0.98484* | *0.99009* | *0.99263* | *0.99124* | *0.99025* | *0.99267* | *0.98884* | *0.99396* | *0.99822* | *0.99651* | *0.99817* |
| *33864.40678* | *0.96311* | *0.97383* | *0.97388* | *0.97914* | *0.97338* | *0.97739* | *0.98109* | *0.98439* | *0.98399* | *0.98933* | *0.9918* | *0.99035* | *0.98936* | *0.99195* | *0.98802* | *0.99326* | *0.9978* | *0.99599* | *0.99775* |
| *33855.2257* | *0.96155* | *0.97286* | *0.97259* | *0.97782* | *0.97209* | *0.97624* | *0.98004* | *0.98357* | *0.98312* | *0.98855* | *0.99094* | *0.98943* | *0.98845* | *0.99119* | *0.98717* | *0.99252* | *0.99735* | *0.99544* | *0.9973* |
| *33846.0496* | *0.95998* | *0.97184* | *0.97127* | *0.97647* | *0.97078* | *0.97507* | *0.97897* | *0.98274* | *0.98224* | *0.98774* | *0.99003* | *0.98847* | *0.9875* | *0.99041* | *0.98631* | *0.99175* | *0.99685* | *0.99486* | *0.9968* |
| *33836.87847* | *0.95838* | *0.9708* | *0.96994* | *0.97509* | *0.96945* | *0.97389* | *0.97788* | *0.98188* | *0.98134* | *0.98691* | *0.98909* | *0.98747* | *0.98652* | *0.98959* | *0.98542* | *0.99095* | *0.99631* | *0.99424* | *0.99626* |
| *33827.71231* | *0.95677* | *0.96971* | *0.96858* | *0.97368* | *0.9681* | *0.97268* | *0.97677* | *0.981* | *0.98041* | *0.98605* | *0.9881* | *0.98643* | *0.98551* | *0.98875* | *0.98451* | *0.99012* | *0.99574* | *0.99359* | *0.99569* |
| *33818.55112* | *0.95514* | *0.96859* | *0.96721* | *0.97224* | *0.96673* | *0.97146* | *0.97564* | *0.9801* | *0.97947* | *0.98518* | *0.98708* | *0.98537* | *0.98447* | *0.98788* | *0.98358* | *0.98925* | *0.99512* | *0.99292* | *0.99507* |
| *33809.39488* | *0.9535* | *0.96743* | *0.96581* | *0.97077* | *0.96534* | *0.97021* | *0.97449* | *0.97918* | *0.97851* | *0.98428* | *0.98602* | *0.98426* | *0.98341* | *0.98699* | *0.98263* | *0.98836* | *0.99446* | *0.99221* | *0.99442* |
| *33800.24361* | *0.95184* | *0.96624* | *0.96439* | *0.96927* | *0.96394* | *0.96895* | *0.97331* | *0.97824* | *0.97753* | *0.98335* | *0.98492* | *0.98313* | *0.98231* | *0.98606* | *0.98167* | *0.98743* | *0.99376* | *0.99147* | *0.99373* |
| *33791.09728* | *0.95016* | *0.96501* | *0.96295* | *0.96773* | *0.96251* | *0.96767* | *0.97212* | *0.97727* | *0.97653* | *0.9824* | *0.98379* | *0.98195* | *0.98119* | *0.98511* | *0.98068* | *0.98647* | *0.99303* | *0.9907* | *0.993* |
| *33781.9559* | *0.94847* | *0.96374* | *0.9615* | *0.96617* | *0.96107* | *0.96637* | *0.97091* | *0.97628* | *0.97551* | *0.98143* | *0.98261* | *0.98075* | *0.98004* | *0.98414* | *0.97968* | *0.98549* | *0.99225* | *0.98991* | *0.99223* |
| *33772.81947* | *0.94676* | *0.96243* | *0.96002* | *0.96459* | *0.9596* | *0.96506* | *0.96967* | *0.97527* | *0.97448* | *0.98044* | *0.98141* | *0.97952* | *0.97886* | *0.98314* | *0.97866* | *0.98447* | *0.99144* | *0.98908* | *0.99143* |
| *33763.68798* | *0.94504* | *0.96109* | *0.95852* | *0.96297* | *0.95812* | *0.96372* | *0.96842* | *0.97424* | *0.97342* | *0.97942* | *0.98016* | *0.97825* | *0.97766* | *0.98211* | *0.97762* | *0.98343* | *0.99059* | *0.98823* | *0.99059* |
| *33754.56143* | *0.94331* | *0.95971* | *0.957* | *0.96133* | *0.95663* | *0.96237* | *0.96714* | *0.97318* | *0.97234* | *0.97838* | *0.97889* | *0.97695* | *0.97643* | *0.98107* | *0.97657* | *0.98236* | *0.98971* | *0.98735* | *0.98972* |
| *33745.43981* | *0.94156* | *0.9583* | *0.95547* | *0.95967* | *0.95511* | *0.96101* | *0.96585* | *0.97209* | *0.97124* | *0.97732* | *0.97758* | *0.97563* | *0.97517* | *0.97999* | *0.9755* | *0.98126* | *0.98879* | *0.98644* | *0.98881* |
| *33736.32311* | *0.93979* | *0.95685* | *0.95391* | *0.95797* | *0.95358* | *0.95962* | *0.96453* | *0.97099* | *0.97012* | *0.97624* | *0.97623* | *0.97427* | *0.9739* | *0.9789* | *0.97441* | *0.98014* | *0.98783* | *0.98551* | *0.98786* |
| *33727.21134* | *0.93802* | *0.95536* | *0.95234* | *0.95626* | *0.95203* | *0.95822* | *0.96319* | *0.96985* | *0.96899* | *0.97513* | *0.97485* | *0.97289* | *0.97259* | *0.97778* | *0.97331* | *0.97899* | *0.98684* | *0.98455* | *0.98689* |
| *33718.1045* | *0.93623* | *0.95383* | *0.95075* | *0.95451* | *0.95046* | *0.9568* | *0.96184* | *0.96869* | *0.96782* | *0.974* | *0.97344* | *0.97147* | *0.97127* | *0.97663* | *0.97219* | *0.97782* | *0.98581* | *0.98357* | *0.98588* |
| *33709.00256* | *0.93442* | *0.95227* | *0.94913* | *0.95275* | *0.94888* | *0.95536* | *0.96046* | *0.96751* | *0.96664* | *0.97285* | *0.972* | *0.97003* | *0.96992* | *0.97547* | *0.97106* | *0.97662* | *0.98474* | *0.98255* | *0.98483* |
| *33699.90555* | *0.93261* | *0.95068* | *0.9475* | *0.95096* | *0.94728* | *0.9539* | *0.95906* | *0.9663* | *0.96544* | *0.97168* | *0.97052* | *0.96856* | *0.96855* | *0.97428* | *0.96991* | *0.9754* | *0.98365* | *0.98152* | *0.98376* |
| *33690.81344* | *0.93078* | *0.94905* | *0.94585* | *0.94915* | *0.94566* | *0.95243* | *0.95764* | *0.96506* | *0.96422* | *0.97048* | *0.96902* | *0.96707* | *0.96716* | *0.97307* | *0.96874* | *0.97415* | *0.98252* | *0.98046* | *0.98265* |
| *33681.72623* | *0.92893* | *0.94738* | *0.94419* | *0.94731* | *0.94403* | *0.95094* | *0.9562* | *0.9638* | *0.96297* | *0.96926* | *0.96748* | *0.96555* | *0.96575* | *0.97184* | *0.96756* | *0.97288* | *0.98135* | *0.97937* | *0.98151* |
| *33672.64393* | *0.92708* | *0.94568* | *0.9425* | *0.94545* | *0.94238* | *0.94943* | *0.95474* | *0.96251* | *0.9617* | *0.96802* | *0.96591* | *0.964* | *0.96431* | *0.97059* | *0.96637* | *0.97159* | *0.98016* | *0.97826* | *0.98034* |
| *33663.56652* | *0.92521* | *0.94394* | *0.9408* | *0.94357* | *0.94071* | *0.94791* | *0.95325* | *0.96119* | *0.96041* | *0.96675* | *0.96432* | *0.96243* | *0.96286* | *0.96932* | *0.96516* | *0.97027* | *0.97893* | *0.97713* | *0.97914* |
| *33654.494* | *0.92333* | *0.94217* | *0.93907* | *0.94167* | *0.93903* | *0.94636* | *0.95175* | *0.95985* | *0.95909* | *0.96546* | *0.96269* | *0.96084* | *0.96139* | *0.96802* | *0.96393* | *0.96893* | *0.97767* | *0.97597* | *0.97791* |
| *33645.42638* | *0.92143* | *0.94037* | *0.93733* | *0.93975* | *0.93733* | *0.9448* | *0.95023* | *0.95848* | *0.95776* | *0.96415* | *0.96104* | *0.95922* | *0.95989* | *0.96671* | *0.9627* | *0.96757* | *0.97638* | *0.97479* | *0.97666* |
| *33636.36364* | *0.91952* | *0.93854* | *0.93557* | *0.93781* | *0.93561* | *0.94322* | *0.94868* | *0.95708* | *0.9564* | *0.96282* | *0.95936* | *0.95758* | *0.95838* | *0.96537* | *0.96144* | *0.96619* | *0.97506* | *0.97359* | *0.97537* |
| *33627.30578* | *0.9176* | *0.93667* | *0.93379* | *0.93585* | *0.93388* | *0.94163* | *0.94711* | *0.95565* | *0.95501* | *0.96147* | *0.95765* | *0.95592* | *0.95685* | *0.96402* | *0.96017* | *0.96479* | *0.97371* | *0.97236* | *0.97405* |
| *33618.25279* | *0.91567* | *0.93477* | *0.93199* | *0.93387* | *0.93212* | *0.94001* | *0.94552* | *0.9542* | *0.95361* | *0.96009* | *0.95591* | *0.95423* | *0.9553* | *0.96264* | *0.95889* | *0.96337* | *0.97233* | *0.97111* | *0.97271* |
| *33609.20468* | *0.91372* | *0.93284* | *0.93018* | *0.93187* | *0.93036* | *0.93838* | *0.94391* | *0.95272* | *0.95218* | *0.95869* | *0.95415* | *0.95253* | *0.95373* | *0.96124* | *0.95759* | *0.96193* | *0.97092* | *0.96984* | *0.97134* |
| *33600.16144* | *0.91176* | *0.93087* | *0.92834* | *0.92985* | *0.92857* | *0.93673* | *0.94228* | *0.95121* | *0.95072* | *0.95726* | *0.95236* | *0.9508* | *0.95214* | *0.95983* | *0.95628* | *0.96047* | *0.96948* | *0.96855* | *0.96994* |
| *33591.12307* | *0.90979* | *0.92888* | *0.92649* | *0.92781* | *0.92677* | *0.93506* | *0.94062* | *0.94967* | *0.94924* | *0.95582* | *0.95055* | *0.94905* | *0.95054* | *0.95839* | *0.95495* | *0.95898* | *0.96801* | *0.96723* | *0.96851* |
| *33582.08955* | *0.9078* | *0.92685* | *0.92462* | *0.92576* | *0.92495* | *0.93338* | *0.93894* | *0.9481* | *0.94774* | *0.95435* | *0.94871* | *0.94728* | *0.94892* | *0.95694* | *0.95361* | *0.95748* | *0.96651* | *0.9659* | *0.96706* |
| *33573.0609* | *0.9058* | *0.9248* | *0.92273* | *0.92368* | *0.92312* | *0.93167* | *0.93724* | *0.94651* | *0.94621* | *0.95285* | *0.94685* | *0.94549* | *0.94728* | *0.95547* | *0.95226* | *0.95597* | *0.96499* | *0.96454* | *0.96558* |
| *33564.03709* | *0.90379* | *0.92271* | *0.92082* | *0.92159* | *0.92127* | *0.92995* | *0.93552* | *0.94489* | *0.94466* | *0.95134* | *0.94497* | *0.94369* | *0.94562* | *0.95397* | *0.95089* | *0.95443* | *0.96344* | *0.96316* | *0.96408* |
| *33555.01814* | *0.90176* | *0.9206* | *0.9189* | *0.91948* | *0.9194* | *0.9282* | *0.93378* | *0.94324* | *0.94309* | *0.9498* | *0.94306* | *0.94186* | *0.94395* | *0.95246* | *0.9495* | *0.95288* | *0.96187* | *0.96176* | *0.96256* |
| *33546.00403* | *0.89972* | *0.91846* | *0.91695* | *0.91736* | *0.91751* | *0.92644* | *0.93201* | *0.94156* | *0.94149* | *0.94824* | *0.94112* | *0.94002* | *0.94226* | *0.95093* | *0.9481* | *0.9513* | *0.96026* | *0.96035* | *0.96101* |
| *33536.99476* | *0.89767* | *0.91629* | *0.91499* | *0.91522* | *0.91561* | *0.92466* | *0.93022* | *0.93986* | *0.93987* | *0.94665* | *0.93917* | *0.93815* | *0.94056* | *0.94939* | *0.94669* | *0.94971* | *0.95864* | *0.9589* | *0.95943* |
| *33527.99033* | *0.8956* | *0.91409* | *0.91301* | *0.91306* | *0.91369* | *0.92286* | *0.92841* | *0.93813* | *0.93822* | *0.94505* | *0.93719* | *0.93627* | *0.93884* | *0.94782* | *0.94525* | *0.94811* | *0.95698* | *0.95744* | *0.95784* |
| *33518.99074* | *0.89352* | *0.91186* | *0.91101* | *0.91089* | *0.91175* | *0.92105* | *0.92658* | *0.93636* | *0.93654* | *0.94342* | *0.9352* | *0.93438* | *0.93711* | *0.94623* | *0.94381* | *0.94649* | *0.95531* | *0.95596* | *0.95622* |
| *33509.99597* | *0.89143* | *0.90961* | *0.90899* | *0.9087* | *0.90979* | *0.91921* | *0.92472* | *0.93458* | *0.93485* | *0.94176* | *0.93318* | *0.93246* | *0.93536* | *0.94463* | *0.94235* | *0.94485* | *0.95361* | *0.95446* | *0.95457* |
| *33501.00604* | *0.88932* | *0.90733* | *0.90695* | *0.90649* | *0.90782* | *0.91735* | *0.92284* | *0.93276* | *0.93312* | *0.94009* | *0.93114* | *0.93054* | *0.93359* | *0.94301* | *0.94087* | *0.94319* | *0.95188* | *0.95294* | *0.95291* |
| *33492.02092* | *0.8872* | *0.90503* | *0.90489* | *0.90427* | *0.90583* | *0.91548* | *0.92094* | *0.93092* | *0.93138* | *0.93839* | *0.92908* | *0.92859* | *0.93181* | *0.94136* | *0.93938* | *0.94152* | *0.95013* | *0.9514* | *0.95122* |
| *33483.04062* | *0.88506* | *0.9027* | *0.90282* | *0.90204* | *0.90382* | *0.91358* | *0.91902* | *0.92905* | *0.92961* | *0.93667* | *0.927* | *0.92663* | *0.93002* | *0.93971* | *0.93788* | *0.93983* | *0.94836* | *0.94984* | *0.94952* |
| *33474.06514* | *0.88291* | *0.90035* | *0.90072* | *0.89979* | *0.9018* | *0.91167* | *0.91708* | *0.92715* | *0.92781* | *0.93492* | *0.9249* | *0.92465* | *0.92821* | *0.93803* | *0.93635* | *0.93812* | *0.94657* | *0.94826* | *0.94779* |
| *33465.09447* | *0.88075* | *0.89797* | *0.89861* | *0.89752* | *0.89976* | *0.90974* | *0.91511* | *0.92523* | *0.92599* | *0.93316* | *0.92279* | *0.92266* | *0.92638* | *0.93633* | *0.93482* | *0.93641* | *0.94475* | *0.94667* | *0.94604* |
| *33456.1286* | *0.87857* | *0.89557* | *0.89648* | *0.89525* | *0.8977* | *0.90778* | *0.91312* | *0.92328* | *0.92415* | *0.93136* | *0.92065* | *0.92066* | *0.92455* | *0.93462* | *0.93326* | *0.93467* | *0.94291* | *0.94505* | *0.94427* |
| *33447.16754* | *0.87638* | *0.89314* | *0.89433* | *0.89295* | *0.89563* | *0.90581* | *0.91111* | *0.9213* | *0.92228* | *0.92955* | *0.9185* | *0.91864* | *0.9227* | *0.93289* | *0.93169* | *0.93292* | *0.94106* | *0.94341* | *0.94249* |
| *33438.21127* | *0.87417* | *0.89069* | *0.89217* | *0.89065* | *0.89353* | *0.90382* | *0.90907* | *0.9193* | *0.92039* | *0.92772* | *0.91633* | *0.91661* | *0.92083* | *0.93114* | *0.93011* | *0.93116* | *0.93918* | *0.94176* | *0.94068* |
| *33429.2598* | *0.87195* | *0.88822* | *0.88998* | *0.88833* | *0.89142* | *0.90181* | *0.90702* | *0.91727* | *0.91847* | *0.92586* | *0.91414* | *0.91456* | *0.91895* | *0.92938* | *0.92851* | *0.92938* | *0.93728* | *0.94008* | *0.93886* |
| *33420.31313* | *0.86971* | *0.88573* | *0.88778* | *0.886* | *0.88929* | *0.89978* | *0.90494* | *0.91522* | *0.91653* | *0.92398* | *0.91194* | *0.9125* | *0.91706* | *0.92759* | *0.92689* | *0.92759* | *0.93536* | *0.93839* | *0.93701* |
| *33411.37124* | *0.86746* | *0.88321* | *0.88556* | *0.88365* | *0.88715* | *0.89773* | *0.90284* | *0.91314* | *0.91456* | *0.92208* | *0.90972* | *0.91043* | *0.91515* | *0.92579* | *0.92525* | *0.92578* | *0.93342* | *0.93668* | *0.93515* |
| *33402.43413* | *0.8652* | *0.88068* | *0.88332* | *0.88129* | *0.88499* | *0.89566* | *0.90072* | *0.91103* | *0.91258* | *0.92015* | *0.90749* | *0.90835* | *0.91323* | *0.92397* | *0.9236* | *0.92396* | *0.93146* | *0.93495* | *0.93328* |
| *33393.50181* | *0.86292* | *0.87812* | *0.88106* | *0.87892* | *0.88281* | *0.89358* | *0.89858* | *0.9089* | *0.91056* | *0.9182* | *0.90524* | *0.90625* | *0.9113* | *0.92213* | *0.92194* | *0.92212* | *0.92949* | *0.9332* | *0.93138* |
| *33384.57425* | *0.86063* | *0.87555* | *0.87879* | *0.87654* | *0.88061* | *0.89147* | *0.89642* | *0.90675* | *0.90853* | *0.91623* | *0.90297* | *0.90414* | *0.90935* | *0.92028* | *0.92025* | *0.92027* | *0.92749* | *0.93143* | *0.92947* |
| *33375.65148* | *0.85832* | *0.87295* | *0.87649* | *0.87414* | *0.8784* | *0.88934* | *0.89423* | *0.90457* | *0.90647* | *0.91424* | *0.90069* | *0.90202* | *0.90739* | *0.91841* | *0.91855* | *0.91841* | *0.92548* | *0.92965* | *0.92754* |
| *33366.73347* | *0.856* | *0.87034* | *0.87418* | *0.87173* | *0.87617* | *0.8872* | *0.89203* | *0.90237* | *0.90439* | *0.91223* | *0.8984* | *0.89989* | *0.90542* | *0.91652* | *0.91684* | *0.91653* | *0.92345* | *0.92785* | *0.9256* |
| *33357.82022* | *0.85366* | *0.86771* | *0.87186* | *0.86931* | *0.87392* | *0.88503* | *0.8898* | *0.90015* | *0.90229* | *0.91019* | *0.89609* | *0.89774* | *0.90343* | *0.91461* | *0.9151* | *0.91464* | *0.9214* | *0.92602* | *0.92363* |
| *33348.91174* | *0.85131* | *0.86506* | *0.86951* | *0.86688* | *0.87166* | *0.88285* | *0.88755* | *0.8979* | *0.90016* | *0.90814* | *0.89377* | *0.89559* | *0.90143* | *0.91269* | *0.91335* | *0.91273* | *0.91934* | *0.92419* | *0.92166* |
| *33340.00801* | *0.84895* | *0.86239* | *0.86715* | *0.86443* | *0.86938* | *0.88065* | *0.88529* | *0.89563* | *0.89802* | *0.90606* | *0.89144* | *0.89342* | *0.89942* | *0.91075* | *0.91159* | *0.91082* | *0.91726* | *0.92233* | *0.91967* |
| *33331.10904* | *0.84657* | *0.85971* | *0.86477* | *0.86198* | *0.86708* | *0.87843* | *0.883* | *0.89333* | *0.89585* | *0.90396* | *0.8891* | *0.89125* | *0.89739* | *0.90879* | *0.9098* | *0.90888* | *0.91517* | *0.92046* | *0.91766* |
| *33322.21481* | *0.84418* | *0.85701* | *0.86237* | *0.85951* | *0.86477* | *0.87619* | *0.88069* | *0.89102* | *0.89366* | *0.90184* | *0.88674* | *0.88906* | *0.89536* | *0.90682* | *0.908* | *0.90694* | *0.91305* | *0.91856* | *0.91564* |
| *33313.32533* | *0.84177* | *0.85429* | *0.85996* | *0.85703* | *0.86244* | *0.87393* | *0.87837* | *0.88868* | *0.89144* | *0.8997* | *0.88437* | *0.88686* | *0.89331* | *0.90482* | *0.90618* | *0.90498* | *0.91093* | *0.91666* | *0.91361* |
| *33304.44059* | *0.83935* | *0.85156* | *0.85753* | *0.85454* | *0.86009* | *0.87165* | *0.87602* | *0.88632* | *0.88921* | *0.89753* | *0.88199* | *0.88465* | *0.89124* | *0.90282* | *0.90435* | *0.90301* | *0.90879* | *0.91473* | *0.91156* |
| *33295.56059* | *0.83692* | *0.84881* | *0.85508* | *0.85204* | *0.85773* | *0.86936* | *0.87365* | *0.88394* | *0.88695* | *0.89535* | *0.8796* | *0.88244* | *0.88917* | *0.90079* | *0.90249* | *0.90103* | *0.90663* | *0.91279* | *0.9095* |
| *33286.68533* | *0.83447* | *0.84605* | *0.85261* | *0.84953* | *0.85535* | *0.86705* | *0.87127* | *0.88154* | *0.88468* | *0.89315* | *0.87719* | *0.88021* | *0.88708* | *0.89875* | *0.90062* | *0.89903* | *0.90446* | *0.91083* | *0.90743* |
| *33277.81479* | *0.83201* | *0.84327* | *0.85013* | *0.84701* | *0.85296* | *0.86472* | *0.86887* | *0.87912* | *0.88238* | *0.89092* | *0.87478* | *0.87797* | *0.88498* | *0.89669* | *0.89874* | *0.89703* | *0.90228* | *0.90885* | *0.90534* |
| *33268.94898* | *0.82953* | *0.84048* | *0.84764* | *0.84448* | *0.85055* | *0.86237* | *0.86645* | *0.87668* | *0.88007* | *0.88868* | *0.87235* | *0.87573* | *0.88286* | *0.89461* | *0.89683* | *0.89501* | *0.90008* | *0.90685* | *0.90324* |
| *33260.08789* | *0.82704* | *0.83767* | *0.84513* | *0.84193* | *0.84812* | *0.86* | *0.86401* | *0.87421* | *0.87773* | *0.88642* | *0.86992* | *0.87347* | *0.88074* | *0.89252* | *0.89491* | *0.89297* | *0.89787* | *0.90484* | *0.90113* |
| *33251.23153* | *0.82454* | *0.83486* | *0.8426* | *0.83938* | *0.84568* | *0.85762* | *0.86155* | *0.87173* | *0.87538* | *0.88414* | *0.86748* | *0.87121* | *0.8786* | *0.89041* | *0.89297* | *0.89093* | *0.89565* | *0.90281* | *0.899* |
| *33242.37987* | *0.82202* | *0.83203* | *0.84006* | *0.83682* | *0.84323* | *0.85522* | *0.85908* | *0.86924* | *0.87301* | *0.88183* | *0.86502* | *0.86894* | *0.87645* | *0.88828* | *0.89102* | *0.88887* | *0.89341* | *0.90077* | *0.89687* |
| *33233.53293* | *0.81949* | *0.82919* | *0.8375* | *0.83424* | *0.84076* | *0.8528* | *0.85658* | *0.86672* | *0.87061* | *0.87951* | *0.86256* | *0.86666* | *0.87429* | *0.88614* | *0.88904* | *0.8868* | *0.89116* | *0.89871* | *0.89472* |
| *33224.6907* | *0.81695* | *0.82633* | *0.83493* | *0.83166* | *0.83827* | *0.85037* | *0.85408* | *0.86418* | *0.8682* | *0.87717* | *0.86009* | *0.86436* | *0.87212* | *0.88398* | *0.88705* | *0.88471* | *0.8889* | *0.89663* | *0.89256* |
| *33215.85317* | *0.81439* | *0.82347* | *0.83234* | *0.82907* | *0.83578* | *0.84792* | *0.85155* | *0.86163* | *0.86578* | *0.87482* | *0.85761* | *0.86207* | *0.86993* | *0.88181* | *0.88504* | *0.88262* | *0.88663* | *0.89454* | *0.89039* |
| *33207.02034* | *0.81182* | *0.82059* | *0.82974* | *0.82647* | *0.83326* | *0.84545* | *0.84901* | *0.85906* | *0.86333* | *0.87244* | *0.85513* | *0.85976* | *0.86773* | *0.87962* | *0.88302* | *0.88051* | *0.88434* | *0.89243* | *0.88821* |
| *33198.19221* | *0.80924* | *0.81771* | *0.82713* | *0.82385* | *0.83073* | *0.84297* | *0.84645* | *0.85647* | *0.86087* | *0.87004* | *0.85263* | *0.85744* | *0.86552* | *0.87741* | *0.88098* | *0.87839* | *0.88205* | *0.8903* | *0.88602* |
| *33189.36877* | *0.80665* | *0.81482* | *0.8245* | *0.82123* | *0.82819* | *0.84047* | *0.84388* | *0.85387* | *0.85839* | *0.86763* | *0.85013* | *0.85512* | *0.8633* | *0.87519* | *0.87892* | *0.87626* | *0.87974* | *0.88816* | *0.88381* |
| *33180.55002* | *0.80404* | *0.81191* | *0.82185* | *0.8186* | *0.82564* | *0.83795* | *0.84129* | *0.85125* | *0.85589* | *0.8652* | *0.84762* | *0.85279* | *0.86107* | *0.87295* | *0.87684* | *0.87412* | *0.87743* | *0.886* | *0.8816* |
| *33171.73595* | *0.80143* | *0.809* | *0.8192* | *0.81596* | *0.82307* | *0.83542* | *0.83869* | *0.84861* | *0.85337* | *0.86275* | *0.8451* | *0.85045* | *0.85882* | *0.8707* | *0.87475* | *0.87196* | *0.8751* | *0.88382* | *0.87938* |
| *33162.92657* | *0.7988* | *0.80608* | *0.81653* | *0.81332* | *0.82049* | *0.83288* | *0.83607* | *0.84596* | *0.85084* | *0.86029* | *0.84258* | *0.8481* | *0.85656* | *0.86843* | *0.87264* | *0.8698* | *0.87277* | *0.88164* | *0.87715* |
| *33154.12186* | *0.79616* | *0.80315* | *0.81384* | *0.81066* | *0.81789* | *0.83032* | *0.83344* | *0.84329* | *0.8483* | *0.85781* | *0.84005* | *0.84575* | *0.85429* | *0.86615* | *0.87052* | *0.86762* | *0.87042* | *0.87943* | *0.87491* |
| *33145.32183* | *0.7935* | *0.80021* | *0.81115* | *0.80799* | *0.81529* | *0.82774* | *0.83079* | *0.84061* | *0.84574* | *0.85531* | *0.83751* | *0.84338* | *0.85201* | *0.86385* | *0.86837* | *0.86543* | *0.86807* | *0.87721* | *0.87266* |
| *33136.52647* | *0.79084* | *0.79726* | *0.80844* | *0.80532* | *0.81267* | *0.82515* | *0.82813* | *0.83792* | *0.84316* | *0.8528* | *0.83497* | *0.84101* | *0.84972* | *0.86153* | *0.86621* | *0.86323* | *0.8657* | *0.87498* | *0.8704* |
| *33127.73577* | *0.78816* | *0.79431* | *0.80572* | *0.80264* | *0.81003* | *0.82255* | *0.82546* | *0.83521* | *0.84057* | *0.85027* | *0.83242* | *0.83864* | *0.84742* | *0.8592* | *0.86404* | *0.86101* | *0.86333* | *0.87273* | *0.86813* |
| *33118.94974* | *0.78548* | *0.79135* | *0.80299* | *0.79995* | *0.80739* | *0.81993* | *0.82277* | *0.83249* | *0.83796* | *0.84772* | *0.82987* | *0.83625* | *0.8451* | *0.85686* | *0.86185* | *0.85879* | *0.86095* | *0.87046* | *0.86585* |
| *33110.16837* | *0.78278* | *0.78839* | *0.80024* | *0.79725* | *0.80473* | *0.8173* | *0.82007* | *0.82976* | *0.83535* | *0.84516* | *0.82731* | *0.83386* | *0.84278* | *0.8545* | *0.85964* | *0.85655* | *0.85856* | *0.86818* | *0.86357* |
| *33101.39165* | *0.78008* | *0.78542* | *0.79749* | *0.79455* | *0.80207* | *0.81466* | *0.81736* | *0.82701* | *0.83271* | *0.84259* | *0.82474* | *0.83146* | *0.84044* | *0.85213* | *0.85742* | *0.8543* | *0.85616* | *0.86589* | *0.86127* |
| *33092.61958* | *0.77736* | *0.78244* | *0.79472* | *0.79183* | *0.79939* | *0.812* | *0.81464* | *0.82425* | *0.83007* | *0.84* | *0.82217* | *0.82905* | *0.83809* | *0.84974* | *0.85518* | *0.85204* | *0.85375* | *0.86358* | *0.85897* |
| *33083.85217* | *0.77463* | *0.77946* | *0.79195* | *0.78911* | *0.7967* | *0.80933* | *0.8119* | *0.82148* | *0.82741* | *0.8374* | *0.8196* | *0.82664* | *0.83573* | *0.84734* | *0.85292* | *0.84977* | *0.85134* | *0.86126* | *0.85666* |
| *33075.08939* | *0.7719* | *0.77648* | *0.78916* | *0.78638* | *0.794* | *0.80664* | *0.80915* | *0.81869* | *0.82474* | *0.83478* | *0.81702* | *0.82422* | *0.83336* | *0.84492* | *0.85065* | *0.84749* | *0.84891* | *0.85892* | *0.85434* |
| *33066.33126* | *0.76915* | *0.77349* | *0.78636* | *0.78365* | *0.79129* | *0.80395* | *0.8064* | *0.8159* | *0.82205* | *0.83215* | *0.81443* | *0.82179* | *0.83098* | *0.84249* | *0.84837* | *0.8452* | *0.84648* | *0.85657* | *0.85202* |
| *33057.57776* | *0.7664* | *0.77049* | *0.78356* | *0.7809* | *0.78857* | *0.80124* | *0.80363* | *0.8131* | *0.81936* | *0.8295* | *0.81184* | *0.81936* | *0.82858* | *0.84005* | *0.84606* | *0.8429* | *0.84405* | *0.8542* | *0.84968* |
| *33048.8289* | *0.76363* | *0.76749* | *0.78074* | *0.77815* | *0.78584* | *0.79853* | *0.80085* | *0.81028* | *0.81665* | *0.82684* | *0.80925* | *0.81691* | *0.82618* | *0.8376* | *0.84375* | *0.84058* | *0.8416* | *0.85183* | *0.84734* |
| *33040.08467* | *0.76086* | *0.76449* | *0.77791* | *0.7754* | *0.7831* | *0.7958* | *0.79806* | *0.80746* | *0.81393* | *0.82417* | *0.80665* | *0.81447* | *0.82377* | *0.83513* | *0.84142* | *0.83826* | *0.83915* | *0.84943* | *0.845* |
| *33031.34506* | *0.75808* | *0.76149* | *0.77508* | *0.77263* | *0.78035* | *0.79306* | *0.79527* | *0.80462* | *0.8112* | *0.82149* | *0.80405* | *0.81201* | *0.82134* | *0.83264* | *0.83907* | *0.83592* | *0.8367* | *0.84703* | *0.84264* |
| *33022.61008* | *0.75529* | *0.75848* | *0.77224* | *0.76986* | *0.7776* | *0.79031* | *0.79246* | *0.80178* | *0.80846* | *0.81879* | *0.80145* | *0.80955* | *0.81891* | *0.83015* | *0.83671* | *0.83357* | *0.83423* | *0.84461* | *0.84028* |
| *33013.87971* | *0.7525* | *0.75548* | *0.76939* | *0.76709* | *0.77483* | *0.78754* | *0.78964* | *0.79893* | *0.80571* | *0.81609* | *0.79884* | *0.80708* | *0.81646* | *0.82764* | *0.83434* | *0.83122* | *0.83176* | *0.84218* | *0.83791* |
| *33005.15396* | *0.74969* | *0.75247* | *0.76653* | *0.76431* | *0.77206* | *0.78477* | *0.78682* | *0.79607* | *0.80295* | *0.81337* | *0.79623* | *0.80461* | *0.81401* | *0.82512* | *0.83195* | *0.82885* | *0.82929* | *0.83974* | *0.83554* |
| *32996.43282* | *0.74688* | *0.74945* | *0.76366* | *0.76152* | *0.76927* | *0.78199* | *0.78399* | *0.7932* | *0.80018* | *0.81064* | *0.79361* | *0.80213* | *0.81154* | *0.82259* | *0.82954* | *0.82647* | *0.82681* | *0.83728* | *0.83316* |
| *32987.71629* | *0.74406* | *0.74644* | *0.76079* | *0.75872* | *0.76648* | *0.7792* | *0.78115* | *0.79032* | *0.79741* | *0.8079* | *0.791* | *0.79964* | *0.80907* | *0.82004* | *0.82713* | *0.82408* | *0.82432* | *0.83482* | *0.83077* |
| *32979.00436* | *0.74124* | *0.74343* | *0.7579* | *0.75592* | *0.76369* | *0.7764* | *0.7783* | *0.78744* | *0.79462* | *0.80514* | *0.78838* | *0.79715* | *0.80658* | *0.81748* | *0.8247* | *0.82168* | *0.82183* | *0.83234* | *0.82838* |
| *32970.29703* | *0.73841* | *0.74041* | *0.75502* | *0.75312* | *0.76088* | *0.77359* | *0.77544* | *0.78455* | *0.79182* | *0.80238* | *0.78575* | *0.79465* | *0.80409* | *0.81492* | *0.82225* | *0.81927* | *0.81933* | *0.82985* | *0.82598* |
| *32961.5943* | *0.73557* | *0.7374* | *0.75212* | *0.75031* | *0.75807* | *0.77078* | *0.77258* | *0.78165* | *0.78902* | *0.79961* | *0.78313* | *0.79215* | *0.80158* | *0.81234* | *0.8198* | *0.81685* | *0.81683* | *0.82734* | *0.82357* |
| *32952.89616* | *0.73272* | *0.73438* | *0.74922* | *0.74749* | *0.75525* | *0.76795* | *0.76971* | *0.77875* | *0.78621* | *0.79683* | *0.7805* | *0.78964* | *0.79907* | *0.80975* | *0.81733* | *0.81442* | *0.81432* | *0.82483* | *0.82116* |
| *32944.20261* | *0.72987* | *0.73137* | *0.74631* | *0.74467* | *0.75243* | *0.76512* | *0.76684* | *0.77584* | *0.78339* | *0.79404* | *0.77787* | *0.78712* | *0.79655* | *0.80714* | *0.81485* | *0.81198* | *0.81181* | *0.8223* | *0.81874* |
| *32935.51365* | *0.72702* | *0.72836* | *0.7434* | *0.74184* | *0.7496* | *0.76228* | *0.76396* | *0.77292* | *0.78056* | *0.79124* | *0.77524* | *0.7846* | *0.79401* | *0.80453* | *0.81235* | *0.80954* | *0.80929* | *0.81977* | *0.81632* |
| *32926.82927* | *0.72416* | *0.72534* | *0.74048* | *0.73901* | *0.74676* | *0.75943* | *0.76107* | *0.77* | *0.77773* | *0.78843* | *0.7726* | *0.78207* | *0.79147* | *0.80191* | *0.80984* | *0.80708* | *0.80677* | *0.81722* | *0.81389* |
| *32918.14947* | *0.72129* | *0.72233* | *0.73756* | *0.73618* | *0.74392* | *0.75657* | *0.75818* | *0.76707* | *0.77489* | *0.78562* | *0.76997* | *0.77953* | *0.78892* | *0.79928* | *0.80732* | *0.80461* | *0.80424* | *0.81466* | *0.81146* |
| *32909.47424* | *0.71842* | *0.71932* | *0.73463* | *0.73334* | *0.74107* | *0.75371* | *0.75529* | *0.76414* | *0.77205* | *0.78279* | *0.76733* | *0.77699* | *0.78636* | *0.79663* | *0.80479* | *0.80213* | *0.80171* | *0.81209* | *0.80902* |
| *32900.80358* | *0.71555* | *0.71631* | *0.7317* | *0.7305* | *0.73822* | *0.75085* | *0.75238* | *0.76121* | *0.7692* | *0.77996* | *0.76469* | *0.77445* | *0.7838* | *0.79398* | *0.80225* | *0.79965* | *0.79918* | *0.80951* | *0.80657* |
| *32892.1375* | *0.71267* | *0.71331* | *0.72877* | *0.72765* | *0.73537* | *0.74797* | *0.74948* | *0.75827* | *0.76634* | *0.77712* | *0.76205* | *0.7719* | *0.78122* | *0.79132* | *0.7997* | *0.79715* | *0.79664* | *0.80693* | *0.80412* |
| *32883.47597* | *0.70979* | *0.7103* | *0.72583* | *0.7248* | *0.73251* | *0.74509* | *0.74657* | *0.75533* | *0.76348* | *0.77428* | *0.7594* | *0.76934* | *0.77864* | *0.78864* | *0.79713* | *0.79465* | *0.7941* | *0.80433* | *0.80167* |
| *32874.81901* | *0.7069* | *0.7073* | *0.72288* | *0.72194* | *0.72964* | *0.74221* | *0.74366* | *0.75238* | *0.76061* | *0.77142* | *0.75676* | *0.76678* | *0.77604* | *0.78596* | *0.79456* | *0.79213* | *0.79155* | *0.80172* | *0.79921* |
| *32866.1666* | *0.70401* | *0.7043* | *0.71994* | *0.71908* | *0.72677* | *0.73932* | *0.74074* | *0.74943* | *0.75774* | *0.76856* | *0.75411* | *0.76421* | *0.77344* | *0.78327* | *0.79197* | *0.78961* | *0.789* | *0.7991* | *0.79675* |
| *32857.51875* | *0.70112* | *0.70131* | *0.71699* | *0.71622* | *0.7239* | *0.73642* | *0.73782* | *0.74648* | *0.75486* | *0.7657* | *0.75146* | *0.76164* | *0.77083* | *0.78057* | *0.78938* | *0.78708* | *0.78645* | *0.79648* | *0.79428* |
| *32848.87544* | *0.69822* | *0.69831* | *0.71404* | *0.71336* | *0.72103* | *0.73352* | *0.7349* | *0.74353* | *0.75198* | *0.76283* | *0.74882* | *0.75906* | *0.76822* | *0.77787* | *0.78677* | *0.78454* | *0.78389* | *0.79385* | *0.79181* |
| *32840.23669* | *0.69533* | *0.69532* | *0.71108* | *0.71049* | *0.71815* | *0.73062* | *0.73197* | *0.74057* | *0.7491* | *0.75995* | *0.74617* | *0.75648* | *0.7656* | *0.77515* | *0.78416* | *0.78199* | *0.78134* | *0.7912* | *0.78933* |
| *32831.60247* | *0.69243* | *0.69234* | *0.70812* | *0.70762* | *0.71527* | *0.72771* | *0.72905* | *0.73761* | *0.74621* | *0.75707* | *0.74352* | *0.75389* | *0.76297* | *0.77243* | *0.78153* | *0.77944* | *0.77877* | *0.78855* | *0.78685* |
| *32822.9728* | *0.68952* | *0.68936* | *0.70517* | *0.70475* | *0.71239* | *0.7248* | *0.72612* | *0.73465* | *0.74332* | *0.75418* | *0.74086* | *0.7513* | *0.76033* | *0.7697* | *0.7789* | *0.77687* | *0.77621* | *0.78589* | *0.78436* |
| *32814.34765* | *0.68662* | *0.68638* | *0.70221* | *0.70187* | *0.7095* | *0.72188* | *0.72319* | *0.73169* | *0.74043* | *0.75129* | *0.73821* | *0.7487* | *0.75768* | *0.76696* | *0.77625* | *0.7743* | *0.77364* | *0.78323* | *0.78187* |
| *32805.72705* | *0.68372* | *0.68341* | *0.69925* | *0.69899* | *0.70661* | *0.71896* | *0.72025* | *0.72873* | *0.73753* | *0.74839* | *0.73556* | *0.7461* | *0.75503* | *0.76422* | *0.7736* | *0.77172* | *0.77107* | *0.78055* | *0.77938* |
| *32797.11097* | *0.68081* | *0.68044* | *0.69628* | *0.69612* | *0.70373* | *0.71604* | *0.71732* | *0.72577* | *0.73464* | *0.74549* | *0.73291* | *0.74349* | *0.75238* | *0.76146* | *0.77094* | *0.76913* | *0.76849* | *0.77787* | *0.77688* |
| *32788.49941* | *0.6779* | *0.67747* | *0.69332* | *0.69323* | *0.70084* | *0.71312* | *0.71439* | *0.7228* | *0.73174* | *0.74259* | *0.73025* | *0.74088* | *0.74971* | *0.75871* | *0.76827* | *0.76654* | *0.76592* | *0.77518* | *0.77438* |
| *32779.89237* | *0.67499* | *0.67451* | *0.69036* | *0.69035* | *0.69795* | *0.71019* | *0.71145* | *0.71984* | *0.72883* | *0.73968* | *0.7276* | *0.73827* | *0.74704* | *0.75594* | *0.7656* | *0.76394* | *0.76334* | *0.77249* | *0.77187* |
| *32771.28986* | *0.67208* | *0.67156* | *0.68739* | *0.68747* | *0.69505* | *0.70726* | *0.70852* | *0.71688* | *0.72593* | *0.73677* | *0.72494* | *0.73565* | *0.74437* | *0.75317* | *0.76291* | *0.76133* | *0.76076* | *0.76979* | *0.76936* |
| *32762.69185* | *0.66917* | *0.66861* | *0.68443* | *0.68458* | *0.69216* | *0.70433* | *0.70558* | *0.71391* | *0.72303* | *0.73386* | *0.72229* | *0.73302* | *0.74168* | *0.75039* | *0.76022* | *0.75871* | *0.75817* | *0.76708* | *0.76684* |
| *32754.09836* | *0.66627* | *0.66567* | *0.68147* | *0.6817* | *0.68927* | *0.7014* | *0.70265* | *0.71095* | *0.72012* | *0.73095* | *0.71963* | *0.7304* | *0.739* | *0.74761* | *0.75752* | *0.75609* | *0.75559* | *0.76436* | *0.76433* |
| *32745.50937* | *0.66336* | *0.66273* | *0.67851* | *0.67881* | *0.68638* | *0.69847* | *0.69971* | *0.70799* | *0.71722* | *0.72803* | *0.71698* | *0.72776* | *0.7363* | *0.74482* | *0.75481* | *0.75346* | *0.753* | *0.76164* | *0.76181* |
| *32736.92489* | *0.66045* | *0.6598* | *0.67554* | *0.67592* | *0.68348* | *0.69554* | *0.69678* | *0.70503* | *0.71431* | *0.72511* | *0.71432* | *0.72513* | *0.73361* | *0.74203* | *0.7521* | *0.75082* | *0.75041* | *0.75892* | *0.75928* |
| *32728.34491* | *0.65754* | *0.65687* | *0.67258* | *0.67303* | *0.68059* | *0.6926* | *0.69385* | *0.70207* | *0.7114* | *0.72219* | *0.71167* | *0.72249* | *0.7309* | *0.73923* | *0.74938* | *0.74818* | *0.74782* | *0.75619* | *0.75675* |
| *32719.76942* | *0.65463* | *0.65396* | *0.66962* | *0.67015* | *0.6777* | *0.68967* | *0.69092* | *0.69911* | *0.7085* | *0.71926* | *0.70901* | *0.71984* | *0.72819* | *0.73643* | *0.74666* | *0.74553* | *0.74523* | *0.75345* | *0.75422* |
| *32711.19843* | *0.65173* | *0.65104* | *0.66667* | *0.66726* | *0.67481* | *0.68673* | *0.68799* | *0.69616* | *0.70559* | *0.71634* | *0.70636* | *0.71719* | *0.72548* | *0.73362* | *0.74392* | *0.74288* | *0.74263* | *0.75071* | *0.75169* |
| *32702.63192* | *0.64882* | *0.64814* | *0.66371* | *0.66437* | *0.67192* | *0.6838* | *0.68506* | *0.6932* | *0.70268* | *0.71341* | *0.7037* | *0.71454* | *0.72276* | *0.73081* | *0.74119* | *0.74022* | *0.74004* | *0.74796* | *0.74915* |
| *32694.0699* | *0.64592* | *0.64524* | *0.66075* | *0.66148* | *0.66903* | *0.68086* | *0.68213* | *0.69025* | *0.69978* | *0.71048* | *0.70105* | *0.71189* | *0.72004* | *0.728* | *0.73845* | *0.73755* | *0.73744* | *0.74522* | *0.74661* |
| *32685.51237* | *0.64302* | *0.64234* | *0.6578* | *0.65859* | *0.66615* | *0.67793* | *0.67921* | *0.68731* | *0.69687* | *0.70756* | *0.69839* | *0.70923* | *0.71732* | *0.72518* | *0.7357* | *0.73488* | *0.73484* | *0.74246* | *0.74407* |
| *32676.95931* | *0.64012* | *0.63946* | *0.65485* | *0.65571* | *0.66326* | *0.675* | *0.67628* | *0.68436* | *0.69397* | *0.70463* | *0.69574* | *0.70657* | *0.71459* | *0.72236* | *0.73294* | *0.7322* | *0.73224* | *0.7397* | *0.74152* |
| *32668.41073* | *0.63722* | *0.63658* | *0.65191* | *0.65282* | *0.66038* | *0.67206* | *0.67336* | *0.68142* | *0.69107* | *0.7017* | *0.69309* | *0.7039* | *0.71186* | *0.71954* | *0.73019* | *0.72952* | *0.72963* | *0.73694* | *0.73897* |
| *32659.86661* | *0.63433* | *0.63371* | *0.64896* | *0.64994* | *0.6575* | *0.66913* | *0.67045* | *0.67848* | *0.68817* | *0.69878* | *0.69043* | *0.70124* | *0.70912* | *0.71671* | *0.72743* | *0.72684* | *0.72703* | *0.73418* | *0.73642* |
| *32651.32697* | *0.63144* | *0.63084* | *0.64602* | *0.64705* | *0.65462* | *0.6662* | *0.66753* | *0.67554* | *0.68527* | *0.69585* | *0.68778* | *0.69857* | *0.70638* | *0.71388* | *0.72466* | *0.72414* | *0.72443* | *0.73141* | *0.73386* |
| *32642.79179* | *0.62855* | *0.62798* | *0.64308* | *0.64417* | *0.65175* | *0.66328* | *0.66462* | *0.67261* | *0.68237* | *0.69292* | *0.68513* | *0.69589* | *0.70364* | *0.71105* | *0.72189* | *0.72145* | *0.72182* | *0.72864* | *0.73131* |
| *32634.26107* | *0.62566* | *0.62514* | *0.64015* | *0.64129* | *0.64887* | *0.66035* | *0.66172* | *0.66968* | *0.67948* | *0.69* | *0.68248* | *0.69322* | *0.70089* | *0.70821* | *0.71912* | *0.71875* | *0.71921* | *0.72587* | *0.72875* |
| *32625.73481* | *0.62278* | *0.62229* | *0.63722* | *0.63841* | *0.646* | *0.65743* | *0.65881* | *0.66675* | *0.67659* | *0.68708* | *0.67983* | *0.69054* | *0.69814* | *0.70538* | *0.71634* | *0.71604* | *0.71661* | *0.72309* | *0.72619* |
| *32617.21301* | *0.6199* | *0.61946* | *0.63429* | *0.63554* | *0.64314* | *0.6545* | *0.65591* | *0.66383* | *0.6737* | *0.68415* | *0.67718* | *0.68786* | *0.69539* | *0.70254* | *0.71356* | *0.71334* | *0.714* | *0.72031* | *0.72362* |
| *32608.69565* | *0.61702* | *0.61663* | *0.63137* | *0.63266* | *0.64028* | *0.65159* | *0.65302* | *0.66092* | *0.67082* | *0.68123* | *0.67453* | *0.68518* | *0.69264* | *0.6997* | *0.71078* | *0.71062* | *0.71139* | *0.71753* | *0.72105* |
| *32600.18274* | *0.61415* | *0.61381* | *0.62845* | *0.62979* | *0.63742* | *0.64867* | *0.65012* | *0.65801* | *0.66793* | *0.67831* | *0.67188* | *0.68249* | *0.68989* | *0.69686* | *0.70799* | *0.70791* | *0.70878* | *0.71475* | *0.71849* |
| *32591.67428* | *0.61128* | *0.611* | *0.62554* | *0.62692* | *0.63456* | *0.64576* | *0.64724* | *0.6551* | *0.66505* | *0.6754* | *0.66923* | *0.6798* | *0.68713* | *0.69402* | *0.7052* | *0.70519* | *0.70617* | *0.71197* | *0.71591* |
| *32583.17025* | *0.60842* | *0.6082* | *0.62263* | *0.62405* | *0.63171* | *0.64285* | *0.64435* | *0.6522* | *0.66218* | *0.67248* | *0.66659* | *0.67711* | *0.68437* | *0.69118* | *0.70241* | *0.70247* | *0.70356* | *0.70918* | *0.71334* |
| *32574.67067* | *0.60556* | *0.60541* | *0.61973* | *0.62119* | *0.62886* | *0.63994* | *0.64148* | *0.6493* | *0.65931* | *0.66957* | *0.66394* | *0.67442* | *0.68161* | *0.68833* | *0.69962* | *0.69974* | *0.70094* | *0.70639* | *0.71077* |
| *32566.17551* | *0.60271* | *0.60262* | *0.61683* | *0.61833* | *0.62602* | *0.63704* | *0.6386* | *0.64641* | *0.65644* | *0.66666* | *0.6613* | *0.67173* | *0.67885* | *0.68549* | *0.69682* | *0.69702* | *0.69833* | *0.70361* | *0.70819* |
| *32557.68479* | *0.59986* | *0.59985* | *0.61393* | *0.61547* | *0.62318* | *0.63414* | *0.63573* | *0.64352* | *0.65357* | *0.66376* | *0.65866* | *0.66904* | *0.67609* | *0.68265* | *0.69403* | *0.69429* | *0.69572* | *0.70082* | *0.70561* |
| *32549.19849* | *0.59701* | *0.59708* | *0.61105* | *0.61262* | *0.62035* | *0.63125* | *0.63287* | *0.64064* | *0.65071* | *0.66085* | *0.65602* | *0.66634* | *0.67332* | *0.67981* | *0.69123* | *0.69155* | *0.69311* | *0.69803* | *0.70303* |
| *32540.71661* | *0.59417* | *0.59432* | *0.60816* | *0.60977* | *0.61752* | *0.62835* | *0.63001* | *0.63777* | *0.64786* | *0.65795* | *0.65338* | *0.66364* | *0.67056* | *0.67696* | *0.68843* | *0.68882* | *0.69049* | *0.69525* | *0.70045* |
| *32532.23916* | *0.59134* | *0.59157* | *0.60529* | *0.60692* | *0.61469* | *0.62547* | *0.62716* | *0.6349* | *0.645* | *0.65506* | *0.65074* | *0.66095* | *0.6678* | *0.67412* | *0.68563* | *0.68608* | *0.68788* | *0.69246* | *0.69787* |
| *32523.76612* | *0.58851* | *0.58882* | *0.60242* | *0.60408* | *0.61187* | *0.62259* | *0.62431* | *0.63204* | *0.64216* | *0.65216* | *0.6481* | *0.65825* | *0.66503* | *0.67128* | *0.68283* | *0.68334* | *0.68527* | *0.68967* | *0.69528* |
| *32515.29749* | *0.58569* | *0.58609* | *0.59955* | *0.60124* | *0.60906* | *0.61971* | *0.62147* | *0.62918* | *0.63932* | *0.64927* | *0.64547* | *0.65555* | *0.66227* | *0.66844* | *0.68003* | *0.6806* | *0.68266* | *0.68688* | *0.6927* |
| *32506.83327* | *0.58287* | *0.58336* | *0.59669* | *0.5984* | *0.60625* | *0.61684* | *0.61864* | *0.62633* | *0.63648* | *0.64639* | *0.64283* | *0.65285* | *0.6595* | *0.6656* | *0.67723* | *0.67786* | *0.68004* | *0.6841* | *0.69011* |
| *32498.37345* | *0.58005* | *0.58065* | *0.59384* | *0.59557* | *0.60345* | *0.61397* | *0.61581* | *0.62348* | *0.63365* | *0.64351* | *0.6402* | *0.65014* | *0.65674* | *0.66277* | *0.67443* | *0.67511* | *0.67743* | *0.68131* | *0.68752* |
| *32489.91804* | *0.57725* | *0.57794* | *0.59099* | *0.59275* | *0.60065* | *0.61111* | *0.61299* | *0.62065* | *0.63082* | *0.64063* | *0.63757* | *0.64744* | *0.65398* | *0.65993* | *0.67163* | *0.67237* | *0.67482* | *0.67853* | *0.68493* |
| *32481.46703* | *0.57445* | *0.57524* | *0.58815* | *0.58992* | *0.59786* | *0.60825* | *0.61017* | *0.61782* | *0.628* | *0.63776* | *0.63494* | *0.64474* | *0.65121* | *0.6571* | *0.66883* | *0.66962* | *0.67221* | *0.67574* | *0.68234* |
| *32473.02041* | *0.57165* | *0.57255* | *0.58532* | *0.58711* | *0.59508* | *0.6054* | *0.60736* | *0.61499* | *0.62518* | *0.63489* | *0.63231* | *0.64204* | *0.64845* | *0.65427* | *0.66603* | *0.66688* | *0.66959* | *0.67296* | *0.67975* |
| *32464.57819* | *0.56887* | *0.56987* | *0.5825* | *0.5843* | *0.5923* | *0.60256* | *0.60456* | *0.61218* | *0.62237* | *0.63203* | *0.62969* | *0.63934* | *0.64569* | *0.65144* | *0.66323* | *0.66413* | *0.66698* | *0.67018* | *0.67716* |
| *32456.14035* | *0.56609* | *0.5672* | *0.57968* | *0.58149* | *0.58953* | *0.59972* | *0.60176* | *0.60937* | *0.61957* | *0.62917* | *0.62707* | *0.63663* | *0.64293* | *0.64861* | *0.66044* | *0.66138* | *0.66437* | *0.6674* | *0.67456* |
| *32447.7069* | *0.56331* | *0.56454* | *0.57687* | *0.57869* | *0.58676* | *0.59689* | *0.59897* | *0.60656* | *0.61677* | *0.62632* | *0.62444* | *0.63393* | *0.64017* | *0.64579* | *0.65764* | *0.65864* | *0.66176* | *0.66463* | *0.67197* |
| *32439.27783* | *0.56054* | *0.56188* | *0.57406* | *0.57589* | *0.584* | *0.59406* | *0.59619* | *0.60377* | *0.61398* | *0.62348* | *0.62182* | *0.63123* | *0.63742* | *0.64297* | *0.65485* | *0.65589* | *0.65915* | *0.66185* | *0.66938* |
| *32430.85314* | *0.55778* | *0.55924* | *0.57127* | *0.5731* | *0.58125* | *0.59124* | *0.59342* | *0.60098* | *0.61119* | *0.62063* | *0.61921* | *0.62853* | *0.63466* | *0.64015* | *0.65205* | *0.65314* | *0.65654* | *0.65908* | *0.66678* |
| *32422.43282* | *0.55503* | *0.55661* | *0.56848* | *0.57032* | *0.5785* | *0.58842* | *0.59065* | *0.5982* | *0.60841* | *0.6178* | *0.61659* | *0.62583* | *0.63191* | *0.63733* | *0.64926* | *0.6504* | *0.65393* | *0.65631* | *0.66419* |
| *32414.01687* | *0.55228* | *0.55398* | *0.5657* | *0.56754* | *0.57576* | *0.58562* | *0.58789* | *0.59543* | *0.60564* | *0.61497* | *0.61398* | *0.62313* | *0.62916* | *0.63452* | *0.64647* | *0.64765* | *0.65133* | *0.65355* | *0.66159* |
| *32405.60529* | *0.54954* | *0.55136* | *0.56292* | *0.56477* | *0.57303* | *0.58282* | *0.58513* | *0.59266* | *0.60287* | *0.61215* | *0.61137* | *0.62043* | *0.62641* | *0.63171* | *0.64368* | *0.6449* | *0.64872* | *0.65079* | *0.65899* |
| *32397.19808* | *0.54681* | *0.54876* | *0.56016* | *0.562* | *0.5703* | *0.58002* | *0.58239* | *0.58991* | *0.60011* | *0.60933* | *0.60876* | *0.61773* | *0.62366* | *0.62891* | *0.6409* | *0.64216* | *0.64611* | *0.64803* | *0.6564* |
| *32388.79523* | *0.54409* | *0.54616* | *0.5574* | *0.55924* | *0.56759* | *0.57724* | *0.57965* | *0.58716* | *0.59736* | *0.60652* | *0.60616* | *0.61504* | *0.62092* | *0.62611* | *0.63812* | *0.63942* | *0.64351* | *0.64527* | *0.6538* |
| *32380.39673* | *0.54137* | *0.54357* | *0.55465* | *0.55649* | *0.56488* | *0.57446* | *0.57692* | *0.58442* | *0.59462* | *0.60371* | *0.60355* | *0.61234* | *0.61818* | *0.62331* | *0.63534* | *0.63667* | *0.64091* | *0.64252* | *0.65121* |
| *32372.00259* | *0.53866* | *0.54099* | *0.55191* | *0.55374* | *0.56217* | *0.57168* | *0.5742* | *0.58169* | *0.59188* | *0.60091* | *0.60095* | *0.60965* | *0.61544* | *0.62052* | *0.63256* | *0.63393* | *0.6383* | *0.63977* | *0.64861* |
| *32363.6128* | *0.53596* | *0.53842* | *0.54918* | *0.551* | *0.55948* | *0.56892* | *0.57148* | *0.57896* | *0.58914* | *0.59812* | *0.59835* | *0.60696* | *0.61271* | *0.61773* | *0.62978* | *0.63119* | *0.6357* | *0.63702* | *0.64602* |
| *32355.22736* | *0.53327* | *0.53586* | *0.54645* | *0.54827* | *0.55679* | *0.56616* | *0.56878* | *0.57624* | *0.58642* | *0.59534* | *0.59576* | *0.60427* | *0.60997* | *0.61495* | *0.62701* | *0.62846* | *0.6331* | *0.63428* | *0.64342* |
| *32346.84626* | *0.53058* | *0.53331* | *0.54373* | *0.54554* | *0.55411* | *0.56341* | *0.56608* | *0.57354* | *0.5837* | *0.59256* | *0.59317* | *0.60158* | *0.60725* | *0.61217* | *0.62425* | *0.62572* | *0.63051* | *0.63154* | *0.64083* |
| *32338.46951* | *0.52791* | *0.53077* | *0.54103* | *0.54282* | *0.55144* | *0.56067* | *0.56339* | *0.57084* | *0.58099* | *0.58979* | *0.59058* | *0.59889* | *0.60452* | *0.6094* | *0.62148* | *0.62299* | *0.62791* | *0.62881* | *0.63824* |
| *32330.09709* | *0.52524* | *0.52823* | *0.53833* | *0.54011* | *0.54877* | *0.55793* | *0.5607* | *0.56815* | *0.57829* | *0.58702* | *0.58799* | *0.59621* | *0.6018* | *0.60663* | *0.61872* | *0.62026* | *0.62531* | *0.62608* | *0.63564* |
| *32321.729* | *0.52258* | *0.52571* | *0.53564* | *0.5374* | *0.54611* | *0.55521* | *0.55803* | *0.56546* | *0.57559* | *0.58426* | *0.5854* | *0.59353* | *0.59909* | *0.60387* | *0.61597* | *0.61753* | *0.62272* | *0.62336* | *0.63305* |
| *32313.36525* | *0.51992* | *0.52319* | *0.53296* | *0.5347* | *0.54346* | *0.55249* | *0.55536* | *0.56279* | *0.5729* | *0.58151* | *0.58282* | *0.59085* | *0.59637* | *0.60111* | *0.61321* | *0.6148* | *0.62013* | *0.62064* | *0.63046* |
| *32305.00582* | *0.51728* | *0.52069* | *0.53028* | *0.53201* | *0.54082* | *0.54978* | *0.55271* | *0.56012* | *0.57022* | *0.57877* | *0.58024* | *0.58817* | *0.59367* | *0.59836* | *0.61047* | *0.61208* | *0.61754* | *0.61793* | *0.62787* |
| *32296.65072* | *0.51464* | *0.51819* | *0.52762* | *0.52933* | *0.53819* | *0.54707* | *0.55006* | *0.55747* | *0.56755* | *0.57604* | *0.57767* | *0.5855* | *0.59096* | *0.59561* | *0.60772* | *0.60936* | *0.61495* | *0.61522* | *0.62528* |
| *32288.29994* | *0.51202* | *0.51571* | *0.52497* | *0.52665* | *0.53557* | *0.54438* | *0.54742* | *0.55482* | *0.56489* | *0.57331* | *0.5751* | *0.58283* | *0.58826* | *0.59287* | *0.60498* | *0.60664* | *0.61237* | *0.61251* | *0.6227* |
| *32279.95347* | *0.5094* | *0.51323* | *0.52232* | *0.52399* | *0.53295* | *0.54169* | *0.54479* | *0.55218* | *0.56223* | *0.57059* | *0.57253* | *0.58016* | *0.58557* | *0.59014* | *0.60225* | *0.60393* | *0.60978* | *0.60982* | *0.62011* |
| *32271.61132* | *0.50679* | *0.51076* | *0.51969* | *0.52133* | *0.53034* | *0.53902* | *0.54216* | *0.54955* | *0.55958* | *0.56788* | *0.56996* | *0.5775* | *0.58288* | *0.58741* | *0.59952* | *0.60122* | *0.6072* | *0.60712* | *0.61752* |
| *32263.27348* | *0.50419* | *0.5083* | *0.51706* | *0.51867* | *0.52774* | *0.53635* | *0.53955* | *0.54693* | *0.55694* | *0.56517* | *0.5674* | *0.57484* | *0.58019* | *0.58468* | *0.59679* | *0.59851* | *0.60462* | *0.60443* | *0.61494* |
| *32254.93995* | *0.5016* | *0.50585* | *0.51444* | *0.51603* | *0.52515* | *0.53369* | *0.53694* | *0.54432* | *0.55431* | *0.56247* | *0.56484* | *0.57218* | *0.57751* | *0.58197* | *0.59407* | *0.59581* | *0.60204* | *0.60175* | *0.61236* |
| *32246.61072* | *0.49901* | *0.50341* | *0.51183* | *0.51339* | *0.52257* | *0.53104* | *0.53435* | *0.54171* | *0.55168* | *0.55979* | *0.56229* | *0.56952* | *0.57484* | *0.57926* | *0.59136* | *0.59311* | *0.59947* | *0.59908* | *0.60978* |
| *32238.28579* | *0.49644* | *0.50098* | *0.50923* | *0.51077* | *0.52* | *0.52839* | *0.53176* | *0.53912* | *0.54906* | *0.55711* | *0.55974* | *0.56687* | *0.57217* | *0.57655* | *0.58865* | *0.59042* | *0.59689* | *0.59641* | *0.6072* |
| *32229.96516* | *0.49387* | *0.49856* | *0.50664* | *0.50815* | *0.51743* | *0.52576* | *0.52918* | *0.53653* | *0.54646* | *0.55443* | *0.55719* | *0.56423* | *0.56951* | *0.57386* | *0.58594* | *0.58772* | *0.59432* | *0.59374* | *0.60462* |
| *32221.64882* | *0.49132* | *0.49614* | *0.50406* | *0.50554* | *0.51488* | *0.52313* | *0.52661* | *0.53396* | *0.54386* | *0.55177* | *0.55464* | *0.56158* | *0.56685* | *0.57117* | *0.58325* | *0.58504* | *0.59176* | *0.59109* | *0.60205* |
| *32213.33677* | *0.48877* | *0.49374* | *0.50149* | *0.50294* | *0.51233* | *0.52052* | *0.52405* | *0.53139* | *0.54126* | *0.54911* | *0.5521* | *0.55895* | *0.5642* | *0.56848* | *0.58055* | *0.58236* | *0.58919* | *0.58843* | *0.59948* |
| *32205.02901* | *0.48623* | *0.49134* | *0.49893* | *0.50034* | *0.50979* | *0.51791* | *0.5215* | *0.52883* | *0.53868* | *0.54646* | *0.54957* | *0.55631* | *0.56155* | *0.56581* | *0.57787* | *0.57968* | *0.58663* | *0.58579* | *0.59691* |
| *32196.72554* | *0.48371* | *0.48896* | *0.49638* | *0.49776* | *0.50726* | *0.51531* | *0.51896* | *0.52628* | *0.53611* | *0.54383* | *0.54703* | *0.55368* | *0.55891* | *0.56314* | *0.57518* | *0.57701* | *0.58407* | *0.58315* | *0.59434* |
| *32188.42634* | *0.48119* | *0.48658* | *0.49384* | *0.49518* | *0.50474* | *0.51272* | *0.51642* | *0.52374* | *0.53354* | *0.54119* | *0.54451* | *0.55106* | *0.55627* | *0.56048* | *0.57251* | *0.57434* | *0.58151* | *0.58052* | *0.59177* |
| *32180.13143* | *0.47868* | *0.48421* | *0.49131* | *0.49261* | *0.50223* | *0.51014* | *0.5139* | *0.52121* | *0.53098* | *0.53857* | *0.54198* | *0.54844* | *0.55364* | *0.55782* | *0.56984* | *0.57168* | *0.57896* | *0.57789* | *0.58921* |
| *32171.84078* | *0.47618* | *0.48185* | *0.48879* | *0.49005* | *0.49972* | *0.50757* | *0.51138* | *0.51869* | *0.52843* | *0.53596* | *0.53946* | *0.54582* | *0.55102* | *0.55517* | *0.56718* | *0.56902* | *0.57641* | *0.57528* | *0.58665* |
| *32163.55441* | *0.47368* | *0.4795* | *0.48628* | *0.4875* | *0.49723* | *0.50501* | *0.50888* | *0.51618* | *0.52589* | *0.53335* | *0.53694* | *0.54321* | *0.54841* | *0.55253* | *0.56452* | *0.56637* | *0.57386* | *0.57267* | *0.58409* |
| *32155.27231* | *0.4712* | *0.47716* | *0.48377* | *0.48496* | *0.49475* | *0.50246* | *0.50638* | *0.51368* | *0.52336* | *0.53076* | *0.53443* | *0.5406* | *0.5458* | *0.5499* | *0.56187* | *0.56372* | *0.57131* | *0.57006* | *0.58154* |
| *32146.99447* | *0.46873* | *0.47483* | *0.48128* | *0.48243* | *0.49227* | *0.49992* | *0.50389* | *0.51119* | *0.52083* | *0.52817* | *0.53192* | *0.538* | *0.54319* | *0.54728* | *0.55923* | *0.56108* | *0.56877* | *0.56746* | *0.57899* |
| *32138.72089* | *0.46627* | *0.4725* | *0.4788* | *0.47991* | *0.4898* | *0.49738* | *0.50141* | *0.5087* | *0.51832* | *0.52559* | *0.52942* | *0.5354* | *0.5406* | *0.54466* | *0.55659* | *0.55844* | *0.56623* | *0.56487* | *0.57644* |
| *32130.45156* | *0.46381* | *0.47019* | *0.47632* | *0.4774* | *0.48734* | *0.49486* | *0.49894* | *0.50623* | *0.51581* | *0.52302* | *0.52692* | *0.53281* | *0.53801* | *0.54205* | *0.55396* | *0.55582* | *0.5637* | *0.56229* | *0.57389* |
| *32122.1865* | *0.46137* | *0.46788* | *0.47386* | *0.47489* | *0.4849* | *0.49235* | *0.49648* | *0.50376* | *0.51332* | *0.52046* | *0.52443* | *0.53023* | *0.53542* | *0.53945* | *0.55134* | *0.55319* | *0.56117* | *0.55972* | *0.57135* |
| *32113.92568* | *0.45893* | *0.46558* | *0.47141* | *0.4724* | *0.48246* | *0.48984* | *0.49403* | *0.50131* | *0.51083* | *0.51791* | *0.52194* | *0.52765* | *0.53285* | *0.53686* | *0.54872* | *0.55057* | *0.55864* | *0.55715* | *0.56881* |
| *32105.66911* | *0.45651* | *0.46329* | *0.46897* | *0.46991* | *0.48002* | *0.48735* | *0.49159* | *0.49886* | *0.50835* | *0.51536* | *0.51945* | *0.52507* | *0.53028* | *0.53427* | *0.54611* | *0.54796* | *0.55611* | *0.55459* | *0.56628* |
| *32097.41678* | *0.45409* | *0.46101* | *0.46653* | *0.46744* | *0.4776* | *0.48486* | *0.48916* | *0.49642* | *0.50587* | *0.51283* | *0.51697* | *0.52251* | *0.52772* | *0.53169* | *0.54351* | *0.54536* | *0.55359* | *0.55203* | *0.56374* |
| *32089.1687* | *0.45169* | *0.45874* | *0.46411* | *0.46497* | *0.47519* | *0.48238* | *0.48673* | *0.494* | *0.50341* | *0.5103* | *0.5145* | *0.51994* | *0.52517* | *0.52913* | *0.54091* | *0.54276* | *0.55108* | *0.54949* | *0.56121* |
| *32080.92486* | *0.44929* | *0.45647* | *0.4617* | *0.46251* | *0.47279* | *0.47992* | *0.48432* | *0.49158* | *0.50096* | *0.50779* | *0.51203* | *0.51739* | *0.52262* | *0.52656* | *0.53833* | *0.54017* | *0.54856* | *0.54695* | *0.55869* |
| *32072.68524* | *0.44691* | *0.45422* | *0.45929* | *0.46006* | *0.47039* | *0.47746* | *0.48191* | *0.48917* | *0.49851* | *0.50528* | *0.50956* | *0.51484* | *0.52008* | *0.52401* | *0.53575* | *0.53758* | *0.54605* | *0.54442* | *0.55617* |
| *32064.44987* | *0.44453* | *0.45197* | *0.4569* | *0.45763* | *0.46801* | *0.47501* | *0.47952* | *0.48677* | *0.49608* | *0.50278* | *0.5071* | *0.51229* | *0.51755* | *0.52147* | *0.53317* | *0.535* | *0.54355* | *0.5419* | *0.55365* |
| *32056.21871* | *0.44216* | *0.44973* | *0.45452* | *0.4552* | *0.46563* | *0.47258* | *0.47713* | *0.48438* | *0.49365* | *0.50029* | *0.50465* | *0.50975* | *0.51502* | *0.51893* | *0.53061* | *0.53243* | *0.54105* | *0.53939* | *0.55114* |
| *32047.99179* | *0.43981* | *0.4475* | *0.45214* | *0.45278* | *0.46327* | *0.47015* | *0.47476* | *0.482* | *0.49123* | *0.49781* | *0.5022* | *0.50722* | *0.51251* | *0.51641* | *0.52805* | *0.52986* | *0.53855* | *0.53688* | *0.54863* |
| *32039.76908* | *0.43746* | *0.44528* | *0.44978* | *0.45037* | *0.46091* | *0.46773* | *0.47239* | *0.47962* | *0.48882* | *0.49534* | *0.49975* | *0.5047* | *0.51* | *0.51389* | *0.5255* | *0.52731* | *0.53606* | *0.53438* | *0.54613* |
| *32031.5506* | *0.43512* | *0.44307* | *0.44743* | *0.44797* | *0.45856* | *0.46532* | *0.47003* | *0.47726* | *0.48642* | *0.49288* | *0.49731* | *0.50218* | *0.5075* | *0.51138* | *0.52296* | *0.52475* | *0.53357* | *0.53189* | *0.54363* |
| *32023.33633* | *0.43279* | *0.44086* | *0.44508* | *0.44558* | *0.45622* | *0.46293* | *0.46768* | *0.47491* | *0.48403* | *0.49043* | *0.49488* | *0.49967* | *0.505* | *0.50888* | *0.52043* | *0.52221* | *0.53109* | *0.52941* | *0.54113* |
| *32015.12627* | *0.43047* | *0.43866* | *0.44275* | *0.4432* | *0.4539* | *0.46054* | *0.46534* | *0.47256* | *0.48164* | *0.48798* | *0.49245* | *0.49716* | *0.50252* | *0.50639* | *0.5179* | *0.51967* | *0.52861* | *0.52694* | *0.53864* |
| *32006.92042* | *0.42817* | *0.43647* | *0.44043* | *0.44083* | *0.45158* | *0.45816* | *0.46301* | *0.47023* | *0.47927* | *0.48555* | *0.49003* | *0.49467* | *0.50004* | *0.5039* | *0.51538* | *0.51714* | *0.52613* | *0.52447* | *0.53615* |
| *31998.71877* | *0.42587* | *0.43429* | *0.43811* | *0.43847* | *0.44926* | *0.45579* | *0.46069* | *0.4679* | *0.4769* | *0.48312* | *0.48761* | *0.49217* | *0.49757* | *0.50143* | *0.51287* | *0.51462* | *0.52366* | *0.52201* | *0.53367* |
| *31990.52133* | *0.42358* | *0.43212* | *0.43581* | *0.43612* | *0.44696* | *0.45343* | *0.45838* | *0.46559* | *0.47455* | *0.48071* | *0.4852* | *0.48969* | *0.49511* | *0.49896* | *0.51037* | *0.51211* | *0.5212* | *0.51956* | *0.53119* |
| *31982.32808* | *0.4213* | *0.42995* | *0.43351* | *0.43378* | *0.44467* | *0.45108* | *0.45607* | *0.46328* | *0.4722* | *0.4783* | *0.48279* | *0.48721* | *0.49266* | *0.49651* | *0.50787* | *0.5096* | *0.51874* | *0.51712* | *0.52872* |
| *31974.13903* | *0.41903* | *0.4278* | *0.43123* | *0.43145* | *0.44239* | *0.44874* | *0.45378* | *0.46098* | *0.46986* | *0.4759* | *0.48039* | *0.48474* | *0.49021* | *0.49406* | *0.50539* | *0.5071* | *0.51628* | *0.51469* | *0.52625* |
| *31965.95418* | *0.41677* | *0.42565* | *0.42896* | *0.42913* | *0.44011* | *0.44641* | *0.4515* | *0.45869* | *0.46753* | *0.47352* | *0.478* | *0.48228* | *0.48778* | *0.49162* | *0.50291* | *0.50461* | *0.51383* | *0.51227* | *0.52379* |
| *31957.77351* | *0.41452* | *0.4235* | *0.42669* | *0.42682* | *0.43785* | *0.4441* | *0.44922* | *0.45641* | *0.46521* | *0.47114* | *0.47561* | *0.47983* | *0.48535* | *0.48919* | *0.50044* | *0.50213* | *0.51139* | *0.50985* | *0.52133* |
| *31949.59703* | *0.41228* | *0.42137* | *0.42444* | *0.42452* | *0.43559* | *0.44179* | *0.44695* | *0.45414* | *0.4629* | *0.46877* | *0.47323* | *0.47738* | *0.48293* | *0.48677* | *0.49798* | *0.49965* | *0.50895* | *0.50745* | *0.51888* |
| *31941.42473* | *0.41005* | *0.41924* | *0.4222* | *0.42222* | *0.43335* | *0.43949* | *0.4447* | *0.45188* | *0.46059* | *0.46641* | *0.47085* | *0.47494* | *0.48052* | *0.48436* | *0.49553* | *0.49718* | *0.50651* | *0.50505* | *0.51643* |
| *31933.25662* | *0.40782* | *0.41713* | *0.41996* | *0.41994* | *0.43111* | *0.4372* | *0.44245* | *0.44963* | *0.4583* | *0.46406* | *0.46848* | *0.47251* | *0.47811* | *0.48196* | *0.49308* | *0.49472* | *0.50408* | *0.50266* | *0.51399* |
| *31925.09268* | *0.40561* | *0.41501* | *0.41774* | *0.41767* | *0.42888* | *0.43492* | *0.44021* | *0.44738* | *0.45601* | *0.46172* | *0.46612* | *0.47008* | *0.47572* | *0.47956* | *0.49065* | *0.49227* | *0.50166* | *0.50028* | *0.51156* |
| *31916.93291* | *0.40341* | *0.41291* | *0.41552* | *0.41541* | *0.42666* | *0.43265* | *0.43798* | *0.44515* | *0.45373* | *0.45938* | *0.46376* | *0.46767* | *0.47334* | *0.47718* | *0.48822* | *0.48983* | *0.49924* | *0.4979* | *0.50913* |
| *31908.77731* | *0.40122* | *0.41082* | *0.41332* | *0.41316* | *0.42445* | *0.43038* | *0.43576* | *0.44292* | *0.45147* | *0.45706* | *0.46141* | *0.46526* | *0.47096* | *0.4748* | *0.4858* | *0.48739* | *0.49682* | *0.49554* | *0.5067* |
| *31900.62588* | *0.39904* | *0.40873* | *0.41112* | *0.41092* | *0.42225* | *0.42813* | *0.43355* | *0.4407* | *0.44921* | *0.45475* | *0.45907* | *0.46286* | *0.46859* | *0.47244* | *0.48339* | *0.48497* | *0.49442* | *0.49319* | *0.50428* |
| *31892.47861* | *0.39686* | *0.40665* | *0.40894* | *0.40869* | *0.42006* | *0.42589* | *0.43135* | *0.4385* | *0.44695* | *0.45244* | *0.45673* | *0.46046* | *0.46623* | *0.47008* | *0.48099* | *0.48255* | *0.49201* | *0.49084* | *0.50187* |
| *31884.3355* | *0.3947* | *0.40457* | *0.40676* | *0.40646* | *0.41788* | *0.42366* | *0.42915* | *0.4363* | *0.44471* | *0.45015* | *0.4544* | *0.45808* | *0.46388* | *0.46773* | *0.4786* | *0.48014* | *0.48962* | *0.4885* | *0.49946* |
| *31876.19655* | *0.39255* | *0.40251* | *0.4046* | *0.40425* | *0.4157* | *0.42144* | *0.42697* | *0.43411* | *0.44248* | *0.44786* | *0.45207* | *0.4557* | *0.46154* | *0.4654* | *0.47621* | *0.47774* | *0.48723* | *0.48617* | *0.49706* |
| *31868.06176* | *0.3904* | *0.40045* | *0.40244* | *0.40205* | *0.41354* | *0.41923* | *0.42479* | *0.43193* | *0.44025* | *0.44558* | *0.44975* | *0.45333* | *0.4592* | *0.46307* | *0.47384* | *0.47534* | *0.48484* | *0.48385* | *0.49467* |
| *31859.93111* | *0.38827* | *0.3984* | *0.4003* | *0.39986* | *0.41138* | *0.41703* | *0.42263* | *0.42975* | *0.43804* | *0.44332* | *0.44744* | *0.45097* | *0.45688* | *0.46075* | *0.47147* | *0.47296* | *0.48246* | *0.48154* | *0.49228* |
| *31851.80462* | *0.38614* | *0.39636* | *0.39816* | *0.39768* | *0.40924* | *0.41483* | *0.42047* | *0.42759* | *0.43583* | *0.44106* | *0.44514* | *0.44862* | *0.45456* | *0.45844* | *0.46911* | *0.47058* | *0.48009* | *0.47924* | *0.48989* |
| *31843.68226* | *0.38403* | *0.39432* | *0.39603* | *0.39551* | *0.4071* | *0.41265* | *0.41832* | *0.42544* | *0.43363* | *0.43881* | *0.44284* | *0.44628* | *0.45226* | *0.45614* | *0.46676* | *0.46822* | *0.47772* | *0.47694* | *0.48752* |
| *31835.56405* | *0.38192* | *0.39229* | *0.39392* | *0.39335* | *0.40497* | *0.41048* | *0.41618* | *0.42329* | *0.43144* | *0.43657* | *0.44055* | *0.44394* | *0.44996* | *0.45385* | *0.46442* | *0.46586* | *0.47536* | *0.47466* | *0.48515* |
| *31827.44998* | *0.37982* | *0.39027* | *0.39181* | *0.39119* | *0.40285* | *0.40832* | *0.41405* | *0.42115* | *0.42926* | *0.43434* | *0.43826* | *0.44162* | *0.44767* | *0.45157* | *0.46209* | *0.46351* | *0.47301* | *0.47238* | *0.48279* |
| *31819.34004* | *0.37773* | *0.38825* | *0.38971* | *0.38905* | *0.40074* | *0.40616* | *0.41192* | *0.41902* | *0.42708* | *0.43212* | *0.43598* | *0.4393* | *0.44539* | *0.44929* | *0.45977* | *0.46117* | *0.47066* | *0.47011* | *0.48043* |
| *31811.23424* | *0.37566* | *0.38625* | *0.38762* | *0.38692* | *0.39864* | *0.40402* | *0.40981* | *0.4169* | *0.42492* | *0.4299* | *0.43371* | *0.43699* | *0.44312* | *0.44703* | *0.45746* | *0.45884* | *0.46832* | *0.46785* | *0.47808* |
| *31803.13256* | *0.37359* | *0.38425* | *0.38554* | *0.3848* | *0.39655* | *0.40188* | *0.40771* | *0.41479* | *0.42276* | *0.4277* | *0.43145* | *0.43469* | *0.44086* | *0.44478* | *0.45515* | *0.45651* | *0.46598* | *0.4656* | *0.47573* |
| *31795.03501* | *0.37153* | *0.38225* | *0.38347* | *0.38269* | *0.39446* | *0.39976* | *0.40561* | *0.41269* | *0.42061* | *0.42551* | *0.4292* | *0.4324* | *0.4386* | *0.44253* | *0.45285* | *0.4542* | *0.46365* | *0.46336* | *0.4734* |
| *31786.94158* | *0.36948* | *0.38027* | *0.38141* | *0.38058* | *0.39239* | *0.39764* | *0.40352* | *0.41059* | *0.41848* | *0.42332* | *0.42695* | *0.43012* | *0.43636* | *0.4403* | *0.45057* | *0.45189* | *0.46133* | *0.46113* | *0.47107* |
| *31778.85227* | *0.36744* | *0.37829* | *0.37936* | *0.37849* | *0.39032* | *0.39554* | *0.40144* | *0.40851* | *0.41634* | *0.42115* | *0.42471* | *0.42784* | *0.43412* | *0.43807* | *0.44829* | *0.4496* | *0.45901* | *0.4589* | *0.46874* |
| *31770.76708* | *0.36541* | *0.37631* | *0.37732* | *0.37641* | *0.38826* | *0.39344* | *0.39937* | *0.40643* | *0.41422* | *0.41898* | *0.42247* | *0.42558* | *0.4319* | *0.43585* | *0.44602* | *0.44731* | *0.4567* | *0.45669* | *0.46643* |
| *31762.686* | *0.36339* | *0.37435* | *0.37529* | *0.37434* | *0.38621* | *0.39135* | *0.39731* | *0.40436* | *0.41211* | *0.41682* | *0.42025* | *0.42332* | *0.42968* | *0.43365* | *0.44376* | *0.44503* | *0.4544* | *0.45448* | *0.46412* |
| *31754.60903* | *0.36137* | *0.37239* | *0.37327* | *0.37227* | *0.38417* | *0.38928* | *0.39525* | *0.4023* | *0.41* | *0.41467* | *0.41803* | *0.42107* | *0.42747* | *0.43145* | *0.44151* | *0.44276* | *0.4521* | *0.45228* | *0.46182* |
| *31746.53616* | *0.35937* | *0.37044* | *0.37125* | *0.37022* | *0.38214* | *0.38721* | *0.39321* | *0.40024* | *0.4079* | *0.41253* | *0.41581* | *0.41883* | *0.42528* | *0.42926* | *0.43927* | *0.4405* | *0.44981* | *0.45009* | *0.45952* |
| *31738.4674* | *0.35738* | *0.36849* | *0.36925* | *0.36818* | *0.38012* | *0.38515* | *0.39117* | *0.3982* | *0.40581* | *0.4104* | *0.41361* | *0.4166* | *0.42309* | *0.42708* | *0.43704* | *0.43825* | *0.44753* | *0.44791* | *0.45723* |
| *31730.40274* | *0.35539* | *0.36655* | *0.36725* | *0.36615* | *0.3781* | *0.3831* | *0.38914* | *0.39616* | *0.40373* | *0.40828* | *0.41141* | *0.41438* | *0.42091* | *0.42491* | *0.43481* | *0.43601* | *0.44526* | *0.44574* | *0.45495* |
| *31722.34218* | *0.35341* | *0.36462* | *0.36527* | *0.36412* | *0.3761* | *0.38106* | *0.38712* | *0.39414* | *0.40166* | *0.40616* | *0.40922* | *0.41217* | *0.41873* | *0.42275* | *0.4326* | *0.43378* | *0.44299* | *0.44357* | *0.45268* |
| *31714.28571* | *0.35145* | *0.3627* | *0.36329* | *0.36211* | *0.3741* | *0.37903* | *0.38511* | *0.39212* | *0.3996* | *0.40406* | *0.40704* | *0.40997* | *0.41657* | *0.4206* | *0.43039* | *0.43155* | *0.44073* | *0.44142* | *0.45042* |
| *31706.23334* | *0.34949* | *0.36078* | *0.36132* | *0.3601* | *0.37211* | *0.37701* | *0.38311* | *0.3901* | *0.39754* | *0.40196* | *0.40487* | *0.40777* | *0.41442* | *0.41846* | *0.42819* | *0.42934* | *0.43848* | *0.43927* | *0.44816* |
| *31698.18505* | *0.34754* | *0.35887* | *0.35936* | *0.35811* | *0.37013* | *0.375* | *0.38111* | *0.3881* | *0.39549* | *0.39988* | *0.4027* | *0.40559* | *0.41227* | *0.41633* | *0.42601* | *0.42713* | *0.43623* | *0.43714* | *0.44591* |
| *31690.14085* | *0.3456* | *0.35696* | *0.35741* | *0.35612* | *0.36816* | *0.37299* | *0.37913* | *0.3861* | *0.39345* | *0.3978* | *0.40055* | *0.40342* | *0.41014* | *0.4142* | *0.42383* | *0.42494* | *0.43399* | *0.43501* | *0.44366* |
| *31682.10072* | *0.34367* | *0.35507* | *0.35547* | *0.35415* | *0.3662* | *0.371* | *0.37715* | *0.38412* | *0.39142* | *0.39573* | *0.3984* | *0.40125* | *0.40801* | *0.41209* | *0.42166* | *0.42275* | *0.43176* | *0.43289* | *0.44143* |
| *31674.06468* | *0.34175* | *0.35317* | *0.35354* | *0.35218* | *0.36424* | *0.36902* | *0.37518* | *0.38214* | *0.3894* | *0.39367* | *0.39625* | *0.39909* | *0.4059* | *0.40999* | *0.4195* | *0.42058* | *0.42953* | *0.43078* | *0.4392* |
| *31666.03271* | *0.33984* | *0.35129* | *0.35162* | *0.35023* | *0.36229* | *0.36704* | *0.37322* | *0.38017* | *0.38738* | *0.39162* | *0.39412* | *0.39695* | *0.40379* | *0.40789* | *0.41735* | *0.41841* | *0.42732* | *0.42867* | *0.43698* |
| *31658.00482* | *0.33793* | *0.34941* | *0.3497* | *0.34828* | *0.36036* | *0.36507* | *0.37126* | *0.3782* | *0.38537* | *0.38957* | *0.39199* | *0.39481* | *0.40169* | *0.4058* | *0.4152* | *0.41625* | *0.42511* | *0.42658* | *0.43477* |
| *31649.98099* | *0.33604* | *0.34754* | *0.3478* | *0.34634* | *0.35843* | *0.36312* | *0.36932* | *0.37625* | *0.38337* | *0.38754* | *0.38988* | *0.39268* | *0.3996* | *0.40373* | *0.41307* | *0.4141* | *0.4229* | *0.42449* | *0.43256* |
| *31641.96123* | *0.33415* | *0.34567* | *0.3459* | *0.34442* | *0.3565* | *0.36117* | *0.36738* | *0.3743* | *0.38138* | *0.38551* | *0.38777* | *0.39056* | *0.39752* | *0.40166* | *0.41094* | *0.41196* | *0.42071* | *0.42242* | *0.43036* |
| *31633.94554* | *0.33227* | *0.34381* | *0.34401* | *0.3425* | *0.35459* | *0.35923* | *0.36545* | *0.37236* | *0.3794* | *0.3835* | *0.38566* | *0.38845* | *0.39545* | *0.3996* | *0.40883* | *0.40983* | *0.41852* | *0.42035* | *0.42818* |
| *31625.9339* | *0.3304* | *0.34196* | *0.34213* | *0.34059* | *0.35269* | *0.3573* | *0.36353* | *0.37043* | *0.37742* | *0.38149* | *0.38357* | *0.38635* | *0.39339* | *0.39755* | *0.40672* | *0.40771* | *0.41634* | *0.41829* | *0.42599* |
| *31617.92632* | *0.32854* | *0.34011* | *0.34026* | *0.33869* | *0.35079* | *0.35538* | *0.36161* | *0.3685* | *0.37546* | *0.37949* | *0.38148* | *0.38426* | *0.39133* | *0.39551* | *0.40462* | *0.40559* | *0.41417* | *0.41624* | *0.42382* |
| *31609.92279* | *0.32669* | *0.33827* | *0.3384* | *0.3368* | *0.3489* | *0.35346* | *0.35971* | *0.36658* | *0.3735* | *0.3775* | *0.37941* | *0.38218* | *0.38929* | *0.39348* | *0.40254* | *0.40349* | *0.41201* | *0.41419* | *0.42166* |
| *31601.92332* | *0.32485* | *0.33644* | *0.33655* | *0.33492* | *0.34702* | *0.35156* | *0.35781* | *0.36468* | *0.37154* | *0.37551* | *0.37734* | *0.3801* | *0.38726* | *0.39146* | *0.40046* | *0.4014* | *0.40985* | *0.41216* | *0.4195* |
| *31593.92789* | *0.32301* | *0.33461* | *0.3347* | *0.33305* | *0.34514* | *0.34966* | *0.35592* | *0.36277* | *0.3696* | *0.37354* | *0.37528* | *0.37804* | *0.38523* | *0.38944* | *0.39839* | *0.39931* | *0.40771* | *0.41013* | *0.41735* |
| *31585.93651* | *0.32119* | *0.33279* | *0.33287* | *0.33119* | *0.34328* | *0.34778* | *0.35403* | *0.36088* | *0.36766* | *0.37157* | *0.37322* | *0.37599* | *0.38321* | *0.38744* | *0.39632* | *0.39724* | *0.40557* | *0.40811* | *0.41521* |
| *31577.94917* | *0.31937* | *0.33098* | *0.33104* | *0.32933* | *0.34142* | *0.3459* | *0.35216* | *0.35899* | *0.36574* | *0.36961* | *0.37118* | *0.37394* | *0.3812* | *0.38545* | *0.39427* | *0.39517* | *0.40344* | *0.4061* | *0.41308* |
| *31569.96587* | *0.31756* | *0.32917* | *0.32922* | *0.32749* | *0.33957* | *0.34403* | *0.35029* | *0.35712* | *0.36381* | *0.36767* | *0.36914* | *0.37191* | *0.37921* | *0.38346* | *0.39223* | *0.39312* | *0.40131* | *0.4041* | *0.41095* |
| *31561.9866* | *0.31576* | *0.32737* | *0.32741* | *0.32566* | *0.33773* | *0.34217* | *0.34843* | *0.35524* | *0.3619* | *0.36572* | *0.36712* | *0.36988* | *0.37722* | *0.38148* | *0.39019* | *0.39107* | *0.3992* | *0.40211* | *0.40884* |
| *31554.01137* | *0.31397* | *0.32557* | *0.32561* | *0.32383* | *0.3359* | *0.34032* | *0.34658* | *0.35338* | *0.36* | *0.36379* | *0.3651* | *0.36787* | *0.37523* | *0.37951* | *0.38817* | *0.38903* | *0.39709* | *0.40013* | *0.40673* |
| *31546.04017* | *0.31219* | *0.32378* | *0.32381* | *0.32201* | *0.33408* | *0.33847* | *0.34474* | *0.35152* | *0.3581* | *0.36187* | *0.36309* | *0.36586* | *0.37326* | *0.37755* | *0.38615* | *0.387* | *0.39499* | *0.39815* | *0.40463* |
| *31538.07299* | *0.31041* | *0.322* | *0.32203* | *0.32021* | *0.33226* | *0.33664* | *0.3429* | *0.34968* | *0.35621* | *0.35995* | *0.36108* | *0.36386* | *0.3713* | *0.3756* | *0.38414* | *0.38499* | *0.3929* | *0.39618* | *0.40254* |
| *31530.10983* | *0.30865* | *0.32022* | *0.32025* | *0.31841* | *0.33045* | *0.33481* | *0.34107* | *0.34783* | *0.35432* | *0.35804* | *0.35909* | *0.36188* | *0.36935* | *0.37366* | *0.38214* | *0.38298* | *0.39081* | *0.39422* | *0.40045* |
| *31522.1507* | *0.30689* | *0.31845* | *0.31848* | *0.31662* | *0.32865* | *0.333* | *0.33925* | *0.346* | *0.35245* | *0.35614* | *0.3571* | *0.3599* | *0.3674* | *0.37173* | *0.38015* | *0.38098* | *0.38874* | *0.39227* | *0.39838* |
| *31514.19558* | *0.30514* | *0.31669* | *0.31672* | *0.31484* | *0.32685* | *0.33119* | *0.33744* | *0.34417* | *0.35058* | *0.35425* | *0.35513* | *0.35793* | *0.36546* | *0.3698* | *0.37817* | *0.37898* | *0.38667* | *0.39033* | *0.39631* |
| *31506.24448* | *0.3034* | *0.31493* | *0.31496* | *0.31307* | *0.32507* | *0.32939* | *0.33563* | *0.34235* | *0.34872* | *0.35237* | *0.35316* | *0.35597* | *0.36354* | *0.36789* | *0.37619* | *0.377* | *0.38461* | *0.38839* | *0.39426* |
| *31498.29739* | *0.30166* | *0.31318* | *0.31322* | *0.31131* | *0.32329* | *0.32759* | *0.33383* | *0.34054* | *0.34687* | *0.35049* | *0.3512* | *0.35402* | *0.36162* | *0.36598* | *0.37423* | *0.37503* | *0.38256* | *0.38646* | *0.39221* |
| *31490.35431* | *0.29994* | *0.31143* | *0.31148* | *0.30955* | *0.32152* | *0.32581* | *0.33204* | *0.33874* | *0.34502* | *0.34862* | *0.34925* | *0.35208* | *0.35971* | *0.36408* | *0.37227* | *0.37307* | *0.38052* | *0.38454* | *0.39017* |
| *31482.41523* | *0.29822* | *0.30969* | *0.30975* | *0.30781* | *0.31975* | *0.32403* | *0.33025* | *0.33694* | *0.34319* | *0.34676* | *0.34731* | *0.35015* | *0.35781* | *0.36219* | *0.37033* | *0.37111* | *0.37849* | *0.38263* | *0.38813* |
| *31474.48015* | *0.29651* | *0.30796* | *0.30803* | *0.30607* | *0.318* | *0.32227* | *0.32848* | *0.33515* | *0.34136* | *0.34491* | *0.34537* | *0.34823* | *0.35591* | *0.36031* | *0.36839* | *0.36917* | *0.37646* | *0.38073* | *0.38611* |
| *31466.54907* | *0.29481* | *0.30623* | *0.30632* | *0.30434* | *0.31625* | *0.32051* | *0.32671* | *0.33337* | *0.33953* | *0.34307* | *0.34345* | *0.34632* | *0.35403* | *0.35844* | *0.36646* | *0.36723* | *0.37445* | *0.37883* | *0.38409* |
| *31458.62199* | *0.29312* | *0.30451* | *0.30461* | *0.30262* | *0.31451* | *0.31875* | *0.32495* | *0.33159* | *0.33772* | *0.34123* | *0.34153* | *0.34442* | *0.35216* | *0.35658* | *0.36454* | *0.3653* | *0.37244* | *0.37695* | *0.38209* |
| *31450.6989* | *0.29143* | *0.30279* | *0.30291* | *0.30092* | *0.31278* | *0.31701* | *0.32319* | *0.32982* | *0.33591* | *0.33941* | *0.33962* | *0.34252* | *0.35029* | *0.35472* | *0.36263* | *0.36338* | *0.37044* | *0.37507* | *0.38009* |
| *31442.77981* | *0.28975* | *0.30108* | *0.30122* | *0.29921* | *0.31105* | *0.31528* | *0.32144* | *0.32806* | *0.33411* | *0.33759* | *0.33772* | *0.34064* | *0.34843* | *0.35288* | *0.36072* | *0.36148* | *0.36845* | *0.3732* | *0.3781* |
| *31434.86469* | *0.28809* | *0.29938* | *0.29954* | *0.29752* | *0.30934* | *0.31355* | *0.3197* | *0.3263* | *0.33231* | *0.33578* | *0.33583* | *0.33877* | *0.34658* | *0.35104* | *0.35883* | *0.35958* | *0.36647* | *0.37133* | *0.37612* |
| *31426.95357* | *0.28642* | *0.29768* | *0.29787* | *0.29584* | *0.30763* | *0.31183* | *0.31797* | *0.32455* | *0.33053* | *0.33397* | *0.33395* | *0.3369* | *0.34474* | *0.34921* | *0.35694* | *0.35769* | *0.36449* | *0.36948* | *0.37415* |
| *31419.04642* | *0.28477* | *0.29599* | *0.2962* | *0.29416* | *0.30592* | *0.31012* | *0.31624* | *0.32281* | *0.32875* | *0.33217* | *0.33208* | *0.33505* | *0.34291* | *0.34739* | *0.35506* | *0.3558* | *0.36253* | *0.36763* | *0.37218* |
| *31411.14325* | *0.28313* | *0.29431* | *0.29454* | *0.29249* | *0.30423* | *0.30841* | *0.31453* | *0.32108* | *0.32698* | *0.33039* | *0.33021* | *0.3332* | *0.34109* | *0.34558* | *0.3532* | *0.35393* | *0.36057* | *0.36579* | *0.37023* |
| *31403.24406* | *0.28149* | *0.29263* | *0.29289* | *0.29084* | *0.30254* | *0.30672* | *0.31281* | *0.31935* | *0.32521* | *0.32861* | *0.32836* | *0.33136* | *0.33927* | *0.34377* | *0.35133* | *0.35207* | *0.35863* | *0.36396* | *0.36828* |
| *31395.34884* | *0.27986* | *0.29096* | *0.29125* | *0.28918* | *0.30086* | *0.30503* | *0.31111* | *0.31763* | *0.32346* | *0.32683* | *0.32651* | *0.32954* | *0.33747* | *0.34198* | *0.34948* | *0.35021* | *0.35669* | *0.36213* | *0.36635* |
| *31387.45758* | *0.27824* | *0.28929* | *0.28961* | *0.28754* | *0.29918* | *0.30335* | *0.30941* | *0.31592* | *0.3217* | *0.32507* | *0.32467* | *0.32772* | *0.33567* | *0.34019* | *0.34764* | *0.34837* | *0.35476* | *0.36031* | *0.36442* |
| *31379.5703* | *0.27662* | *0.28763* | *0.28798* | *0.28591* | *0.29752* | *0.30168* | *0.30772* | *0.31421* | *0.31996* | *0.32331* | *0.32284* | *0.32591* | *0.33388* | *0.33841* | *0.3458* | *0.34653* | *0.35283* | *0.35851* | *0.3625* |
| *31371.68697* | *0.27501* | *0.28598* | *0.28636* | *0.28428* | *0.29586* | *0.30002* | *0.30604* | *0.31251* | *0.31823* | *0.32156* | *0.32102* | *0.32411* | *0.3321* | *0.33664* | *0.34398* | *0.3447* | *0.35092* | *0.3567* | *0.36058* |
| *31363.80761* | *0.27342* | *0.28433* | *0.28475* | *0.28267* | *0.29421* | *0.29836* | *0.30436* | *0.31082* | *0.3165* | *0.31982* | *0.31921* | *0.32232* | *0.33033* | *0.33487* | *0.34216* | *0.34288* | *0.34902* | *0.35491* | *0.35868* |
| *31355.9322* | *0.27182* | *0.28268* | *0.28314* | *0.28106* | *0.29256* | *0.29671* | *0.30269* | *0.30913* | *0.31477* | *0.31808* | *0.31741* | *0.32054* | *0.32856* | *0.33312* | *0.34035* | *0.34107* | *0.34712* | *0.35312* | *0.35679* |
| *31348.06075* | *0.27024* | *0.28105* | *0.28154* | *0.27946* | *0.29093* | *0.29507* | *0.30103* | *0.30745* | *0.31306* | *0.31636* | *0.31561* | *0.31877* | *0.32681* | *0.33137* | *0.33855* | *0.33927* | *0.34524* | *0.35134* | *0.3549* |
| *31340.19325* | *0.26866* | *0.27942* | *0.27995* | *0.27786* | *0.28929* | *0.29344* | *0.29937* | *0.30578* | *0.31135* | *0.31464* | *0.31383* | *0.31701* | *0.32506* | *0.32963* | *0.33675* | *0.33748* | *0.34336* | *0.34957* | *0.35303* |
| *31332.3297* | *0.2671* | *0.27779* | *0.27837* | *0.27628* | *0.28767* | *0.29181* | *0.29773* | *0.30412* | *0.30965* | *0.31292* | *0.31205* | *0.31525* | *0.32332* | *0.3279* | *0.33497* | *0.3357* | *0.34149* | *0.34781* | *0.35116* |
| *31324.47009* | *0.26553* | *0.27618* | *0.27679* | *0.2747* | *0.28606* | *0.29019* | *0.29608* | *0.30246* | *0.30796* | *0.31122* | *0.31029* | *0.31351* | *0.32159* | *0.32618* | *0.33319* | *0.33392* | *0.33963* | *0.34605* | *0.3493* |
| *31316.61442* | *0.26398* | *0.27456* | *0.27522* | *0.27313* | *0.28445* | *0.28858* | *0.29445* | *0.3008* | *0.30627* | *0.30952* | *0.30853* | *0.31178* | *0.31987* | *0.32447* | *0.33142* | *0.33216* | *0.33778* | *0.3443* | *0.34745* |
| *31308.76269* | *0.26243* | *0.27296* | *0.27366* | *0.27157* | *0.28285* | *0.28698* | *0.29282* | *0.29916* | *0.30459* | *0.30783* | *0.30678* | *0.31005* | *0.31816* | *0.32276* | *0.32966* | *0.3304* | *0.33593* | *0.34256* | *0.34561* |
| *31300.9149* | *0.2609* | *0.27136* | *0.2721* | *0.27002* | *0.28125* | *0.28539* | *0.2912* | *0.29752* | *0.30292* | *0.30615* | *0.30503* | *0.30833* | *0.31645* | *0.32106* | *0.32791* | *0.32865* | *0.3341* | *0.34083* | *0.34377* |
| *31293.07104* | *0.25936* | *0.26976* | *0.27056* | *0.26848* | *0.27966* | *0.2838* | *0.28958* | *0.29589* | *0.30125* | *0.30447* | *0.3033* | *0.30663* | *0.31475* | *0.31937* | *0.32616* | *0.32691* | *0.33228* | *0.3391* | *0.34195* |
| *31285.23112* | *0.25784* | *0.26818* | *0.26902* | *0.26694* | *0.27808* | *0.28222* | *0.28798* | *0.29426* | *0.29959* | *0.30281* | *0.30158* | *0.30493* | *0.31306* | *0.31769* | *0.32443* | *0.32518* | *0.33046* | *0.33738* | *0.34013* |
| *31277.39512* | *0.25632* | *0.2666* | *0.26748* | *0.26541* | *0.27651* | *0.28064* | *0.28638* | *0.29264* | *0.29794* | *0.30114* | *0.29986* | *0.30324* | *0.31138* | *0.31601* | *0.3227* | *0.32346* | *0.32865* | *0.33567* | *0.33833* |
| *31269.56304* | *0.25481* | *0.26502* | *0.26596* | *0.26389* | *0.27494* | *0.27908* | *0.28478* | *0.29103* | *0.29629* | *0.29949* | *0.29816* | *0.30156* | *0.30971* | *0.31434* | *0.32098* | *0.32174* | *0.32685* | *0.33396* | *0.33653* |
| *31261.73489* | *0.25331* | *0.26345* | *0.26444* | *0.26238* | *0.27338* | *0.27752* | *0.28319* | *0.28942* | *0.29465* | *0.29785* | *0.29646* | *0.29989* | *0.30805* | *0.31269* | *0.31927* | *0.32004* | *0.32506* | *0.33227* | *0.33474* |
| *31253.91065* | *0.25182* | *0.26189* | *0.26292* | *0.26087* | *0.27183* | *0.27597* | *0.28161* | *0.28782* | *0.29302* | *0.29621* | *0.29477* | *0.29823* | *0.30639* | *0.31103* | *0.31756* | *0.31834* | *0.32328* | *0.33058* | *0.33296* |
| *31246.09033* | *0.25033* | *0.26033* | *0.26142* | *0.25937* | *0.27028* | *0.27442* | *0.28004* | *0.28623* | *0.2914* | *0.29458* | *0.29309* | *0.29657* | *0.30474* | *0.30939* | *0.31587* | *0.31665* | *0.32151* | *0.32889* | *0.33118* |
| *31238.27392* | *0.24885* | *0.25878* | *0.25992* | *0.25788* | *0.26874* | *0.27289* | *0.27847* | *0.28464* | *0.28978* | *0.29295* | *0.29142* | *0.29493* | *0.3031* | *0.30775* | *0.31418* | *0.31497* | *0.31975* | *0.32722* | *0.32942* |
| *31230.46142* | *0.24737* | *0.25723* | *0.25843* | *0.2564* | *0.26721* | *0.27136* | *0.27691* | *0.28306* | *0.28817* | *0.29133* | *0.28976* | *0.29329* | *0.30146* | *0.30612* | *0.3125* | *0.3133* | *0.31799* | *0.32555* | *0.32767* |
| *31222.65283* | *0.24591* | *0.25569* | *0.25695* | *0.25493* | *0.26568* | *0.26983* | *0.27536* | *0.28149* | *0.28656* | *0.28972* | *0.2881* | *0.29167* | *0.29984* | *0.3045* | *0.31083* | *0.31164* | *0.31625* | *0.32389* | *0.32592* |
| *31214.84814* | *0.24445* | *0.25416* | *0.25547* | *0.25346* | *0.26417* | *0.26832* | *0.27381* | *0.27992* | *0.28496* | *0.28812* | *0.28646* | *0.29005* | *0.29822* | *0.30289* | *0.30916* | *0.30998* | *0.31451* | *0.32223* | *0.32418* |
| *31207.04736* | *0.243* | *0.25263* | *0.254* | *0.252* | *0.26265* | *0.26681* | *0.27227* | *0.27836* | *0.28337* | *0.28653* | *0.28482* | *0.28844* | *0.29661* | *0.30128* | *0.30751* | *0.30833* | *0.31278* | *0.32058* | *0.32245* |
| *31199.25047* | *0.24155* | *0.25111* | *0.25254* | *0.25055* | *0.26115* | *0.26531* | *0.27073* | *0.27681* | *0.28179* | *0.28494* | *0.28319* | *0.28684* | *0.29501* | *0.29969* | *0.30586* | *0.30669* | *0.31107* | *0.31894* | *0.32073* |
| *31191.45747* | *0.24011* | *0.2496* | *0.25108* | *0.2491* | *0.25965* | *0.26381* | *0.2692* | *0.27526* | *0.28021* | *0.28335* | *0.28157* | *0.28525* | *0.29342* | *0.2981* | *0.30422* | *0.30506* | *0.30936* | *0.31731* | *0.31902* |
| *31183.66837* | *0.23868* | *0.24809* | *0.24963* | *0.24766* | *0.25816* | *0.26233* | *0.26768* | *0.27372* | *0.27864* | *0.28178* | *0.27996* | *0.28366* | *0.29183* | *0.29651* | *0.30259* | *0.30344* | *0.30765* | *0.31568* | *0.31732* |
| *31175.88316* | *0.23726* | *0.24659* | *0.24819* | *0.24623* | *0.25667* | *0.26085* | *0.26617* | *0.27219* | *0.27707* | *0.28021* | *0.27836* | *0.28209* | *0.29025* | *0.29494* | *0.30096* | *0.30183* | *0.30596* | *0.31406* | *0.31562* |
| *31168.10183* | *0.23584* | *0.24509* | *0.24675* | *0.24481* | *0.25519* | *0.25937* | *0.26466* | *0.27066* | *0.27552* | *0.27865* | *0.27677* | *0.28052* | *0.28868* | *0.29337* | *0.29934* | *0.30022* | *0.30428* | *0.31245* | *0.31393* |
| *31160.32439* | *0.23443* | *0.2436* | *0.24532* | *0.2434* | *0.25372* | *0.25791* | *0.26316* | *0.26913* | *0.27396* | *0.2771* | *0.27518* | *0.27897* | *0.28712* | *0.29181* | *0.29773* | *0.29862* | *0.3026* | *0.31084* | *0.31226* |
| *31152.55083* | *0.23303* | *0.24212* | *0.2439* | *0.24199* | *0.25226* | *0.25645* | *0.26166* | *0.26762* | *0.27242* | *0.27555* | *0.2736* | *0.27742* | *0.28556* | *0.29025* | *0.29613* | *0.29704* | *0.30094* | *0.30924* | *0.31059* |
| *31144.78114* | *0.23163* | *0.24064* | *0.24248* | *0.24059* | *0.2508* | *0.255* | *0.26017* | *0.26611* | *0.27088* | *0.27401* | *0.27204* | *0.27588* | *0.28402* | *0.28871* | *0.29454* | *0.29545* | *0.29928* | *0.30765* | *0.30893* |
| *31137.01533* | *0.23024* | *0.23917* | *0.24107* | *0.23919* | *0.24935* | *0.25355* | *0.25869* | *0.26461* | *0.26935* | *0.27248* | *0.27048* | *0.27435* | *0.28247* | *0.28717* | *0.29295* | *0.29388* | *0.29763* | *0.30607* | *0.30727* |
| *31129.2534* | *0.22886* | *0.23771* | *0.23967* | *0.23781* | *0.2479* | *0.25211* | *0.25721* | *0.26311* | *0.26783* | *0.27095* | *0.26893* | *0.27282* | *0.28094* | *0.28563* | *0.29137* | *0.29231* | *0.29599* | *0.30449* | *0.30563* |
| *31121.49533* | *0.22749* | *0.23625* | *0.23827* | *0.23643* | *0.24646* | *0.25068* | *0.25574* | *0.26162* | *0.26631* | *0.26943* | *0.26738* | *0.27131* | *0.27942* | *0.28411* | *0.2898* | *0.29076* | *0.29436* | *0.30291* | *0.30399* |
| *31113.74112* | *0.22612* | *0.23479* | *0.23688* | *0.23505* | *0.24503* | *0.24925* | *0.25428* | *0.26014* | *0.2648* | *0.26792* | *0.26585* | *0.2698* | *0.2779* | *0.28259* | *0.28823* | *0.28921* | *0.29274* | *0.30135* | *0.30237* |
| *31105.99078* | *0.22476* | *0.23335* | *0.2355* | *0.23369* | *0.24361* | *0.24784* | *0.25282* | *0.25866* | *0.26329* | *0.26642* | *0.26433* | *0.2683* | *0.27639* | *0.28108* | *0.28668* | *0.28767* | *0.29113* | *0.29979* | *0.30075* |
| *31098.2443* | *0.2234* | *0.23191* | *0.23412* | *0.23233* | *0.24219* | *0.24642* | *0.25137* | *0.25719* | *0.26179* | *0.26492* | *0.26281* | *0.26681* | *0.27489* | *0.27958* | *0.28513* | *0.28613* | *0.28952* | *0.29824* | *0.29914* |
| *31090.50168* | *0.22205* | *0.23047* | *0.23275* | *0.23098* | *0.24077* | *0.24502* | *0.24993* | *0.25572* | *0.2603* | *0.26343* | *0.2613* | *0.26533* | *0.27339* | *0.27808* | *0.28359* | *0.28461* | *0.28793* | *0.29669* | *0.29754* |
| *31082.76291* | *0.22071* | *0.22904* | *0.23139* | *0.22964* | *0.23937* | *0.24362* | *0.24849* | *0.25426* | *0.25881* | *0.26194* | *0.2598* | *0.26386* | *0.2719* | *0.27659* | *0.28205* | *0.28309* | *0.28634* | *0.29516* | *0.29594* |
| *31075.028* | *0.21938* | *0.22762* | *0.23003* | *0.2283* | *0.23797* | *0.24223* | *0.24706* | *0.25281* | *0.25733* | *0.26046* | *0.25831* | *0.26239* | *0.27042* | *0.27511* | *0.28052* | *0.28158* | *0.28476* | *0.29362* | *0.29436* |
| *31067.29693* | *0.21805* | *0.22621* | *0.22868* | *0.22697* | *0.23658* | *0.24084* | *0.24563* | *0.25136* | *0.25586* | *0.25899* | *0.25683* | *0.26094* | *0.26895* | *0.27364* | *0.279* | *0.28007* | *0.28319* | *0.2921* | *0.29278* |
| *31059.56971* | *0.21673* | *0.2248* | *0.22734* | *0.22564* | *0.23519* | *0.23946* | *0.24422* | *0.24992* | *0.2544* | *0.25753* | *0.25535* | *0.25949* | *0.26748* | *0.27217* | *0.27749* | *0.27858* | *0.28163* | *0.29058* | *0.29121* |
| *31051.84633* | *0.21541* | *0.22339* | *0.226* | *0.22433* | *0.23381* | *0.23809* | *0.2428* | *0.24849* | *0.25294* | *0.25607* | *0.25388* | *0.25805* | *0.26602* | *0.27071* | *0.27599* | *0.27709* | *0.28008* | *0.28907* | *0.28965* |
| *31044.12679* | *0.2141* | *0.222* | *0.22467* | *0.22302* | *0.23244* | *0.23673* | *0.2414* | *0.24706* | *0.25148* | *0.25462* | *0.25243* | *0.25662* | *0.26457* | *0.26925* | *0.27449* | *0.27561* | *0.27853* | *0.28756* | *0.2881* |
| *31036.41108* | *0.2128* | *0.22061* | *0.22334* | *0.22172* | *0.23107* | *0.23537* | *0.24* | *0.24564* | *0.25004* | *0.25318* | *0.25098* | *0.25519* | *0.26313* | *0.26781* | *0.273* | *0.27414* | *0.277* | *0.28606* | *0.28656* |
| *31028.69922* | *0.2115* | *0.21922* | *0.22203* | *0.22042* | *0.22971* | *0.23402* | *0.2386* | *0.24422* | *0.2486* | *0.25174* | *0.24954* | *0.25378* | *0.26169* | *0.26637* | *0.27152* | *0.27267* | *0.27547* | *0.28457* | *0.28502* |
| *31020.99118* | *0.21022* | *0.21784* | *0.22071* | *0.21913* | *0.22835* | *0.23267* | *0.23722* | *0.24281* | *0.24716* | *0.25031* | *0.2481* | *0.25237* | *0.26026* | *0.26493* | *0.27004* | *0.27122* | *0.27395* | *0.28309* | *0.28349* |
| *31013.28697* | *0.20893* | *0.21647* | *0.21941* | *0.21785* | *0.227* | *0.23133* | *0.23583* | *0.24141* | *0.24573* | *0.24888* | *0.24668* | *0.25097* | *0.25884* | *0.26351* | *0.26857* | *0.26977* | *0.27244* | *0.28161* | *0.28197* |
| *31005.58659* | *0.20766* | *0.21511* | *0.21811* | *0.21657* | *0.22566* | *0.23* | *0.23446* | *0.24001* | *0.24431* | *0.24746* | *0.24526* | *0.24958* | *0.25742* | *0.26209* | *0.26711* | *0.26833* | *0.27094* | *0.28013* | *0.28046* |
| *30997.89003* | *0.20639* | *0.21375* | *0.21682* | *0.2153* | *0.22433* | *0.22867* | *0.23309* | *0.23862* | *0.2429* | *0.24605* | *0.24385* | *0.24819* | *0.25601* | *0.26067* | *0.26565* | *0.26689* | *0.26945* | *0.27867* | *0.27896* |
| *30990.19729* | *0.20513* | *0.2124* | *0.21553* | *0.21404* | *0.223* | *0.22735* | *0.23173* | *0.23724* | *0.24149* | *0.24465* | *0.24245* | *0.24682* | *0.25461* | *0.25927* | *0.26421* | *0.26546* | *0.26796* | *0.27721* | *0.27746* |
| *30982.50837* | *0.20387* | *0.21105* | *0.21425* | *0.21279* | *0.22168* | *0.22604* | *0.23037* | *0.23586* | *0.24009* | *0.24325* | *0.24106* | *0.24545* | *0.25322* | *0.25787* | *0.26277* | *0.26404* | *0.26649* | *0.27575* | *0.27597* |
| *30974.82327* | *0.20262* | *0.20971* | *0.21298* | *0.21154* | *0.22036* | *0.22473* | *0.22902* | *0.23449* | *0.23869* | *0.24186* | *0.23967* | *0.24409* | *0.25183* | *0.25647* | *0.26133* | *0.26263* | *0.26502* | *0.27431* | *0.27449* |
| *30967.14197* | *0.20138* | *0.20838* | *0.21171* | *0.21029* | *0.21905* | *0.22343* | *0.22768* | *0.23312* | *0.23731* | *0.24047* | *0.2383* | *0.24273* | *0.25045* | *0.25509* | *0.25991* | *0.26123* | *0.26356* | *0.27286* | *0.27302* |
| *30959.46448* | *0.20014* | *0.20705* | *0.21045* | *0.20906* | *0.21775* | *0.22214* | *0.22634* | *0.23176* | *0.23592* | *0.2391* | *0.23693* | *0.24139* | *0.24908* | *0.25371* | *0.25849* | *0.25983* | *0.26211* | *0.27143* | *0.27156* |
| *30951.7908* | *0.19891* | *0.20573* | *0.20919* | *0.20783* | *0.21645* | *0.22085* | *0.22501* | *0.23041* | *0.23455* | *0.23772* | *0.23557* | *0.24005* | *0.24771* | *0.25234* | *0.25708* | *0.25844* | *0.26067* | *0.27* | *0.2701* |
| *30944.12093* | *0.19768* | *0.20441* | *0.20794* | *0.2066* | *0.21516* | *0.21957* | *0.22369* | *0.22906* | *0.23318* | *0.23636* | *0.23421* | *0.23872* | *0.24635* | *0.25097* | *0.25567* | *0.25705* | *0.25923* | *0.26858* | *0.26866* |
| *30936.45485* | *0.19647* | *0.2031* | *0.2067* | *0.20539* | *0.21387* | *0.21829* | *0.22237* | *0.22772* | *0.23181* | *0.235* | *0.23287* | *0.2374* | *0.245* | *0.24961* | *0.25427* | *0.25568* | *0.25781* | *0.26716* | *0.26722* |
| *30928.79257* | *0.19526* | *0.2018* | *0.20546* | *0.20418* | *0.21259* | *0.21702* | *0.22105* | *0.22639* | *0.23046* | *0.23365* | *0.23153* | *0.23608* | *0.24365* | *0.24826* | *0.25288* | *0.25431* | *0.25639* | *0.26575* | *0.26578* |
| *30921.13408* | *0.19405* | *0.20051* | *0.20423* | *0.20297* | *0.21132* | *0.21576* | *0.21975* | *0.22506* | *0.22911* | *0.2323* | *0.2302* | *0.23477* | *0.24231* | *0.24691* | *0.2515* | *0.25295* | *0.25498* | *0.26435* | *0.26436* |
| *30913.47939* | *0.19285* | *0.19922* | *0.20301* | *0.20178* | *0.21006* | *0.2145* | *0.21845* | *0.22374* | *0.22776* | *0.23096* | *0.22888* | *0.23347* | *0.24098* | *0.24557* | *0.25012* | *0.25159* | *0.25358* | *0.26295* | *0.26294* |
| *30905.82849* | *0.19166* | *0.19793* | *0.20179* | *0.20059* | *0.2088* | *0.21325* | *0.21715* | *0.22242* | *0.22642* | *0.22963* | *0.22757* | *0.23218* | *0.23965* | *0.24424* | *0.24875* | *0.25024* | *0.25218* | *0.26156* | *0.26153* |
| *30898.18137* | *0.19047* | *0.19666* | *0.20057* | *0.1994* | *0.20754* | *0.21201* | *0.21587* | *0.22111* | *0.22509* | *0.2283* | *0.22626* | *0.23089* | *0.23833* | *0.24291* | *0.24739* | *0.2489* | *0.2508* | *0.26018* | *0.26013* |
| *30890.53803* | *0.18929* | *0.19539* | *0.19937* | *0.19822* | *0.20629* | *0.21077* | *0.21458* | *0.2198* | *0.22377* | *0.22698* | *0.22497* | *0.22962* | *0.23702* | *0.24159* | *0.24603* | *0.24757* | *0.24942* | *0.2588* | *0.25874* |
| *30882.89848* | *0.18812* | *0.19412* | *0.19817* | *0.19705* | *0.20505* | *0.20954* | *0.21331* | *0.21851* | *0.22245* | *0.22567* | *0.22368* | *0.22835* | *0.23572* | *0.24028* | *0.24468* | *0.24624* | *0.24805* | *0.25743* | *0.25735* |
| *30875.2627* | *0.18695* | *0.19287* | *0.19697* | *0.19588* | *0.20382* | *0.20831* | *0.21204* | *0.21721* | *0.22114* | *0.22436* | *0.22239* | *0.22708* | *0.23442* | *0.23897* | *0.24333* | *0.24492* | *0.24669* | *0.25606* | *0.25598* |
| *30867.6307* | *0.18579* | *0.19162* | *0.19578* | *0.19473* | *0.20259* | *0.20709* | *0.21077* | *0.21593* | *0.21983* | *0.22306* | *0.22112* | *0.22583* | *0.23312* | *0.23767* | *0.242* | *0.24361* | *0.24534* | *0.2547* | *0.25461* |
| *30860.00247* | *0.18463* | *0.19037* | *0.1946* | *0.19357* | *0.20137* | *0.20588* | *0.20952* | *0.21465* | *0.21853* | *0.22177* | *0.21985* | *0.22458* | *0.23184* | *0.23637* | *0.24067* | *0.2423* | *0.244* | *0.25335* | *0.25324* |
| *30852.37801* | *0.18348* | *0.18913* | *0.19343* | *0.19242* | *0.20015* | *0.20467* | *0.20827* | *0.21338* | *0.21724* | *0.22048* | *0.21859* | *0.22334* | *0.23056* | *0.23509* | *0.23934* | *0.241* | *0.24266* | *0.252* | *0.25189* |
| *30844.75732* | *0.18234* | *0.1879* | *0.19226* | *0.19128* | *0.19894* | *0.20347* | *0.20702* | *0.21211* | *0.21595* | *0.2192* | *0.21734* | *0.2221* | *0.22929* | *0.2338* | *0.23803* | *0.23971* | *0.24133* | *0.25066* | *0.25054* |
| *30837.14039* | *0.18121* | *0.18668* | *0.19109* | *0.19015* | *0.19773* | *0.20227* | *0.20578* | *0.21085* | *0.21467* | *0.21792* | *0.21609* | *0.22088* | *0.22802* | *0.23253* | *0.23672* | *0.23842* | *0.24001* | *0.24932* | *0.2492* |
| *30829.52722* | *0.18007* | *0.18546* | *0.18993* | *0.18902* | *0.19654* | *0.20108* | *0.20455* | *0.20959* | *0.21339* | *0.21665* | *0.21486* | *0.21965* | *0.22676* | *0.23126* | *0.23542* | *0.23714* | *0.2387* | *0.248* | *0.24787* |
| *30821.91781* | *0.17895* | *0.18425* | *0.18878* | *0.1879* | *0.19534* | *0.1999* | *0.20332* | *0.20834* | *0.21212* | *0.21539* | *0.21363* | *0.21844* | *0.22551* | *0.23* | *0.23412* | *0.23587* | *0.23739* | *0.24667* | *0.24654* |
| *30814.31215* | *0.17783* | *0.18304* | *0.18764* | *0.18678* | *0.19416* | *0.19872* | *0.2021* | *0.2071* | *0.21086* | *0.21413* | *0.21241* | *0.21724* | *0.22426* | *0.22874* | *0.23283* | *0.2346* | *0.23609* | *0.24536* | *0.24522* |
| *30806.71025* | *0.17672* | *0.18184* | *0.1865* | *0.18567* | *0.19298* | *0.19755* | *0.20089* | *0.20586* | *0.20961* | *0.21288* | *0.21119* | *0.21604* | *0.22302* | *0.22749* | *0.23154* | *0.23335* | *0.2348* | *0.24404* | *0.24391* |
| *30799.1121* | *0.17561* | *0.18065* | *0.18536* | *0.18457* | *0.1918* | *0.19639* | *0.19968* | *0.20463* | *0.20836* | *0.21164* | *0.20998* | *0.21484* | *0.22179* | *0.22624* | *0.23027* | *0.23209* | *0.23352* | *0.24274* | *0.24261* |
| *30791.51769* | *0.17451* | *0.17946* | *0.18423* | *0.18347* | *0.19064* | *0.19523* | *0.19848* | *0.2034* | *0.20711* | *0.2104* | *0.20878* | *0.21366* | *0.22056* | *0.22501* | *0.229* | *0.23085* | *0.23225* | *0.24144* | *0.24131* |
| *30783.92703* | *0.17342* | *0.17828* | *0.18311* | *0.18238* | *0.18948* | *0.19407* | *0.19728* | *0.20219* | *0.20587* | *0.20917* | *0.20759* | *0.21248* | *0.21934* | *0.22377* | *0.22773* | *0.22961* | *0.23098* | *0.24015* | *0.24002* |
| *30776.34011* | *0.17233* | *0.17711* | *0.18199* | *0.18129* | *0.18832* | *0.19293* | *0.19609* | *0.20097* | *0.20464* | *0.20795* | *0.20641* | *0.21131* | *0.21812* | *0.22255* | *0.22648* | *0.22838* | *0.22972* | *0.23886* | *0.23874* |
| *30768.75693* | *0.17125* | *0.17594* | *0.18088* | *0.18021* | *0.18717* | *0.19179* | *0.19491* | *0.19977* | *0.20342* | *0.20673* | *0.20523* | *0.21014* | *0.21692* | *0.22133* | *0.22523* | *0.22715* | *0.22847* | *0.23758* | *0.23746* |
| *30761.17748* | *0.17017* | *0.17478* | *0.17978* | *0.17913* | *0.18603* | *0.19065* | *0.19373* | *0.19857* | *0.2022* | *0.20552* | *0.20406* | *0.20899* | *0.21571* | *0.22012* | *0.22398* | *0.22593* | *0.22723* | *0.23631* | *0.2362* |
| *30753.60177* | *0.1691* | *0.17363* | *0.17868* | *0.17807* | *0.18489* | *0.18952* | *0.19255* | *0.19737* | *0.20099* | *0.20431* | *0.20289* | *0.20784* | *0.21452* | *0.21891* | *0.22274* | *0.22472* | *0.22599* | *0.23504* | *0.23493* |
| *30746.02979* | *0.16804* | *0.17248* | *0.17759* | *0.177* | *0.18376* | *0.1884* | *0.19139* | *0.19618* | *0.19978* | *0.20311* | *0.20174* | *0.20669* | *0.21333* | *0.21771* | *0.22151* | *0.22351* | *0.22476* | *0.23377* | *0.23368* |
| *30738.46154* | *0.16698* | *0.17134* | *0.1765* | *0.17595* | *0.18263* | *0.18728* | *0.19023* | *0.195* | *0.19858* | *0.20192* | *0.20059* | *0.20555* | *0.21215* | *0.21651* | *0.22029* | *0.22231* | *0.22354* | *0.23252* | *0.23243* |
| *30730.89701* | *0.16593* | *0.1702* | *0.17542* | *0.1749* | *0.18151* | *0.18617* | *0.18907* | *0.19382* | *0.19739* | *0.20073* | *0.19944* | *0.20442* | *0.21097* | *0.21532* | *0.21907* | *0.22111* | *0.22233* | *0.23126* | *0.2312* |
| *30723.3362* | *0.16488* | *0.16908* | *0.17434* | *0.17385* | *0.1804* | *0.18506* | *0.18793* | *0.19265* | *0.1962* | *0.19955* | *0.19831* | *0.2033* | *0.2098* | *0.21414* | *0.21785* | *0.21993* | *0.22112* | *0.23002* | *0.22996* |
| *30715.77912* | *0.16384* | *0.16795* | *0.17327* | *0.17281* | *0.17929* | *0.18396* | *0.18678* | *0.19149* | *0.19502* | *0.19837* | *0.19718* | *0.20218* | *0.20863* | *0.21296* | *0.21665* | *0.21875* | *0.21992* | *0.22878* | *0.22874* |
| *30708.22575* | *0.16281* | *0.16684* | *0.17221* | *0.17178* | *0.17819* | *0.18287* | *0.18565* | *0.19033* | *0.19385* | *0.1972* | *0.19606* | *0.20107* | *0.20748* | *0.21179* | *0.21545* | *0.21757* | *0.21873* | *0.22755* | *0.22752* |
| *30700.67609* | *0.16178* | *0.16573* | *0.17115* | *0.17075* | *0.17709* | *0.18178* | *0.18452* | *0.18918* | *0.19268* | *0.19604* | *0.19495* | *0.19996* | *0.20632* | *0.21063* | *0.21426* | *0.2164* | *0.21755* | *0.22632* | *0.22631* |
| *30693.13015* | *0.16076* | *0.16463* | *0.1701* | *0.16973* | *0.17601* | *0.18069* | *0.18339* | *0.18803* | *0.19151* | *0.19488* | *0.19384* | *0.19886* | *0.20518* | *0.20947* | *0.21307* | *0.21524* | *0.21637* | *0.2251* | *0.2251* |
| *30685.58791* | *0.15974* | *0.16353* | *0.16905* | *0.16871* | *0.17492* | *0.17962* | *0.18228* | *0.18689* | *0.19036* | *0.19373* | *0.19274* | *0.19777* | *0.20404* | *0.20832* | *0.21189* | *0.21408* | *0.2152* | *0.22388* | *0.2239* |
| *30678.04938* | *0.15873* | *0.16244* | *0.16801* | *0.1677* | *0.17384* | *0.17855* | *0.18116* | *0.18576* | *0.18921* | *0.19259* | *0.19164* | *0.19669* | *0.2029* | *0.20717* | *0.21071* | *0.21293* | *0.21404* | *0.22267* | *0.22271* |
| *30670.51455* | *0.15772* | *0.16136* | *0.16697* | *0.1667* | *0.17277* | *0.17748* | *0.18006* | *0.18463* | *0.18806* | *0.19145* | *0.19056* | *0.19561* | *0.20178* | *0.20603* | *0.20954* | *0.21179* | *0.21288* | *0.22147* | *0.22153* |
| *30662.98343* | *0.15672* | *0.16028* | *0.16594* | *0.1657* | *0.17171* | *0.17642* | *0.17896* | *0.18351* | *0.18693* | *0.19032* | *0.18948* | *0.19453* | *0.20066* | *0.20489* | *0.20838* | *0.21065* | *0.21174* | *0.22027* | *0.22035* |
| *30655.456* | *0.15573* | *0.15921* | *0.16492* | *0.1647* | *0.17065* | *0.17537* | *0.17786* | *0.18239* | *0.18579* | *0.18919* | *0.1884* | *0.19347* | *0.19954* | *0.20376* | *0.20723* | *0.20952* | *0.2106* | *0.21908* | *0.21918* |
| *30647.93226* | *0.15474* | *0.15815* | *0.1639* | *0.16372* | *0.16959* | *0.17432* | *0.17678* | *0.18128* | *0.18467* | *0.18807* | *0.18734* | *0.19241* | *0.19843* | *0.20264* | *0.20608* | *0.20839* | *0.20946* | *0.21789* | *0.21802* |
| *30640.41222* | *0.15376* | *0.15709* | *0.16289* | *0.16273* | *0.16855* | *0.17328* | *0.17569* | *0.18018* | *0.18355* | *0.18696* | *0.18628* | *0.19135* | *0.19733* | *0.20152* | *0.20493* | *0.20727* | *0.20833* | *0.21671* | *0.21686* |
| *30632.89587* | *0.15278* | *0.15604* | *0.16188* | *0.16176* | *0.1675* | *0.17224* | *0.17462* | *0.17908* | *0.18244* | *0.18585* | *0.18522* | *0.19031* | *0.19623* | *0.20041* | *0.2038* | *0.20616* | *0.20721* | *0.21554* | *0.21571* |
| *30625.3832* | *0.15181* | *0.155* | *0.16088* | *0.16079* | *0.16647* | *0.17121* | *0.17355* | *0.17799* | *0.18133* | *0.18475* | *0.18417* | *0.18926* | *0.19514* | *0.19931* | *0.20266* | *0.20505* | *0.2061* | *0.21437* | *0.21456* |
| *30617.87422* | *0.15085* | *0.15396* | *0.15989* | *0.15982* | *0.16544* | *0.17018* | *0.17248* | *0.1769* | *0.18023* | *0.18366* | *0.18313* | *0.18823* | *0.19405* | *0.19821* | *0.20154* | *0.20395* | *0.205* | *0.2132* | *0.21342* |
| *30610.36892* | *0.14989* | *0.15293* | *0.1589* | *0.15886* | *0.16441* | *0.16916* | *0.17143* | *0.17582* | *0.17913* | *0.18257* | *0.1821* | *0.1872* | *0.19297* | *0.19711* | *0.20042* | *0.20286* | *0.2039* | *0.21205* | *0.21229* |
| *30602.8673* | *0.14894* | *0.1519* | *0.15791* | *0.15791* | *0.16339* | *0.16815* | *0.17037* | *0.17475* | *0.17804* | *0.18148* | *0.18107* | *0.18618* | *0.1919* | *0.19602* | *0.19931* | *0.20177* | *0.2028* | *0.21089* | *0.21117* |
| *30595.36935* | *0.14799* | *0.15088* | *0.15693* | *0.15696* | *0.16238* | *0.16714* | *0.16933* | *0.17368* | *0.17696* | *0.18041* | *0.18005* | *0.18516* | *0.19083* | *0.19494* | *0.1982* | *0.20068* | *0.20172* | *0.20975* | *0.21005* |
| *30587.87508* | *0.14705* | *0.14987* | *0.15596* | *0.15601* | *0.16137* | *0.16614* | *0.16829* | *0.17262* | *0.17589* | *0.17933* | *0.17904* | *0.18415* | *0.18977* | *0.19386* | *0.1971* | *0.19961* | *0.20064* | *0.20861* | *0.20894* |
| *30580.38447* | *0.14611* | *0.14886* | *0.15499* | *0.15508* | *0.16037* | *0.16514* | *0.16725* | *0.17156* | *0.17482* | *0.17827* | *0.17803* | *0.18314* | *0.18871* | *0.19279* | *0.196* | *0.19853* | *0.19957* | *0.20747* | *0.20783* |
| *30572.89754* | *0.14518* | *0.14787* | *0.15403* | *0.15414* | *0.15938* | *0.16415* | *0.16622* | *0.17051* | *0.17375* | *0.17721* | *0.17703* | *0.18214* | *0.18766* | *0.19173* | *0.19491* | *0.19747* | *0.1985* | *0.20634* | *0.20673* |
| *30565.41427* | *0.14426* | *0.14687* | *0.15307* | *0.15322* | *0.15839* | *0.16316* | *0.1652* | *0.16947* | *0.17269* | *0.17616* | *0.17603* | *0.18115* | *0.18662* | *0.19067* | *0.19383* | *0.19641* | *0.19745* | *0.20522* | *0.20564* |
| *30557.93466* | *0.14334* | *0.14589* | *0.15212* | *0.1523* | *0.1574* | *0.16218* | *0.16418* | *0.16843* | *0.17164* | *0.17511* | *0.17504* | *0.18016* | *0.18558* | *0.18961* | *0.19275* | *0.19535* | *0.19639* | *0.2041* | *0.20455* |
| *30550.45872* | *0.14242* | *0.14491* | *0.15118* | *0.15138* | *0.15643* | *0.16121* | *0.16317* | *0.1674* | *0.17059* | *0.17407* | *0.17406* | *0.17918* | *0.18455* | *0.18857* | *0.19168* | *0.19431* | *0.19535* | *0.20299* | *0.20347* |
| *30542.98643* | *0.14152* | *0.14393* | *0.15024* | *0.15047* | *0.15545* | *0.16024* | *0.16217* | *0.16637* | *0.16955* | *0.17303* | *0.17309* | *0.1782* | *0.18352* | *0.18752* | *0.19062* | *0.19326* | *0.19431* | *0.20189* | *0.2024* |
| *30535.51779* | *0.14061* | *0.14296* | *0.14931* | *0.14956* | *0.15449* | *0.15927* | *0.16117* | *0.16535* | *0.16852* | *0.172* | *0.17212* | *0.17723* | *0.1825* | *0.18649* | *0.18956* | *0.19223* | *0.19328* | *0.20078* | *0.20133* |
| *30528.05281* | *0.13972* | *0.142* | *0.14838* | *0.14866* | *0.15353* | *0.15832* | *0.16017* | *0.16434* | *0.16749* | *0.17098* | *0.17115* | *0.17627* | *0.18148* | *0.18546* | *0.18851* | *0.1912* | *0.19225* | *0.19969* | *0.20027* |
| *30520.59147* | *0.13883* | *0.14105* | *0.14745* | *0.14777* | *0.15257* | *0.15736* | *0.15919* | *0.16333* | *0.16647* | *0.16996* | *0.17019* | *0.17531* | *0.18047* | *0.18443* | *0.18746* | *0.19017* | *0.19123* | *0.1986* | *0.19921* |
| *30513.13378* | *0.13794* | *0.1401* | *0.14654* | *0.14688* | *0.15162* | *0.15642* | *0.1582* | *0.16233* | *0.16546* | *0.16895* | *0.16924* | *0.17436* | *0.17947* | *0.18341* | *0.18642* | *0.18915* | *0.19022* | *0.19752* | *0.19816* |
| *30505.67974* | *0.13706* | *0.13916* | *0.14563* | *0.146* | *0.15068* | *0.15548* | *0.15723* | *0.16133* | *0.16445* | *0.16794* | *0.1683* | *0.17341* | *0.17847* | *0.1824* | *0.18538* | *0.18814* | *0.18921* | *0.19644* | *0.19712* |
| *30498.22933* | *0.13618* | *0.13822* | *0.14472* | *0.14512* | *0.14974* | *0.15454* | *0.15626* | *0.16034* | *0.16344* | *0.16694* | *0.16736* | *0.17247* | *0.17747* | *0.18139* | *0.18435* | *0.18713* | *0.18821* | *0.19537* | *0.19608* |
| *30490.78257* | *0.13532* | *0.13729* | *0.14382* | *0.14425* | *0.14881* | *0.15361* | *0.15529* | *0.15936* | *0.16244* | *0.16595* | *0.16642* | *0.17154* | *0.17649* | *0.18038* | *0.18333* | *0.18613* | *0.18722* | *0.1943* | *0.19505* |
| *30483.33944* | *0.13445* | *0.13637* | *0.14292* | *0.14338* | *0.14788* | *0.15268* | *0.15434* | *0.15838* | *0.16145* | *0.16496* | *0.1655* | *0.17061* | *0.1755* | *0.17939* | *0.18231* | *0.18513* | *0.18623* | *0.19324* | *0.19403* |
| *30475.89994* | *0.13359* | *0.13545* | *0.14203* | *0.14252* | *0.14696* | *0.15176* | *0.15338* | *0.15741* | *0.16047* | *0.16398* | *0.16457* | *0.16968* | *0.17453* | *0.17839* | *0.1813* | *0.18414* | *0.18525* | *0.19219* | *0.19301* |
| *30468.46407* | *0.13274* | *0.13454* | *0.14115* | *0.14166* | *0.14605* | *0.15085* | *0.15244* | *0.15644* | *0.15949* | *0.163* | *0.16366* | *0.16876* | *0.17356* | *0.17741* | *0.18029* | *0.18315* | *0.18428* | *0.19114* | *0.192* |
| *30461.03183* | *0.13189* | *0.13364* | *0.14027* | *0.14081* | *0.14514* | *0.14994* | *0.1515* | *0.15548* | *0.15851* | *0.16203* | *0.16275* | *0.16785* | *0.17259* | *0.17643* | *0.17929* | *0.18217* | *0.18331* | *0.19009* | *0.19099* |
| *30453.60322* | *0.13105* | *0.13274* | *0.13939* | *0.13996* | *0.14424* | *0.14904* | *0.15056* | *0.15452* | *0.15755* | *0.16107* | *0.16184* | *0.16694* | *0.17163* | *0.17545* | *0.1783* | *0.1812* | *0.18235* | *0.18905* | *0.18999* |
| *30446.17823* | *0.13021* | *0.13185* | *0.13853* | *0.13912* | *0.14334* | *0.14814* | *0.14963* | *0.15357* | *0.15658* | *0.16011* | *0.16095* | *0.16604* | *0.17068* | *0.17448* | *0.17731* | *0.18023* | *0.18139* | *0.18802* | *0.18899* |
| *30438.75686* | *0.12938* | *0.13096* | *0.13766* | *0.13828* | *0.14244* | *0.14725* | *0.14871* | *0.15263* | *0.15563* | *0.15916* | *0.16005* | *0.16514* | *0.16973* | *0.17352* | *0.17632* | *0.17926* | *0.18044* | *0.18699* | *0.188* |
| *30431.3391* | *0.12856* | *0.13008* | *0.13681* | *0.13745* | *0.14156* | *0.14636* | *0.14779* | *0.15169* | *0.15468* | *0.15821* | *0.15917* | *0.16425* | *0.16879* | *0.17256* | *0.17534* | *0.1783* | *0.1795* | *0.18597* | *0.18702* |
| *30423.92496* | *0.12774* | *0.12921* | *0.13595* | *0.13662* | *0.14068* | *0.14548* | *0.14688* | *0.15076* | *0.15373* | *0.15727* | *0.15829* | *0.16336* | *0.16785* | *0.17161* | *0.17437* | *0.17735* | *0.17856* | *0.18496* | *0.18604* |
| *30416.51443* | *0.12692* | *0.12834* | *0.13511* | *0.1358* | *0.1398* | *0.1446* | *0.14597* | *0.14983* | *0.1528* | *0.15633* | *0.15741* | *0.16248* | *0.16692* | *0.17066* | *0.17341* | *0.1764* | *0.17763* | *0.18395* | *0.18507* |
| *30409.10751* | *0.12611* | *0.12748* | *0.13426* | *0.13498* | *0.13893* | *0.14373* | *0.14507* | *0.14891* | *0.15186* | *0.1554* | *0.15654* | *0.16161* | *0.16599* | *0.16972* | *0.17245* | *0.17546* | *0.1767* | *0.18294* | *0.1841* |
| *30401.7042* | *0.12531* | *0.12663* | *0.13343* | *0.13417* | *0.13807* | *0.14286* | *0.14417* | *0.148* | *0.15094* | *0.15448* | *0.15568* | *0.16074* | *0.16507* | *0.16878* | *0.17149* | *0.17452* | *0.17578* | *0.18194* | *0.18314* |
| *30394.30449* | *0.12451* | *0.12578* | *0.1326* | *0.13337* | *0.13721* | *0.142* | *0.14329* | *0.14709* | *0.15002* | *0.15356* | *0.15482* | *0.15987* | *0.16415* | *0.16785* | *0.17054* | *0.17359* | *0.17486* | *0.18095* | *0.18218* |
| *30386.90838* | *0.12372* | *0.12494* | *0.13177* | *0.13257* | *0.13636* | *0.14115* | *0.1424* | *0.14619* | *0.1491* | *0.15265* | *0.15397* | *0.15901* | *0.16324* | *0.16692* | *0.1696* | *0.17267* | *0.17396* | *0.17996* | *0.18124* |
| *30379.51587* | *0.12293* | *0.1241* | *0.13095* | *0.13177* | *0.13551* | *0.1403* | *0.14152* | *0.14529* | *0.14819* | *0.15174* | *0.15312* | *0.15816* | *0.16234* | *0.166* | *0.16866* | *0.17175* | *0.17305* | *0.17898* | *0.18029* |
| *30372.12696* | *0.12215* | *0.12327* | *0.13014* | *0.13098* | *0.13467* | *0.13945* | *0.14065* | *0.1444* | *0.14729* | *0.15084* | *0.15228* | *0.15731* | *0.16144* | *0.16508* | *0.16773* | *0.17083* | *0.17216* | *0.178* | *0.17935* |
| *30364.74164* | *0.12137* | *0.12244* | *0.12933* | *0.13019* | *0.13383* | *0.13862* | *0.13979* | *0.14351* | *0.14639* | *0.14994* | *0.15144* | *0.15647* | *0.16055* | *0.16417* | *0.1668* | *0.16992* | *0.17127* | *0.17703* | *0.17842* |
| *30357.35991* | *0.12059* | *0.12163* | *0.12852* | *0.12941* | *0.133* | *0.13778* | *0.13892* | *0.14263* | *0.1455* | *0.14905* | *0.15061* | *0.15563* | *0.15966* | *0.16327* | *0.16588* | *0.16902* | *0.17038* | *0.17606* | *0.17749* |
| *30349.98177* | *0.11983* | *0.12082* | *0.12772* | *0.12864* | *0.13217* | *0.13695* | *0.13807* | *0.14176* | *0.14462* | *0.14817* | *0.14979* | *0.1548* | *0.15877* | *0.16237* | *0.16496* | *0.16812* | *0.1695* | *0.1751* | *0.17657* |
| *30342.60722* | *0.11907* | *0.12001* | *0.12693* | *0.12787* | *0.13135* | *0.13613* | *0.13722* | *0.14089* | *0.14374* | *0.14729* | *0.14897* | *0.15397* | *0.15789* | *0.16148* | *0.16405* | *0.16722* | *0.16863* | *0.17414* | *0.17565* |
| *30335.23624* | *0.11831* | *0.11921* | *0.12614* | *0.1271* | *0.13054* | *0.13531* | *0.13637* | *0.14003* | *0.14286* | *0.14642* | *0.14815* | *0.15314* | *0.15702* | *0.16059* | *0.16315* | *0.16633* | *0.16776* | *0.17319* | *0.17474* |
| *30327.86885* | *0.11756* | *0.11842* | *0.12536* | *0.12634* | *0.12973* | *0.1345* | *0.13554* | *0.13917* | *0.142* | *0.14555* | *0.14734* | *0.15233* | *0.15615* | *0.1597* | *0.16225* | *0.16545* | *0.16689* | *0.17225* | *0.17384* |
| *30320.50504* | *0.11681* | *0.11763* | *0.12458* | *0.12558* | *0.12892* | *0.13369* | *0.1347* | *0.13832* | *0.14113* | *0.14469* | *0.14654* | *0.15151* | *0.15529* | *0.15883* | *0.16135* | *0.16457* | *0.16604* | *0.17131* | *0.17294* |
| *30313.1448* | *0.11607* | *0.11685* | *0.1238* | *0.12483* | *0.12812* | *0.13289* | *0.13388* | *0.13748* | *0.14028* | *0.14383* | *0.14574* | *0.15071* | *0.15443* | *0.15795* | *0.16046* | *0.1637* | *0.16518* | *0.17038* | *0.17204* |
| *30305.78813* | *0.11533* | *0.11607* | *0.12304* | *0.12409* | *0.12733* | *0.13209* | *0.13305* | *0.13664* | *0.13943* | *0.14298* | *0.14495* | *0.1499* | *0.15358* | *0.15709* | *0.15958* | *0.16283* | *0.16434* | *0.16945* | *0.17116* |
| *30298.43504* | *0.1146* | *0.1153* | *0.12227* | *0.12334* | *0.12654* | *0.1313* | *0.13224* | *0.1358* | *0.13858* | *0.14214* | *0.14416* | *0.1491* | *0.15274* | *0.15622* | *0.1587* | *0.16196* | *0.1635* | *0.16852* | *0.17027* |
| *30291.08551* | *0.11387* | *0.11454* | *0.12151* | *0.12261* | *0.12576* | *0.13051* | *0.13143* | *0.13497* | *0.13774* | *0.1413* | *0.14338* | *0.14831* | *0.15189* | *0.15537* | *0.15783* | *0.16111* | *0.16266* | *0.16761* | *0.16939* |
| *30283.73954* | *0.11315* | *0.11378* | *0.12076* | *0.12188* | *0.12498* | *0.12973* | *0.13062* | *0.13415* | *0.13691* | *0.14046* | *0.1426* | *0.14752* | *0.15106* | *0.15451* | *0.15696* | *0.16025* | *0.16183* | *0.16669* | *0.16852* |
| *30276.39714* | *0.11244* | *0.11303* | *0.12001* | *0.12115* | *0.12421* | *0.12895* | *0.12982* | *0.13333* | *0.13608* | *0.13964* | *0.14183* | *0.14674* | *0.15023* | *0.15367* | *0.1561* | *0.1594* | *0.16101* | *0.16579* | *0.16765* |
| *30269.0583* | *0.11173* | *0.11228* | *0.11927* | *0.12043* | *0.12345* | *0.12818* | *0.12903* | *0.13252* | *0.13526* | *0.13881* | *0.14106* | *0.14596* | *0.1494* | *0.15283* | *0.15525* | *0.15856* | *0.16019* | *0.16489* | *0.16679* |
| *30261.72301* | *0.11102* | *0.11154* | *0.11853* | *0.11971* | *0.12269* | *0.12741* | *0.12824* | *0.13171* | *0.13444* | *0.138* | *0.1403* | *0.14519* | *0.14858* | *0.15199* | *0.1544* | *0.15772* | *0.15937* | *0.16399* | *0.16593* |
| *30254.39128* | *0.11032* | *0.1108* | *0.1178* | *0.119* | *0.12193* | *0.12665* | *0.12746* | *0.13091* | *0.13363* | *0.13718* | *0.13954* | *0.14442* | *0.14776* | *0.15116* | *0.15355* | *0.15689* | *0.15856* | *0.1631* | *0.16508* |
| *30247.0631* | *0.10962* | *0.11007* | *0.11707* | *0.11829* | *0.12118* | *0.12589* | *0.12668* | *0.13012* | *0.13282* | *0.13638* | *0.13879* | *0.14366* | *0.14695* | *0.15033* | *0.15271* | *0.15606* | *0.15776* | *0.16221* | *0.16423* |
| *30239.73847* | *0.10893* | *0.10935* | *0.11635* | *0.11759* | *0.12043* | *0.12514* | *0.12591* | *0.12933* | *0.13202* | *0.13558* | *0.13804* | *0.1429* | *0.14615* | *0.14951* | *0.15188* | *0.15524* | *0.15696* | *0.16133* | *0.16339* |
| *30232.41738* | *0.10825* | *0.10863* | *0.11563* | *0.11689* | *0.11969* | *0.1244* | *0.12514* | *0.12855* | *0.13123* | *0.13478* | *0.1373* | *0.14214* | *0.14535* | *0.1487* | *0.15105* | *0.15442* | *0.15617* | *0.16046* | *0.16255* |
| *30225.09984* | *0.10757* | *0.10792* | *0.11492* | *0.1162* | *0.11896* | *0.12366* | *0.12438* | *0.12777* | *0.13044* | *0.13399* | *0.13656* | *0.14139* | *0.14455* | *0.14788* | *0.15022* | *0.15361* | *0.15538* | *0.15959* | *0.16172* |
| *30217.78584* | *0.10689* | *0.10722* | *0.11421* | *0.11551* | *0.11823* | *0.12292* | *0.12362* | *0.12699* | *0.12966* | *0.13321* | *0.13583* | *0.14065* | *0.14376* | *0.14708* | *0.1494* | *0.1528* | *0.1546* | *0.15873* | *0.1609* |
| *30210.47538* | *0.10622* | *0.10652* | *0.11351* | *0.11482* | *0.11751* | *0.12219* | *0.12287* | *0.12623* | *0.12888* | *0.13243* | *0.1351* | *0.13991* | *0.14298* | *0.14628* | *0.14859* | *0.152* | *0.15382* | *0.15787* | *0.16007* |
| *30203.16846* | *0.10555* | *0.10582* | *0.11281* | *0.11414* | *0.11679* | *0.12146* | *0.12212* | *0.12546* | *0.12811* | *0.13165* | *0.13438* | *0.13917* | *0.1422* | *0.14548* | *0.14778* | *0.1512* | *0.15305* | *0.15701* | *0.15926* |
| *30195.86507* | *0.10489* | *0.10513* | *0.11212* | *0.11347* | *0.11607* | *0.12074* | *0.12138* | *0.12471* | *0.12734* | *0.13089* | *0.13366* | *0.13844* | *0.14142* | *0.14469* | *0.14698* | *0.1504* | *0.15228* | *0.15616* | *0.15845* |
| *30188.56521* | *0.10424* | *0.10445* | *0.11143* | *0.1128* | *0.11536* | *0.12002* | *0.12065* | *0.12396* | *0.12658* | *0.13012* | *0.13295* | *0.13771* | *0.14065* | *0.14391* | *0.14618* | *0.14962* | *0.15152* | *0.15532* | *0.15764* |
| *30181.26888* | *0.10358* | *0.10377* | *0.11075* | *0.11213* | *0.11466* | *0.11931* | *0.11992* | *0.12321* | *0.12583* | *0.12937* | *0.13224* | *0.13699* | *0.13988* | *0.14313* | *0.14539* | *0.14883* | *0.15076* | *0.15448* | *0.15684* |
| *30173.97608* | *0.10294* | *0.1031* | *0.11007* | *0.11147* | *0.11396* | *0.1186* | *0.1192* | *0.12247* | *0.12508* | *0.12861* | *0.13154* | *0.13628* | *0.13912* | *0.14235* | *0.1446* | *0.14805* | *0.15001* | *0.15365* | *0.15604* |
| *30166.6868* | *0.10229* | *0.10243* | *0.1094* | *0.11081* | *0.11327* | *0.1179* | *0.11848* | *0.12174* | *0.12433* | *0.12787* | *0.13084* | *0.13556* | *0.13837* | *0.14158* | *0.14382* | *0.14728* | *0.14926* | *0.15282* | *0.15525* |
| *30159.40104* | *0.10166* | *0.10177* | *0.10873* | *0.11016* | *0.11258* | *0.11721* | *0.11776* | *0.12101* | *0.12359* | *0.12712* | *0.13015* | *0.13486* | *0.13762* | *0.14082* | *0.14304* | *0.14651* | *0.14851* | *0.152* | *0.15446* |
| *30152.1188* | *0.10103* | *0.10112* | *0.10807* | *0.10952* | *0.1119* | *0.11651* | *0.11706* | *0.12028* | *0.12286* | *0.12639* | *0.12946* | *0.13415* | *0.13687* | *0.14006* | *0.14227* | *0.14574* | *0.14778* | *0.15118* | *0.15368* |
| *30144.84007* | *0.1004* | *0.10047* | *0.10741* | *0.10887* | *0.11122* | *0.11583* | *0.11635* | *0.11956* | *0.12213* | *0.12566* | *0.12878* | *0.13345* | *0.13613* | *0.1393* | *0.1415* | *0.14498* | *0.14704* | *0.15037* | *0.1529* |
| *30137.56486* | *0.09977* | *0.09982* | *0.10676* | *0.10823* | *0.11054* | *0.11514* | *0.11566* | *0.11885* | *0.12141* | *0.12493* | *0.1281* | *0.13276* | *0.1354* | *0.13855* | *0.14074* | *0.14423* | *0.14631* | *0.14956* | *0.15213* |
| *30130.29316* | *0.09916* | *0.09918* | *0.10611* | *0.1076* | *0.10988* | *0.11447* | *0.11496* | *0.11814* | *0.12069* | *0.12421* | *0.12742* | *0.13207* | *0.13466* | *0.1378* | *0.13998* | *0.14348* | *0.14559* | *0.14876* | *0.15136* |
| *30123.02497* | *0.09854* | *0.09855* | *0.10546* | *0.10697* | *0.10921* | *0.11379* | *0.11428* | *0.11744* | *0.11998* | *0.12349* | *0.12675* | *0.13138* | *0.13394* | *0.13706* | *0.13923* | *0.14273* | *0.14487* | *0.14796* | *0.1506* |
| *30115.76028* | *0.09793* | *0.09792* | *0.10482* | *0.10635* | *0.10855* | *0.11313* | *0.11359* | *0.11674* | *0.11927* | *0.12278* | *0.12609* | *0.1307* | *0.13322* | *0.13633* | *0.13848* | *0.14199* | *0.14416* | *0.14717* | *0.14984* |
| *30108.4991* | *0.09733* | *0.0973* | *0.10419* | *0.10573* | *0.1079* | *0.11246* | *0.11292* | *0.11605* | *0.11857* | *0.12208* | *0.12542* | *0.13003* | *0.1325* | *0.1356* | *0.13774* | *0.14126* | *0.14345* | *0.14639* | *0.14909* |
| *30101.24141* | *0.09673* | *0.09668* | *0.10356* | *0.10511* | *0.10725* | *0.1118* | *0.11224* | *0.11536* | *0.11788* | *0.12138* | *0.12477* | *0.12936* | *0.13179* | *0.13487* | *0.13701* | *0.14053* | *0.14274* | *0.14561* | *0.14834* |
| *30093.98723* | *0.09614* | *0.09607* | *0.10293* | *0.1045* | *0.10661* | *0.11115* | *0.11158* | *0.11468* | *0.11718* | *0.12068* | *0.12411* | *0.12869* | *0.13108* | *0.13415* | *0.13627* | *0.1398* | *0.14204* | *0.14483* | *0.1476* |
| *30086.73654* | *0.09555* | *0.09546* | *0.10231* | *0.10389* | *0.10597* | *0.1105* | *0.11092* | *0.114* | *0.1165* | *0.11999* | *0.12347* | *0.12802* | *0.13038* | *0.13343* | *0.13555* | *0.13908* | *0.14134* | *0.14406* | *0.14686* |
| *30079.48934* | *0.09496* | *0.09486* | *0.1017* | *0.10329* | *0.10534* | *0.10986* | *0.11026* | *0.11333* | *0.11582* | *0.11931* | *0.12282* | *0.12737* | *0.12968* | *0.13272* | *0.13483* | *0.13836* | *0.14065* | *0.14329* | *0.14612* |
| *30072.24564* | *0.09438* | *0.09427* | *0.10109* | *0.10269* | *0.10471* | *0.10922* | *0.10961* | *0.11267* | *0.11514* | *0.11863* | *0.12218* | *0.12671* | *0.12899* | *0.13201* | *0.13411* | *0.13765* | *0.13997* | *0.14253* | *0.14539* |
| *30065.00542* | *0.0938* | *0.09368* | *0.10048* | *0.1021* | *0.10409* | *0.10858* | *0.10896* | *0.11201* | *0.11447* | *0.11796* | *0.12155* | *0.12606* | *0.1283* | *0.13131* | *0.1334* | *0.13694* | *0.13928* | *0.14177* | *0.14467* |
| *30057.76868* | *0.09323* | *0.09309* | *0.09988* | *0.10151* | *0.10347* | *0.10795* | *0.10832* | *0.11135* | *0.11381* | *0.11729* | *0.12092* | *0.12541* | *0.12761* | *0.13062* | *0.13269* | *0.13623* | *0.13861* | *0.14102* | *0.14395* |
| *30050.53543* | *0.09266* | *0.09251* | *0.09928* | *0.10092* | *0.10285* | *0.10733* | *0.10768* | *0.1107* | *0.11315* | *0.11662* | *0.12029* | *0.12477* | *0.12694* | *0.12992* | *0.13199* | *0.13553* | *0.13793* | *0.14028* | *0.14323* |
| *30043.30567* | *0.0921* | *0.09193* | *0.09869* | *0.10034* | *0.10224* | *0.10671* | *0.10705* | *0.11005* | *0.1125* | *0.11596* | *0.11967* | *0.12413* | *0.12626* | *0.12924* | *0.13129* | *0.13484* | *0.13726* | *0.13954* | *0.14252* |
| *30036.07937* | *0.09154* | *0.09136* | *0.0981* | *0.09976* | *0.10164* | *0.10609* | *0.10642* | *0.10941* | *0.11185* | *0.11531* | *0.11905* | *0.1235* | *0.12559* | *0.12855* | *0.1306* | *0.13415* | *0.1366* | *0.1388* | *0.14181* |
| *30028.85656* | *0.09099* | *0.0908* | *0.09752* | *0.09919* | *0.10104* | *0.10548* | *0.1058* | *0.10878* | *0.1112* | *0.11466* | *0.11844* | *0.12287* | *0.12493* | *0.12787* | *0.12991* | *0.13346* | *0.13594* | *0.13807* | *0.14111* |
| *30021.63722* | *0.09044* | *0.09024* | *0.09694* | *0.09862* | *0.10044* | *0.10487* | *0.10519* | *0.10815* | *0.11056* | *0.11401* | *0.11783* | *0.12225* | *0.12427* | *0.1272* | *0.12923* | *0.13278* | *0.13528* | *0.13734* | *0.14041* |
| *30014.42134* | *0.08989* | *0.08968* | *0.09636* | *0.09806* | *0.09985* | *0.10427* | *0.10457* | *0.10752* | *0.10993* | *0.11337* | *0.11723* | *0.12163* | *0.12361* | *0.12653* | *0.12855* | *0.13211* | *0.13463* | *0.13662* | *0.13972* |
| *30007.20894* | *0.08935* | *0.08913* | *0.09579* | *0.0975* | *0.09927* | *0.10367* | *0.10397* | *0.1069* | *0.1093* | *0.11274* | *0.11663* | *0.12101* | *0.12296* | *0.12587* | *0.12788* | *0.13143* | *0.13398* | *0.13591* | *0.13903* |
| *30000* | *0.08881* | *0.08859* | *0.09523* | *0.09694* | *0.09869* | *0.10308* | *0.10337* | *0.10629* | *0.10868* | *0.11211* | *0.11603* | *0.1204* | *0.12231* | *0.12521* | *0.12721* | *0.13077* | *0.13334* | *0.13519* | *0.13834* |
| *29992.79452* | *0.08828* | *0.08805* | *0.09467* | *0.09639* | *0.09811* | *0.10249* | *0.10277* | *0.10567* | *0.10806* | *0.11148* | *0.11544* | *0.11979* | *0.12167* | *0.12455* | *0.12654* | *0.1301* | *0.1327* | *0.13449* | *0.13766* |
| *29985.59251* | *0.08775* | *0.08751* | *0.09411* | *0.09584* | *0.09754* | *0.10191* | *0.10218* | *0.10507* | *0.10744* | *0.11086* | *0.11485* | *0.11918* | *0.12103* | *0.1239* | *0.12589* | *0.12944* | *0.13207* | *0.13379* | *0.13699* |
| *29978.39395* | *0.08723* | *0.08698* | *0.09356* | *0.0953* | *0.09697* | *0.10133* | *0.10159* | *0.10447* | *0.10683* | *0.11025* | *0.11427* | *0.11858* | *0.1204* | *0.12326* | *0.12523* | *0.12879* | *0.13144* | *0.13309* | *0.13631* |
| *29971.19885* | *0.08671* | *0.08646* | *0.09301* | *0.09476* | *0.09641* | *0.10075* | *0.10101* | *0.10387* | *0.10623* | *0.10964* | *0.11369* | *0.11799* | *0.11977* | *0.12262* | *0.12458* | *0.12814* | *0.13081* | *0.1324* | *0.13565* |
| *29964.0072* | *0.0862* | *0.08594* | *0.09247* | *0.09423* | *0.09585* | *0.10018* | *0.10043* | *0.10328* | *0.10563* | *0.10903* | *0.11311* | *0.11739* | *0.11915* | *0.12198* | *0.12394* | *0.12749* | *0.13019* | *0.13171* | *0.13498* |
| *29956.819* | *0.08569* | *0.08542* | *0.09193* | *0.0937* | *0.0953* | *0.09961* | *0.09985* | *0.10269* | *0.10504* | *0.10843* | *0.11254* | *0.11681* | *0.11853* | *0.12135* | *0.1233* | *0.12685* | *0.12957* | *0.13103* | *0.13432* |
| *29949.63425* | *0.08518* | *0.08491* | *0.09139* | *0.09317* | *0.09475* | *0.09905* | *0.09929* | *0.10211* | *0.10445* | *0.10784* | *0.11198* | *0.11622* | *0.11791* | *0.12072* | *0.12266* | *0.12621* | *0.12896* | *0.13035* | *0.13367* |
| *29942.45294* | *0.08468* | *0.08441* | *0.09086* | *0.09265* | *0.0942* | *0.09849* | *0.09872* | *0.10154* | *0.10386* | *0.10724* | *0.11141* | *0.11564* | *0.1173* | *0.1201* | *0.12203* | *0.12558* | *0.12835* | *0.12968* | *0.13302* |
| *29935.27508* | *0.08418* | *0.08391* | *0.09034* | *0.09213* | *0.09366* | *0.09794* | *0.09816* | *0.10097* | *0.10328* | *0.10666* | *0.11085* | *0.11506* | *0.11669* | *0.11948* | *0.12141* | *0.12495* | *0.12775* | *0.12901* | *0.13237* |
| *29928.10066* | *0.08369* | *0.08341* | *0.08981* | *0.09161* | *0.09313* | *0.09739* | *0.09761* | *0.1004* | *0.10271* | *0.10608* | *0.1103* | *0.11449* | *0.11609* | *0.11886* | *0.12078* | *0.12433* | *0.12714* | *0.12835* | *0.13173* |
| *29920.92968* | *0.0832* | *0.08292* | *0.0893* | *0.0911* | *0.0926* | *0.09685* | *0.09706* | *0.09984* | *0.10214* | *0.1055* | *0.10975* | *0.11392* | *0.11549* | *0.11826* | *0.12017* | *0.12371* | *0.12655* | *0.12769* | *0.1311* |
| *29913.76213* | *0.08271* | *0.08243* | *0.08878* | *0.09059* | *0.09207* | *0.09631* | *0.09651* | *0.09928* | *0.10157* | *0.10493* | *0.1092* | *0.11336* | *0.1149* | *0.11765* | *0.11955* | *0.12309* | *0.12595* | *0.12703* | *0.13046* |
| *29906.59801* | *0.08223* | *0.08195* | *0.08827* | *0.09009* | *0.09155* | *0.09577* | *0.09597* | *0.09873* | *0.10101* | *0.10436* | *0.10865* | *0.1128* | *0.11431* | *0.11705* | *0.11895* | *0.12248* | *0.12536* | *0.12638* | *0.12983* |
| *29899.43733* | *0.08175* | *0.08147* | *0.08777* | *0.08959* | *0.09103* | *0.09524* | *0.09544* | *0.09818* | *0.10046* | *0.10379* | *0.10812* | *0.11224* | *0.11373* | *0.11645* | *0.11834* | *0.12187* | *0.12478* | *0.12574* | *0.12921* |
| *29892.28007* | *0.08128* | *0.081* | *0.08727* | *0.0891* | *0.09052* | *0.09471* | *0.0949* | *0.09763* | *0.09991* | *0.10324* | *0.10758* | *0.11169* | *0.11315* | *0.11586* | *0.11775* | *0.12127* | *0.1242* | *0.1251* | *0.12859* |
| *29885.12624* | *0.08081* | *0.08053* | *0.08677* | *0.08861* | *0.09001* | *0.09419* | *0.09438* | *0.0971* | *0.09936* | *0.10268* | *0.10705* | *0.11114* | *0.11257* | *0.11527* | *0.11715* | *0.12067* | *0.12362* | *0.12446* | *0.12797* |
| *29877.97583* | *0.08034* | *0.08007* | *0.08628* | *0.08812* | *0.08951* | *0.09367* | *0.09386* | *0.09656* | *0.09882* | *0.10213* | *0.10652* | *0.11059* | *0.112* | *0.11469* | *0.11656* | *0.12008* | *0.12305* | *0.12383* | *0.12736* |
| *29870.82885* | *0.07988* | *0.07961* | *0.08579* | *0.08764* | *0.089* | *0.09315* | *0.09334* | *0.09603* | *0.09828* | *0.10159* | *0.10599* | *0.11005* | *0.11143* | *0.11411* | *0.11598* | *0.11948* | *0.12248* | *0.12321* | *0.12675* |
| *29863.68528* | *0.07943* | *0.07915* | *0.08531* | *0.08716* | *0.08851* | *0.09264* | *0.09282* | *0.09551* | *0.09775* | *0.10105* | *0.10547* | *0.10951* | *0.11087* | *0.11354* | *0.11539* | *0.1189* | *0.12191* | *0.12259* | *0.12615* |
| *29856.54513* | *0.07897* | *0.0787* | *0.08483* | *0.08668* | *0.08802* | *0.09214* | *0.09232* | *0.09499* | *0.09722* | *0.10051* | *0.10496* | *0.10898* | *0.11031* | *0.11297* | *0.11482* | *0.11832* | *0.12135* | *0.12197* | *0.12554* |
| *29849.40839* | *0.07852* | *0.07825* | *0.08435* | *0.08621* | *0.08753* | *0.09163* | *0.09181* | *0.09447* | *0.0967* | *0.09998* | *0.10444* | *0.10845* | *0.10975* | *0.1124* | *0.11425* | *0.11774* | *0.12079* | *0.12136* | *0.12495* |
| *29842.27506* | *0.07808* | *0.07781* | *0.08388* | *0.08574* | *0.08705* | *0.09114* | *0.09131* | *0.09396* | *0.09618* | *0.09945* | *0.10393* | *0.10792* | *0.1092* | *0.11184* | *0.11368* | *0.11716* | *0.12024* | *0.12075* | *0.12436* |
| *29835.14514* | *0.07764* | *0.07738* | *0.08341* | *0.08528* | *0.08657* | *0.09064* | *0.09081* | *0.09345* | *0.09567* | *0.09893* | *0.10343* | *0.1074* | *0.10865* | *0.11128* | *0.11312* | *0.11659* | *0.11969* | *0.12015* | *0.12377* |
| *29828.01863* | *0.0772* | *0.07694* | *0.08295* | *0.08482* | *0.08609* | *0.09015* | *0.09032* | *0.09295* | *0.09516* | *0.09841* | *0.10293* | *0.10688* | *0.10811* | *0.11073* | *0.11256* | *0.11603* | *0.11914* | *0.11955* | *0.12318* |
| *29820.89552* | *0.07677* | *0.07651* | *0.08249* | *0.08436* | *0.08562* | *0.08967* | *0.08984* | *0.09245* | *0.09465* | *0.0979* | *0.10243* | *0.10637* | *0.10757* | *0.11018* | *0.112* | *0.11546* | *0.11859* | *0.11896* | *0.1226* |
| *29813.77581* | *0.07634* | *0.07609* | *0.08203* | *0.08391* | *0.08516* | *0.08918* | *0.08935* | *0.09196* | *0.09415* | *0.09739* | *0.10193* | *0.10585* | *0.10704* | *0.10964* | *0.11145* | *0.11491* | *0.11806* | *0.11837* | *0.12203* |
| *29806.65951* | *0.07591* | *0.07567* | *0.08158* | *0.08346* | *0.08469* | *0.08871* | *0.08887* | *0.09147* | *0.09365* | *0.09688* | *0.10144* | *0.10535* | *0.10651* | *0.1091* | *0.11091* | *0.11435* | *0.11752* | *0.11778* | *0.12145* |
| *29799.54659* | *0.07549* | *0.07525* | *0.08113* | *0.08301* | *0.08423* | *0.08823* | *0.0884* | *0.09098* | *0.09316* | *0.09638* | *0.10095* | *0.10484* | *0.10598* | *0.10856* | *0.11036* | *0.1138* | *0.11699* | *0.1172* | *0.12089* |
| *29792.43708* | *0.07507* | *0.07484* | *0.08069* | *0.08257* | *0.08378* | *0.08776* | *0.08793* | *0.0905* | *0.09267* | *0.09588* | *0.10047* | *0.10434* | *0.10546* | *0.10803* | *0.10983* | *0.11326* | *0.11646* | *0.11662* | *0.12032* |
| *29785.33095* | *0.07465* | *0.07444* | *0.08025* | *0.08213* | *0.08333* | *0.0873* | *0.08746* | *0.09003* | *0.09219* | *0.09539* | *0.09999* | *0.10384* | *0.10494* | *0.1075* | *0.10929* | *0.11271* | *0.11593* | *0.11605* | *0.11976* |
| *29778.22821* | *0.07424* | *0.07403* | *0.07981* | *0.0817* | *0.08288* | *0.08683* | *0.087* | *0.08956* | *0.09171* | *0.0949* | *0.09951* | *0.10335* | *0.10443* | *0.10698* | *0.10876* | *0.11218* | *0.11541* | *0.11548* | *0.1192* |
| *29771.12886* | *0.07384* | *0.07363* | *0.07938* | *0.08127* | *0.08244* | *0.08638* | *0.08655* | *0.08909* | *0.09124* | *0.09442* | *0.09904* | *0.10286* | *0.10392* | *0.10646* | *0.10824* | *0.11164* | *0.11489* | *0.11492* | *0.11865* |
| *29764.03289* | *0.07343* | *0.07324* | *0.07895* | *0.08084* | *0.082* | *0.08592* | *0.08609* | *0.08862* | *0.09077* | *0.09394* | *0.09857* | *0.10237* | *0.10341* | *0.10594* | *0.10772* | *0.11111* | *0.11438* | *0.11436* | *0.1181* |
| *29756.94031* | *0.07303* | *0.07285* | *0.07852* | *0.08042* | *0.08157* | *0.08547* | *0.08564* | *0.08817* | *0.0903* | *0.09346* | *0.0981* | *0.10189* | *0.10291* | *0.10543* | *0.1072* | *0.11058* | *0.11387* | *0.11381* | *0.11756* |
| *29749.8511* | *0.07264* | *0.07246* | *0.0781* | *0.08* | *0.08114* | *0.08503* | *0.0852* | *0.08771* | *0.08984* | *0.09299* | *0.09764* | *0.10141* | *0.10241* | *0.10492* | *0.10669* | *0.11006* | *0.11336* | *0.11326* | *0.11701* |
| *29742.76527* | *0.07224* | *0.07208* | *0.07769* | *0.07958* | *0.08071* | *0.08458* | *0.08476* | *0.08726* | *0.08938* | *0.09252* | *0.09718* | *0.10093* | *0.10192* | *0.10442* | *0.10618* | *0.10954* | *0.11286* | *0.11271* | *0.11648* |
| *29735.68282* | *0.07185* | *0.0717* | *0.07727* | *0.07917* | *0.08029* | *0.08414* | *0.08432* | *0.08681* | *0.08893* | *0.09206* | *0.09673* | *0.10046* | *0.10143* | *0.10392* | *0.10568* | *0.10903* | *0.11236* | *0.11217* | *0.11594* |
| *29728.60374* | *0.07147* | *0.07132* | *0.07686* | *0.07876* | *0.07987* | *0.08371* | *0.08389* | *0.08637* | *0.08848* | *0.0916* | *0.09627* | *0.09999* | *0.10094* | *0.10343* | *0.10518* | *0.10852* | *0.11186* | *0.11163* | *0.11541* |
| *29721.52803* | *0.07109* | *0.07095* | *0.07646* | *0.07835* | *0.07946* | *0.08328* | *0.08346* | *0.08593* | *0.08803* | *0.09115* | *0.09582* | *0.09952* | *0.10046* | *0.10293* | *0.10468* | *0.10801* | *0.11137* | *0.1111* | *0.11488* |
| *29714.45568* | *0.07071* | *0.07058* | *0.07605* | *0.07795* | *0.07904* | *0.08285* | *0.08303* | *0.0855* | *0.08759* | *0.0907* | *0.09538* | *0.09906* | *0.09998* | *0.10245* | *0.10419* | *0.10751* | *0.11088* | *0.11057* | *0.11436* |
| *29707.3867* | *0.07033* | *0.07022* | *0.07565* | *0.07755* | *0.07864* | *0.08243* | *0.08261* | *0.08507* | *0.08716* | *0.09025* | *0.09493* | *0.0986* | *0.0995* | *0.10196* | *0.1037* | *0.10701* | *0.11039* | *0.11005* | *0.11384* |
| *29700.32108* | *0.06996* | *0.06986* | *0.07526* | *0.07715* | *0.07823* | *0.08201* | *0.0822* | *0.08464* | *0.08672* | *0.08981* | *0.0945* | *0.09815* | *0.09903* | *0.10148* | *0.10322* | *0.10651* | *0.10991* | *0.10953* | *0.11333* |
| *29693.25883* | *0.0696* | *0.06951* | *0.07487* | *0.07676* | *0.07783* | *0.08159* | *0.08178* | *0.08422* | *0.0863* | *0.08937* | *0.09406* | *0.0977* | *0.09856* | *0.10101* | *0.10274* | *0.10602* | *0.10943* | *0.10901* | *0.11281* |
| *29686.19993* | *0.06923* | *0.06915* | *0.07448* | *0.07637* | *0.07744* | *0.08118* | *0.08137* | *0.0838* | *0.08587* | *0.08893* | *0.09363* | *0.09725* | *0.0981* | *0.10054* | *0.10226* | *0.10553* | *0.10895* | *0.1085* | *0.11231* |
| *29679.14439* | *0.06887* | *0.06881* | *0.07409* | *0.07598* | *0.07705* | *0.08077* | *0.08097* | *0.08339* | *0.08545* | *0.0885* | *0.0932* | *0.0968* | *0.09764* | *0.10007* | *0.10179* | *0.10505* | *0.10848* | *0.10799* | *0.1118* |
| *29672.09219* | *0.06851* | *0.06846* | *0.07371* | *0.0756* | *0.07666* | *0.08037* | *0.08057* | *0.08298* | *0.08503* | *0.08807* | *0.09277* | *0.09636* | *0.09718* | *0.0996* | *0.10132* | *0.10456* | *0.10801* | *0.10749* | *0.1113* |
| *29665.04335* | *0.06816* | *0.06812* | *0.07334* | *0.07522* | *0.07627* | *0.07996* | *0.08017* | *0.08257* | *0.08462* | *0.08765* | *0.09235* | *0.09592* | *0.09673* | *0.09914* | *0.10086* | *0.10409* | *0.10754* | *0.10699* | *0.1108* |
| *29657.99786* | *0.06781* | *0.06778* | *0.07296* | *0.07485* | *0.07589* | *0.07957* | *0.07978* | *0.08217* | *0.08421* | *0.08723* | *0.09193* | *0.09548* | *0.09628* | *0.09869* | *0.1004* | *0.10361* | *0.10708* | *0.10649* | *0.11031* |
| *29650.95572* | *0.06746* | *0.06745* | *0.07259* | *0.07447* | *0.07551* | *0.07917* | *0.07939* | *0.08177* | *0.08381* | *0.08682* | *0.09151* | *0.09505* | *0.09584* | *0.09823* | *0.09994* | *0.10314* | *0.10662* | *0.106* | *0.10982* |
| *29643.91691* | *0.06712* | *0.06712* | *0.07222* | *0.07411* | *0.07514* | *0.07878* | *0.079* | *0.08137* | *0.08341* | *0.0864* | *0.0911* | *0.09462* | *0.0954* | *0.09779* | *0.09949* | *0.10268* | *0.10616* | *0.10551* | *0.10933* |
| *29636.88145* | *0.06678* | *0.06679* | *0.07186* | *0.07374* | *0.07477* | *0.0784* | *0.07862* | *0.08098* | *0.08301* | *0.086* | *0.09069* | *0.0942* | *0.09496* | *0.09734* | *0.09904* | *0.10221* | *0.10571* | *0.10503* | *0.10885* |
| *29629.84933* | *0.06644* | *0.06647* | *0.0715* | *0.07338* | *0.0744* | *0.07801* | *0.07824* | *0.0806* | *0.08262* | *0.08559* | *0.09028* | *0.09377* | *0.09452* | *0.0969* | *0.09859* | *0.10175* | *0.10526* | *0.10455* | *0.10837* |
| *29622.82054* | *0.0661* | *0.06615* | *0.07114* | *0.07302* | *0.07404* | *0.07763* | *0.07786* | *0.08021* | *0.08223* | *0.08519* | *0.08988* | *0.09335* | *0.09409* | *0.09646* | *0.09815* | *0.1013* | *0.10481* | *0.10407* | *0.10789* |
| *29615.79509* | *0.06577* | *0.06584* | *0.07079* | *0.07266* | *0.07368* | *0.07725* | *0.07749* | *0.07983* | *0.08184* | *0.0848* | *0.08948* | *0.09294* | *0.09367* | *0.09603* | *0.09771* | *0.10085* | *0.10437* | *0.1036* | *0.10742* |
| *29608.77297* | *0.06544* | *0.06553* | *0.07044* | *0.07231* | *0.07332* | *0.07688* | *0.07712* | *0.07946* | *0.08146* | *0.0844* | *0.08908* | *0.09253* | *0.09324* | *0.0956* | *0.09728* | *0.1004* | *0.10393* | *0.10313* | *0.10695* |
| *29601.75418* | *0.06512* | *0.06522* | *0.07009* | *0.07196* | *0.07297* | *0.07651* | *0.07676* | *0.07908* | *0.08108* | *0.08401* | *0.08869* | *0.09212* | *0.09282* | *0.09517* | *0.09685* | *0.09995* | *0.10349* | *0.10267* | *0.10648* |
| *29594.73871* | *0.0648* | *0.06491* | *0.06975* | *0.07161* | *0.07262* | *0.07615* | *0.0764* | *0.07872* | *0.08071* | *0.08363* | *0.0883* | *0.09171* | *0.09241* | *0.09475* | *0.09642* | *0.09951* | *0.10305* | *0.10221* | *0.10602* |
| *29587.72657* | *0.06448* | *0.06461* | *0.06941* | *0.07127* | *0.07227* | *0.07578* | *0.07604* | *0.07835* | *0.08034* | *0.08325* | *0.08791* | *0.09131* | *0.09199* | *0.09433* | *0.096* | *0.09907* | *0.10262* | *0.10176* | *0.10556* |
| *29580.71775* | *0.06417* | *0.06431* | *0.06907* | *0.07093* | *0.07193* | *0.07542* | *0.07569* | *0.07799* | *0.07997* | *0.08287* | *0.08752* | *0.0909* | *0.09158* | *0.09391* | *0.09558* | *0.09864* | *0.10219* | *0.1013* | *0.1051* |
| *29573.71226* | *0.06385* | *0.06402* | *0.06874* | *0.07059* | *0.07159* | *0.07507* | *0.07534* | *0.07763* | *0.07961* | *0.08249* | *0.08714* | *0.09051* | *0.09118* | *0.0935* | *0.09517* | *0.09821* | *0.10177* | *0.10085* | *0.10465* |
| *29566.71007* | *0.06354* | *0.06372* | *0.06841* | *0.07026* | *0.07126* | *0.07471* | *0.07499* | *0.07728* | *0.07925* | *0.08212* | *0.08676* | *0.09011* | *0.09078* | *0.09309* | *0.09476* | *0.09778* | *0.10134* | *0.10041* | *0.1042* |
| *29559.71121* | *0.06324* | *0.06344* | *0.06808* | *0.06992* | *0.07092* | *0.07437* | *0.07465* | *0.07693* | *0.07889* | *0.08175* | *0.08638* | *0.08972* | *0.09038* | *0.09268* | *0.09435* | *0.09735* | *0.10093* | *0.09997* | *0.10376* |
| *29552.71565* | *0.06294* | *0.06315* | *0.06776* | *0.0696* | *0.07059* | *0.07402* | *0.07431* | *0.07658* | *0.07854* | *0.08139* | *0.08601* | *0.08933* | *0.08998* | *0.09228* | *0.09394* | *0.09693* | *0.10051* | *0.09953* | *0.10331* |
| *29545.72341* | *0.06264* | *0.06287* | *0.06744* | *0.06927* | *0.07027* | *0.07368* | *0.07397* | *0.07624* | *0.07819* | *0.08103* | *0.08564* | *0.08895* | *0.08959* | *0.09188* | *0.09354* | *0.09652* | *0.1001* | *0.0991* | *0.10288* |
| *29538.73448* | *0.06234* | *0.06259* | *0.06712* | *0.06895* | *0.06994* | *0.07334* | *0.07364* | *0.0759* | *0.07784* | *0.08067* | *0.08527* | *0.08857* | *0.0892* | *0.09149* | *0.09314* | *0.0961* | *0.09969* | *0.09867* | *0.10244* |
| *29531.74885* | *0.06205* | *0.06231* | *0.06681* | *0.06863* | *0.06963* | *0.073* | *0.07331* | *0.07556* | *0.0775* | *0.08032* | *0.08491* | *0.08819* | *0.08881* | *0.0911* | *0.09275* | *0.09569* | *0.09928* | *0.09824* | *0.10201* |
| *29524.76652* | *0.06175* | *0.06204* | *0.06649* | *0.06831* | *0.06931* | *0.07267* | *0.07299* | *0.07523* | *0.07716* | *0.07997* | *0.08455* | *0.08781* | *0.08843* | *0.09071* | *0.09236* | *0.09528* | *0.09887* | *0.09782* | *0.10158* |
| *29517.7875* | *0.06147* | *0.06177* | *0.06619* | *0.068* | *0.069* | *0.07234* | *0.07266* | *0.0749* | *0.07683* | *0.07962* | *0.08419* | *0.08744* | *0.08805* | *0.09033* | *0.09197* | *0.09488* | *0.09847* | *0.0974* | *0.10115* |
| *29510.81177* | *0.06118* | *0.0615* | *0.06588* | *0.06769* | *0.06869* | *0.07201* | *0.07234* | *0.07457* | *0.07649* | *0.07928* | *0.08383* | *0.08707* | *0.08768* | *0.08994* | *0.09159* | *0.09448* | *0.09807* | *0.09699* | *0.10073* |
| *29503.83934* | *0.0609* | *0.06124* | *0.06558* | *0.06738* | *0.06838* | *0.07169* | *0.07203* | *0.07425* | *0.07617* | *0.07894* | *0.08348* | *0.0867* | *0.08731* | *0.08957* | *0.09121* | *0.09408* | *0.09768* | *0.09658* | *0.10031* |
| *29496.8702* | *0.06062* | *0.06098* | *0.06528* | *0.06708* | *0.06807* | *0.07137* | *0.07172* | *0.07393* | *0.07584* | *0.0786* | *0.08313* | *0.08634* | *0.08694* | *0.08919* | *0.09083* | *0.09369* | *0.09728* | *0.09617* | *0.09989* |
| *29489.90436* | *0.06034* | *0.06072* | *0.06498* | *0.06678* | *0.06777* | *0.07105* | *0.07141* | *0.07361* | *0.07552* | *0.07827* | *0.08278* | *0.08598* | *0.08657* | *0.08882* | *0.09046* | *0.09329* | *0.09689* | *0.09577* | *0.09948* |
| *29482.9418* | *0.06007* | *0.06047* | *0.06469* | *0.06648* | *0.06748* | *0.07073* | *0.0711* | *0.0733* | *0.0752* | *0.07794* | *0.08244* | *0.08562* | *0.08621* | *0.08845* | *0.09009* | *0.09291* | *0.09651* | *0.09537* | *0.09907* |
| *29475.98253* | *0.0598* | *0.06022* | *0.0644* | *0.06618* | *0.06718* | *0.07042* | *0.0708* | *0.07299* | *0.07489* | *0.07761* | *0.08209* | *0.08526* | *0.08585* | *0.08809* | *0.08972* | *0.09252* | *0.09612* | *0.09497* | *0.09866* |
| *29469.02655* | *0.05953* | *0.05997* | *0.06411* | *0.06589* | *0.06689* | *0.07012* | *0.0705* | *0.07268* | *0.07457* | *0.07729* | *0.08176* | *0.08491* | *0.08549* | *0.08773* | *0.08936* | *0.09214* | *0.09574* | *0.09458* | *0.09826* |
| *29462.07385* | *0.05927* | *0.05973* | *0.06383* | *0.0656* | *0.0666* | *0.06981* | *0.0702* | *0.07238* | *0.07427* | *0.07697* | *0.08142* | *0.08456* | *0.08514* | *0.08737* | *0.089* | *0.09176* | *0.09536* | *0.09419* | *0.09786* |
| *29455.12443* | *0.059* | *0.05948* | *0.06355* | *0.06531* | *0.06632* | *0.06951* | *0.06991* | *0.07208* | *0.07396* | *0.07665* | *0.08109* | *0.08421* | *0.08479* | *0.08702* | *0.08865* | *0.09139* | *0.09499* | *0.0938* | *0.09746* |
| *29448.17828* | *0.05875* | *0.05924* | *0.06327* | *0.06502* | *0.06603* | *0.06921* | *0.06961* | *0.07178* | *0.07366* | *0.07634* | *0.08075* | *0.08387* | *0.08444* | *0.08666* | *0.08829* | *0.09102* | *0.09461* | *0.09342* | *0.09706* |
| *29441.23541* | *0.05849* | *0.05901* | *0.06299* | *0.06474* | *0.06575* | *0.06891* | *0.06933* | *0.07148* | *0.07336* | *0.07602* | *0.08043* | *0.08353* | *0.0841* | *0.08632* | *0.08794* | *0.09065* | *0.09424* | *0.09304* | *0.09667* |
| *29434.29582* | *0.05823* | *0.05877* | *0.06272* | *0.06446* | *0.06548* | *0.06862* | *0.06904* | *0.07119* | *0.07306* | *0.07572* | *0.0801* | *0.08319* | *0.08376* | *0.08597* | *0.0876* | *0.09028* | *0.09388* | *0.09266* | *0.09628* |
| *29427.35949* | *0.05798* | *0.05854* | *0.06245* | *0.06418* | *0.0652* | *0.06833* | *0.06876* | *0.07091* | *0.07277* | *0.07541* | *0.07978* | *0.08285* | *0.08342* | *0.08563* | *0.08725* | *0.08992* | *0.09351* | *0.09229* | *0.0959* |
| *29420.42643* | *0.05773* | *0.05831* | *0.06218* | *0.06391* | *0.06493* | *0.06804* | *0.06848* | *0.07062* | *0.07248* | *0.07511* | *0.07946* | *0.08252* | *0.08309* | *0.08529* | *0.08691* | *0.08956* | *0.09315* | *0.09192* | *0.09552* |
| *29413.49664* | *0.05749* | *0.05809* | *0.06192* | *0.06364* | *0.06466* | *0.06776* | *0.06821* | *0.07034* | *0.07219* | *0.07481* | *0.07914* | *0.08219* | *0.08276* | *0.08496* | *0.08657* | *0.0892* | *0.09279* | *0.09156* | *0.09514* |
| *29406.57012* | *0.05724* | *0.05786* | *0.06165* | *0.06337* | *0.0644* | *0.06748* | *0.06793* | *0.07006* | *0.07191* | *0.07452* | *0.07883* | *0.08186* | *0.08243* | *0.08462* | *0.08624* | *0.08885* | *0.09243* | *0.09119* | *0.09476* |
| *29399.64685* | *0.057* | *0.05764* | *0.0614* | *0.0631* | *0.06414* | *0.0672* | *0.06766* | *0.06978* | *0.07163* | *0.07423* | *0.07851* | *0.08154* | *0.0821* | *0.08429* | *0.08591* | *0.0885* | *0.09208* | *0.09083* | *0.09439* |
| *29392.72684* | *0.05676* | *0.05743* | *0.06114* | *0.06284* | *0.06388* | *0.06692* | *0.0674* | *0.06951* | *0.07135* | *0.07394* | *0.0782* | *0.08121* | *0.08178* | *0.08397* | *0.08558* | *0.08815* | *0.09173* | *0.09048* | *0.09402* |
| *29385.8101* | *0.05653* | *0.05721* | *0.06089* | *0.06258* | *0.06362* | *0.06665* | *0.06713* | *0.06924* | *0.07108* | *0.07365* | *0.0779* | *0.08089* | *0.08146* | *0.08364* | *0.08526* | *0.08781* | *0.09138* | *0.09013* | *0.09365* |
| *29378.8966* | *0.05629* | *0.057* | *0.06063* | *0.06232* | *0.06337* | *0.06638* | *0.06687* | *0.06898* | *0.07081* | *0.07337* | *0.07759* | *0.08058* | *0.08115* | *0.08332* | *0.08494* | *0.08746* | *0.09103* | *0.08978* | *0.09328* |
| *29371.98636* | *0.05606* | *0.05679* | *0.06039* | *0.06206* | *0.06311* | *0.06611* | *0.06661* | *0.06871* | *0.07054* | *0.07309* | *0.07729* | *0.08026* | *0.08083* | *0.08301* | *0.08462* | *0.08713* | *0.09069* | *0.08943* | *0.09292* |
| *29365.07937* | *0.05584* | *0.05658* | *0.06014* | *0.06181* | *0.06287* | *0.06584* | *0.06636* | *0.06845* | *0.07027* | *0.07281* | *0.07699* | *0.07995* | *0.08052* | *0.08269* | *0.0843* | *0.08679* | *0.09035* | *0.08909* | *0.09256* |
| *29358.17562* | *0.05561* | *0.05638* | *0.0599* | *0.06156* | *0.06262* | *0.06558* | *0.06611* | *0.06819* | *0.07001* | *0.07253* | *0.07669* | *0.07964* | *0.08022* | *0.08238* | *0.08399* | *0.08646* | *0.09001* | *0.08875* | *0.09221* |
| *29351.27512* | *0.05539* | *0.05617* | *0.05966* | *0.06131* | *0.06238* | *0.06532* | *0.06586* | *0.06794* | *0.06975* | *0.07226* | *0.0764* | *0.07934* | *0.07991* | *0.08207* | *0.08368* | *0.08613* | *0.08967* | *0.08841* | *0.09186* |
| *29344.37786* | *0.05517* | *0.05597* | *0.05942* | *0.06107* | *0.06214* | *0.06507* | *0.06561* | *0.06768* | *0.06949* | *0.07199* | *0.07611* | *0.07903* | *0.07961* | *0.08177* | *0.08337* | *0.0858* | *0.08934* | *0.08808* | *0.0915* |
| *29337.48385* | *0.05495* | *0.05578* | *0.05918* | *0.06082* | *0.0619* | *0.06481* | *0.06536* | *0.06743* | *0.06924* | *0.07173* | *0.07582* | *0.07873* | *0.07931* | *0.08146* | *0.08307* | *0.08548* | *0.08901* | *0.08775* | *0.09116* |
| *29330.59307* | *0.05473* | *0.05558* | *0.05895* | *0.06058* | *0.06166* | *0.06456* | *0.06512* | *0.06719* | *0.06899* | *0.07146* | *0.07553* | *0.07843* | *0.07901* | *0.08116* | *0.08277* | *0.08516* | *0.08868* | *0.08742* | *0.09081* |
| *29323.70553* | *0.05452* | *0.05539* | *0.05872* | *0.06034* | *0.06143* | *0.06431* | *0.06488* | *0.06694* | *0.06874* | *0.0712* | *0.07524* | *0.07814* | *0.07872* | *0.08087* | *0.08247* | *0.08484* | *0.08835* | *0.0871* | *0.09047* |
| *29316.82122* | *0.0543* | *0.0552* | *0.05849* | *0.06011* | *0.0612* | *0.06407* | *0.06465* | *0.0667* | *0.06849* | *0.07095* | *0.07496* | *0.07784* | *0.07843* | *0.08057* | *0.08218* | *0.08452* | *0.08803* | *0.08678* | *0.09013* |
| *29309.94015* | *0.0541* | *0.05501* | *0.05827* | *0.05987* | *0.06097* | *0.06382* | *0.06441* | *0.06646* | *0.06825* | *0.07069* | *0.07468* | *0.07755* | *0.07814* | *0.08028* | *0.08189* | *0.08421* | *0.08771* | *0.08646* | *0.0898* |
| *29303.0623* | *0.05389* | *0.05483* | *0.05804* | *0.05964* | *0.06075* | *0.06358* | *0.06418* | *0.06623* | *0.06801* | *0.07044* | *0.07441* | *0.07726* | *0.07786* | *0.07999* | *0.0816* | *0.0839* | *0.08739* | *0.08615* | *0.08946* |
| *29296.18768* | *0.05368* | *0.05464* | *0.05782* | *0.05941* | *0.06052* | *0.06334* | *0.06395* | *0.06599* | *0.06777* | *0.07019* | *0.07413* | *0.07698* | *0.07757* | *0.07971* | *0.08131* | *0.08359* | *0.08708* | *0.08583* | *0.08913* |
| *29289.31629* | *0.05348* | *0.05446* | *0.0576* | *0.05918* | *0.0603* | *0.06311* | *0.06373* | *0.06576* | *0.06754* | *0.06994* | *0.07386* | *0.07669* | *0.07729* | *0.07943* | *0.08103* | *0.08329* | *0.08676* | *0.08553* | *0.0888* |
| *29282.44812* | *0.05328* | *0.05428* | *0.05739* | *0.05896* | *0.06008* | *0.06287* | *0.06351* | *0.06553* | *0.06731* | *0.0697* | *0.07359* | *0.07641* | *0.07702* | *0.07915* | *0.08075* | *0.08299* | *0.08645* | *0.08522* | *0.08848* |
| *29275.58317* | *0.05308* | *0.05411* | *0.05717* | *0.05874* | *0.05987* | *0.06264* | *0.06328* | *0.06531* | *0.06708* | *0.06946* | *0.07332* | *0.07613* | *0.07674* | *0.07887* | *0.08047* | *0.08269* | *0.08614* | *0.08492* | *0.08816* |
| *29268.72143* | *0.05289* | *0.05393* | *0.05696* | *0.05852* | *0.05966* | *0.06241* | *0.06307* | *0.06508* | *0.06685* | *0.06922* | *0.07305* | *0.07586* | *0.07647* | *0.07859* | *0.0802* | *0.08239* | *0.08584* | *0.08462* | *0.08784* |
| *29261.86292* | *0.05269* | *0.05376* | *0.05675* | *0.0583* | *0.05944* | *0.06219* | *0.06285* | *0.06486* | *0.06662* | *0.06898* | *0.07279* | *0.07558* | *0.0762* | *0.07832* | *0.07992* | *0.0821* | *0.08553* | *0.08432* | *0.08752* |
| *29255.00761* | *0.0525* | *0.05359* | *0.05655* | *0.05809* | *0.05924* | *0.06196* | *0.06264* | *0.06465* | *0.0664* | *0.06875* | *0.07253* | *0.07531* | *0.07594* | *0.07805* | *0.07965* | *0.08181* | *0.08523* | *0.08403* | *0.0872* |
| *29248.15552* | *0.05231* | *0.05343* | *0.05634* | *0.05787* | *0.05903* | *0.06174* | *0.06243* | *0.06443* | *0.06618* | *0.06852* | *0.07227* | *0.07504* | *0.07567* | *0.07779* | *0.07939* | *0.08152* | *0.08493* | *0.08374* | *0.08689* |
| *29241.30664* | *0.05213* | *0.05326* | *0.05614* | *0.05766* | *0.05883* | *0.06152* | *0.06222* | *0.06422* | *0.06597* | *0.06829* | *0.07201* | *0.07478* | *0.07541* | *0.07752* | *0.07912* | *0.08123* | *0.08463* | *0.08345* | *0.08658* |
| *29234.46096* | *0.05194* | *0.0531* | *0.05594* | *0.05745* | *0.05863* | *0.06131* | *0.06201* | *0.06401* | *0.06575* | *0.06806* | *0.07176* | *0.07451* | *0.07515* | *0.07726* | *0.07886* | *0.08095* | *0.08434* | *0.08316* | *0.08628* |
| *29227.61849* | *0.05176* | *0.05294* | *0.05574* | *0.05725* | *0.05843* | *0.06109* | *0.06181* | *0.0638* | *0.06554* | *0.06784* | *0.07151* | *0.07425* | *0.07489* | *0.077* | *0.0786* | *0.08067* | *0.08405* | *0.08288* | *0.08597* |
| *29220.77922* | *0.05158* | *0.05278* | *0.05555* | *0.05704* | *0.05823* | *0.06088* | *0.06161* | *0.06359* | *0.06533* | *0.06762* | *0.07126* | *0.07399* | *0.07464* | *0.07675* | *0.07835* | *0.08039* | *0.08376* | *0.0826* | *0.08567* |
| *29213.94315* | *0.0514* | *0.05262* | *0.05536* | *0.05684* | *0.05803* | *0.06067* | *0.06141* | *0.06339* | *0.06512* | *0.0674* | *0.07101* | *0.07373* | *0.07439* | *0.07649* | *0.07809* | *0.08012* | *0.08347* | *0.08232* | *0.08537* |
| *29207.11028* | *0.05122* | *0.05247* | *0.05516* | *0.05664* | *0.05784* | *0.06046* | *0.06121* | *0.06319* | *0.06492* | *0.06718* | *0.07076* | *0.07348* | *0.07414* | *0.07624* | *0.07784* | *0.07984* | *0.08319* | *0.08205* | *0.08508* |
| *29200.2806* | *0.05105* | *0.05231* | *0.05498* | *0.05644* | *0.05765* | *0.06026* | *0.06102* | *0.06299* | *0.06472* | *0.06697* | *0.07052* | *0.07323* | *0.07389* | *0.07599* | *0.07759* | *0.07957* | *0.0829* | *0.08178* | *0.08478* |
| *29193.45412* | *0.05088* | *0.05216* | *0.05479* | *0.05624* | *0.05746* | *0.06006* | *0.06082* | *0.06279* | *0.06452* | *0.06676* | *0.07028* | *0.07297* | *0.07365* | *0.07575* | *0.07735* | *0.07931* | *0.08262* | *0.08151* | *0.08449* |
| *29186.63083* | *0.05071* | *0.05201* | *0.0546* | *0.05605* | *0.05728* | *0.05985* | *0.06063* | *0.0626* | *0.06432* | *0.06655* | *0.07004* | *0.07273* | *0.07341* | *0.0755* | *0.07711* | *0.07904* | *0.08234* | *0.08124* | *0.0842* |
| *29179.81073* | *0.05054* | *0.05187* | *0.05442* | *0.05586* | *0.0571* | *0.05966* | *0.06045* | *0.06241* | *0.06412* | *0.06634* | *0.0698* | *0.07248* | *0.07317* | *0.07526* | *0.07687* | *0.07878* | *0.08207* | *0.08098* | *0.08392* |
| *29172.99381* | *0.05037* | *0.05172* | *0.05424* | *0.05567* | *0.05691* | *0.05946* | *0.06026* | *0.06222* | *0.06393* | *0.06613* | *0.06957* | *0.07224* | *0.07293* | *0.07503* | *0.07663* | *0.07852* | *0.08179* | *0.08072* | *0.08363* |
| *29166.18008* | *0.05021* | *0.05158* | *0.05406* | *0.05548* | *0.05673* | *0.05927* | *0.06008* | *0.06203* | *0.06374* | *0.06593* | *0.06934* | *0.07199* | *0.0727* | *0.07479* | *0.07639* | *0.07826* | *0.08152* | *0.08046* | *0.08335* |
| *29159.36953* | *0.05004* | *0.05144* | *0.05389* | *0.0553* | *0.05656* | *0.05907* | *0.0599* | *0.06185* | *0.06355* | *0.06573* | *0.06911* | *0.07176* | *0.07247* | *0.07456* | *0.07616* | *0.07801* | *0.08125* | *0.0802* | *0.08307* |
| *29152.56216* | *0.04988* | *0.0513* | *0.05371* | *0.05511* | *0.05638* | *0.05889* | *0.05972* | *0.06166* | *0.06336* | *0.06553* | *0.06888* | *0.07152* | *0.07224* | *0.07432* | *0.07593* | *0.07775* | *0.08098* | *0.07995* | *0.08279* |
| *29145.75796* | *0.04972* | *0.05116* | *0.05354* | *0.05493* | *0.05621* | *0.0587* | *0.05954* | *0.06148* | *0.06318* | *0.06534* | *0.06865* | *0.07128* | *0.07201* | *0.0741* | *0.0757* | *0.0775* | *0.08072* | *0.0797* | *0.08252* |
| *29138.95695* | *0.04957* | *0.05102* | *0.05337* | *0.05475* | *0.05604* | *0.05851* | *0.05937* | *0.0613* | *0.063* | *0.06515* | *0.06843* | *0.07105* | *0.07179* | *0.07387* | *0.07547* | *0.07725* | *0.08046* | *0.07945* | *0.08225* |
| *29132.1591* | *0.04941* | *0.05089* | *0.0532* | *0.05457* | *0.05587* | *0.05833* | *0.05919* | *0.06113* | *0.06282* | *0.06495* | *0.0682* | *0.07082* | *0.07156* | *0.07364* | *0.07525* | *0.07701* | *0.0802* | *0.07921* | *0.08198* |
| *29125.36443* | *0.04926* | *0.05076* | *0.05304* | *0.0544* | *0.0557* | *0.05815* | *0.05902* | *0.06095* | *0.06264* | *0.06477* | *0.06798* | *0.07059* | *0.07134* | *0.07342* | *0.07503* | *0.07677* | *0.07994* | *0.07897* | *0.08171* |
| *29118.57293* | *0.04911* | *0.05063* | *0.05287* | *0.05422* | *0.05554* | *0.05797* | *0.05885* | *0.06078* | *0.06246* | *0.06458* | *0.06776* | *0.07036* | *0.07113* | *0.0732* | *0.07481* | *0.07652* | *0.07968* | *0.07873* | *0.08145* |
| *29111.78459* | *0.04896* | *0.0505* | *0.05271* | *0.05405* | *0.05537* | *0.05779* | *0.05869* | *0.06061* | *0.06229* | *0.06439* | *0.06755* | *0.07014* | *0.07091* | *0.07299* | *0.07459* | *0.07629* | *0.07942* | *0.07849* | *0.08119* |
| *29104.99942* | *0.04881* | *0.05037* | *0.05255* | *0.05388* | *0.05521* | *0.05762* | *0.05852* | *0.06044* | *0.06212* | *0.06421* | *0.06733* | *0.06992* | *0.0707* | *0.07277* | *0.07438* | *0.07605* | *0.07917* | *0.07825* | *0.08093* |
| *29098.21741* | *0.04866* | *0.05025* | *0.05239* | *0.05371* | *0.05505* | *0.05744* | *0.05836* | *0.06028* | *0.06195* | *0.06403* | *0.06712* | *0.0697* | *0.07048* | *0.07256* | *0.07417* | *0.07582* | *0.07892* | *0.07802* | *0.08067* |
| *29091.43856* | *0.04852* | *0.05012* | *0.05223* | *0.05355* | *0.05489* | *0.05727* | *0.0582* | *0.06011* | *0.06178* | *0.06385* | *0.06691* | *0.06948* | *0.07028* | *0.07235* | *0.07396* | *0.07558* | *0.07867* | *0.07779* | *0.08041* |
| *29084.66286* | *0.04838* | *0.05* | *0.05208* | *0.05338* | *0.05474* | *0.0571* | *0.05804* | *0.05995* | *0.06162* | *0.06368* | *0.0667* | *0.06926* | *0.07007* | *0.07214* | *0.07375* | *0.07535* | *0.07843* | *0.07756* | *0.08016* |
| *29077.89032* | *0.04823* | *0.04988* | *0.05192* | *0.05322* | *0.05459* | *0.05694* | *0.05789* | *0.05979* | *0.06145* | *0.0635* | *0.0665* | *0.06905* | *0.06986* | *0.07193* | *0.07354* | *0.07513* | *0.07818* | *0.07734* | *0.07991* |
| *29071.12094* | *0.0481* | *0.04976* | *0.05177* | *0.05306* | *0.05443* | *0.05677* | *0.05773* | *0.05963* | *0.06129* | *0.06333* | *0.06629* | *0.06884* | *0.06966* | *0.07173* | *0.07334* | *0.0749* | *0.07794* | *0.07711* | *0.07966* |
| *29064.35471* | *0.04796* | *0.04965* | *0.05162* | *0.0529* | *0.05428* | *0.05661* | *0.05758* | *0.05948* | *0.06113* | *0.06316* | *0.06609* | *0.06863* | *0.06946* | *0.07153* | *0.07314* | *0.07468* | *0.0777* | *0.07689* | *0.07941* |
| *29057.59162* | *0.04782* | *0.04953* | *0.05148* | *0.05274* | *0.05414* | *0.05645* | *0.05743* | *0.05932* | *0.06098* | *0.06299* | *0.06589* | *0.06842* | *0.06926* | *0.07133* | *0.07294* | *0.07446* | *0.07746* | *0.07667* | *0.07917* |
| *29050.83169* | *0.04769* | *0.04942* | *0.05133* | *0.05258* | *0.05399* | *0.05629* | *0.05728* | *0.05917* | *0.06082* | *0.06283* | *0.06569* | *0.06821* | *0.06907* | *0.07113* | *0.07274* | *0.07424* | *0.07723* | *0.07646* | *0.07893* |
| *29044.07489* | *0.04755* | *0.04931* | *0.05119* | *0.05243* | *0.05384* | *0.05613* | *0.05713* | *0.05902* | *0.06067* | *0.06266* | *0.06549* | *0.06801* | *0.06887* | *0.07094* | *0.07255* | *0.07402* | *0.07699* | *0.07624* | *0.07869* |
| *29037.32124* | *0.04742* | *0.0492* | *0.05104* | *0.05228* | *0.0537* | *0.05597* | *0.05699* | *0.05887* | *0.06052* | *0.0625* | *0.0653* | *0.0678* | *0.06868* | *0.07074* | *0.07236* | *0.07381* | *0.07676* | *0.07603* | *0.07845* |
| *29030.57073* | *0.04729* | *0.04909* | *0.0509* | *0.05213* | *0.05356* | *0.05582* | *0.05684* | *0.05872* | *0.06037* | *0.06234* | *0.0651* | *0.0676* | *0.06849* | *0.07055* | *0.07217* | *0.0736* | *0.07653* | *0.07582* | *0.07822* |
| *29023.82336* | *0.04717* | *0.04898* | *0.05076* | *0.05198* | *0.05342* | *0.05567* | *0.0567* | *0.05858* | *0.06022* | *0.06218* | *0.06491* | *0.06741* | *0.0683* | *0.07036* | *0.07198* | *0.07339* | *0.0763* | *0.07562* | *0.07798* |
| *29017.07912* | *0.04704* | *0.04887* | *0.05063* | *0.05183* | *0.05328* | *0.05552* | *0.05656* | *0.05844* | *0.06007* | *0.06203* | *0.06472* | *0.06721* | *0.06811* | *0.07018* | *0.07179* | *0.07318* | *0.07607* | *0.07541* | *0.07775* |
| *29010.33802* | *0.04692* | *0.04877* | *0.05049* | *0.05169* | *0.05315* | *0.05537* | *0.05642* | *0.0583* | *0.05993* | *0.06187* | *0.06453* | *0.06701* | *0.06793* | *0.06999* | *0.07161* | *0.07297* | *0.07585* | *0.07521* | *0.07752* |
| *29003.60005* | *0.04679* | *0.04867* | *0.05036* | *0.05154* | *0.05301* | *0.05522* | *0.05629* | *0.05816* | *0.05979* | *0.06172* | *0.06435* | *0.06682* | *0.06775* | *0.06981* | *0.07143* | *0.07277* | *0.07563* | *0.07501* | *0.0773* |
| *28996.8652* | *0.04667* | *0.04857* | *0.05022* | *0.0514* | *0.05288* | *0.05507* | *0.05615* | *0.05802* | *0.05965* | *0.06157* | *0.06416* | *0.06663* | *0.06757* | *0.06963* | *0.07125* | *0.07257* | *0.07541* | *0.07481* | *0.07707* |
| *28990.13349* | *0.04655* | *0.04846* | *0.05009* | *0.05126* | *0.05275* | *0.05493* | *0.05602* | *0.05788* | *0.05951* | *0.06142* | *0.06398* | *0.06644* | *0.06739* | *0.06945* | *0.07107* | *0.07237* | *0.07519* | *0.07461* | *0.07685* |
| *28983.4049* | *0.04643* | *0.04837* | *0.04996* | *0.05112* | *0.05262* | *0.05479* | *0.05589* | *0.05775* | *0.05937* | *0.06127* | *0.0638* | *0.06625* | *0.06721* | *0.06927* | *0.07089* | *0.07217* | *0.07497* | *0.07442* | *0.07663* |
| *28976.67943* | *0.04631* | *0.04827* | *0.04984* | *0.05098* | *0.05249* | *0.05465* | *0.05576* | *0.05762* | *0.05924* | *0.06112* | *0.06362* | *0.06607* | *0.06704* | *0.0691* | *0.07072* | *0.07198* | *0.07475* | *0.07423* | *0.07641* |
| *28969.95708* | *0.0462* | *0.04817* | *0.04971* | *0.05085* | *0.05237* | *0.05451* | *0.05563* | *0.05748* | *0.0591* | *0.06098* | *0.06344* | *0.06588* | *0.06686* | *0.06892* | *0.07055* | *0.07178* | *0.07454* | *0.07404* | *0.07619* |
| *28963.23785* | *0.04608* | *0.04808* | *0.04958* | *0.05071* | *0.05224* | *0.05437* | *0.0555* | *0.05736* | *0.05897* | *0.06084* | *0.06327* | *0.0657* | *0.06669* | *0.06875* | *0.07038* | *0.07159* | *0.07433* | *0.07385* | *0.07598* |
| *28956.52174* | *0.04597* | *0.04798* | *0.04946* | *0.05058* | *0.05212* | *0.05424* | *0.05538* | *0.05723* | *0.05884* | *0.0607* | *0.06309* | *0.06552* | *0.06652* | *0.06858* | *0.07021* | *0.0714* | *0.07412* | *0.07366* | *0.07577* |
| *28949.80874* | *0.04586* | *0.04789* | *0.04934* | *0.05045* | *0.052* | *0.05411* | *0.05526* | *0.0571* | *0.05871* | *0.06056* | *0.06292* | *0.06534* | *0.06636* | *0.06841* | *0.07004* | *0.07121* | *0.07391* | *0.07348* | *0.07556* |
| *28943.09885* | *0.04575* | *0.0478* | *0.04922* | *0.05032* | *0.05188* | *0.05397* | *0.05513* | *0.05698* | *0.05859* | *0.06042* | *0.06275* | *0.06517* | *0.06619* | *0.06825* | *0.06988* | *0.07103* | *0.07371* | *0.0733* | *0.07535* |
| *28936.39208* | *0.04564* | *0.04771* | *0.0491* | *0.05019* | *0.05176* | *0.05384* | *0.05501* | *0.05685* | *0.05846* | *0.06029* | *0.06258* | *0.06499* | *0.06603* | *0.06808* | *0.06971* | *0.07084* | *0.0735* | *0.07312* | *0.07514* |
| *28929.6884* | *0.04553* | *0.04762* | *0.04898* | *0.05006* | *0.05164* | *0.05372* | *0.0549* | *0.05673* | *0.05834* | *0.06015* | *0.06241* | *0.06482* | *0.06587* | *0.06792* | *0.06955* | *0.07066* | *0.0733* | *0.07294* | *0.07494* |
| *28922.98784* | *0.04543* | *0.04754* | *0.04887* | *0.04994* | *0.05153* | *0.05359* | *0.05478* | *0.05661* | *0.05822* | *0.06002* | *0.06225* | *0.06465* | *0.06571* | *0.06776* | *0.06939* | *0.07048* | *0.0731* | *0.07277* | *0.07473* |
| *28916.29038* | *0.04532* | *0.04745* | *0.04876* | *0.04982* | *0.05141* | *0.05346* | *0.05466* | *0.0565* | *0.05809* | *0.05989* | *0.06208* | *0.06448* | *0.06555* | *0.0676* | *0.06924* | *0.0703* | *0.0729* | *0.07259* | *0.07453* |
| *28909.59602* | *0.04522* | *0.04737* | *0.04864* | *0.04969* | *0.0513* | *0.05334* | *0.05455* | *0.05638* | *0.05798* | *0.05976* | *0.06192* | *0.06431* | *0.06539* | *0.06745* | *0.06908* | *0.07013* | *0.0727* | *0.07242* | *0.07433* |
| *28902.90476* | *0.04512* | *0.04728* | *0.04853* | *0.04957* | *0.05119* | *0.05322* | *0.05444* | *0.05627* | *0.05786* | *0.05964* | *0.06176* | *0.06414* | *0.06524* | *0.06729* | *0.06893* | *0.06995* | *0.07251* | *0.07225* | *0.07414* |
| *28896.21659* | *0.04502* | *0.0472* | *0.04842* | *0.04945* | *0.05108* | *0.0531* | *0.05433* | *0.05615* | *0.05774* | *0.05951* | *0.0616* | *0.06398* | *0.06509* | *0.06714* | *0.06878* | *0.06978* | *0.07231* | *0.07208* | *0.07394* |
| *28889.53152* | *0.04492* | *0.04712* | *0.04831* | *0.04933* | *0.05097* | *0.05298* | *0.05422* | *0.05604* | *0.05763* | *0.05939* | *0.06144* | *0.06382* | *0.06493* | *0.06699* | *0.06863* | *0.06961* | *0.07212* | *0.07192* | *0.07375* |
| *28882.84954* | *0.04482* | *0.04704* | *0.04821* | *0.04922* | *0.05086* | *0.05286* | *0.05411* | *0.05593* | *0.05752* | *0.05926* | *0.06128* | *0.06366* | *0.06478* | *0.06684* | *0.06848* | *0.06944* | *0.07193* | *0.07175* | *0.07356* |
| *28876.17066* | *0.04472* | *0.04696* | *0.0481* | *0.0491* | *0.05076* | *0.05274* | *0.054* | *0.05582* | *0.0574* | *0.05914* | *0.06113* | *0.0635* | *0.06464* | *0.06669* | *0.06833* | *0.06927* | *0.07174* | *0.07159* | *0.07337* |
| *28869.49486* | *0.04463* | *0.04689* | *0.048* | *0.04899* | *0.05066* | *0.05263* | *0.0539* | *0.05571* | *0.05729* | *0.05902* | *0.06098* | *0.06334* | *0.06449* | *0.06654* | *0.06819* | *0.06911* | *0.07155* | *0.07143* | *0.07318* |
| *28862.82214* | *0.04454* | *0.04681* | *0.04789* | *0.04888* | *0.05055* | *0.05251* | *0.05379* | *0.05561* | *0.05719* | *0.05891* | *0.06082* | *0.06318* | *0.06435* | *0.0664* | *0.06804* | *0.06894* | *0.07137* | *0.07127* | *0.073* |
| *28856.15251* | *0.04444* | *0.04674* | *0.04779* | *0.04876* | *0.05045* | *0.0524* | *0.05369* | *0.0555* | *0.05708* | *0.05879* | *0.06067* | *0.06303* | *0.0642* | *0.06626* | *0.0679* | *0.06878* | *0.07118* | *0.07111* | *0.07281* |
| *28849.48597* | *0.04435* | *0.04666* | *0.04769* | *0.04865* | *0.05035* | *0.05229* | *0.05359* | *0.0554* | *0.05697* | *0.05868* | *0.06053* | *0.06287* | *0.06406* | *0.06612* | *0.06776* | *0.06862* | *0.071* | *0.07096* | *0.07263* |
| *28842.8225* | *0.04426* | *0.04659* | *0.04759* | *0.04855* | *0.05025* | *0.05218* | *0.05349* | *0.0553* | *0.05687* | *0.05856* | *0.06038* | *0.06272* | *0.06392* | *0.06598* | *0.06762* | *0.06846* | *0.07082* | *0.07081* | *0.07245* |
| *28836.16211* | *0.04417* | *0.04652* | *0.04749* | *0.04844* | *0.05015* | *0.05207* | *0.05339* | *0.0552* | *0.05677* | *0.05845* | *0.06023* | *0.06257* | *0.06378* | *0.06584* | *0.06749* | *0.06831* | *0.07064* | *0.07065* | *0.07227* |
| *28829.50479* | *0.04408* | *0.04645* | *0.0474* | *0.04833* | *0.05006* | *0.05197* | *0.05329* | *0.0551* | *0.05667* | *0.05834* | *0.06009* | *0.06242* | *0.06365* | *0.0657* | *0.06735* | *0.06815* | *0.07047* | *0.0705* | *0.0721* |
| *28822.85055* | *0.044* | *0.04638* | *0.0473* | *0.04823* | *0.04996* | *0.05186* | *0.0532* | *0.055* | *0.05657* | *0.05823* | *0.05995* | *0.06227* | *0.06351* | *0.06557* | *0.06722* | *0.068* | *0.07029* | *0.07036* | *0.07192* |
| *28816.19938* | *0.04391* | *0.04631* | *0.04721* | *0.04812* | *0.04987* | *0.05176* | *0.0531* | *0.0549* | *0.05647* | *0.05812* | *0.0598* | *0.06213* | *0.06338* | *0.06543* | *0.06709* | *0.06785* | *0.07012* | *0.07021* | *0.07175* |
| *28809.55127* | *0.04382* | *0.04624* | *0.04711* | *0.04802* | *0.04978* | *0.05165* | *0.05301* | *0.05481* | *0.05637* | *0.05802* | *0.05966* | *0.06198* | *0.06325* | *0.0653* | *0.06696* | *0.0677* | *0.06994* | *0.07006* | *0.07158* |
| *28802.90624* | *0.04374* | *0.04617* | *0.04702* | *0.04792* | *0.04969* | *0.05155* | *0.05292* | *0.05471* | *0.05627* | *0.05791* | *0.05953* | *0.06184* | *0.06312* | *0.06517* | *0.06683* | *0.06755* | *0.06977* | *0.06992* | *0.07141* |
| *28796.26427* | *0.04366* | *0.04611* | *0.04693* | *0.04782* | *0.0496* | *0.05145* | *0.05283* | *0.05462* | *0.05618* | *0.05781* | *0.05939* | *0.0617* | *0.06299* | *0.06504* | *0.0667* | *0.0674* | *0.0696* | *0.06978* | *0.07124* |
| *28789.62536* | *0.04358* | *0.04604* | *0.04684* | *0.04772* | *0.04951* | *0.05135* | *0.05274* | *0.05453* | *0.05609* | *0.05771* | *0.05925* | *0.06156* | *0.06286* | *0.06492* | *0.06658* | *0.06725* | *0.06944* | *0.06964* | *0.07107* |
| *28782.98951* | *0.0435* | *0.04598* | *0.04676* | *0.04763* | *0.04942* | *0.05126* | *0.05265* | *0.05444* | *0.05599* | *0.0576* | *0.05912* | *0.06142* | *0.06273* | *0.06479* | *0.06645* | *0.06711* | *0.06927* | *0.0695* | *0.07091* |
| *28776.35672* | *0.04342* | *0.04592* | *0.04667* | *0.04753* | *0.04933* | *0.05116* | *0.05256* | *0.05435* | *0.0559* | *0.0575* | *0.05899* | *0.06129* | *0.06261* | *0.06467* | *0.06633* | *0.06697* | *0.06911* | *0.06937* | *0.07074* |
| *28769.72699* | *0.04334* | *0.04586* | *0.04658* | *0.04744* | *0.04925* | *0.05106* | *0.05248* | *0.05426* | *0.05581* | *0.05741* | *0.05885* | *0.06115* | *0.06249* | *0.06454* | *0.06621* | *0.06683* | *0.06894* | *0.06923* | *0.07058* |
| *28763.10031* | *0.04326* | *0.0458* | *0.0465* | *0.04734* | *0.04916* | *0.05097* | *0.05239* | *0.05417* | *0.05572* | *0.05731* | *0.05872* | *0.06102* | *0.06237* | *0.06442* | *0.06609* | *0.06669* | *0.06878* | *0.0691* | *0.07042* |
| *28756.47668* | *0.04319* | *0.04574* | *0.04641* | *0.04725* | *0.04908* | *0.05088* | *0.05231* | *0.05409* | *0.05564* | *0.05721* | *0.05859* | *0.06088* | *0.06224* | *0.0643* | *0.06597* | *0.06655* | *0.06862* | *0.06897* | *0.07026* |
| *28749.85611* | *0.04311* | *0.04568* | *0.04633* | *0.04716* | *0.049* | *0.05079* | *0.05223* | *0.05401* | *0.05555* | *0.05712* | *0.05847* | *0.06075* | *0.06213* | *0.06418* | *0.06585* | *0.06641* | *0.06846* | *0.06884* | *0.07011* |
| *28743.23858* | *0.04304* | *0.04562* | *0.04625* | *0.04707* | *0.04892* | *0.0507* | *0.05214* | *0.05392* | *0.05547* | *0.05703* | *0.05834* | *0.06062* | *0.06201* | *0.06407* | *0.06574* | *0.06628* | *0.0683* | *0.06871* | *0.06995* |
| *28736.62409* | *0.04296* | *0.04556* | *0.04617* | *0.04698* | *0.04884* | *0.05061* | *0.05206* | *0.05384* | *0.05538* | *0.05693* | *0.05822* | *0.0605* | *0.06189* | *0.06395* | *0.06563* | *0.06615* | *0.06815* | *0.06858* | *0.0698* |
| *28730.01265* | *0.04289* | *0.04551* | *0.04609* | *0.04689* | *0.04876* | *0.05052* | *0.05198* | *0.05376* | *0.0553* | *0.05684* | *0.05809* | *0.06037* | *0.06178* | *0.06384* | *0.06551* | *0.06602* | *0.06799* | *0.06846* | *0.06965* |
| *28723.40426* | *0.04282* | *0.04545* | *0.04601* | *0.0468* | *0.04868* | *0.05043* | *0.05191* | *0.05368* | *0.05522* | *0.05675* | *0.05797* | *0.06024* | *0.06166* | *0.06372* | *0.0654* | *0.06588* | *0.06784* | *0.06833* | *0.0695* |
| *28716.7989* | *0.04275* | *0.0454* | *0.04594* | *0.04672* | *0.04861* | *0.05035* | *0.05183* | *0.0536* | *0.05514* | *0.05666* | *0.05785* | *0.06012* | *0.06155* | *0.06361* | *0.06529* | *0.06576* | *0.06769* | *0.06821* | *0.06935* |
| *28710.19657* | *0.04268* | *0.04534* | *0.04586* | *0.04663* | *0.04853* | *0.05026* | *0.05175* | *0.05352* | *0.05506* | *0.05658* | *0.05773* | *0.06* | *0.06144* | *0.0635* | *0.06518* | *0.06563* | *0.06754* | *0.06809* | *0.0692* |
| *28703.59729* | *0.04261* | *0.04529* | *0.04578* | *0.04655* | *0.04846* | *0.05018* | *0.05168* | *0.05345* | *0.05498* | *0.05649* | *0.05761* | *0.05987* | *0.06133* | *0.06339* | *0.06508* | *0.0655* | *0.06739* | *0.06797* | *0.06906* |
| *28697.00103* | *0.04254* | *0.04524* | *0.04571* | *0.04647* | *0.04838* | *0.0501* | *0.05161* | *0.05337* | *0.0549* | *0.05641* | *0.05749* | *0.05975* | *0.06123* | *0.06329* | *0.06497* | *0.06538* | *0.06724* | *0.06785* | *0.06891* |
| *28690.40781* | *0.04248* | *0.04519* | *0.04564* | *0.04638* | *0.04831* | *0.05001* | *0.05153* | *0.0533* | *0.05483* | *0.05632* | *0.05738* | *0.05963* | *0.06112* | *0.06318* | *0.06487* | *0.06526* | *0.0671* | *0.06773* | *0.06877* |
| *28683.81762* | *0.04241* | *0.04514* | *0.04557* | *0.0463* | *0.04824* | *0.04993* | *0.05146* | *0.05322* | *0.05475* | *0.05624* | *0.05726* | *0.05952* | *0.06101* | *0.06308* | *0.06476* | *0.06513* | *0.06695* | *0.06762* | *0.06863* |
| *28677.23045* | *0.04235* | *0.04509* | *0.04549* | *0.04622* | *0.04817* | *0.04986* | *0.05139* | *0.05315* | *0.05468* | *0.05616* | *0.05715* | *0.0594* | *0.06091* | *0.06297* | *0.06466* | *0.06501* | *0.06681* | *0.0675* | *0.06849* |
| *28670.64631* | *0.04228* | *0.04504* | *0.04542* | *0.04615* | *0.0481* | *0.04978* | *0.05132* | *0.05308* | *0.05461* | *0.05608* | *0.05704* | *0.05928* | *0.06081* | *0.06287* | *0.06456* | *0.06489* | *0.06667* | *0.06739* | *0.06835* |
| *28664.06519* | *0.04222* | *0.04499* | *0.04536* | *0.04607* | *0.04803* | *0.0497* | *0.05125* | *0.05301* | *0.05453* | *0.056* | *0.05693* | *0.05917* | *0.0607* | *0.06277* | *0.06446* | *0.06478* | *0.06653* | *0.06728* | *0.06821* |
| *28657.48709* | *0.04216* | *0.04494* | *0.04529* | *0.04599* | *0.04796* | *0.04962* | *0.05118* | *0.05294* | *0.05446* | *0.05592* | *0.05682* | *0.05906* | *0.0606* | *0.06267* | *0.06436* | *0.06466* | *0.06639* | *0.06717* | *0.06808* |
| *28650.91201* | *0.0421* | *0.04489* | *0.04522* | *0.04592* | *0.0479* | *0.04955* | *0.05112* | *0.05287* | *0.05439* | *0.05584* | *0.05671* | *0.05895* | *0.0605* | *0.06257* | *0.06427* | *0.06455* | *0.06625* | *0.06706* | *0.06794* |
| *28644.33995* | *0.04204* | *0.04485* | *0.04515* | *0.04584* | *0.04783* | *0.04948* | *0.05105* | *0.05281* | *0.05432* | *0.05576* | *0.0566* | *0.05884* | *0.06041* | *0.06247* | *0.06417* | *0.06443* | *0.06612* | *0.06695* | *0.06781* |
| *28637.7709* | *0.04198* | *0.0448* | *0.04509* | *0.04577* | *0.04777* | *0.0494* | *0.05099* | *0.05274* | *0.05426* | *0.05569* | *0.05649* | *0.05873* | *0.06031* | *0.06238* | *0.06408* | *0.06432* | *0.06598* | *0.06685* | *0.06768* |
| *28631.20486* | *0.04192* | *0.04476* | *0.04502* | *0.04569* | *0.0477* | *0.04933* | *0.05092* | *0.05267* | *0.05419* | *0.05562* | *0.05639* | *0.05862* | *0.06021* | *0.06228* | *0.06398* | *0.06421* | *0.06585* | *0.06674* | *0.06755* |
| *28624.64183* | *0.04186* | *0.04472* | *0.04496* | *0.04562* | *0.04764* | *0.04926* | *0.05086* | *0.05261* | *0.05412* | *0.05554* | *0.05628* | *0.05851* | *0.06012* | *0.06219* | *0.06389* | *0.0641* | *0.06572* | *0.06664* | *0.06742* |
| *28618.08182* | *0.04181* | *0.04467* | *0.0449* | *0.04555* | *0.04758* | *0.04919* | *0.0508* | *0.05255* | *0.05406* | *0.05547* | *0.05618* | *0.05841* | *0.06003* | *0.0621* | *0.0638* | *0.06399* | *0.06558* | *0.06654* | *0.06729* |
| *28611.5248* | *0.04175* | *0.04463* | *0.04484* | *0.04548* | *0.04752* | *0.04912* | *0.05074* | *0.05248* | *0.05399* | *0.0554* | *0.05608* | *0.0583* | *0.05993* | *0.062* | *0.06371* | *0.06388* | *0.06545* | *0.06644* | *0.06717* |
| *28604.97079* | *0.04169* | *0.04459* | *0.04477* | *0.04541* | *0.04746* | *0.04905* | *0.05068* | *0.05242* | *0.05393* | *0.05533* | *0.05598* | *0.0582* | *0.05984* | *0.06191* | *0.06362* | *0.06378* | *0.06533* | *0.06634* | *0.06704* |
| *28598.41979* | *0.04164* | *0.04455* | *0.04471* | *0.04534* | *0.0474* | *0.04899* | *0.05062* | *0.05236* | *0.05387* | *0.05526* | *0.05588* | *0.0581* | *0.05975* | *0.06182* | *0.06353* | *0.06367* | *0.0652* | *0.06624* | *0.06692* |
| *28591.87178* | *0.04159* | *0.04451* | *0.04465* | *0.04528* | *0.04734* | *0.04892* | *0.05056* | *0.0523* | *0.05381* | *0.05519* | *0.05578* | *0.058* | *0.05966* | *0.06174* | *0.06345* | *0.06357* | *0.06507* | *0.06614* | *0.0668* |
| *28585.32677* | *0.04153* | *0.04447* | *0.0446* | *0.04521* | *0.04728* | *0.04885* | *0.0505* | *0.05224* | *0.05375* | *0.05512* | *0.05568* | *0.0579* | *0.05958* | *0.06165* | *0.06336* | *0.06347* | *0.06495* | *0.06605* | *0.06668* |
| *28578.78476* | *0.04148* | *0.04443* | *0.04454* | *0.04515* | *0.04722* | *0.04879* | *0.05044* | *0.05219* | *0.05369* | *0.05506* | *0.05558* | *0.0578* | *0.05949* | *0.06156* | *0.06328* | *0.06337* | *0.06482* | *0.06595* | *0.06656* |
| *28572.24574* | *0.04143* | *0.04439* | *0.04448* | *0.04508* | *0.04717* | *0.04873* | *0.05039* | *0.05213* | *0.05363* | *0.05499* | *0.05549* | *0.0577* | *0.0594* | *0.06148* | *0.0632* | *0.06327* | *0.0647* | *0.06586* | *0.06644* |
| *28565.70971* | *0.04138* | *0.04435* | *0.04443* | *0.04502* | *0.04711* | *0.04866* | *0.05033* | *0.05207* | *0.05357* | *0.05493* | *0.05539* | *0.0576* | *0.05932* | *0.0614* | *0.06311* | *0.06317* | *0.06458* | *0.06577* | *0.06633* |
| *28559.17667* | *0.04133* | *0.04431* | *0.04437* | *0.04495* | *0.04706* | *0.0486* | *0.05028* | *0.05202* | *0.05352* | *0.05486* | *0.0553* | *0.05751* | *0.05924* | *0.06131* | *0.06303* | *0.06307* | *0.06446* | *0.06568* | *0.06621* |
| *28552.64662* | *0.04128* | *0.04428* | *0.04432* | *0.04489* | *0.047* | *0.04854* | *0.05023* | *0.05196* | *0.05346* | *0.0548* | *0.05521* | *0.05741* | *0.05915* | *0.06123* | *0.06295* | *0.06297* | *0.06434* | *0.06559* | *0.0661* |
| *28546.11956* | *0.04123* | *0.04424* | *0.04426* | *0.04483* | *0.04695* | *0.04848* | *0.05017* | *0.05191* | *0.0534* | *0.05474* | *0.05512* | *0.05732* | *0.05907* | *0.06115* | *0.06287* | *0.06288* | *0.06422* | *0.0655* | *0.06598* |
| *28539.59547* | *0.04118* | *0.04421* | *0.04421* | *0.04477* | *0.0469* | *0.04842* | *0.05012* | *0.05185* | *0.05335* | *0.05468* | *0.05503* | *0.05723* | *0.05899* | *0.06107* | *0.0628* | *0.06278* | *0.06411* | *0.06541* | *0.06587* |
| *28533.07437* | *0.04114* | *0.04417* | *0.04416* | *0.04471* | *0.04685* | *0.04836* | *0.05007* | *0.0518* | *0.0533* | *0.05462* | *0.05494* | *0.05714* | *0.05891* | *0.06099* | *0.06272* | *0.06269* | *0.06399* | *0.06532* | *0.06576* |
| *28526.55625* | *0.04109* | *0.04414* | *0.04411* | *0.04465* | *0.0468* | *0.0483* | *0.05002* | *0.05175* | *0.05324* | *0.05456* | *0.05485* | *0.05705* | *0.05883* | *0.06091* | *0.06264* | *0.0626* | *0.06388* | *0.06524* | *0.06565* |
| *28520.04111* | *0.04104* | *0.0441* | *0.04406* | *0.04459* | *0.04675* | *0.04825* | *0.04997* | *0.0517* | *0.05319* | *0.0545* | *0.05476* | *0.05696* | *0.05876* | *0.06084* | *0.06257* | *0.06251* | *0.06377* | *0.06515* | *0.06555* |
| *28513.52894* | *0.041* | *0.04407* | *0.04401* | *0.04454* | *0.0467* | *0.04819* | *0.04992* | *0.05165* | *0.05314* | *0.05444* | *0.05468* | *0.05687* | *0.05868* | *0.06076* | *0.0625* | *0.06242* | *0.06365* | *0.06507* | *0.06544* |
| *28507.01975* | *0.04095* | *0.04404* | *0.04396* | *0.04448* | *0.04665* | *0.04814* | *0.04987* | *0.0516* | *0.05309* | *0.05438* | *0.05459* | *0.05678* | *0.05861* | *0.06069* | *0.06242* | *0.06233* | *0.06354* | *0.06499* | *0.06533* |
| *28500.51352* | *0.04091* | *0.044* | *0.04391* | *0.04442* | *0.0466* | *0.04808* | *0.04982* | *0.05155* | *0.05304* | *0.05433* | *0.05451* | *0.0567* | *0.05853* | *0.06061* | *0.06235* | *0.06224* | *0.06343* | *0.06491* | *0.06523* |
| *28494.01027* | *0.04087* | *0.04397* | *0.04386* | *0.04437* | *0.04655* | *0.04803* | *0.04978* | *0.0515* | *0.05299* | *0.05427* | *0.05442* | *0.05661* | *0.05846* | *0.06054* | *0.06228* | *0.06216* | *0.06332* | *0.06483* | *0.06513* |
| *28487.50998* | *0.04082* | *0.04394* | *0.04381* | *0.04431* | *0.04651* | *0.04798* | *0.04973* | *0.05146* | *0.05294* | *0.05422* | *0.05434* | *0.05653* | *0.05838* | *0.06047* | *0.06221* | *0.06207* | *0.06322* | *0.06475* | *0.06502* |
| *28481.01266* | *0.04078* | *0.04391* | *0.04377* | *0.04426* | *0.04646* | *0.04792* | *0.04969* | *0.05141* | *0.05289* | *0.05416* | *0.05426* | *0.05644* | *0.05831* | *0.0604* | *0.06214* | *0.06199* | *0.06311* | *0.06467* | *0.06492* |
| *28474.5183* | *0.04074* | *0.04388* | *0.04372* | *0.04421* | *0.04642* | *0.04787* | *0.04964* | *0.05136* | *0.05285* | *0.05411* | *0.05418* | *0.05636* | *0.05824* | *0.06033* | *0.06207* | *0.0619* | *0.06301* | *0.06459* | *0.06482* |
| *28468.0269* | *0.0407* | *0.04385* | *0.04367* | *0.04415* | *0.04637* | *0.04782* | *0.0496* | *0.05132* | *0.0528* | *0.05406* | *0.0541* | *0.05628* | *0.05817* | *0.06026* | *0.06201* | *0.06182* | *0.0629* | *0.06451* | *0.06472* |
| *28461.53846* | *0.04066* | *0.04382* | *0.04363* | *0.0441* | *0.04633* | *0.04777* | *0.04955* | *0.05127* | *0.05275* | *0.05401* | *0.05402* | *0.0562* | *0.0581* | *0.06019* | *0.06194* | *0.06174* | *0.0628* | *0.06444* | *0.06463* |
| *28455.05298* | *0.04062* | *0.04379* | *0.04359* | *0.04405* | *0.04628* | *0.04772* | *0.04951* | *0.05123* | *0.05271* | *0.05396* | *0.05394* | *0.05612* | *0.05804* | *0.06013* | *0.06187* | *0.06166* | *0.0627* | *0.06437* | *0.06453* |
| *28448.57045* | *0.04058* | *0.04377* | *0.04354* | *0.044* | *0.04624* | *0.04767* | *0.04947* | *0.05119* | *0.05267* | *0.05391* | *0.05386* | *0.05604* | *0.05797* | *0.06006* | *0.06181* | *0.06158* | *0.0626* | *0.06429* | *0.06444* |
| *28442.09088* | *0.04054* | *0.04374* | *0.0435* | *0.04395* | *0.0462* | *0.04762* | *0.04943* | *0.05114* | *0.05262* | *0.05386* | *0.05379* | *0.05597* | *0.0579* | *0.05999* | *0.06175* | *0.0615* | *0.0625* | *0.06422* | *0.06434* |
| *28435.61425* | *0.0405* | *0.04371* | *0.04346* | *0.0439* | *0.04616* | *0.04758* | *0.04939* | *0.0511* | *0.05258* | *0.05381* | *0.05371* | *0.05589* | *0.05784* | *0.05993* | *0.06168* | *0.06142* | *0.0624* | *0.06415* | *0.06425* |
| *28429.14058* | *0.04047* | *0.04368* | *0.04342* | *0.04386* | *0.04612* | *0.04753* | *0.04935* | *0.05106* | *0.05254* | *0.05376* | *0.05364* | *0.05581* | *0.05777* | *0.05987* | *0.06162* | *0.06135* | *0.0623* | *0.06408* | *0.06416* |
| *28422.66985* | *0.04043* | *0.04366* | *0.04338* | *0.04381* | *0.04608* | *0.04749* | *0.04931* | *0.05102* | *0.0525* | *0.05371* | *0.05356* | *0.05574* | *0.05771* | *0.0598* | *0.06156* | *0.06127* | *0.0622* | *0.06401* | *0.06406* |
| *28416.20207* | *0.04039* | *0.04363* | *0.04334* | *0.04376* | *0.04604* | *0.04744* | *0.04927* | *0.05098* | *0.05245* | *0.05367* | *0.05349* | *0.05566* | *0.05765* | *0.05974* | *0.0615* | *0.0612* | *0.06211* | *0.06394* | *0.06397* |
| *28409.73723* | *0.04036* | *0.04361* | *0.0433* | *0.04372* | *0.046* | *0.0474* | *0.04923* | *0.05094* | *0.05241* | *0.05362* | *0.05342* | *0.05559* | *0.05758* | *0.05968* | *0.06144* | *0.06112* | *0.06201* | *0.06387* | *0.06389* |
| *28403.27533* | *0.04032* | *0.04358* | *0.04326* | *0.04367* | *0.04596* | *0.04735* | *0.04919* | *0.0509* | *0.05237* | *0.05358* | *0.05335* | *0.05552* | *0.05752* | *0.05962* | *0.06138* | *0.06105* | *0.06192* | *0.06381* | *0.0638* |
| *28396.81637* | *0.04029* | *0.04356* | *0.04322* | *0.04363* | *0.04592* | *0.04731* | *0.04915* | *0.05086* | *0.05234* | *0.05353* | *0.05328* | *0.05545* | *0.05746* | *0.05956* | *0.06132* | *0.06098* | *0.06182* | *0.06374* | *0.06371* |
| *28390.36035* | *0.04026* | *0.04353* | *0.04318* | *0.04358* | *0.04589* | *0.04727* | *0.04912* | *0.05083* | *0.0523* | *0.05349* | *0.05321* | *0.05538* | *0.0574* | *0.0595* | *0.06127* | *0.06091* | *0.06173* | *0.06368* | *0.06362* |
| *28383.90726* | *0.04022* | *0.04351* | *0.04314* | *0.04354* | *0.04585* | *0.04722* | *0.04908* | *0.05079* | *0.05226* | *0.05344* | *0.05314* | *0.05531* | *0.05734* | *0.05944* | *0.06121* | *0.06084* | *0.06164* | *0.06361* | *0.06354* |
| *28377.45711* | *0.04019* | *0.04349* | *0.04311* | *0.04349* | *0.04581* | *0.04718* | *0.04904* | *0.05075* | *0.05222* | *0.0534* | *0.05307* | *0.05524* | *0.05729* | *0.05939* | *0.06115* | *0.06077* | *0.06155* | *0.06355* | *0.06346* |
| *28371.00988* | *0.04016* | *0.04346* | *0.04307* | *0.04345* | *0.04578* | *0.04714* | *0.04901* | *0.05072* | *0.05219* | *0.05336* | *0.053* | *0.05517* | *0.05723* | *0.05933* | *0.0611* | *0.0607* | *0.06146* | *0.06349* | *0.06337* |
| *28364.56559* | *0.04012* | *0.04344* | *0.04304* | *0.04341* | *0.04574* | *0.0471* | *0.04897* | *0.05068* | *0.05215* | *0.05332* | *0.05294* | *0.0551* | *0.05717* | *0.05927* | *0.06105* | *0.06063* | *0.06137* | *0.06342* | *0.06329* |
| *28358.12422* | *0.04009* | *0.04342* | *0.043* | *0.04337* | *0.04571* | *0.04706* | *0.04894* | *0.05065* | *0.05211* | *0.05328* | *0.05287* | *0.05504* | *0.05712* | *0.05922* | *0.06099* | *0.06056* | *0.06129* | *0.06336* | *0.06321* |
| *28351.68578* | *0.04006* | *0.0434* | *0.04297* | *0.04333* | *0.04568* | *0.04702* | *0.04891* | *0.05061* | *0.05208* | *0.05324* | *0.05281* | *0.05497* | *0.05706* | *0.05916* | *0.06094* | *0.0605* | *0.0612* | *0.0633* | *0.06313* |
| *28345.25026* | *0.04003* | *0.04338* | *0.04293* | *0.04329* | *0.04564* | *0.04698* | *0.04887* | *0.05058* | *0.05204* | *0.0532* | *0.05274* | *0.05491* | *0.05701* | *0.05911* | *0.06089* | *0.06043* | *0.06112* | *0.06324* | *0.06305* |
| *28338.81766* | *0.04* | *0.04335* | *0.0429* | *0.04325* | *0.04561* | *0.04695* | *0.04884* | *0.05055* | *0.05201* | *0.05316* | *0.05268* | *0.05484* | *0.05695* | *0.05906* | *0.06084* | *0.06037* | *0.06103* | *0.06319* | *0.06297* |
| *28332.38798* | *0.03997* | *0.04333* | *0.04287* | *0.04321* | *0.04558* | *0.04691* | *0.04881* | *0.05051* | *0.05198* | *0.05312* | *0.05262* | *0.05478* | *0.0569* | *0.05901* | *0.06079* | *0.06031* | *0.06095* | *0.06313* | *0.06289* |
| *28325.96121* | *0.03994* | *0.04331* | *0.04283* | *0.04317* | *0.04555* | *0.04687* | *0.04878* | *0.05048* | *0.05194* | *0.05308* | *0.05256* | *0.05472* | *0.05685* | *0.05895* | *0.06074* | *0.06024* | *0.06087* | *0.06307* | *0.06282* |
| *28319.53736* | *0.03991* | *0.04329* | *0.0428* | *0.04313* | *0.04551* | *0.04684* | *0.04875* | *0.05045* | *0.05191* | *0.05305* | *0.0525* | *0.05466* | *0.0568* | *0.0589* | *0.06069* | *0.06018* | *0.06078* | *0.06302* | *0.06274* |
| *28313.11643* | *0.03988* | *0.04327* | *0.04277* | *0.0431* | *0.04548* | *0.0468* | *0.04872* | *0.05042* | *0.05188* | *0.05301* | *0.05244* | *0.0546* | *0.05674* | *0.05885* | *0.06064* | *0.06012* | *0.0607* | *0.06296* | *0.06267* |
| *28306.6984* | *0.03986* | *0.04326* | *0.04274* | *0.04306* | *0.04545* | *0.04676* | *0.04869* | *0.05039* | *0.05185* | *0.05297* | *0.05238* | *0.05453* | *0.05669* | *0.05881* | *0.06059* | *0.06006* | *0.06062* | *0.06291* | *0.06259* |
| *28300.28329* | *0.03983* | *0.04324* | *0.04271* | *0.04302* | *0.04542* | *0.04673* | *0.04866* | *0.05036* | *0.05182* | *0.05294* | *0.05232* | *0.05448* | *0.05665* | *0.05876* | *0.06055* | *0.06* | *0.06054* | *0.06285* | *0.06252* |
| *28293.87108* | *0.0398* | *0.04322* | *0.04268* | *0.04299* | *0.04539* | *0.0467* | *0.04863* | *0.05033* | *0.05179* | *0.0529* | *0.05226* | *0.05442* | *0.0566* | *0.05871* | *0.0605* | *0.05994* | *0.06047* | *0.0628* | *0.06245* |
| *28287.46177* | *0.03978* | *0.0432* | *0.04265* | *0.04295* | *0.04537* | *0.04666* | *0.0486* | *0.0503* | *0.05176* | *0.05287* | *0.0522* | *0.05436* | *0.05655* | *0.05866* | *0.06045* | *0.05988* | *0.06039* | *0.06275* | *0.06238* |
| *28281.05537* | *0.03975* | *0.04318* | *0.04262* | *0.04292* | *0.04534* | *0.04663* | *0.04857* | *0.05027* | *0.05173* | *0.05283* | *0.05214* | *0.0543* | *0.0565* | *0.05862* | *0.06041* | *0.05983* | *0.06031* | *0.0627* | *0.06231* |
| *28274.65187* | *0.03972* | *0.04316* | *0.04259* | *0.04288* | *0.04531* | *0.0466* | *0.04855* | *0.05024* | *0.0517* | *0.0528* | *0.05209* | *0.05424* | *0.05645* | *0.05857* | *0.06036* | *0.05977* | *0.06024* | *0.06265* | *0.06224* |
| *28268.25127* | *0.0397* | *0.04315* | *0.04256* | *0.04285* | *0.04528* | *0.04656* | *0.04852* | *0.05022* | *0.05167* | *0.05277* | *0.05203* | *0.05419* | *0.05641* | *0.05852* | *0.06032* | *0.05971* | *0.06016* | *0.0626* | *0.06217* |
| *28261.85357* | *0.03967* | *0.04313* | *0.04253* | *0.04282* | *0.04525* | *0.04653* | *0.04849* | *0.05019* | *0.05164* | *0.05273* | *0.05198* | *0.05413* | *0.05636* | *0.05848* | *0.06028* | *0.05966* | *0.06009* | *0.06255* | *0.0621* |
| *28255.45876* | *0.03965* | *0.04311* | *0.04251* | *0.04278* | *0.04523* | *0.0465* | *0.04847* | *0.05016* | *0.05161* | *0.0527* | *0.05192* | *0.05408* | *0.05632* | *0.05844* | *0.06023* | *0.05961* | *0.06001* | *0.0625* | *0.06203* |
| *28249.06685* | *0.03962* | *0.0431* | *0.04248* | *0.04275* | *0.0452* | *0.04647* | *0.04844* | *0.05014* | *0.05159* | *0.05267* | *0.05187* | *0.05403* | *0.05627* | *0.05839* | *0.06019* | *0.05955* | *0.05994* | *0.06245* | *0.06197* |
| *28242.67782* | *0.0396* | *0.04308* | *0.04245* | *0.04272* | *0.04517* | *0.04644* | *0.04841* | *0.05011* | *0.05156* | *0.05264* | *0.05182* | *0.05397* | *0.05623* | *0.05835* | *0.06015* | *0.0595* | *0.05987* | *0.0624* | *0.0619* |
| *28236.29169* | *0.03958* | *0.04306* | *0.04243* | *0.04269* | *0.04515* | *0.04641* | *0.04839* | *0.05008* | *0.05153* | *0.05261* | *0.05177* | *0.05392* | *0.05619* | *0.05831* | *0.06011* | *0.05945* | *0.0598* | *0.06236* | *0.06184* |
| *28229.90844* | *0.03955* | *0.04305* | *0.0424* | *0.04266* | *0.04512* | *0.04638* | *0.04836* | *0.05006* | *0.05151* | *0.05258* | *0.05171* | *0.05387* | *0.05614* | *0.05827* | *0.06007* | *0.05939* | *0.05973* | *0.06231* | *0.06177* |
| *28223.52808* | *0.03953* | *0.04303* | *0.04238* | *0.04263* | *0.0451* | *0.04635* | *0.04834* | *0.05003* | *0.05148* | *0.05255* | *0.05166* | *0.05382* | *0.0561* | *0.05822* | *0.06003* | *0.05934* | *0.05966* | *0.06226* | *0.06171* |
| *28217.1506* | *0.03951* | *0.04302* | *0.04235* | *0.04259* | *0.04508* | *0.04632* | *0.04832* | *0.05001* | *0.05146* | *0.05252* | *0.05161* | *0.05377* | *0.05606* | *0.05818* | *0.05999* | *0.05929* | *0.05959* | *0.06222* | *0.06165* |
| *28210.77601* | *0.03949* | *0.043* | *0.04233* | *0.04257* | *0.04505* | *0.0463* | *0.04829* | *0.04999* | *0.05143* | *0.05249* | *0.05156* | *0.05372* | *0.05602* | *0.05814* | *0.05995* | *0.05924* | *0.05952* | *0.06217* | *0.06159* |
| *28204.40429* | *0.03947* | *0.04299* | *0.0423* | *0.04254* | *0.04503* | *0.04627* | *0.04827* | *0.04996* | *0.05141* | *0.05246* | *0.05151* | *0.05367* | *0.05598* | *0.0581* | *0.05992* | *0.0592* | *0.05946* | *0.06213* | *0.06153* |
| *28198.03545* | *0.03944* | *0.04297* | *0.04228* | *0.04251* | *0.045* | *0.04624* | *0.04825* | *0.04994* | *0.05139* | *0.05244* | *0.05147* | *0.05362* | *0.05594* | *0.05807* | *0.05988* | *0.05915* | *0.05939* | *0.06209* | *0.06147* |
| *28191.66949* | *0.03942* | *0.04296* | *0.04226* | *0.04248* | *0.04498* | *0.04621* | *0.04822* | *0.04992* | *0.05136* | *0.05241* | *0.05142* | *0.05357* | *0.0559* | *0.05803* | *0.05984* | *0.0591* | *0.05932* | *0.06205* | *0.06141* |
| *28185.3064* | *0.0394* | *0.04295* | *0.04223* | *0.04245* | *0.04496* | *0.04619* | *0.0482* | *0.04989* | *0.05134* | *0.05238* | *0.05137* | *0.05352* | *0.05586* | *0.05799* | *0.05981* | *0.05905* | *0.05926* | *0.062* | *0.06135* |
| *28178.94618* | *0.03938* | *0.04293* | *0.04221* | *0.04242* | *0.04494* | *0.04616* | *0.04818* | *0.04987* | *0.05132* | *0.05236* | *0.05132* | *0.05348* | *0.05582* | *0.05795* | *0.05977* | *0.05901* | *0.0592* | *0.06196* | *0.06129* |
| *28172.58883* | *0.03936* | *0.04292* | *0.04219* | *0.0424* | *0.04492* | *0.04614* | *0.04816* | *0.04985* | *0.05129* | *0.05233* | *0.05128* | *0.05343* | *0.05579* | *0.05792* | *0.05974* | *0.05896* | *0.05913* | *0.06192* | *0.06123* |
| *28166.23435* | *0.03934* | *0.04291* | *0.04217* | *0.04237* | *0.04489* | *0.04611* | *0.04814* | *0.04983* | *0.05127* | *0.0523* | *0.05123* | *0.05338* | *0.05575* | *0.05788* | *0.0597* | *0.05892* | *0.05907* | *0.06188* | *0.06118* |
| *28159.88274* | *0.03932* | *0.04289* | *0.04214* | *0.04234* | *0.04487* | *0.04609* | *0.04812* | *0.04981* | *0.05125* | *0.05228* | *0.05119* | *0.05334* | *0.05571* | *0.05784* | *0.05967* | *0.05887* | *0.05901* | *0.06184* | *0.06112* |
| *28153.53399* | *0.0393* | *0.04288* | *0.04212* | *0.04232* | *0.04485* | *0.04606* | *0.0481* | *0.04979* | *0.05123* | *0.05225* | *0.05114* | *0.05329* | *0.05568* | *0.05781* | *0.05963* | *0.05883* | *0.05895* | *0.0618* | *0.06107* |
| *28147.1881* | *0.03929* | *0.04287* | *0.0421* | *0.04229* | *0.04483* | *0.04604* | *0.04808* | *0.04977* | *0.05121* | *0.05223* | *0.0511* | *0.05325* | *0.05564* | *0.05778* | *0.0596* | *0.05878* | *0.05889* | *0.06177* | *0.06101* |
| *28140.84507* | *0.03927* | *0.04286* | *0.04208* | *0.04227* | *0.04481* | *0.04602* | *0.04806* | *0.04975* | *0.05119* | *0.0522* | *0.05106* | *0.05321* | *0.05561* | *0.05774* | *0.05957* | *0.05874* | *0.05883* | *0.06173* | *0.06096* |
| *28134.5049* | *0.03925* | *0.04285* | *0.04206* | *0.04224* | *0.04479* | *0.04599* | *0.04804* | *0.04973* | *0.05117* | *0.05218* | *0.05101* | *0.05316* | *0.05557* | *0.05771* | *0.05954* | *0.0587* | *0.05877* | *0.06169* | *0.06091* |
| *28128.16759* | *0.03923* | *0.04283* | *0.04204* | *0.04222* | *0.04477* | *0.04597* | *0.04802* | *0.04971* | *0.05115* | *0.05216* | *0.05097* | *0.05312* | *0.05554* | *0.05767* | *0.0595* | *0.05866* | *0.05871* | *0.06165* | *0.06085* |
| *28121.83313* | *0.03921* | *0.04282* | *0.04202* | *0.04219* | *0.04475* | *0.04595* | *0.048* | *0.04969* | *0.05113* | *0.05213* | *0.05093* | *0.05308* | *0.05551* | *0.05764* | *0.05947* | *0.05862* | *0.05865* | *0.06162* | *0.0608* |
| *28115.50152* | *0.0392* | *0.04281* | *0.042* | *0.04217* | *0.04473* | *0.04593* | *0.04798* | *0.04967* | *0.05111* | *0.05211* | *0.05089* | *0.05304* | *0.05547* | *0.05761* | *0.05944* | *0.05858* | *0.05859* | *0.06158* | *0.06075* |
| *28109.17276* | *0.03918* | *0.0428* | *0.04198* | *0.04215* | *0.04472* | *0.0459* | *0.04797* | *0.04965* | *0.05109* | *0.05209* | *0.05085* | *0.053* | *0.05544* | *0.05758* | *0.05941* | *0.05854* | *0.05854* | *0.06155* | *0.0607* |
| *28102.84685* | *0.03916* | *0.04279* | *0.04196* | *0.04212* | *0.0447* | *0.04588* | *0.04795* | *0.04963* | *0.05107* | *0.05207* | *0.05081* | *0.05296* | *0.05541* | *0.05755* | *0.05938* | *0.0585* | *0.05848* | *0.06151* | *0.06065* |
| *28096.52379* | *0.03915* | *0.04278* | *0.04195* | *0.0421* | *0.04468* | *0.04586* | *0.04793* | *0.04962* | *0.05105* | *0.05205* | *0.05077* | *0.05292* | *0.05538* | *0.05752* | *0.05935* | *0.05846* | *0.05843* | *0.06148* | *0.0606* |
| *28090.20358* | *0.03913* | *0.04277* | *0.04193* | *0.04208* | *0.04466* | *0.04584* | *0.04791* | *0.0496* | *0.05104* | *0.05202* | *0.05073* | *0.05288* | *0.05535* | *0.05749* | *0.05932* | *0.05842* | *0.05837* | *0.06144* | *0.06055* |
| *28083.8862* | *0.03911* | *0.04276* | *0.04191* | *0.04206* | *0.04465* | *0.04582* | *0.0479* | *0.04958* | *0.05102* | *0.052* | *0.05069* | *0.05284* | *0.05532* | *0.05746* | *0.0593* | *0.05838* | *0.05832* | *0.06141* | *0.06051* |
| *28077.57167* | *0.0391* | *0.04275* | *0.04189* | *0.04203* | *0.04463* | *0.0458* | *0.04788* | *0.04956* | *0.051* | *0.05198* | *0.05065* | *0.0528* | *0.05528* | *0.05743* | *0.05927* | *0.05834* | *0.05826* | *0.06138* | *0.06046* |
| *28071.25998* | *0.03908* | *0.04274* | *0.04187* | *0.04201* | *0.04461* | *0.04578* | *0.04786* | *0.04955* | *0.05098* | *0.05196* | *0.05062* | *0.05277* | *0.05526* | *0.0574* | *0.05924* | *0.05831* | *0.05821* | *0.06135* | *0.06041* |
| *28064.95112* | *0.03907* | *0.04273* | *0.04186* | *0.04199* | *0.0446* | *0.04576* | *0.04785* | *0.04953* | *0.05097* | *0.05194* | *0.05058* | *0.05273* | *0.05523* | *0.05737* | *0.05921* | *0.05827* | *0.05816* | *0.06132* | *0.06037* |
| *28058.6451* | *0.03905* | *0.04272* | *0.04184* | *0.04197* | *0.04458* | *0.04574* | *0.04783* | *0.04951* | *0.05095* | *0.05192* | *0.05054* | *0.05269* | *0.0552* | *0.05734* | *0.05919* | *0.05824* | *0.05811* | *0.06128* | *0.06032* |
| *28052.34191* | *0.03904* | *0.04271* | *0.04182* | *0.04195* | *0.04456* | *0.04572* | *0.04782* | *0.0495* | *0.05093* | *0.0519* | *0.05051* | *0.05266* | *0.05517* | *0.05731* | *0.05916* | *0.0582* | *0.05806* | *0.06125* | *0.06028* |
| *28046.04155* | *0.03902* | *0.0427* | *0.04181* | *0.04193* | *0.04455* | *0.0457* | *0.0478* | *0.04948* | *0.05092* | *0.05189* | *0.05047* | *0.05262* | *0.05514* | *0.05729* | *0.05913* | *0.05817* | *0.05801* | *0.06122* | *0.06023* |
| *28039.74402* | *0.03901* | *0.04269* | *0.04179* | *0.04191* | *0.04453* | *0.04569* | *0.04779* | *0.04947* | *0.0509* | *0.05187* | *0.05044* | *0.05259* | *0.05511* | *0.05726* | *0.05911* | *0.05813* | *0.05796* | *0.06119* | *0.06019* |
| *28033.44932* | *0.039* | *0.04268* | *0.04178* | *0.04189* | *0.04452* | *0.04567* | *0.04777* | *0.04945* | *0.05089* | *0.05185* | *0.0504* | *0.05255* | *0.05509* | *0.05723* | *0.05908* | *0.0581* | *0.05791* | *0.06116* | *0.06014* |
| *28027.15745* | *0.03898* | *0.04267* | *0.04176* | *0.04187* | *0.0445* | *0.04565* | *0.04776* | *0.04944* | *0.05087* | *0.05183* | *0.05037* | *0.05252* | *0.05506* | *0.05721* | *0.05906* | *0.05806* | *0.05786* | *0.06113* | *0.0601* |
| *28020.86839* | *0.03897* | *0.04267* | *0.04175* | *0.04185* | *0.04449* | *0.04563* | *0.04774* | *0.04942* | *0.05086* | *0.05181* | *0.05033* | *0.05248* | *0.05503* | *0.05718* | *0.05903* | *0.05803* | *0.05781* | *0.06111* | *0.06006* |
| *28014.58216* | *0.03896* | *0.04266* | *0.04173* | *0.04184* | *0.04447* | *0.04562* | *0.04773* | *0.04941* | *0.05084* | *0.0518* | *0.0503* | *0.05245* | *0.05501* | *0.05716* | *0.05901* | *0.058* | *0.05776* | *0.06108* | *0.06002* |
| *28008.29876* | *0.03894* | *0.04265* | *0.04172* | *0.04182* | *0.04446* | *0.0456* | *0.04772* | *0.0494* | *0.05083* | *0.05178* | *0.05027* | *0.05242* | *0.05498* | *0.05713* | *0.05898* | *0.05797* | *0.05772* | *0.06105* | *0.05998* |
| *28002.01816* | *0.03893* | *0.04264* | *0.0417* | *0.0418* | *0.04445* | *0.04558* | *0.0477* | *0.04938* | *0.05081* | *0.05176* | *0.05023* | *0.05238* | *0.05495* | *0.05711* | *0.05896* | *0.05794* | *0.05767* | *0.06102* | *0.05994* |
| *27995.74039* | *0.03892* | *0.04263* | *0.04169* | *0.04178* | *0.04443* | *0.04557* | *0.04769* | *0.04937* | *0.0508* | *0.05174* | *0.0502* | *0.05235* | *0.05493* | *0.05708* | *0.05894* | *0.0579* | *0.05763* | *0.061* | *0.0599* |
| *27989.46543* | *0.03891* | *0.04263* | *0.04167* | *0.04176* | *0.04442* | *0.04555* | *0.04768* | *0.04936* | *0.05079* | *0.05173* | *0.05017* | *0.05232* | *0.05491* | *0.05706* | *0.05892* | *0.05787* | *0.05758* | *0.06097* | *0.05986* |
| *27983.19328* | *0.03889* | *0.04262* | *0.04166* | *0.04175* | *0.04441* | *0.04553* | *0.04766* | *0.04934* | *0.05077* | *0.05171* | *0.05014* | *0.05229* | *0.05488* | *0.05703* | *0.05889* | *0.05784* | *0.05754* | *0.06094* | *0.05982* |
| *27976.92394* | *0.03888* | *0.04261* | *0.04165* | *0.04173* | *0.04439* | *0.04552* | *0.04765* | *0.04933* | *0.05076* | *0.0517* | *0.05011* | *0.05226* | *0.05486* | *0.05701* | *0.05887* | *0.05781* | *0.05749* | *0.06092* | *0.05978* |
| *27970.65741* | *0.03887* | *0.0426* | *0.04163* | *0.04171* | *0.04438* | *0.0455* | *0.04764* | *0.04932* | *0.05075* | *0.05168* | *0.05008* | *0.05223* | *0.05483* | *0.05699* | *0.05885* | *0.05778* | *0.05745* | *0.06089* | *0.05974* |
| *27964.39368* | *0.03886* | *0.0426* | *0.04162* | *0.0417* | *0.04437* | *0.04549* | *0.04763* | *0.0493* | *0.05073* | *0.05167* | *0.05005* | *0.0522* | *0.05481* | *0.05697* | *0.05883* | *0.05775* | *0.05741* | *0.06087* | *0.0597* |
| *27958.13277* | *0.03885* | *0.04259* | *0.04161* | *0.04168* | *0.04435* | *0.04547* | *0.04762* | *0.04929* | *0.05072* | *0.05165* | *0.05002* | *0.05217* | *0.05479* | *0.05694* | *0.05881* | *0.05773* | *0.05736* | *0.06084* | *0.05967* |
| *27951.87465* | *0.03884* | *0.04258* | *0.0416* | *0.04166* | *0.04434* | *0.04546* | *0.0476* | *0.04928* | *0.05071* | *0.05164* | *0.04999* | *0.05214* | *0.05477* | *0.05692* | *0.05879* | *0.0577* | *0.05732* | *0.06082* | *0.05963* |
| *27945.61934* | *0.03882* | *0.04258* | *0.04158* | *0.04165* | *0.04433* | *0.04545* | *0.04759* | *0.04927* | *0.0507* | *0.05162* | *0.04996* | *0.05211* | *0.05474* | *0.0569* | *0.05877* | *0.05767* | *0.05728* | *0.06079* | *0.0596* |
| *27939.36682* | *0.03881* | *0.04257* | *0.04157* | *0.04163* | *0.04432* | *0.04543* | *0.04758* | *0.04926* | *0.05068* | *0.05161* | *0.04993* | *0.05208* | *0.05472* | *0.05688* | *0.05875* | *0.05764* | *0.05724* | *0.06077* | *0.05956* |
| *27933.1171* | *0.0388* | *0.04256* | *0.04156* | *0.04162* | *0.04431* | *0.04542* | *0.04757* | *0.04925* | *0.05067* | *0.05159* | *0.0499* | *0.05206* | *0.0547* | *0.05686* | *0.05873* | *0.05762* | *0.0572* | *0.06075* | *0.05953* |
| *27926.87018* | *0.03879* | *0.04256* | *0.04155* | *0.0416* | *0.0443* | *0.0454* | *0.04756* | *0.04923* | *0.05066* | *0.05158* | *0.04987* | *0.05203* | *0.05468* | *0.05684* | *0.05871* | *0.05759* | *0.05716* | *0.06073* | *0.05949* |
| *27920.62605* | *0.03878* | *0.04255* | *0.04154* | *0.04159* | *0.04428* | *0.04539* | *0.04755* | *0.04922* | *0.05065* | *0.05157* | *0.04985* | *0.052* | *0.05466* | *0.05682* | *0.05869* | *0.05756* | *0.05712* | *0.0607* | *0.05946* |
| *27914.38471* | *0.03877* | *0.04254* | *0.04153* | *0.04157* | *0.04427* | *0.04538* | *0.04754* | *0.04921* | *0.05064* | *0.05155* | *0.04982* | *0.05197* | *0.05464* | *0.0568* | *0.05867* | *0.05754* | *0.05708* | *0.06068* | *0.05942* |
| *27908.14616* | *0.03876* | *0.04254* | *0.04151* | *0.04156* | *0.04426* | *0.04536* | *0.04753* | *0.0492* | *0.05063* | *0.05154* | *0.04979* | *0.05195* | *0.05462* | *0.05678* | *0.05865* | *0.05751* | *0.05704* | *0.06066* | *0.05939* |
| *27901.9104* | *0.03875* | *0.04253* | *0.0415* | *0.04155* | *0.04425* | *0.04535* | *0.04752* | *0.04919* | *0.05062* | *0.05153* | *0.04977* | *0.05192* | *0.0546* | *0.05676* | *0.05863* | *0.05749* | *0.057* | *0.06064* | *0.05936* |
| *27895.67743* | *0.03874* | *0.04253* | *0.04149* | *0.04153* | *0.04424* | *0.04534* | *0.04751* | *0.04918* | *0.05061* | *0.05151* | *0.04974* | *0.0519* | *0.05458* | *0.05674* | *0.05862* | *0.05746* | *0.05697* | *0.06062* | *0.05932* |
| *27889.44724* | *0.03873* | *0.04252* | *0.04148* | *0.04152* | *0.04423* | *0.04533* | *0.0475* | *0.04917* | *0.0506* | *0.0515* | *0.04972* | *0.05187* | *0.05456* | *0.05672* | *0.0586* | *0.05744* | *0.05693* | *0.0606* | *0.05929* |
| *27883.21983* | *0.03873* | *0.04252* | *0.04147* | *0.04151* | *0.04422* | *0.04532* | *0.04749* | *0.04916* | *0.05059* | *0.05149* | *0.04969* | *0.05185* | *0.05454* | *0.0567* | *0.05858* | *0.05741* | *0.05689* | *0.06058* | *0.05926* |
| *27876.9952* | *0.03872* | *0.04251* | *0.04146* | *0.04149* | *0.04421* | *0.0453* | *0.04748* | *0.04915* | *0.05058* | *0.05148* | *0.04967* | *0.05182* | *0.05452* | *0.05669* | *0.05856* | *0.05739* | *0.05686* | *0.06056* | *0.05923* |
| *27870.77335* | *0.03871* | *0.04251* | *0.04145* | *0.04148* | *0.0442* | *0.04529* | *0.04747* | *0.04914* | *0.05057* | *0.05146* | *0.04964* | *0.0518* | *0.0545* | *0.05667* | *0.05855* | *0.05737* | *0.05682* | *0.06054* | *0.0592* |
| *27864.55428* | *0.0387* | *0.0425* | *0.04144* | *0.04147* | *0.04419* | *0.04528* | *0.04746* | *0.04913* | *0.05056* | *0.05145* | *0.04962* | *0.05177* | *0.05448* | *0.05665* | *0.05853* | *0.05734* | *0.05678* | *0.06052* | *0.05917* |
| *27858.33798* | *0.03869* | *0.04249* | *0.04143* | *0.04145* | *0.04418* | *0.04527* | *0.04745* | *0.04912* | *0.05055* | *0.05144* | *0.04959* | *0.05175* | *0.05447* | *0.05663* | *0.05851* | *0.05732* | *0.05675* | *0.0605* | *0.05914* |
| *27852.12446* | *0.03868* | *0.04249* | *0.04142* | *0.04144* | *0.04417* | *0.04526* | *0.04744* | *0.04912* | *0.05054* | *0.05143* | *0.04957* | *0.05173* | *0.05445* | *0.05662* | *0.0585* | *0.0573* | *0.05672* | *0.06048* | *0.05911* |
| *27845.9137* | *0.03867* | *0.04248* | *0.04141* | *0.04143* | *0.04417* | *0.04525* | *0.04743* | *0.04911* | *0.05053* | *0.05142* | *0.04955* | *0.0517* | *0.05443* | *0.0566* | *0.05848* | *0.05728* | *0.05668* | *0.06046* | *0.05908* |
| *27839.70572* | *0.03866* | *0.04248* | *0.0414* | *0.04142* | *0.04416* | *0.04524* | *0.04743* | *0.0491* | *0.05052* | *0.05141* | *0.04952* | *0.05168* | *0.05441* | *0.05658* | *0.05847* | *0.05726* | *0.05665* | *0.06044* | *0.05905* |
| *27833.5005* | *0.03866* | *0.04248* | *0.04139* | *0.04141* | *0.04415* | *0.04523* | *0.04742* | *0.04909* | *0.05051* | *0.0514* | *0.0495* | *0.05166* | *0.0544* | *0.05657* | *0.05845* | *0.05723* | *0.05661* | *0.06042* | *0.05903* |
| *27827.29805* | *0.03865* | *0.04247* | *0.04139* | *0.04139* | *0.04414* | *0.04522* | *0.04741* | *0.04908* | *0.0505* | *0.05139* | *0.04948* | *0.05164* | *0.05438* | *0.05655* | *0.05844* | *0.05721* | *0.05658* | *0.06041* | *0.059* |
| *27821.09836* | *0.03864* | *0.04247* | *0.04138* | *0.04138* | *0.04413* | *0.04521* | *0.0474* | *0.04907* | *0.0505* | *0.05138* | *0.04946* | *0.05161* | *0.05436* | *0.05654* | *0.05842* | *0.05719* | *0.05655* | *0.06039* | *0.05897* |
| *27814.90144* | *0.03863* | *0.04246* | *0.04137* | *0.04137* | *0.04412* | *0.0452* | *0.04739* | *0.04906* | *0.05049* | *0.05137* | *0.04943* | *0.05159* | *0.05435* | *0.05652* | *0.05841* | *0.05717* | *0.05652* | *0.06037* | *0.05894* |
| *27808.70727* | *0.03863* | *0.04246* | *0.04136* | *0.04136* | *0.04411* | *0.04519* | *0.04739* | *0.04906* | *0.05048* | *0.05136* | *0.04941* | *0.05157* | *0.05433* | *0.0565* | *0.05839* | *0.05715* | *0.05649* | *0.06035* | *0.05892* |
| *27802.51586* | *0.03862* | *0.04245* | *0.04135* | *0.04135* | *0.04411* | *0.04518* | *0.04738* | *0.04905* | *0.05047* | *0.05135* | *0.04939* | *0.05155* | *0.05432* | *0.05649* | *0.05838* | *0.05713* | *0.05645* | *0.06034* | *0.05889* |
| *27796.32721* | *0.03861* | *0.04245* | *0.04134* | *0.04134* | *0.0441* | *0.04517* | *0.04737* | *0.04904* | *0.05046* | *0.05134* | *0.04937* | *0.05153* | *0.0543* | *0.05648* | *0.05837* | *0.05711* | *0.05642* | *0.06032* | *0.05886* |
| *27790.14132* | *0.0386* | *0.04244* | *0.04134* | *0.04133* | *0.04409* | *0.04516* | *0.04736* | *0.04903* | *0.05046* | *0.05133* | *0.04935* | *0.05151* | *0.05429* | *0.05646* | *0.05835* | *0.05709* | *0.05639* | *0.0603* | *0.05884* |
| *27783.95817* | *0.0386* | *0.04244* | *0.04133* | *0.04132* | *0.04408* | *0.04515* | *0.04736* | *0.04903* | *0.05045* | *0.05132* | *0.04933* | *0.05149* | *0.05427* | *0.05645* | *0.05834* | *0.05707* | *0.05636* | *0.06029* | *0.05881* |
| *27777.77778* | *0.03859* | *0.04244* | *0.04132* | *0.04131* | *0.04408* | *0.04514* | *0.04735* | *0.04902* | *0.05044* | *0.05131* | *0.04931* | *0.05147* | *0.05426* | *0.05643* | *0.05833* | *0.05706* | *0.05633* | *0.06027* | *0.05879* |
