# Supplementary material for: Additional data for evaluation of the excited state dipole moments of anisole
Source: Data Brief. 2018 Oct 3;21:313–5. doi: 10.1016/j.dib.2018.09.110 (PMC6197573; doi:10.1016/j.dib.2018.09.110)
Supplement: Supplementary file 7 — Supplementary material [file mmc7.docx]

*Table S5: Cartesian coordinates of anisole S_0_ in bohr units from the CC2/cc-pVTZ calculations using the Turbomole program package.*

*C 0.29514957 0.00251005 0.09552629*

*C 0.36325862 0.00279133 2.71969272*

*C 2.68805953 0.00175078 3.98506250*

*C 4.94040276 0.00032928 2.60276573*

*C 4.83942652 -0.00012598 -0.03836629*

*C 2.53570432 0.00098059 -1.30547147*

*O 2.55346006 0.00236519 6.56185082*

*H 4.90204262 0.00169625 7.86565457*

*H -1.35088692 0.00375032 3.83282143*

*H 6.75429875 -0.00026448 3.53610674*

*H 6.58995725 -0.00133602 -1.09559467*

*H 2.47933655 0.00050984 -3.34751819*

*H 4.43356120 0.00226577 9.86196638*

*H 6.00524213 -1.68222636 7.41458968*

*H 6.00654007 1.68459697 7.41394908*

*H -1.51284126 0.00335154 -0.85942358*
