# Supplementary material for: Additional data for evaluation of the excited state dipole moments of anisole
Source: Data Brief. 2018 Oct 3;21:313–5. doi: 10.1016/j.dib.2018.09.110 (PMC6197573; doi:10.1016/j.dib.2018.09.110)
Supplement: Supplementary file 8 — Supplementary material [file mmc8.docx]

*Table S6: Cartesian coordinates of anisole S_1_ in bohr units from the CC2/cc-pVTZ calculations using the Turbomole program package.*

C 0.20805155 0.00235130 0.05644450

C 0.29346900 0.00279969 2.76223243

C 2.69816034 0.00182905 3.96742708

C 5.04323249 0.00030120 2.61427622

C 4.91727699 -0.00013485 -0.08356094

C 2.51814961 0.00094956 -1.30692729

O 2.57899068 0.00253942 6.51833936

C 4.91378926 0.00168473 7.89861695

H -1.38115088 0.00386696 3.92743255

H 6.83592273 -0.00036947 3.57985896

H 6.62977115 -0.00119659 -1.19338397

H 2.46278741 0.00068919 -3.35167403

H 4.37382036 0.00240575 9.87495599

H 6.00247092 -1.68671623 7.45610529

H 6.00414126 1.68880893 7.45534136

H -1.57617111 0.00313640 -0.93187272
